# Constrain as an advantage: selective metal-templated synthesis of sterically hindered phthalocyanine photosensitizers and their application for light-triggered release from liposomes

**DOI:** 10.1039/d6ra03003c

**Published:** 2026-07-02

**Authors:** Aleksandr Penni, Riikka Rimmistö, Inka Ojala, Sebastian Pätsch, Benedict Josua Elvers, Carola Schulzke, Timo Laaksonen, Nikita Durandin, Alexander Efimov

**Affiliations:** a Tampere University, Faculty of Engineering and Natural Sciences Korkeakoulunkatu 8 33720 Tampere Finland alexandre.efimov@tuni.fi; b University of Helsinki, Faculty of Pharmacy Viikinkaari 5 E 00790 Helsinki Finland; c University of Greifswald, Institute of Biochemistry, Bioinorganic Chemistry Felix-Hausdorff-Str. 4 17489 Greifswald Germany

## Abstract

Sterically constrained positional isomers of a non-aggregating α-aryl tetrasubstituted phthalocyanine were selectively obtained in high yields for the first time *via* a Mg-templated synthesis route. X-ray diffraction studies confirmed the structural assignments performed using NMR spectroscopy. Pd^II^ complexes of the three regioisomerically pure phthalocyanines (Pcs) with *C*_4h_, *C*_s_, and *C*_2v_ symmetry (denoted *windmill*, *dragon*, and *frog*, respectively) were employed as photosensitizers in a comparative study of light-triggered cargo release from liposomes. While the three PdPcs demonstrated similarly high singlet oxygen generation quantum yields in solution, their performance in the controlled rupture of ROS-sensitive liposomal carriers was considerably different. The dragon and frog PdPcs showed a drastically higher efficiency in the light-induced cargo release than the windmill PdPc regioisomer. This behavior was attributed to the enhanced ability of the less symmetrical *C*_s_ and *C*_2v_ isomers to incorporate into the lipid bilayers, whereas the more symmetrical *C*_4h_ complex forms its own, separate crystalline phase.

## Abstract 2

Sterically constrained positional isomers of a non-aggregating α-aryl tetrasubstituted phthalocyanine were selectively obtained in high yields for the first time *via* a Mg-templated synthesis route.

## Graphical abstract

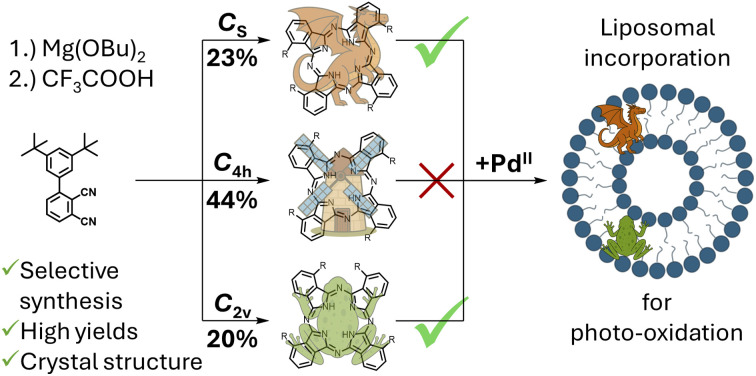

## Introduction

**Scheme 1:**
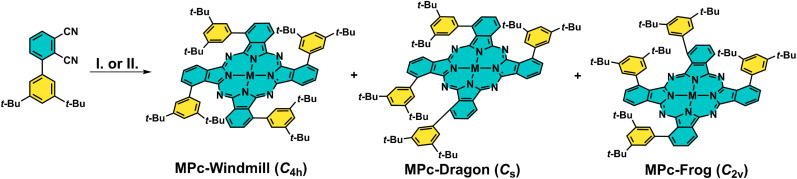
Metal-promoted phthalonitrile cyclotetramerization reaction. Conditions I: MX_*n*_, DBU, 1-pentanol, 150 °C, Ar. Conditions II: 1. Mg(OBu)_2_, 1-butanol, 130 °C, Ar, 17 h; 2. TFA, CHCl_3_, 65 °C, Ar, 2 h.

Cyclotetramerization of monosubstituted phthalonitriles generates mixtures of regioisomeric phthalocyanines (Pcs).^1^ In many cases, the isomers are not separable by conventional techniques and are not isolated in pure form.^2^ If a sufficiently large substituent is placed at the α-phthalo position of the phthalonitrile, the resulting steric hindrance may force the phthalonitrile monomers to tetramerize in an orderly manner, especially at moderate temperatures, producing the sterically unconstrained *C*_4h_-symmetric isomer in decent yield.^3–7^ Thus, in the classical lithium alkoxide-promoted tetramerization reaction of a phthalonitrile bearing a bulky 3,5-di-(*tert*-butyl)phenyl α-phthalo substituent, the free-base *C*_4h_ Pc regioisomer (*windmill*) is formed with the highest yield of *ca.* 50%.^8^ In contrast, due to the steric crowding of the sterically demanding aryl α-phthalo substituents, the amounts of the *C*_s_ (*dragon*) and *C*_2v_ (*frog*) positional isomers obtained are minor at about 3% combined yield (the three regioisomers are shown as their respective metal complexes in Scheme 1).^8^ The limited amounts of sterically congested Pc regioisomers produced *via* traditional routes make their detailed studies and potential applications challenging. Moreover, methods for the selective and efficient synthesis of such compounds have not been reported to date.

Previously, we have demonstrated that the three Pc regioisomers (*windmill*, *dragon*, and *frog*) exhibit substantially different spectral and electrochemical properties.^8^ However, a method for the directed synthesis of the sterically constrained Pc regioisomers would enable more extensive studies of the properties of these compounds. Regioisomeric mixtures of metal phthalocyanines (MPcs) can be effectively produced by phthalonitrile tetramerization in the presence of metal salts and organic bases like DBN, DBU, or DMAE.^9–15^ It is known that the metal ion plays a crucial role in the cyclotetramerization of phthalonitrile monomers into a Pc macrocycle.^1^ Hence, we reasoned that the yields of the sterically constrained *dragon C*_s_ and *frog C*_2v_ isomers can be increased *via* appropriate metal-templated Pc ring construction. To this end, we set out to probe the influence of the nature of the metal template on the regioisomeric distribution of the Pcs formed. In addition, we intended to explore post-synthetic removal of the templating metal, as this would enable the targeted synthesis of different MPcs of the desired regioisomer type by metallation of the individual free-base Pc precursors.^16,17^

One of the most important applications of MPcs is photosensitization, particularly in photodynamic therapy (PDT) and light-triggered drug delivery.^18–28^ Pd^II^Pc complexes stand out in this regard owing to their pronounced ability to produce ^1^O_2_ and other reactive oxygen species (ROS) upon irradiation with visible light.^29–37^ Our recent studies on light-induced drug release using MPcs and ROS-sensitive liposomes showed that the photosensitization of liposomal carriers is a complex interplay of oxidative and thermal processes and that the photosensitization efficiency depends significantly on the photophysical properties and the way of structural incorporation of the photosensitizer into the liposomal bilayer.^38–40^ Since regioisomeric free-base Pcs as well as their metal complexes were found to possess dissimilar solubilities, optical properties, and redox potentials,^8^ we hypothesized that, for regioisomeric PdPcs, distinct behavior in liposomal membranes may be anticipated. This prompted us to investigate the application of a series of regioisomeric PdPcs as photosensitizers for light-triggered cargo release from ROS-sensitive liposomal carriers.

## Results and discussion

### Synthesis

**Scheme 2:**
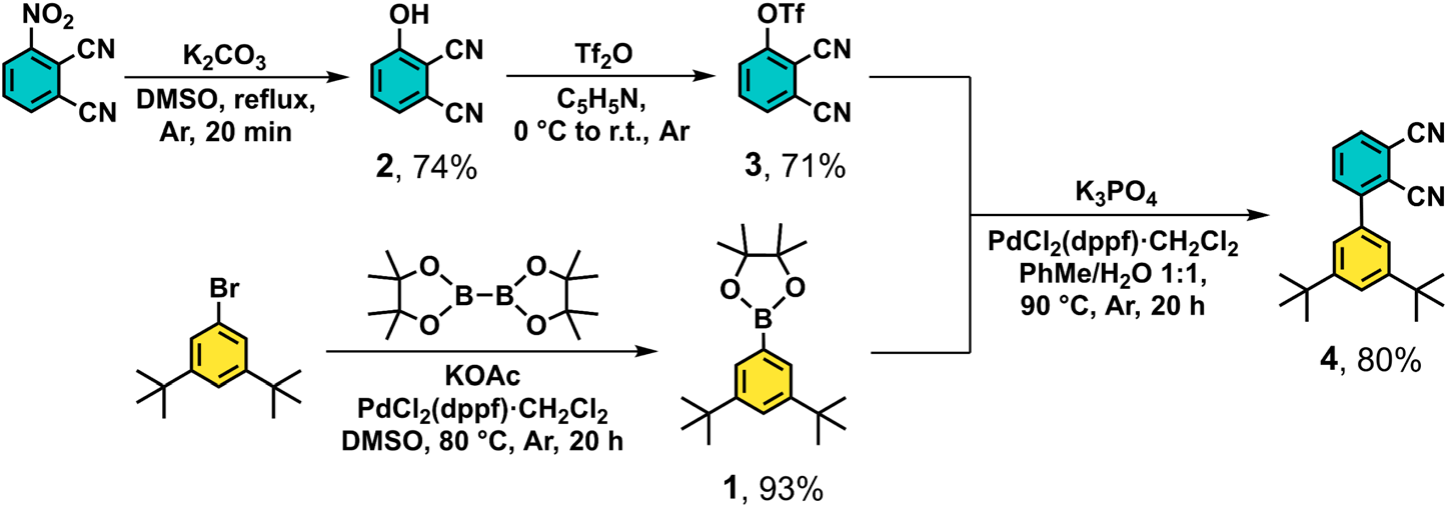
Synthesis of 3,5-di-(*tert*-butyl)phenyl-2,3-dicyanobenzene (4).

The starting phthalonitrile, 3,5-di-(*tert*-butyl)phenyl-2,3-dicyanobenzene (4), was prepared in multi-gram quantity by a four-step convergent synthesis (Scheme 2). Although its synthesis has previously been described, in this work, the reported procedures^41^ were optimized and scaled up. For instance, contrary to the published method,^41,42^ the use of sodium nitrite in the conversion of 3-nitrophthalonitrile to 3-hydroxyphthalonitrile 2 proved to be unnecessary, and the elaborate isolation and purification procedure could be replaced by a quick and facile work-up that furnished 3-hydroxyphthalonitrile 2 on a gram scale in good yield and sufficient purity.

A well-established route to metal phthalocyanines (MPcs) comprises a phthalonitrile cyclotetramerization reaction performed at an elevated temperature in a high-boiling primary alcohol in the presence of a metal salt and a strong organic base like DBU.^9–11,13^ Somewhat surprisingly, we found that, for phthalonitrile 4, good results with this procedure could be obtained only in the case of copper(ii) salts (Scheme 1 and Table 1). In the reaction with CuBr_2_, the ratio of the *windmill*/*dragon*/*frog* regiosiomers was found to be 1 : 1.4 : 1.2, respectively (18 : 25 : 21 per cent isolated gravimetric yields, 64% combined yield). Very similar regioisomeric distribution and yields were obtained in the reaction with CuCl_2_·2H_2_O (Table 1). The fourth regioisomer of *D*_2h_ point group symmetry was not observed in any of the reactions carried out with phthalonitrile 4. As was the case for the free-base Pcs,^8^CuPc-Windmill could be easily isolated by washing the mixture of the regioisomeric CuPcs with hexane. The hexane-insoluble residue comprised individual CuPc-Windmill, whereas CuPc-Dragon and CuPc-Frog were freely soluble in hexane and were separated by column chromatography on silica gel.

**Table 1:** Results of the metal-promoted phthalonitrile cyclotetramerization reaction (Scheme 1). W = MPc-Windmill, D = MPc-Dragon, F = MPc-Frog

Activator	Yield (%) (W : D : F)	W : D : F ratio
LiOBu	42 combined^8^	*Ca.* 1 : 0.05 : 0.05
Metal-free^a^	Trace	—
AgNO_3_^a^	No Pc formation	—
Co(OAc)_2_·4H_2_O^a^	11 : 11 : 9^b^	1 : 1 : 0.82^b^
Cs_2_CO_3_^a^	No Pc formation	—
CuBr_2_^a^	18 : 25 : 21	1 : 1.39 : 1.17
CuCl_2_·2H_2_O^a^	20 : 27 : 16	1 : 1.35 : 0.8
FeBr_2_ or FeCl_2_·4H_2_O^a^	No Pcs could be isolated	—
LiBH_4_^a^^,^^c^	26 combined	nd
LiBr^a^^,^^c^	18 : 2 : 5	1 : 0.11 : 0.28
Li_2_CO_3_^a^	Trace	—
MgCl_2_^a^	Trace	—
Mn(OAc)_2_·4H_2_O^a^	No Pc formation	—
NiCl_2_^a^	7 combined^b^	*Ca.* 1 : 2 : 1^b^
PdCl_2_^a^	9 combined^d^	nd
Zn(OAc)_2_^a^	Intractable mixture	—
Mg(OBu)_2_	44 : 23 : 20	1 : 0.52 : 0.46

a With DBU.

b Impurities were present according to ^1^H NMR analysis.

c Free-base Pcs were isolated (M = H_2_).

d An impurity was present according to TLC analysis.

Our attempts to demetallate the CuPcs by the action of acids (H_2_SO_4_, CH_3_SO_3_H) or other methods (reduction with Li metal; treatment with EDTA) were unsuccessful. Thus, the DBU/Cu^II^ synthetic route was unsuitable for the preparation of the free-base Pcs, as the Cu^II^ template could not be expelled from the assembled macrocycle.

In search of a better template, we screened different metal salts in the phthalonitrile cyclotetramerization reaction in 1-pentanol with DBU as a base. The reaction in the absence of any metal salt as well as the reactions carried out with MgCl_2_ or Li_2_CO_3_ gave trace amounts of Pc compounds (Table 1). In the reactions performed in the presence of AgNO_3_, Cs_2_CO_3_, or Mn(OAc)_2_·4H_2_O, no detectable amount of Pc compounds was produced. LiBH_4_ and LiBr promoted the reaction, although the yields of the free-base Pcs were low. In the reaction with Zn(OAc)_2_, a mixture of unidentified species containing minor amounts of Pc compounds was formed. In the reactions with FeBr_2_ or FeCl_2_·4H_2_O, Pc formation was evident, although rapid and extensive decomposition occurred during attempted product isolation. With Co(OAc)_2_·4H_2_O, NiCl_2_, and PdCl_2_, impure products were produced in low yields.

A major improvement was achieved by utilizing magnesium(ii) butoxide as the reaction promoter. The sterically congested Pc regioisomers were obtained with significantly increased yields (44% *C*_4h_, 23% *C*_s_, and 20% *C*_2v_; 87% combined yield) in comparison with the conventional lithium alkoxide route (42% combined yield). H_2_Pc-Windmill, H_2_Pc-Dragon, and H_2_Pc-Frog free bases were isolated by treatment of the reaction mixture with trifluoroacetic acid^7,43–45^ to remove the Mg^2+^ template from the macrocyclic core (Scheme 1) and purified as previously described.^8^

Although the use of Mg^II^ as a metal template for phthalonitrile cyclotetramerization has been described in the literature, the source of metal, the reaction conditions, and the yields of the products vary considerably.^7,43–49^ In Table S1 we highlight the relevance of our findings (entry 1) in comparison with the reported Mg^II^-templated reactions (entries 2–18). The most efficient published method employed magnesium pentoxide in pentanol/octanol mixed solvent (entries 2–5). The use of I_2_-activated Mg in 1-butanol resulted in mostly moderate yields (entries 6–8, 10, 12, 13, 15, 16), and Mg^II^ salts in the presence of an organic base (entries 14, 17–18) gave the lowest yields.

Based on the literature data, the likely reaction mechanism for the Mg^II^ alkoxide-promoted Pc assembly involves alkoxide-initiated dimerization of the phthalonitrile molecules to produce half-Pcs of the kind reported previously (Scheme S1).^50–55^ Two types of such iminodiisoindoles may be envisaged to form in the case of an α-substituted phthalonitrile: one bearing the α-aryl substituents in the *exo*,*exo*-arrangement, while the other would have those substituents *exo*,*endo*-positioned. The third possibility with the *endo*,*endo*-arranged α-aryl groups would be too strained. For the Pc ring assembly, the coordination of the two half-Pcs around the templating metal ion can be either square-planar or tetrahedral. In a subsequent step, the adjacent half-Pcs undergo intramolecular nucleophilic addition to produce the alkoxy-substituted ring-modified Pcs,^56–61^ followed by the expulsion of the alkoxy substituents to form the final regioisomeric Pcs.

The assembly of the half-Pcs around the templating metal ion is the step which determines the regioselectivity of our Mg^II^ butoxide-promoted Pc synthesis (*C*_4h_ : *C*_s_ : *C*_2v_ = 1 : 0.52 : 0.46). When the two *exo*,*endo*-diisoindoles coordinate to the Mg^2+^ centre, the bulky α-substituents do not experience any geometric constraints in a tetrahedral or square-planar arrangement around the Mg^2+^ template during the assembly of the *C*_4h_ regioisomer, which explains its higher yield. On the contrary, formation of the *C*_s_ and *C*_2v_ regioisomers is favoured by the tetrahedral coordination geometry, since the square-planar arrangement of the two half-Pcs is impeded by the steric repulsion of the bulky substituents. The advantage of using Mg^2+^ over Li^+^ to produce the constrained *C*_s_ and *C*_2v_ regioisomers can be attributed to the more favourable arrangement of the anionic half-Pcs around the divalent Mg^2+^*versus* the monovalent Li^+^, which leads to a minimized steric repulsion of the neighbouring α-aryl-isoindole units. Our results are supported by the experimental reports on regioisomeric α-L-menthol-substituted Pcs (Table S1, entry 13), which were prepared *via* Mg^2+^- and Li^+^-templated phthalonitrile cyclotetramerization resulting in different regioisomeric distributions.^43,62^ A similar overall mechanism may be proposed for the phthalonitrile cyclotetramerization reaction in the presence of Cu^2+^ and DBU.

For the investigation of the PdPcs and their application in liposomal release, we required a synthetic strategy to produce regioisomerically pure PdPc-Windmill, PdPc-Dragon, and PdPc-Frog. As shown in Table 1, metal-templated phthalonitrile cyclotetramerization with PdCl_2_ was unsuccessful for the preparation of the desired PdPcs. Alternatively, the free-base H_2_Pcs can be metallated with the respective metal salt in DMF which is demonstrated in this work with copper(ii) and in our previous study with cobalt(ii).^8^ With PdCl_2_ and Pd(OAc)_2_ as a Pd^II^ source, however, the reductive nature of DMF resulted in the formation of Pd metal instead of Pc macrocycle metallation. By changing the solvent from DMF to diphenyl ether we were able to obtain PdPc-Windmill, PdPc-Dragon, and PdPc-Frog in good yields after column chromatography.

### Spectroscopic characterisation

**Fig. 1:**
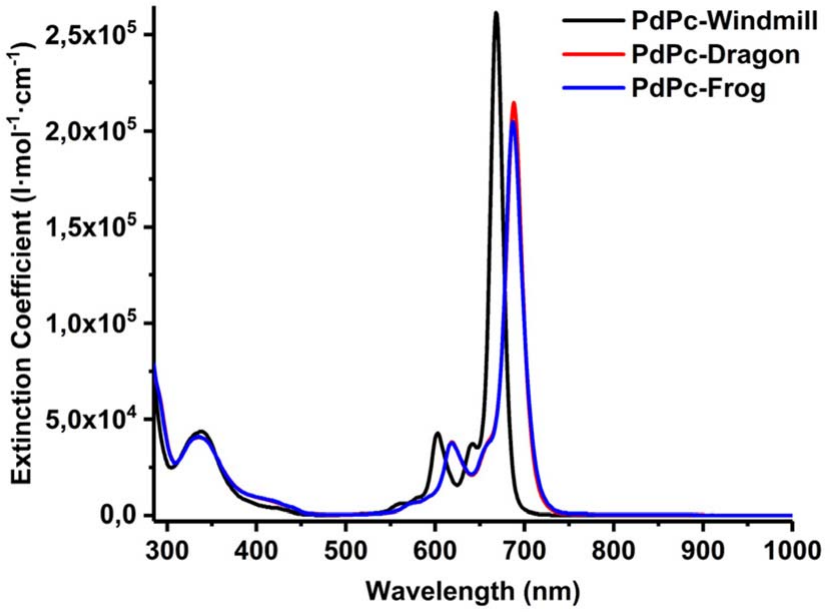
UV-vis absorption spectra of the individual regioisomeric PdPcs in toluene.

The UV-vis absorption spectra of the regioisomeric PdPcs (Fig. 1) display unsplit Q bands indicating successful metalation, and the high values of the extinction coefficients at the Q bands attest to the purity of the compounds. The Q band absorption maxima of the *dragon* and *frog* regioisomers are red-shifted by 20 nm relative to that of the *windmill* complex due to the saddle-type Pc ring distortion, as observed previously for the regioisomeric Co^II^Pcs.^8^

**Fig. 2:**
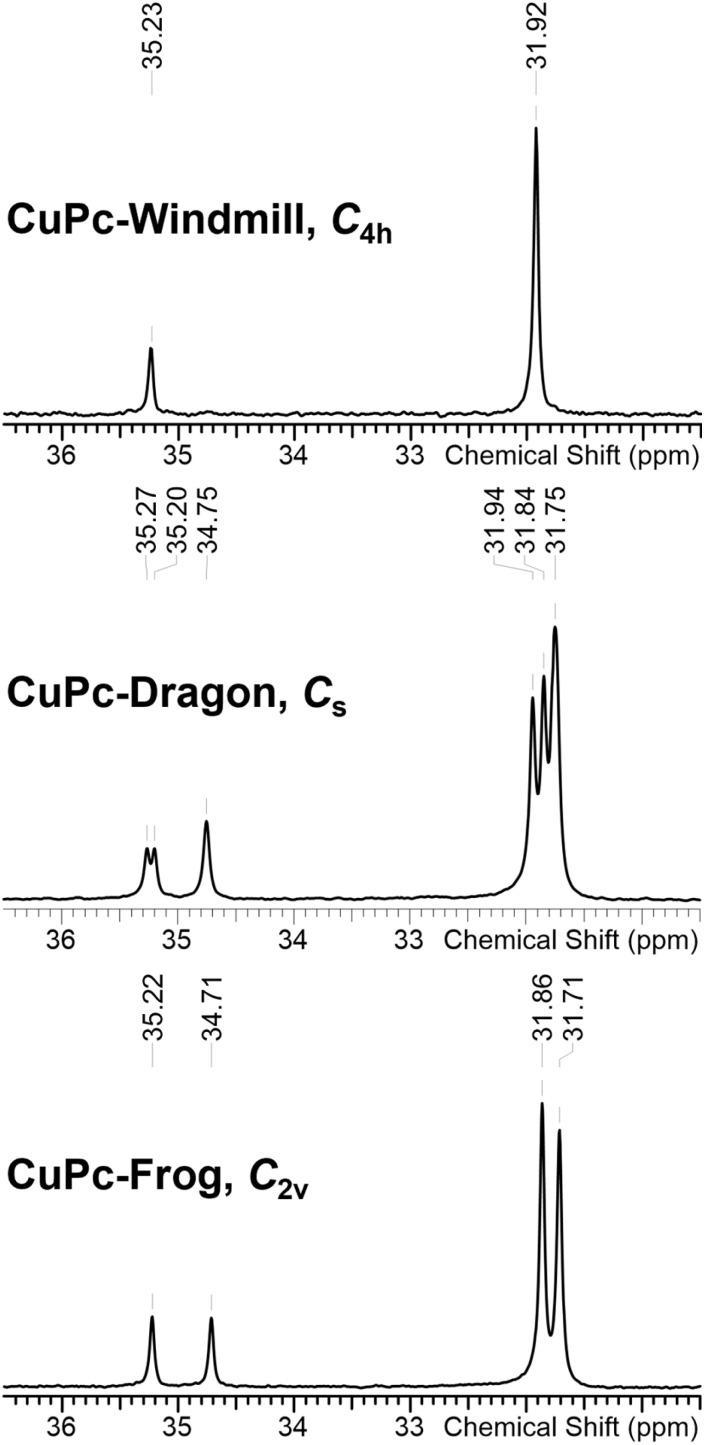
*tert*-Butyl groups' resonances in the ^13^C{^1^H} NMR spectra (126 MHz, CDCl_3_) of the regioisomeric CuPcs. The signals at *ca.* 32 ppm correspond to the methyl groups, and the signals at *ca.* 35 ppm belong to the quaternary carbons. The higher the number of signals, the lower the point group symmetry of the molecule.

The high-resolution mass spectra of the compounds under study demonstrate monoisotopic masses within 5 ppm of the theoretical values, and the observed isotopic patterns perfectly match those calculated (see SI Fig. S1–S7). For the CuPcs, there is an overlap of the M^+^ and [M + H]^+^ signals. The NMR spectra of the compounds obtained display sets of signals with chemical shifts, multiplicities, and integral intensities in perfect agreement with the proposed structures (see SI Fig. S10–S59). Signal assignment for the ^1^H and ^13^C NMR spectra was performed with the help of two-dimensional techniques (see SI Fig. S60–S81). Due to the paramagnetic nature of the metal centre in the CuPcs, the ^1^H NMR spectra are not readily amenable to interpretation (see SI Fig. S48, S50, and S52). The aromatic region of the ^13^C NMR spectra contains few broadened resonances, most of which could not be assigned. Yet the *tert*-butyl groups, owing to their sufficient spatial separation from the paramagnetic metal atom, give rise to sharp ^13^C signals. The number of *tert*-butyl resonances for each regioisomer is indicative of the molecular symmetry of the species, and this can be conveniently used for structural assignment (Fig. 2).

### Single-crystal X-ray diffraction studies

**Fig. 3:**
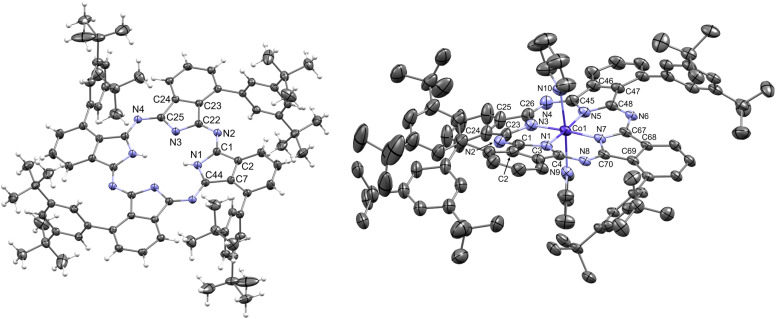
Molecular views of H_2_Pc-Windmill (Left; *R*_int_ = 4.04%) and [CoPc-Dragon(py)_2_] (Right; *R*_int_ = 9.10%). Displacement ellipsoids are drawn at the 50% (Left) and 30% (Right) probability levels, and solvent molecules and disorder are omitted for clarity. For [CoPc-Dragon(py)_2_], hydrogen atoms are omitted for clarity. Right: The four isoindole rings are labelled for data evaluation: Ring A – N7–C67–C68–C69–C70; Ring B – N1–C1–C2–C3–C4; Ring C – N3–C23–C24–C25–C26; Ring D – N5–C45–C46–C47–C48.

Crystal structures for two types of positional isomers were obtained: *windmill* (*C*_4h_, H_2_Pc-Windmill and NiPc-Windmill) and *dragon* (*C*_s_, CoPc-Dragon) (Tables 2 and S2). The molecular structures of these compounds are shown in Fig. 3, S82, and S83. The structures of the regioisomeric H_2_Pcs and Co^II^Pcs studied herein were previously determined by NMR spectroscopy and further corroborated by theoretical calculations.^8^ The crystallographic data now obtained confirm the correctness of the original structural assignments.

**Table 2:** Crystallographic data and refinement parameters for H_2_Pc-Windmill, NiPc-Windmill, and [CoPc-Dragon(py)_2_]

	H_2_Pc-Windmill	NiPc-Windmill	[CoPc-Dragon(py)_2_]
CCDC number	2540123	2540124	2540125
Chemical formula	C_88_H_98_N_8_·2(CHCl_3_)[+3.5(CHCl_3_)]	C_88_H_96_N_8_Ni·2(CHCl_3_)[+3.6(CHCl_3_)]	C_98_H_106_CoN_10_[+1.6CHCl_3_]
*M*_r_	1506.47	1563.17	1482.85
Crystal system, space group	Triclinic, *P*1̄	Triclinic, *P*1̄	Monoclinic, *P*2_1_/*c*
Temperature (K)	121	122	120
*a*, *b*, *c* (Å)	9.1346 (1), 16.5089 (3), 17.1896 (3)	9.1452 (2), 16.4727 (4), 17.1941 (4)	30.8527 (10), 16.1188 (3), 18.8367 (3)
*α*, *β*, *γ* (°)	74.833 (2), 85.774 (1), 82.337 (1)	74.601 (2), 85.688 (2), 82.464 (2)	90, 93.802 (2), 90
*V* (Å^3^)	2477.55 (7)	2473.46 (10)	9347.0 (4)
*Z*	1	1	4
Radiation type	Cu Kα	Cu Kα	Cu Kα
Measured reflections	49447	48170	77189
Independent reflections	10561	9982	16486
Observed reflections [*I* > 2*s*(*I*)]	9531	8979	9863
*R*[*F*^2^ > 2*s*(*F*^2^)]	0.057	0.086	0.069
w*R*(*F*^2^)	0.163	0.256	0.211
*S*	1.05	1.06	1.03

The central motif of a phthalocyanine is described by the bond lengths and angles of the five-membered ring of the isoindole unit (N_i_, C_α_ and C_β_) as well as the aza bridge (N_br_). These data are presented in Table 3. There is no significant difference between other substituted (α and β) and unsubstituted phthalocyanines, respectively, because of the rigidity of the main aromatic ring system.^63–67^ The insertion of nickel into the central cavity of the ligand results in small changes, indicated by the elongation of N_i_–C_α_ and shortening of the aza bridge (N_br_–C_α_). Similar adjustments of the ring system were reported for an α-substituted *N*,*N*-dibutylamino phthalocyanine nickel complex.^66^

**Table 3:** Selected average bond lengths (Å) and angles (°) of H_2_Pc-Windmill, NiPc-Windmill and [CoPc-Dragon(py)_2_]. For [CoPc-Dragon(py)_2_], the letter indicates the respective isoindole ring

	H_2_Pc-Windmill	NiPc-Windmill	[CoPc-Dragon(py)_2_]
N_i_–C_α_	1.369	1.380	A: 1.373; B: 1.372; C: 1.379; D: 1.378
C_α_–C_β_	1.459	1.445	A: 1.450; B: 1.463; C: 1.452; D: 1.462
N_br_–C_α_	1.327	1.319	1.323
M–N_i_	—	1.905	A: 1.909(3)^a^; B: 1.916(3)^a^; C: 1.903(3)^a^; D: 1.916(3)^a^
N_i_–C_α_–N_br_	128.1	127.8	A: 126.2; B: 126.3; C: 126.8; D: 127.2
C_α_–N_i_–C_α_	109.4	106.6	A: 106.6(3)^a^; B: 107.7(3)^a^; C: 107.6(3)^a^; D: 107.7(3)^a^
C_α_–N_br_–C_α_	123.3	121.0	121.8

a No mean value.

The crystal lattice in H_2_Pc-Windmill and NiPc-Windmill is arranged in a “slipped-stacked” packing which is indicated by an off-set to the adjacent molecules along the stacking axis (Fig. S84).^65^ Interestingly, the aromatic ring systems of stacked molecules are separated from each other by the spacer-like bulky di-(*tert*-butyl)phenyl groups similar to comparable phthalocyanines with the *C*_4h_ symmetry.^6,68^ As a result, intermolecular π–π-stacking with subsequent aggregation is prevented. This is depicted by the center-to-center distance (H_2_Pc-Windmill: 9.14 Å; NiPc-Windmill: 9.16 Å) and the distance between two stacked phthalocyanine planes (H_2_Pc-Windmill: 7.39 Å; NiPc-Windmill: 7.44 Å). In addition, the spatial separation of the phthalocyanines results in a voluminous network of solvent-filled channels in between the molecules as well as alongside the stacked columns (Fig. S84).

Crystals of a square-planar CoPc-Dragon without inclusion of a solvent could not be obtained under various crystallization conditions. [CoPc-Dragon(py)_2_] could be isolated as crystals suitable for SC-XRD analysis when CoPc-Dragon was crystallized from a solution containing pyridine. Having performed an extensive literature search, we are certain that the crystal structure of [CoPc-Dragon(py)_2_] is the second example of an α-substituted phthalocyanine with the *C*_s_ symmetry (in terms of the substitution pattern of the Pc ring), after a menthol-substituted MgPc complex.^43^ In the crystal structure of [CoPc-Dragon(py)_2_], the major component (76%) represents the known motif^69,70^ with two pyridines serving as classic Lewis bases which coordinate the cobalt atom at the apical positions *via* the nitrogen atoms (Fig. 3, Right). Due to this additional axial ligation, the geometry changes from square-planar to octahedral with an unusual distortion of the vertical axis (N_py_–Co–N_py_ = 175.2°). The minor component (24%) indicates that one pyridine is flipped, resulting in a coordination based on π-interactions. The axial coordination of the pyridine leads to a different packing which is directed by a columnar “zigzag” orientation of the phthalocyanine rings along the crystallographic *c* axis. In between these columns, the space is filled with residual solvent molecules (Fig. S85).

So as to the main motif, SC-XRD studies confirm that the phthalocyanine ring deforms into a saddle-like conformation as predicted by DFT calculations.^8^ This effect may be attributed to the stacked arrangement of two adjacent di-(*tert*-butyl)phenyl moieties (Ring B and C). The two remaining α-substituents (ring A and D) show no perpendicular orientation relative to the isoindole ring as in H_2_Pc-Windmill. Instead, these are tilted by 51.2° (ring A) and 56.3° (ring D). Although, the phthalocyanine ring of the *dragon* is distorted, the structural parameters of the central motif indicate no significant changes compared to the *windmill* (Table 3).

### Singlet oxygen quantum yields

The regioisomeric PdPcs under study are expected to have high yields of intersystem crossing due to the heavy atom effect of Pd^II^, which in turn should lead to efficient singlet oxygen generation. Anticipating future application of the PdPcs regioisomers in ROS-triggered cargo release, we determined singlet oxygen quantum yields using the distinctive emission peak of ^1^O_2_ at 1270 nm. Indeed, the singlet oxygen quantum yields in toluene upon 640 nm excitation were found to be 81%, 90%, and 94% for PdPc-Windmill, PdPc-Frog, and PdPc-Dragon, respectively. Such high values result from suppressed aggregation due to the bulky di-(*tert*-butyl)phenyl groups, high solubility of the compounds in toluene, and the heavy-atom effect of Pd^II^. Altogether, this makes all three compounds promising candidates for PDT and light-induced oxidation of liposomes in cargo release applications. Spectroscopic properties of the studied compounds are summarized in Table 4.

**Table 4:** Spectroscopic properties of the PdPcs studied

Compound	Soret band (*λ*, *ε*)	Q band (*λ*, *ε*)	*Φ*_Δ_
PdPc-Windmill	339 nm/43 500 M^−1^ cm^−1^	668 nm/261 600 M^−1^ cm^−1^	81%
PdPc-Dragon	335 nm/40 500 M^−1^ cm^−1^	688 nm/214 400 M^−1^ cm^−1^	94%
PdPc-Frog	336 nm/40 900 M^−1^ cm^−1^	687 nm/204 800 M^−1^ cm^−1^	90%

### Characteristics of liposomes

The preparation of liposomes containing PdPcs was challenging due to the high molecular mass of phthalocyanines and their slow encapsulation process into liposomal bilayers. A two-times longer hydration time was needed compared to the liposomes containing verteporfin (VERT), and a sequential extrusion through 800 and 400 or 200 nm membranes was necessary before the successful extrusion through a 100 nm membrane. This was caused by precipitation of the PdPc molecules during the hydration process, and their clogging of the extrusion membranes. Despite the technical difficulties, the final uniformity of PdPc liposomes was high (Table 5). The particle size and polydispersity index (PDI) of the resultant liposomes are suitable for a clinical treatment, being in line with generally accepted values.^71^ The zeta potential of the liposomes was positive as a cationic DOTAP was used as one of the main components.^72^ Furthermore, the liposomes showed favorable stability (see SI Fig. S86). During 30 days of storage at 4 °C or 7 days of storage at 37 °C, the particle size and PDI remained stable, and a passive leakage of calcein from the liposomes was not detected. The absorbance of PdPc-Dragon and PdPc-Frog in liposomes fluctuated a bit more during the storing period compared to VERT which had superior stability at both temperatures. However, this fluctuation was not statistically significant. Incubation of the PdPcs liposomes in serum for 48 h at r.t. did not reveal any changes in the absorption either (see SI Fig. S87).

**Table 5:** Properties of PdPc and VERT liposomes. PDI = polydispersity index, ZP = zeta potential, EE% = encapsulation efficiency, <LOD = below the detection limit

Liposome formulation	Size (nm)	PDI	ZP (mV)	EE% (%)
PdPc-Windmill	125.2 ± 0.3	0.117 ± 0.008	25.2	<LOD
PdPc-Dragon	111.8 ± 7.8	0.113 ± 0.035	30.3 ± 0.2	58.3 ± 2.9
PdPc-Frog	107.7 ± 3.5	0.116 ± 0.004	27.7 ± 4.6	62.9 ± 4.0
VERT	122.2 ± 3.7	0.120 ± 0.010	27.5 ± 2.4	89.2 ± 1.9

The encapsulation efficiencies of the PdPcs in liposomes were lower than for VERT (Table 5). As mentioned above, this could already be seen during the preparation process, in which extrusion membranes with a series of pore sizes were required due to PdPc precipitation outside the liposomes. Especially, PdPc-Windmill got visibly stuck to the extrusion membranes, turning the color of the liposome solution from blue to white during extrusion. Thus, it was not surprising that the amount of encapsulated PdPc-Windmill was below 10.1%, corresponding to the detection limit of the spectrophotometric measurements. Instead, PdPc-Dragon and PdPc-Frog were encapsulated in liposomes in sufficient amounts (58.3 ± 2.9% and 62.9 ± 4.0%, respectively). For VERT, the encapsulation efficiency was higher than described earlier in neutral liposomes.^73^ This may be explained by the positive charged DOTAP, used in our liposomes, interacting with VERT which has a negative charge at pH 7.4.^74^

### Photo-oxidation effect of photosensitizers (PS) on liposome lipid bilayer

The calcein release study was conducted to assess the intensity of the photo-oxidation effect of PdPcs on lipid bilayers, providing an estimation of the potential of synthesized PSs in photodynamic treatment and light-triggered drug release. Calcein is an emissive hydrophilic molecule whose fluorescence self-quenches at high concentrations.^75^ Thus, when a lipid film is hydrated with a calcein solution, calcein molecules are encapsulated in liposomes, and their fluorescence decreases. When the structure of liposomes is destabilized by photo-oxidation, calcein diffuses out, and the fluorescence increases.

**Fig. 4:**
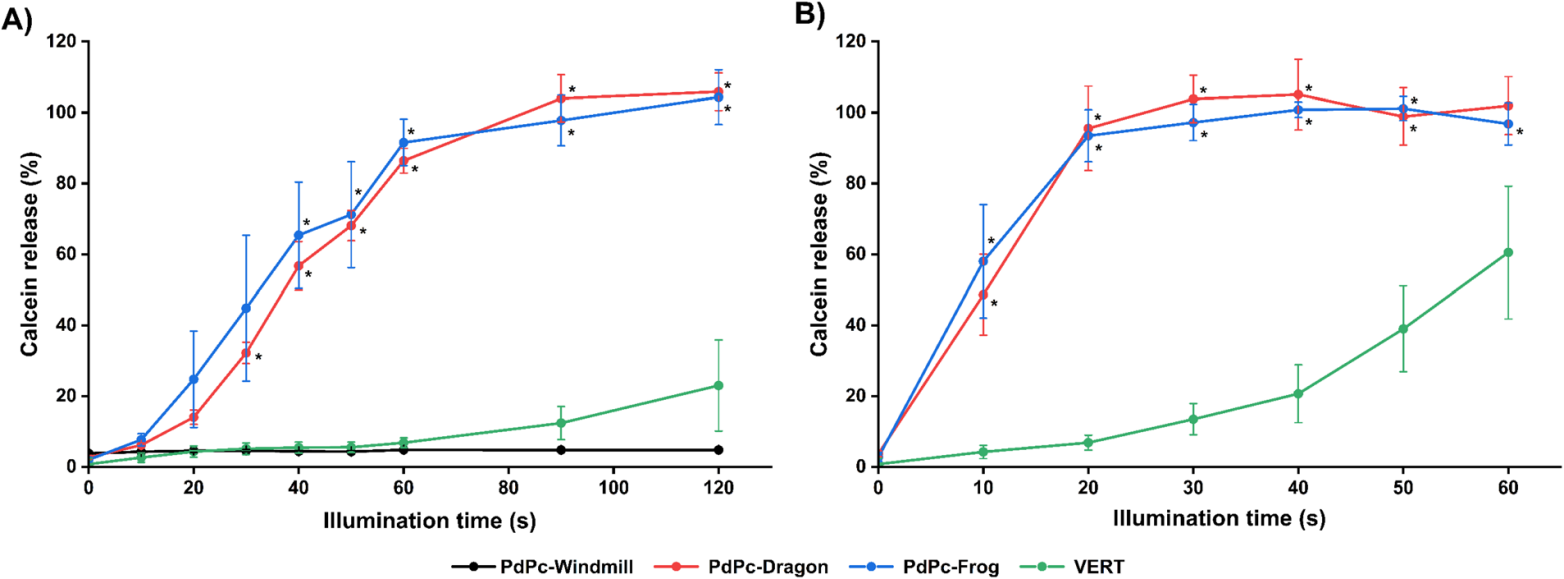
The relative calcein release from PdPc and VERT liposomes at power densities of 25 mW cm^−2^ (A) and 75 mW cm^−2^ (B) with a wavelength of 690 nm. For PdPc-Windmill, a power density of 28 mW cm^−2^ and a wavelength of 660 nm were used. The statistically significant differences (*p* < 0.05) between PdPcs and VERT are marked with *.

As PdPc-Windmill showed low encapsulation efficiency in liposomes, it was not surprising that the calcein release from PdPc-Windmill liposomes was extremely low (Fig. 4A). However, it provided a solid proof that the liposomes had a high stability under illumination without incorporated photosensitizer as the relative calcein release from PdPc-Windmill liposomes was only 5.8 ± 0.3% even after 5 minutes of light exposure. Therefore, we conclude that the potential laser-induced heating of samples did not affect markedly the integrity of the liposomal lipid bilayer itself, and the calcein release was mainly caused by the photo-oxidation. From the controls which were not exposed to the laser beam, the relative calcein release was 3.8 ± 0.3%, 2.7 ± 0.5%, 2.0 ± 0.5%, and 0.8 ± 1.7% for PdPc-Windmill, PdPc-Dragon, PdPc-Frog, and VERT liposomes, relatively.

Contrasting the problems related to PdPc-Windmill liposomes, the light-induced calcein release from PdPc-Dragon and PdPc-Frog liposomes was very efficient. Both formulations achieved complete cargo release in 90 seconds at a power density of 25 mW cm^−2^, and in 40 seconds at a power density of 75 mW cm^−2^ (Fig. 4). At the same time points, the relative calcein releases from VERT liposomes were only 12.4 ± 4.7% and 20.7 ± 8.2%, although the encapsulation efficiency of VERT in the liposomes was higher than the encapsulation efficiencies of PdPc-Dragon and PdPc-Frog. In the presence of an oxygen scavenger (Na_2_SO_3_), the calcein releases for all three formulations remained below 10% even at higher power densities (see SI Fig. S88), indicating that the calcein release is ROS-dependent.^39^ Therefore, the differences in cargo release can be explained by notably higher photo-oxidative effect of PdPc-Dragon and PdPc-Frog compared to VERT. Given that the quantum yields of singlet oxygen for the three photosensitizers were comparable (94%, 90% and 76–79%,^76^ respectively), the stronger photo-oxidative effect of Pcs may be attributed to their higher lipophilicity in comparison to VERT and thus deeper intercalation of Pcs inside the lipid bilayer. This ensures closer proximity of the generated singlet oxygen to its targets, *i.e.* unsaturated lipids, and results in a longer residence time of singlet oxygen within the lipid bilayer.^73^ Both factors contribute to a much more efficient lipid oxidation and faster cargo release from the phthalocyanine-sensitized liposomes.

## Conclusions

Individually resolved sterically congested *dragon* (*C*_s_) and *frog* (*C*_2v_) regioisomers of α-tetrakis[3,5-di-(*tert*-butyl)phenyl] phthalocyanine were prepared in very high yields by a magnesium(ii)-templated Pc ring assembly reaction with alkoxide as a base. The Mg^II^ template allowed easy and mild demetallation with trifluoroacetic acid and subsequent preparation of the respective Pd^II^ complexes, which are otherwise inaccessible. The PdPcs were found to be efficient ^1^O_2_ generators in solution with quantum yields exceeding 80% for all the three regioisomers.

The three isomers have dramatically different solubilities and crystallization abilities. Thus far, their structures were elucidated only by NMR spectroscopy. We obtained single crystals for the free-base H_2_Pc-Windmill, its Ni^II^ complex, and CoPc-Dragon. X-ray diffraction studies proved the correctness of the structures and provided bond lengths and angles, which were in good agreement with those predicted previously by molecular modelling.

A fundamental difference between the isomers was demonstrated by their vastly different encapsulation efficiencies into liposomes. The distorted *dragon C*_s_ and *frog C*_2v_Pcs were easily incorporated into the liposomal shells, whereas the symmetrical *C*_4h_PdPc-Windmill did not enter the lipid bilayer. As a consequence, PdPc-Dragon and PdPc-Frog functioned as efficient photosensitizers in light-triggered cargo release from liposomal carriers. The calcein release studies showed a markedly higher effect of PdPc-Dragon and PdPc-Frog on disruption of liposomal lipid bilayers in comparison with Verteporfin – a benchmark porphyrinic compound which is in clinical use for photodynamic therapy. Thus, our findings are highly encouraging and provide a compelling rationale for the preparation of sterically constrained Pc regioisomers. Our research will continue in the pursuit of PdPc-sensitized liposomes as new agents for photodynamic therapy and drug release applications.

## Materials and methods

### General notes

Analytical and preparative TLC was performed on precoated normal phase silica gel 60 F_254_ aluminium-backed plates (Merck). Column chromatography was performed using silica gel 60 (40–63 µm particle size, Merck). In the synthetic procedures for compounds 1–4, the indicated elevated temperatures refer to the oil bath temperatures monitored with a partial immersion thermometer. For all the other reactions, the indicated elevated temperatures refer to the aluminum heating block temperatures monitored with a partial immersion thermometer. NMR spectra were recorded on a JEOL JNM-ECZ500R 500 MHz instrument. Chemical shifts are reported in parts per million (ppm) and referenced to TMS (0.00 ppm) or residual CHCl_3_ (7.26 ppm), or residual DMSO-d_5_ (2.50 ppm) for ^1^H NMR spectra and to CDCl_3_ (77.16 ppm) or DMSO-d_6_ (39.52 ppm) for ^13^C NMR spectra. High-resolution ESI-TOF mass spectra were measured on a JEOL ESI-TOF JMS-T100LP instrument in the positive mode using reserpine as a mass correction reference. UV-vis absorption spectra were recorded with a Shimadzu UV-1800 spectrophotometer in 1 cm quartz cuvettes. Solvents and chemicals were obtained commercially and, unless otherwise stated, were used as received. Dimethylformamide was distilled over P_2_O_5_ under reduced pressure and stored over activated 3 Å molecular sieves under argon. 1,8-Diazabicyclo[5.4.0]undec-7-ene (DBU) was distilled under reduced pressure.

For the preparation and characterization of liposomes, 1,2-dioleoyl-3-trimethylammonium-propane (chloride salt) (DOTAP; 890890C, Avanti Research, USA), 1,2-distearoyl-*sn*-glycero-3-phosphoethanolamine-*N*-[methoxy(polyethylene glycol)-2000] (ammonium salt) (DSPE-PEG2K; 880120P, Avanti Research, USA), cholesterol (CHOL; C8667, Sigma-Aldrich, USA), verteporfin (VERT; SML0534, Sigma-Aldrich, USA), HEPES (H4034, Sigma-Aldrich, USA), calcein (C0875, Sigma-Aldrich, USA), Sephadex® G-50 medium (GE17-0043-01, Cytiva, USA), pyridine (360570, Sigma-Aldrich, USA), and sodium sulfite (Na_2_SO_3_; 1.06657.0500, Merck, Germany) were purchased from Merck (Germany). Sodium chloride (NaCl; 207790010, Thermo Scientific, USA) and Gibco™ fetal bovine serum (FBS; A5209401, Thermo Scientific, USA) were purchased from Thermo Fisher Scientific (USA).

### Phthalonitrile synthesis

#### 2-(3,5-Di-(*tert*-butyl)phenyl)-4,4,5,5-tetramethyl-[1,3,2]dioxaborolane (1)

A mixture of 1-bromo-3,5-di-(*tert*-butyl)benzene (2.000 g, 7.429 mmol), bis(pinacolato)diboron (2.075 g, 8.173 mmol), potassium acetate (2.209 g, 22.509 mmol), and PdCl_2_(dppf)·CH_2_Cl_2_ (183 mg, 0.224 mmol) in DMSO (50 ml) was sparged with argon at r.t. for 15 min and stirred magnetically at 80 °C for 20 h under argon. The mixture was cooled down to r.t., diluted with water (150 ml) and extracted with hexane/EtOAc 1 : 1 (1 × 150 ml, 1 × 100 ml, 1 × 50 ml). The combined organic extracts were washed with water (2 × 100 ml), dried over Na_2_SO_4_, filtered and evaporated to dryness under reduced pressure. The residue was chromatographed on silica gel (hexane/EtOAc 15 : 1). Evaporation of appropriately pooled fractions and subsequent drying under vacuum delivered the title compound as a white solid (2.122 g, 93%). The ^1^H and ^13^C{^1^H} NMR spectra match those previously reported.^41^

#### 3-Hydroxyphthalonitrile (2)

A mixture of 3-nitrophthalonitrile (3.00 g, 17.33 mmol) and potassium carbonate (2.72 g, 19.68 mmol) in DMSO (20 ml) was sparged with argon at r.t. for 15 min and refluxed with magnetic stirring for 20 min under argon. The mixture was cooled down to r.t. and cold deionized water (100 ml) was added. The pH was adjusted to *ca.* 2 with conc. aq. HCl (about 4 ml) and the mixture was stirred magnetically at r.t. for 30 min. The precipitate was filtered off, washed with cold deionized water (4 × 3 ml), pressed dry on the filter and dried in air for 15 min. The filter cake was dissolved in portions of acetone (60 ml in total), and the resulting mixture was filtered, leaving behind a dark tarry residue. The filtrate was dried over Na_2_SO_4_, filtered, evaporated to dryness under reduced pressure, and the residue was dried under vacuum. Brown powder; 1.86 g, 74%. The ^1^H and ^13^C{^1^H} NMR spectra match those previously reported.^41^

#### 2,3-Dicyanophenyl trifluoromethanesulfonate (3)

To a magnetically stirred solution of 3-hydroxyphthalonitrile 2 (3.063 g, 21.26 mmol) in pyridine (25 ml) at 0 °C was added trifluoromethanesulfonic anhydride (4.50 ml, 7.50 g, 26.59 mmol) portionwise *via* a syringe over 20 min under argon. The mixture was stirred at 0 °C for an additional 30 min and then at r.t. for 12 h under argon. The mixture was partitioned between water (50 ml) and CHCl_3_ (50 ml), and the organic layer was separated. The aqueous layer was extracted with CHCl_3_ (2 × 20 ml). The combined organic phases were dried over Na_2_SO_4_, filtered and evaporated to dryness under reduced pressure. The residue was chromatographed on silica gel (CHCl_3_). Evaporation of appropriately pooled fractions and subsequent drying under vacuum afforded the title compound as an off-white solid (4.182 g, 71%). The ^1^H, ^13^C{^1^H}, and ^19^F{^1^H} NMR spectra match those previously reported.^41^

#### 3,5-Di-(*tert*-butyl)phenyl-2,3-dicyanobenzene (4)

A mixture of 2-(3,5-di-(*tert*-butyl)phenyl)-4,4,5,5-tetramethyl-[1,3,2]dioxaborolane 1 (1.000 g, 3.167 mmol), 2,3-dicyanophenyl trifluoromethanesulfonate 3 (927 mg, 3.357 mmol), K_3_PO_4_ (2.017 g, 9.501 mmol) and PdCl_2_(dppf)·CH_2_Cl_2_ (129 mg, 0.158 mmol) in toluene/water 1 : 1 (40 ml) was sparged with argon at r.t. for 10 min and stirred magnetically at 90 °C for 20 h under argon. The mixture was cooled down to r.t. and the organic layer was separated. The aqueous layer was extracted with toluene (2 × 10 ml), and the combined organic phases were dried over Na_2_SO_4_, filtered and evaporated to dryness under reduced pressure. The residue was dissolved in CH_2_Cl_2_ (10 ml), the solution was filtered through a short pad of silica gel, and the silica gel was flushed with CH_2_Cl_2_ (100 ml). The solvent was removed under reduced pressure, and the residue was recrystallized from boiling heptane (10 ml). The precipitate was filtered off, washed with hexane (2 × 1 ml) and dried under vacuum. White powder; 798 mg, 80%. The ^1^H and ^13^C{^1^H} NMR spectra match those previously reported.^41^

### Phthalocyanine synthesis

#### Regioisomeric free-base Pcs (H_2_Pc-Windmill*C*_4h_, H_2_Pc-Dragon*C*_s_, H_2_Pc-Frog*C*_2v_)

To iodine-activated magnesium turnings (48 mg, 1.975 mmol) was added 1-butanol (10 ml) and the mixture was stirred magnetically in a 40 ml screw-cap glass vial at 125 °C for 2.5 h under a static argon atmosphere with an exhaust gas outlet connected to an oil bubbler to vent the evolved H_2_. The resulting mixture was cooled down to r.t. and 3,5-di-(*tert*-butyl)phenyl-2,3-dicyanobenzene (200 mg, 0.632 mmol) was added. The mixture was sparged with argon for 30 min and stirred magnetically at 130 °C for 17 h under argon. The mixture was cooled down to r.t. and trifluoroacetic acid (1.5 ml, 2.21 g, 19.4 mmol) and CHCl_3_ (5 ml) were added. The mixture was stirred magnetically at 65 °C for 2 h under argon. The mixture was cooled down to r.t., washed with sat. aq. NaHCO_3_ (30 ml), and the organic layer was separated. The aqueous layer was extracted with CHCl_3_ (2 × 10 ml). The combined organic phases were dried over Na_2_SO_4_, filtered and evaporated to dryness under reduced pressure. The residue was chromatographed on silica gel (hexane/CH_2_Cl_2_ 2 : 1). The colored band was collected and evaporated to dryness under reduced pressure to give a mixture of the three regioisomeric H_2_Pcs. The residue was suspended in hexane (8 ml), the suspension was centrifuged, the supernatant was pipetted off, and the operation was repeated twice more using 8 ml of hexane each time. The precipitate was dried under vacuum to afford H_2_Pc-Windmill as a turquoise powder (89 mg, 44%). The combined hexane supernatants were evaporated to dryness under reduced pressure, and the residue was chromatographed on silica gel (hexane/CH_2_Cl_2_ 8 : 1 to 2 : 1). Evaporation of appropriately pooled fractions and subsequent drying under vacuum gave H_2_Pc-Dragon (46 mg, 23%) and H_2_Pc-Frog (41 mg, 20%) as dark green powders. The ^1^H and ^13^C{^1^H} NMR spectra for the materials obtained match those previously reported.^8^ The compounds can be conveniently recrystallized from the following solvent mixtures: H_2_Pc-Windmill, CHCl_3_/EtOH; H_2_Pc-Dragon, CHCl_3_/MeCN; H_2_Pc-Frog, CHCl_3_/EtOH.

#### Regioisomeric PdPcs (PdPc-Windmill, PdPc-Dragon, PdPc-Frog)

A mixture of the respective H_2_Pc (18 mg, 0.0142 mmol) and palladium(ii) acetate (15 mg, 0.0668 mmol) in diphenyl ether (3 ml) was sparged with argon at r.t. for 15 min and stirred magnetically at 260 °C for 2 h under argon. Isolation of PdPc-Windmill. Methanol (8 ml) was added to the reaction mixture at r.t., the resulting suspension was centrifuged, the precipitate was washed with methanol (2 × 4 ml) and dried under vacuum. The residue was chromatographed on silica gel (hexane/CH_2_Cl_2_ 3 : 1), and the colored band was collected. The solvent was removed under reduced pressure, the residue was washed with methanol (2 × 2 ml), acetonitrile (2 × 1 ml) and hexane (2 × 1 ml) and dried under vacuum. The residue was recrystallized from CHCl_3_/MeOH by slow evaporation at r.t. in darkness. The precipitate was collected by filtration, washed with methanol (2 × 2 ml) and dried under vacuum. Navy blue powder; 12 mg, 62%. ^1^H NMR (500 MHz, CDCl_3_): *δ* = 8.36 (dd, *J* = 7.3, 1.2 Hz, 4H), 7.98 (dd, *J* = 7.3, 1.2 Hz, 4H), 7.97 (t, *J* = 1.9 Hz, 4H), 7.94 (t, *J* = 7.3 Hz, 4H), 7.87 (d, *J* = 1.9 Hz, 8H), 1.51 (s, 72H) ppm; ^13^C{^1^H} NMR (126 MHz, CDCl_3_): *δ* = 150.7, 143.3, 142.2, 141.0, 140.3, 138.3, 133.7, 131.9, 128.7, 124.2, 122.5, 122.0, 35.4, 32.0 ppm; HRMS (ESI): *m*/*z* calcd for C_88_H_97_N_8_Pd^+^: 1371.68907 [M + H]^+^, found: 1371.68766; UV-vis (toluene, nm (l mol^−1^ cm^−1^)): *λ*_max_ (*ε*) 339 (43 500), 668 (261 600). Isolation of PdPc-Dragon and PdPc-Frog. The diphenyl ether was distilled off under reduced pressure, and the residue was chromatographed on silica gel (hexane/CH_2_Cl_2_ 8 : 1 to 3 : 1). The colored band was collected, and the solvent was removed under reduced pressure. The residue was washed with methanol (2 × 2 ml) and recrystallized from CHCl_3_/MeOH by slow evaporation at r.t. in darkness. The precipitate was collected by filtration, washed with methanol (2 × 2 ml) and dried under vacuum to afford PdPc-Dragon (11 mg, 56%) and PdPc-Frog (15 mg, 77%) as dark green powders. Characterization data for PdPc-Dragon. ^1^H NMR (500 MHz, CDCl_3_): *δ* = 9.51 (dd, *J* = 7.5, 1.0 Hz, 1H), 9.48 (dd, *J* = 7.5, 0.9 Hz, 1H), 8.36 (dd, *J* = 7.2, 1.3 Hz, 1H), 8.22 (t, *J* = 7.4 Hz, 1H), 8.19 (dd, *J* = 7.4, 1.0 Hz, 1H), 8.12 (dd, *J* = 7.3, 1.0 Hz, 1H), 8.11 (t, *J* = 7.3 Hz, 1H), 8.03 (dd, *J* = 7.5, 0.9 Hz, 1H), 7.99 (t, *J* = 1.8 Hz, 1H), 7.98–7.94 (m, 4H), 7.93 (t, *J* = 1.8 Hz, 1H), 7.90 (dd, *J* = 7.6, 0.9 Hz, 1H), 7.85 (d, *J* = 1.9 Hz, 2H), 7.81 (d, *J* = 1.7 Hz, 2H), 7.79 (d, *J* = 1.7 Hz, 2H), 7.79 (t, *J* = 7.5 Hz, 1H), 7.15 (t, *J* = 1.6 Hz, 2H), 1.53 (s, 18H), 1.48 (s, 18H), 1.22 (s, 18H), 1.21 (s, 18H) ppm; ^13^C{^1^H} NMR (126 MHz, CDCl_3_): *δ* = 150.8, 149.1, 143.9, 143.7, 143.4, 143.11, 143.09, 143.05, 142.9, 142.6, 141.5, 141.1, 140.5, 140.2, 140.04, 140.03, 139.72, 139.67, 138.30, 138.29, 137.69, 137.67, 134.1, 133.9, 133.5, 132.2, 132.0, 131.9, 131.7, 129.6, 129.3, 129.1, 128.9, 124.3, 123.98, 123.97, 123.8, 122.5, 122.2, 122.1, 121.8, 121.7, 121.20, 121.18, 121.0, 35.43, 35.37, 34.94, 34.93, 32.0, 31.9, 31.69, 31.65 ppm; HRMS (ESI): *m*/*z* calcd for C_88_H_97_N_8_Pd^+^: 1371.68907 [M + H]^+^, found: 1371.68416; UV-vis (toluene, nm (l mol^−1^ cm^−1^)): *λ*_max_ (*ε*) 335 (40 500), 688 (214 400). Characterization data for PdPc-Frog. ^1^H NMR (500 MHz, CDCl_3_): *δ* = 9.53 (dd, *J* = 7.5, 0.9 Hz, 2H), 8.23 (t, *J* = 7.4 Hz, 2H), 8.17 (dd, *J* = 7.3, 0.9 Hz, 2H), 8.11 (dd, *J* = 7.3, 0.9 Hz, 2H), 7.96 (t, *J* = 1.9 Hz, 2H), 7.91 (d, *J* = 1.9 Hz, 4H), 7.89 (dd, *J* = 7.5, 0.9 Hz, 2H), 7.79 (t, *J* = 7.5 Hz, 2H), 7.75 (d, *J* = 1.7 Hz, 4H), 7.12 (t, *J* = 1.7 Hz, 2H), 1.50 (s, 36H), 1.19 (s, 36H) ppm; ^13^C{^1^H} NMR (126 MHz, CDCl_3_): *δ* = 150.8, 149.1, 143.4, 143.3, 143.1, 141.5, 140.1, 140.0, 139.6, 138.4, 137.6, 133.9, 133.8, 132.1, 131.7, 129.4, 129.1, 124.3, 123.8, 122.1, 121.8, 121.75, 121.1, 35.4, 34.9, 31.9, 31.6 ppm; HRMS (ESI): *m*/*z* calcd for C_88_H_96_N_8_PdNa^+^: 1393.67102 [M + Na]^+^, found: 1393.66445; UV-vis (toluene, nm (l mol^−1^ cm^−1^)): *λ*_max_ (*ε*) 336 (40 900), 687 (204 800).

#### CuPc-Windmill

A mixture of H_2_Pc-Windmill (50 mg, 0.0395 mmol) and copper(ii) chloride dihydrate (69 mg, 0.405 mmol) in dimethylformamide (5 ml) was stirred magnetically at 110 °C for 20 h under argon. The solvent was removed under reduced pressure, and the residue was chromatographed on silica gel (hexane/CH_2_Cl_2_ 5 : 1 to 4 : 1). The colored band was collected, the solvent was removed under reduced pressure, and the residue was recrystallized from CH_2_Cl_2_/EtOH by slow evaporation at r.t. in darkness. The precipitate was collected by filtration, washed with ethanol (3 × 2 ml) and dried under vacuum to afford CuPc-Windmill as a dark blue powder (48 mg, 91%). The analytical data (TLC mobility, NMR, UV-vis) for the material obtained match that for the compound produced in the phthalonitrile cyclotetramerization reaction performed with CuBr_2_ (*vide infra*).

### Phthalonitrile tetramerization reactions in the presence of a metal salt and DBU in 1-pentanol

#### Reaction with CuBr_2_

A mixture of 3,5-di-(*tert*-butyl)phenyl-2,3-dicyanobenzene (1 g, 3.163 mmol), copper(ii) bromide (220 mg, 0.985 mmol), and DBU (482 mg, 3.167 mmol) in 1-pentanol (40 ml) was sparged with argon at r.t. for 25 min and refluxed with magnetic stirring for 6 h under argon. The mixture was cooled down to r.t. and the solvent was removed under reduced pressure. The residue was chromatographed on silica gel (hexane/CH_2_Cl_2_ 2 : 1), the colored band was collected and evaporated to dryness under reduced pressure. The residue was suspended in hexane (15 ml), the suspension was centrifuged, the supernatant was pipetted off, and the operation was repeated twice more using 15 ml of hexane each time. The precipitate was dried under vacuum to afford CuPc-Windmill as a dark blue powder (194 mg, 18%). The combined hexane supernatants were evaporated to dryness under reduced pressure, and the residue was chromatographed on silica gel (hexane/toluene 8 : 1 to 1 : 1). Evaporation of appropriately pooled fractions followed by recrystallisation, filtration of the precipitates, and drying under vacuum gave CuPc-Dragon (262 mg, 25%, recrystallized from CHCl_3_/MeOH by slow evaporation at r.t. in darkness) and CuPc-Frog (215 mg, 21%, recrystallized from CHCl_3_/EtOH by slow evaporation at r.t. in darkness) as dark green powders. Characterization data for CuPc-Windmill. ^1^H NMR (500 MHz, CDCl_3_): *δ* = 14.04 (very br s), 9.80 (br s), 7.87 (s), 7.70 (br s), 1.43 (s) ppm; ^13^C{^1^H} NMR (126 MHz, CDCl_3_): *δ* = 150.4, 142.1, 121.9, 121.7, 35.2, 31.9 ppm; HRMS (ESI): *m*/*z* calcd for C_88_H_96_N_8_Cu^+^: 1327.70485 M^+^, found: 1327.70390; UV-vis (toluene, nm (l mol^−1^ cm^−1^)): *λ*_max_ (*ε*) 340 (48900), 687 (226100). Characterization data for CuPc-Dragon. ^1^H NMR (500 MHz, CDCl_3_): *δ* = 15.63 (very br s), 12.52 (very br s), 10.72 (br s), 9.50 (br s), 7.89 (s), 7.83 (s), 7.74 (br s), 7.21 (s), 1.44 (s), 1.40 (s), 1.16 (s) ppm; ^13^C{^1^H} NMR (126 MHz, CDCl_3_): *δ* = 150.5, 149.5, 141.9, 139.3, 129.1, 128.3, 122.5, 121.7, 121.65, 120.3, 35.3, 35.2, 34.8, 31.9, 31.8, 31.75 ppm; HRMS (ESI): *m*/*z* calcd for C_88_H_96_N_8_Cu^+^: 1327.70485 M^+^, found: 1327.70418; UV-vis (toluene, nm (l mol^−1^ cm^−1^)): *λ*_max_ (*ε*) 340 (48800), 707 (200600). Characterization data for CuPc-Frog. ^1^H NMR (500 MHz, CDCl_3_): *δ* = 16.60 (very br s), 13.50 (very br s), 10.93 (br s), 9.32 (br s), 7.86 (s), 7.72 (br s), 7.19 (s), 1.42 (s), 1.13 (s) ppm; ^13^C{^1^H} NMR (126 MHz, CDCl_3_): *δ* = 150.6, 149.5, 142.1, 139.3, 122.4, 121.7, 120.2, 35.2, 34.7, 31.9, 31.7 ppm; HRMS (ESI): *m*/*z* calcd for C_88_H_96_N_8_Cu^+^: 1327.70485 M^+^, found: 1327.70468; UV-vis (toluene, nm (l mol^−1^ cm^−1^)): *λ*_max_ (*ε*) 340 (49500), 706 (183600).

#### Reaction with CuCl_2_·2H_2_O

A mixture of 3,5-di-(*tert*-butyl)phenyl-2,3-dicyanobenzene (100 mg, 0.3163 mmol), copper(ii) chloride dihydrate (17 mg, 0.0997 mmol), and DBU (100 mg, 0.657 mmol) in 1-pentanol (4 ml) was sparged with argon at r.t. for 20 min and stirred magnetically at 150 °C for 5 h under argon. The mixture was cooled down to r.t. and the solvent was removed under reduced pressure. The residue was chromatographed on silica gel (hexane/CH_2_Cl_2_ 2 : 1), the colored band was collected and evaporated to dryness under reduced pressure. The residue was suspended in hexane (4 ml), the suspension was centrifuged, the supernatant was pipetted off, and the operation was repeated twice more using 4 ml of hexane each time. The precipitate was dried under vacuum to afford CuPc-Windmill as a dark blue powder (21 mg, 20%). The combined hexane supernatants were evaporated to dryness under reduced pressure, and the residue was chromatographed on silica gel (hexane/toluene 8 : 1 to 1 : 1). Evaporation of appropriately pooled fractions and subsequent drying under vacuum gave CuPc-Dragon (28 mg, 27%) and CuPc-Frog (21 mg, 20%) as dark green powders. The analytical data (TLC mobility, NMR, UV-vis) for the materials obtained match that for the compounds produced in the phthalonitrile tetramerization reaction performed with CuBr_2_ (*vide supra*).

#### Reaction with Co(OAc)_2_·4H_2_O

A mixture of 3,5-di-(*tert*-butyl)phenyl-2,3-dicyanobenzene (100 mg, 0.3163 mmol), cobalt(ii) acetate tetrahydrate (25 mg, 0.100 mmol), and DBU (48 mg, 0.316 mmol) in 1-pentanol (4 ml) was sparged with argon at r.t. for 10 min and stirred magnetically at 150 °C for 5.5 h under argon. The mixture was cooled down to r.t. and the solvent was removed under reduced pressure. The residue was chromatographed on silica gel (hexane/CH_2_Cl_2_ 2 : 1), the colored band was collected and evaporated to dryness under reduced pressure. The residue was suspended in hexane (4 ml), the suspension was centrifuged, the supernatant was pipetted off, and the operation was repeated twice more using 4 ml of hexane each time. The precipitate was dried under vacuum to afford CoPc-Windmill as a dark blue powder (11 mg, 11% nominal yield). The combined hexane supernatants were evaporated to dryness under reduced pressure, and the residue was subjected to preparative TLC (hexane/toluene 2 : 1) to give CoPc-Dragon (11 mg, 11% nominal yield) and CoPc-Frog (9 mg, 9% nominal yield) as dark green powders. The ^1^H and ^13^C{^1^H} NMR spectra for the materials obtained essentially match those previously reported,^8^ however, unidentified impurities and grease were present.

#### Reaction with NiCl_2_

A mixture of 3,5-di-(*tert*-butyl)phenyl-2,3-dicyanobenzene (100 mg, 0.3163 mmol), nickel(ii) chloride (13 mg, 0.100 mmol), and DBU (100 mg, 0.657 mmol) in 1-pentanol (4 ml) was sparged with argon at r.t. for 20 min and stirred magnetically at 150 °C for 5 h under argon. The mixture was cooled down to r.t. and the solvent was removed under reduced pressure. The residue was chromatographed on silica gel (hexane/CH_2_Cl_2_ 2 : 1), the colored band was collected and evaporated to dryness under reduced pressure to give a mixture of the three regioisomeric NiPcs as a dark green powder (7 mg, 7% nominal yield). The separation of the regioisomers was not attempted. The regioisomeric ratio estimated by integration of a ^1^H NMR spectrum of the obtained mixture was *ca.* 1 : 2 : 1 for NiPc-Windmill, NiPc-Dragon, and NiPc-Frog, respectively. Unidentified impurities were present in the ^1^H NMR spectrum. HRMS (ESI): *m*/*z* calcd for C_88_H_96_N_8_NiNa^+^: 1345.70036 [M + Na]^+^, found: 1345.69849.

#### Reaction with PdCl_2_

A mixture of 3,5-di-(*tert*-butyl)phenyl-2,3-dicyanobenzene (100 mg, 0.3163 mmol), palladium(ii) chloride (17 mg, 0.0959 mmol), and DBU (100 mg, 0.657 mmol) in 1-pentanol (4 ml) was sparged with argon at r.t. for 15 min and stirred magnetically at 150 °C for 16.5 h under argon. The mixture was cooled down to r.t. and the solvent was removed under reduced pressure. The residue was chromatographed on silica gel (hexane/toluene 2 : 1), the colored band was collected and evaporated to dryness under reduced pressure to give a mixture of the three regioisomeric PdPcs as a dark green powder (9 mg, 9% nominal yield). The separation of the regioisomers was not attempted. An unidentified impurity was present according to TLC analysis (*R*_f_ 0.6 (hexane/toluene 2 : 1)). *R*_f_ values for the PdPcs (hexane/toluene 2 : 1): PdPc-Windmill, 0.7; PdPc-Dragon, 0.5; PdPc-Frog, 0.4.

#### Reaction with LiBr

A mixture of 3,5-di-(*tert*-butyl)phenyl-2,3-dicyanobenzene (100 mg, 0.3163 mmol), lithium bromide (21 mg, 0.242 mmol), and DBU (100 mg, 0.657 mmol) in 1-pentanol (4 ml) was sparged with argon at r.t. for 15 min and stirred magnetically at 150 °C for 5 h under argon. The mixture was cooled down to r.t., diluted with EtOAc (5 ml), washed with water (2 × 10 ml), dried over Na_2_SO_4_, filtered and evaporated to dryness under reduced pressure. The residue was chromatographed on silica gel (hexane/CH_2_Cl_2_ 2 : 1), the colored band was collected and evaporated to dryness under reduced pressure. The residue was suspended in hexane (4 ml), the suspension was centrifuged, the supernatant was pipetted off, and the operation was repeated twice more using 4 ml of hexane each time. The precipitate was dried under vacuum to afford H_2_Pc-Windmill as a turquoise powder (18 mg, 18%). The combined hexane supernatants were evaporated to dryness under reduced pressure, and the residue was chromatographed on silica gel (hexane/CH_2_Cl_2_ 8 : 1 to 2 : 1). Evaporation of appropriately pooled fractions followed by recrystallisation from CH_2_Cl_2_/MeCN by slow evaporation at r.t. in darkness, filtration of the precipitates, and drying under vacuum gave H_2_Pc-Dragon (2 mg, 2%) and H_2_Pc-Frog (5 mg, 5%) as dark green powders. The ^1^H NMR spectra for the materials obtained match those previously reported.^8^

#### Reaction with LiBH_4_

A mixture of 3,5-di-(*tert*-butyl)phenyl-2,3-dicyanobenzene (54 mg, 0.171 mmol), lithium tetrahydroborate (14 mg, 0.642 mmol), and DBU (63 mg, 0.414 mmol) in 1-pentanol (3 ml) was sparged with argon at r.t. for 15 min and stirred magnetically at 150 °C for 3 h under argon. The mixture was cooled down to r.t., diluted with EtOAc (3 ml), washed with water (2 × 5 ml), dried over Na_2_SO_4_, filtered and evaporated to dryness under reduced pressure. The residue was chromatographed on silica gel (hexane/CH_2_Cl_2_ 2 : 1), the colored band was collected and evaporated to dryness under reduced pressure to give a mixture of the three regioisomeric H_2_Pcs as a dark green powder (14 mg, 26%). The separation of the regioisomers was not attempted.

### Single crystal X-ray diffraction analysis

Single crystals suitable for X-ray diffraction analysis were grown by slow layer diffusion between CHCl_3_ and MeOH (NiPc-Windmill) and by slow evaporation at r.t. from CHCl_3_/EtOH (H_2_Pc-Windmill) and CHCl_3_/MeOH/pyridine ([CoPc-Dragon(py)_2_]). The single crystal X-ray diffraction data were recorded at 120 K on a Rigaku XtaLAB Synergy diffractometer (Neu-Isenburg, Germany) with mirror-monochromated Cu-*K*_α_ radiation (*λ* = 1.54184 Å) and a hybrid pixel array detector (HyPix). The samples were mounted on LithoLoops produced by MolecularDimensions (Sheffield, UK) and fixed on pins produced by Hampton Research (Aliso Viejo, CA, USA). The absorption correction was performed using CrysAlisPro (different versions; Rigaku OD, 2022, Tokyo, Japan) with the Gaussian method. All structures were solved by dual methods with SHELXT and refined by full-matrix least-squares techniques using the SHELXL-2019 executables and the WingX GUI.^77–80^ For all three crystal structures, diffuse electron density in the channel-like voids originating from residual solvent molecules was removed with PLATON/SQUEEZE.^81^ Details for the crystals, data collection, and refinements are provided in the SI (pages S61–S78, Tables S2–S5).

### Singlet oxygen generation quantum yield measurements

Singlet oxygen luminescence was measured using a time-correlated single-photon counting (TCSPC) system Fluotime 300 (Pico-Quant GmBH, Germany). The system consisted of a PicoHarp 330 controller and a PDL 820 driver. The samples were placed in 1 cm^2^ SOG cuvette and excited at 640 nm with the pulsed diode laser head LDH-D-C-640. The singlet oxygen emission was recorded using a near-infrared photomultiplier tube (NIR PMT, Hamamatsu, Japan) in the 1220–1350 nm spectral range, with a distinct maximum detected at 1270 nm. Conventional unsubstituted Zn phthalocyanine (ZnPc) was used as a reference (58% singlet oxygen quantum yield in toluene)^41^ to determine the singlet oxygen quantum yield of PdPc-Windmill, PdPc-Frog, and PdPc-Dragon. In brief, the absorption spectra of PdPc-Windmill, PdPc-Frog, PdPc-Dragon, and ZnPc in toluene were recorded at three different concentrations per compound, ensuring that the optical density did not exceed 0.2 in any case. The samples were excited at 640 nm, and the emission spectra of singlet oxygen were measured. The integrated emission intensity (from 1220 to 1350 nm) was plotted against the absorbance of a sensitizer at 640 nm. The singlet oxygen quantum yield was then calculated using the following equation:
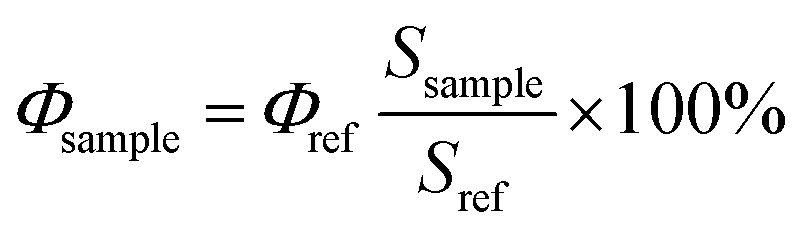
where, *Φ*_sample_ is the singlet oxygen quantum yield of the sample in toluene, *Φ*_ref_ is the singlet oxygen quantum yield of the ZnPc in toluene (58%), *S* is the slope of the plot of absorbance at 640 nm *vs.* integrated emission intensity.

### Preparation and characterization of liposomes

Liposomes were prepared by the thin-film hydration method^75^ by using a lipid composition of DOTAP : DSPE-PEG2K : Chol (in a molar ratio of 45 : 5 : 50). 20 µmol of lipids and 0.2 µmol of PdPc-Windmill, PdPc-Dragon, PdPc-Frog, or VERT dissolved in chloroform were mixed, and chloroform was evaporated under 450 ± 10 mbar vacuum for 40 minutes, and then, 85 ± 10 mbar vacuum for 10 minutes at 67 °C by using a rotary evaporator (Büchi Rotavapor R-100, Büchi Vacuum Pump V-100, Büchi Labortechnik AG, Switzerland). The lipid film was hydrated with 1000 µl of HEPES buffer (20 mM HEPES, 140 mM NaCl, pH 7.4) for the characterization of liposomes, or with 1000 µl of calcein solution (60 mM, 280 mOsm, pH 7.4) for the calcein release study. Hydration was performed in a water bath at 67 °C, and samples were frequently vortexed. PdPc-containing samples were also sonicated in an ultrasonic water bath (Sonorex Digiplus DL 102 H, Bandelin, Germany) for 1 minute after each vortexing to improve the hydration process. Liposomes were extruded 11 times through 800, 400 or 200, and 100 nm poresized polycarbonate membranes (Whatman®, Cytiva, USA) with Mini-Extruder (Avanti Research, USA) to prepare a unilamellar and homogenic liposome formulation. Liposomes were also purified by size-exclusion chromatography to remove unencapsulated molecules. In purification, the HEPES buffer was used as an eluent in a column (Econo-Column 1.0 cm x 20 cm, Bio-Rad, USA) packed with Sephadex® G-50 medium. The final lipid concentration in liposome solution was 2.0 mM. After preparation, the liposomes were protected from light and stored at +4 °C. Three independent batches of liposomes were prepared for the experiments described below, unless otherwise stated. Each biological replicate was run in three technical replicates.

Particle size, polydispersity index (PDI), and zeta potential of liposomes were measured using a Zetasizer Nano instrument (Malvern Panalytical, UK). For the determination of particle size and PDI, 10 µl of liposomes were diluted in 990 µl of HEPES buffer. For zeta potential, 20 µl of liposomes were diluted in 980 µl of deionized water, and a DTS1070 folded capillary cell (Malvern Panalytical, UK) was used in the measurements. One replication was conducted to determine the zeta potential of PdPc-Windmill liposomes, and two replications for others.

Encapsulation efficiencies (EE%s) of PSs in liposomes were determined based on absorbances of PSs. Previously, a molar absorption coefficient of each PS was determined by absorbance spectra of standard samples in a pyridine-HEPES buffer solution (3 : 1 (v/v)) with multiple concentrations of PS. The spectra were recorded at wavelengths from 400 to 800 nm with a UV-vis spectrophotometer (UV-3600, Shimadzu, Japan). Absorbances of PS at the wavelength corresponding to the absorption maximum were plotted against concentrations of standard samples, and the molar absorption coefficient was determined from a slope of that linear calibration curve. Absorbance of PS in a pyridine–liposome solution (3 : 1 (v/v)) were measured at the absorption maximum, and EE% of each PS in liposomes was calculated by using the following equation:
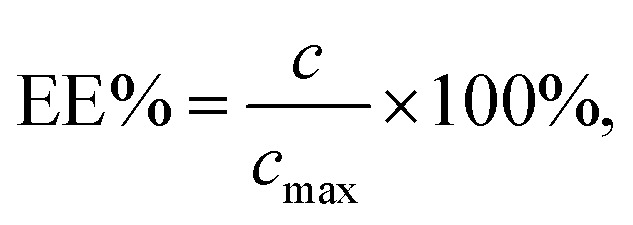
where *c* is the concentration of PS in liposomes based on the Beer–Lambert's law and the molar absorption coefficient of PS, and *c*_max_ is the calculated maximum concentration of PS in liposomes. Absorbances were measured in a mixture of pyridine and HEPES buffer as all used PSs had a low water solubility.

The stability of liposomes was determined based on the passive calcein release, and the changes in particle size, PDI, and absorbance of PSs. Measurements were performed as described above on days 1, 7, 14, 21, and 28 for the liposomes stored at 4 °C, and on days 1, and 7 for the liposomes stored at 37 °C. The stability of PdPc-Windmill liposomes was not determined due to the low encapsulation of PS.

### Calcein release study

The calcein release study was performed as described earlier^75^ with some exceptions. Liposomes were diluted in HEPES buffer at a 1 : 10 (v/v) ratio to prevent the self-quenching of calcein, and liposomes were illuminated on a white 96-well plate with an illumination system^40^ developed by our research group. Experiments in oxygen-deficient conditions were conducted using potent ground state oxygen scavenger sodium sulfite (20 mM), with one biological replicate. Based on the wavelength of maximum absorbance of PSs, PdPc-Windmill liposomes were exposed to 660 nm light, and other liposomes were illuminated with a wavelength of 690 nm. Power densities of 25 and 75 mW cm^−2^ were used for liposomes containing PdPc-Dragon, PdPc-Frog, or VERT. For PdPc-Windmill liposomes, only a power density of 28 mW cm^−2^ was used for practical reasons. Multiple light doses were used for each PSs. The illumination was performed at 37 °C.

The fluorescence of released calcein was measured on a black 96-well plate with Fluoroskan Ascent (Thermo Fisher Scientific, USA) (ex. 485 nm, em. 538 nm, for practical reasons). To determine the total calcein content and the relative calcein release, liposomes were degraded with 10 µl of 10% Triton X-100 solution, and the fluorescence was measured again. The relative calcein release from liposomes was calculated by using the following equation:
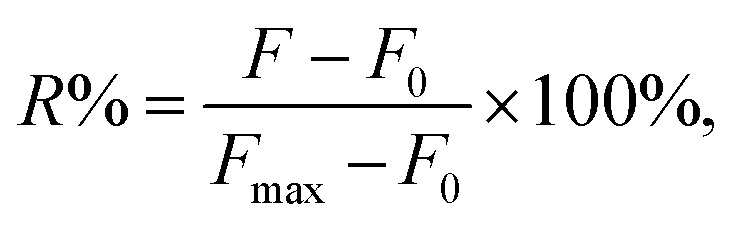
where *R*% is the relative calcein release, *F* is the fluorescence of calcein from illuminated sample, *F*_0_ is the fluorescence of calcein from non-illuminated control sample stored at 4 °C, and *F*_max_ is the fluorescence of calcein after addition of 10% Triton X-100.^75^

### Data analysis

The data for the calcein release study were analyzed with IBM SPSS Statistics v. 31.0.1.0 (IBM, USA). Outlier assessment was performed using visual inspection of box plots, and their significance was tested with Shapiro–Wilk test at a 5% alpha level. Significant outliers were not detected in the data. The statistical significance of the differences in the calcein release between the PSs was evaluated using one-way ANOVA with Bonferroni correction. Also, the statistical significance of the differences in the absorbance stability between PSs was determined using one-way ANOVA with Bonferroni correction. The results were considered statistically significant for *p*-values below 0.05. The rest of the liposome-related data were analyzed with Microsoft Excel v. 2512 (Microsoft, USA). Graphs were created with Origin 2024b v. 10.1.5.132 (OriginLab, USA).

## Author contributions

Aleksandr Penni: investigation (synthesis, characterisation), writing – original draft; Riikka Rimmistö: investigation (preparation of liposomes, photo-oxidation studies), writing – original draft; Inka Ojala: investigation (synthesis); Sebastian Pätsch: investigation (synthesis, characterisation), writing – review and editing; Benedict Josua Elvers: investigation (SC-XRD measurement and data evaluation); Carola Schulzke: investigation (SC-XRD measurement and data evaluation); Timo Laaksonen: conceptualization, writing – review and editing, project administration, funding acquisition; Nikita Durandin: investigation (singlet oxygen studies), writing – review and editing, funding acquisition; Alexander Efimov: conceptualization, writing – review and editing, project administration, funding acquisition.

## Conflicts of interest

There are no conflicts to declare.

## Supplementary Material

RA-016-D6RA03003C-s001

RA-016-D6RA03003C-s002

## Data Availability

CCDC 2540123–2540125 contain the supplementary crystallographic data for this paper.^82*a*–*c*^ Experimental data (NMR, HR-MS, UV/vis spectra and liposomal stability) supporting this article are included in the supplementary information (SI). Supplementary information is available. See DOI: https://doi.org/10.1039/d6ra03003c.

## References

[cit1] The Porphyrin Handbook, ed. K. M. Kadish, K. M. Smith and R. Guilard, Elsevier, San Diego, California, USA, 2003, vol. 15

[cit2] Handbook of Porphyrin Science, ed. K. M. Kadish, K. M. Smith and R. Guilard, World Scientific Publishing Company, Singapore, 2010, vol. 3

[cit3] Leznoff C. C., Hu M., McArthur C. R., Qin Y., Van Lier J. E. (1994). Can. J. Chem..

[cit4] Leznoff C. C., Hu M., Nolan K. J. M. (1996). Commun. Chem..

[cit5] Iida N., Tanaka K., Tokunaga E., Takahashi H., Shibata N. (2015). ChemistryOpen.

[cit6] Yamamoto S., Kuribayashi K., Murakami T. N., Kwon E., Stillman M. J., Kobayashi N., Segawa H., Kimura M. (2017). Chem.–Eur. J..

[cit7] Mulholland K. D., Yoon S., Rennie C. C., Sitch E. K., McKay A. I., Edkins K., Edkins R. M. (2020). Commun. Chem..

[cit8] Tkachenko N., Golovanov V., Penni A., Vesamäki S., Ajayakumar M. R., Muranaka A., Kobayashi N., Efimov A. (2024). Phys. Chem. Chem. Phys..

[cit9] Tomoda H., Saito S., Ogawa S., Shiraishi S. (1980). Chem. Lett..

[cit10] Tomoda H., Saito S., Shiraishi S. (1983). Chem. Lett..

[cit11] Wöhrle D., Schnurpfeil G., Knothe G. (1992). Dyes Pigm..

[cit12] Rager C., Schmid G., Hanack M. (1999). Chem.–Eur. J..

[cit13] Denekamp I. M., Veenstra F. L. P., Jungbacker P., Rothenberg G. (2019). Appl. Organomet. Chem..

[cit14] Langerreiter D., Kostiainen M. A., Kaabel S., Anaya-Plaza E. (2022). Angew. Chem., Int. Ed..

[cit15] Hutsch S., Niewind M., Grätz S., Borchardt L. (2025). Chem.–Eur. J..

[cit16] Szymczak J., Sobotta L., Dlugaszewska J., Kryjewski M., Mielcarek J. (2021). Dyes Pigm..

[cit17] Szymczak J., Rębiś T., Mielcarek J., Kryjewski M. (2022). Synth. Met..

[cit18] Li X., Peng X.-H., Zheng B.-D., Tang J., Zhao Y., Zheng B.-Y., Ke M.-R., Huang J.-D. (2018). Chem. Sci..

[cit19] Grammatikova N. E., George L., Ahmed Z., Candeias N. R., Durandin N. A., Efimov A. (2019). J. Mater. Chem. B.

[cit20] Lo P.-C., Rodríguez-Morgade M. S., Pandey R. K., Ng D. K. P., Torres T., Dumoulin F. (2020). Chem. Soc. Rev..

[cit21] Galstyan A. (2021). Chem.–Eur. J..

[cit22] Zheng B.-D., He Q.-X., Li X., Yoon J., Huang J.-D. (2021). Coord. Chem. Rev..

[cit23] Castro K. A. D. F., Prandini J. A., Biazzotto J. C., Tomé J. P. C., Da Silva R. S., Lourenço L. M. O. (2022). Front. Chem..

[cit24] Otvagin V. F., Kuzmina N. S., Kudriashova E. S., Nyuchev A. V., Gavryushin A. E., Fedorov A. Yu. (2022). J. Med. Chem..

[cit25] Lima E., Reis L. V. (2023). Molecules.

[cit26] Channabasavana Hundi Puttaningaiah K. P., Hur J. (2024). Inorg. Chem. Commun..

[cit27] Repetowski P., Warszyńska M., Kostecka A., Pucelik B., Barzowska A., Emami A., İşci Ü., Dumoulin F., Dąbrowski J. M. (2024). ACS Appl. Mater. Interfaces.

[cit28] Chornovolenko K., Koczorowski T. (2025). Molecules.

[cit29] Ogunbayo T. B., Nyokong T. (2010). J. Mol. Struct..

[cit30] Van Leeuwen M., Beeby A., Fernandes I., Ashworth S. H. (2013). Photochem. Photobiol. Sci..

[cit31] Dumas A., Couvreur P. (2015). Chem. Sci..

[cit32] Che Y., Yang W., Tang G., Dumoulin F., Zhao J., Liu L., İşci Ü. (2018). J. Mater. Chem. C.

[cit33] Furuyama T., Miyaji Y., Maeda K., Maeda H., Segi M. (2019). Chem.–Eur. J..

[cit34] Kulu I., Mantareva V., Kussovski V., Angelov I., Durmuş M. (2022). J. Mol. Struct..

[cit35] Mantareva V. N., Kussovski V., Orozova P., Dimitrova L., Kulu I., Angelov I., Durmus M., Najdenski H. (2022). Biomedicines.

[cit36] Ziental D., Mlynarczyk D. T., Kolasinski E., Güzel E., Dlugaszewska J., Popenda Ł., Jurga S., Goslinski T., Sobotta L. (2022). Pharmaceutics.

[cit37] Zalevskaya O. A., Gur’eva Ya. A., Kutchin A. V. (2023). Russ. Chem. Rev..

[cit38] Lem O., Gangurde P., Koivuniemi A., Keskinen A., Efimov A., Durandin N., Laaksonen T. (2024). Carbohydr. Polym..

[cit39] Lem O., Kekki R., Koivuniemi A., Efimov A., Laaksonen T., Durandin N. (2024). Mater. Adv..

[cit40] Eftekhari A., Lem O., Efimov A., Laaksonen T., Durandin N. (2025). Analyst.

[cit41] Ranta J., Kumpulainen T., Lemmetyinen H., Efimov A. (2010). J. Org. Chem..

[cit42] Leznoff C. C., Drew D. M. (1996). Can. J. Chem..

[cit43] Szymczak J., Rebis T., Kotkowiak M., Wicher B., Sobotta L., Tykarska E., Mielcarek J., Kryjewski M. (2021). Dyes Pigm..

[cit44] Kimura T., Kudo C., Nakajo S. (2019). Eur. J. Inorg. Chem..

[cit45] Vagin S., Hanack M. (2004). Eur. J. Org Chem..

[cit46] Tillo A., Stolarska M., Kryjewski M., Popenda L., Sobotta L., Jurga S., Mielcarek J., Goslinski T. (2016). Dyes Pigm..

[cit47] Zimcik P., Malkova A., Hruba L., Miletin M., Novakova V. (2017). Dyes Pigm..

[cit48] Da Silva R. N., Cunha Â., Tomé A. C. (2018). Eur. J. Med. Chem..

[cit49] Carvalho E. F. A., Calvete M. J. F., Tomé A. C., Cavaleiro J. A. S. (2009). Tetrahedron Lett..

[cit50] Hurley T. J., Robinson M. A., Trotz S. I. (1967). Inorg. Chem..

[cit51] Oliver S. W., Smith T. D. (1987). J. Chem. Soc. Perkin Trans. 2.

[cit52] Nolan K., Hu M., Leznoff C. (1997). Synlett.

[cit53] Fukuda T., Shigeyoshi N., Fuyuhiro A., Ishikawa N. (2013). Dalton Trans..

[cit54] Fukuda T., Kikukawa Y., Fuyuhiro A., Kobayashi N., Ishikawa N. (2014). Chem. Lett..

[cit55] Ishizaki T., Fukuda T., Akaki M., Fuyuhiro A., Hagiwara M., Ishikawa N. (2019). Inorg. Chem..

[cit56] Molek C. D., Halfen J. A., Loe J. C., McGaff R. W. (2001). Commun. Chem..

[cit57] Fukuda T., Kikukawa Y., Tsuruya R., Fuyuhiro A., Ishikawa N., Kobayashi N. (2011). Inorg. Chem..

[cit58] Kikukawa Y., Fukuda T., Fuyuhiro A., Ishikawa N., Kobayashi N. (2011). Commun. Chem..

[cit59] Karabach Y. Y., Kopylovich M. N., Luzyanin K. V., Guedes Da Silva M. F. C., Kukushkin V. Yu., Pombeiro A. J. L. (2017). Inorg. Chim. Acta.

[cit60] Nogita K., Sugahara T., Miki K., Mu H., Kobayashi M., Harada H., Ohe K. (2024). Commun. Chem..

[cit61] Nogita K., Kataoka Y., Sugahara T., Miki K., Mu H., Ohe K. (2025). Chem.–Eur. J..

[cit62] Lv W., Duan J., Ai S. (2019). Chin. Chem. Lett..

[cit63] Kasuga K., Asano K., Lin L., Sugimori T., Handa M., Abe K., Kikkawa T., Fujiwara T. (1997). Bull. Chem. Soc. Jpn..

[cit64] Ruf M., Lawrence A. M., Noll B. C., Pierpont C. G. (1998). Inorg. Chem..

[cit65] Engel M. K. , in The Porphyrin Handbook, Elsevier, San Diego, California, USA, 2003, vol. 20, pp. 1–242

[cit66] Chen Y., Fang W., Wang K., Liu W., Jiang J. (2016). Inorg. Chem..

[cit67] Peterson M., Hunt C., Wang Z., Heinrich S. E., Wu G., Ménard G. (2020). Dalton Trans..

[cit68] Sugimori T., Okamoto S., Kotoh N., Handa M., Kasuga K. (2000). Chem. Lett..

[cit69] Cariati F., Morazzoni F., Zocchi M. (1978). J. Chem. Soc., Trans..

[cit70] Janczak J., Kubiak R. (2003). Inorg. Chim. Acta.

[cit71] Danaei M., Dehghankhold M., Ataei S., Hasanzadeh Davarani F., Javanmard R., Dokhani A., Khorasani S., Mozafari M. R. (2018). Pharmaceutics.

[cit72] Smith M. C., Crist R. M., Clogston J. D., McNeil S. E. (2017). Anal. Bioanal. Chem..

[cit73] Massiot J., Makky A., Di Meo F., Chapron D., Trouillas P., Rosilio V. (2017). Phys. Chem. Chem. Phys..

[cit74] Calori I. R., Braga G., Tessaro A. L., Caetano W., Tedesco A. C., Hioka N. (2022). J. Mol. Liq..

[cit75] Lajunen T., Kontturi L.-S., Viitala L., Manna M., Cramariuc O., Róg T., Bunker A., Laaksonen T., Viitala T., Murtomäki L., Urtti A. (2016). Mol. Pharm..

[cit76] Aveline B., Hasan T., Redmond R. W. (1994). Photochem. Photobiol..

[cit77] Sheldrick G. M. (2008). Acta Crystallogr., Sect. A:Found. Crystallogr..

[cit78] Sheldrick G. M. (2015). Acta Crystallogr., Sect. A:Found. Adv..

[cit79] Sheldrick G. M. (2015). Acta Crystallogr., Sect. C: Struct. Chem..

[cit80] Farrugia L. J. (2012). J. Appl. Crystallogr..

[cit81] Spek A. L. (2015). Acta Crystallogr., Sect. C: Struct. Chem..

[cit82] (a) CCDC 2540123: Experimental Crystal Structure Determination, 2026, 10.5517/ccdc.csd.cc2r86gq

