# Supplementary material for: Constrain as an advantage: selective metal-templated synthesis of sterically hindered phthalocyanine photosensitizers and their application for light-triggered release from liposomes
Source: RSC Adv. 2026 Jul 2;16(34):33332–46. doi: 10.1039/d6ra03003c (PMC13326660; doi:10.1039/d6ra03003c)
Supplement: RA-016-D6RA03003C-s001 [file RA-016-D6RA03003C-s001.pdf]

Supplementary Information (SI) for RSC Advances.
This journal is © The Royal Society of Chemistry 2026

Supporting Information

Constrain as an advantage: selective metal-templated synthesis of sterically hindered phthalocyanine photosensitizers and their application for light-triggered release from liposomes

Aleksandr Penni[a], Riikka Rimmistö[b], Inka Ojala[a], Sebastian Pätsch[a], Benedict Josua Elvers[c], Carola Schulzke[c], Timo Laaksonen[b], Nikita Durandin[a], Alexander Efimov[a]

[a] Tampere University, Faculty of Engineering and Natural Sciences, Korkeakoulunkatu 8, 33720 Tampere, Finland.

[b] University of Helsinki, Faculty of Pharmacy, Viikinkaari 5 E, 00790 Helsinki, Finland.

[c] University of Greifswald, Institute of Biochemistry, Bioinorganic Chemistry, Felix-Hausdorff-Str. 4, 17489 Greifswald, Germany.

Table of Contents

Literature examples of Mg^{II}-promoted Pc syntheses 3

Mechanistic considerations for the Mg^{II}-templated phthalonitrile cyclotetramerization reaction 4

Experimental information 5

HR-MS data 5

UV–vis absorption spectra 12

NMR spectra 14

Crystallographic details 61

Liposomal stability 79

PdPc stability in serum 80

Calcein release in the presence of an oxygen scavenger 81

References 82

Literature examples of Mg^{II}-promoted Pc syntheses

Table 1: Comparison of the Mg^{II}-templated phthalonitrile cyclotetramerization reactions reported in the literature. (TMP = 2,2,6,6,-Tetramethylpiperidine; DBU = 1,8-Diazabicyclo(5.4.0)undec-7-ene; tw = this work)

	Substitution at Pc ring	Substituent	Mg source	Solvent	Yield MgPc	Demetalation	Yield H_2Pc	Ref
**1**	α	3,5-di-(*tert*-butyl)phenyl	Mg/I_2	butanol	-	yes	87%	tw
**2**	β	4-(diethylaminosulfonyl)phenoxy	Mg	octanol / pentanol	86%	no	-	1
**3**	β/β’	4-(isopropylaminosulfonyl)phenoxy	Mg	octanol / pentanol	82%	no	-	1
**4**	β/β’	4-(thiazol-2-ylaminosulfonyl)phenoxy	Mg	octanol / pentanol	78%	no	-	1
**5**	β/β’	3-(trifluoromethyl)phenyl	Mg	octanol / pentanol	76%	yes	73%	2
**6**	α/β/β’/α’	α: butoxy / β: 4-mercaptopyridine	Mg/I_2	butanol	60%	yes	38%	3
**7**	β/β’	2,6-bis(diphenyl)benzenethioether	Mg/I_2	butanol	60%	yes	41%	4
**8**	α/β/β’/α’	α: butoxy / β: 2-mercaptopyridine	Mg/I_2	butanol	50%	yes	93%	3
**9**	β/β’	4-(diethylaminosulfonyl)phenoxy	Mg	pentanol	50%	yes	72%	5
**10**	α/β/β’/α’	α: octoxy / β: 2-mercaptopyridine	Mg/I_2	butanol	48%	yes	48%	3
**11**	β/β’	3-(trifluoromethyl)phenoxy	Mg	octanol / pentanol	46%	yes	84%	2
**12**	α/α’	3,5-bis(diphenoxy)phenyl	Mg/I_2	butanol	42%	no	-	6
**13**	α	L-menthol	Mg/I_2	butanol	40%	no	-	7
**14**	β	hexoxy	$MgBr_2$ / TMP	heterogeneous	30%	no	-	8
**15**	α/α’	propoxy	Mg/I_2	butanol	13%	no	-	6
**16**	α/α’	phenoxy	Mg/I_2	butanol	11%	no	-	6
**17**	α/β’	3,5-bis(trifluoromethyl)phenyl	$Mg(OAc)_2$ / DBU	pentanol	5%	no	-	9
**18**	α/β’	3,5-bis(trifluoromethyl)phenyl	$Mg(OAc)_2$ / DBU	pentanol	-	yes	5%	9

Mechanistic considerations for the Mg^{II}-templated phthalonitrile cyclotetramerization reaction

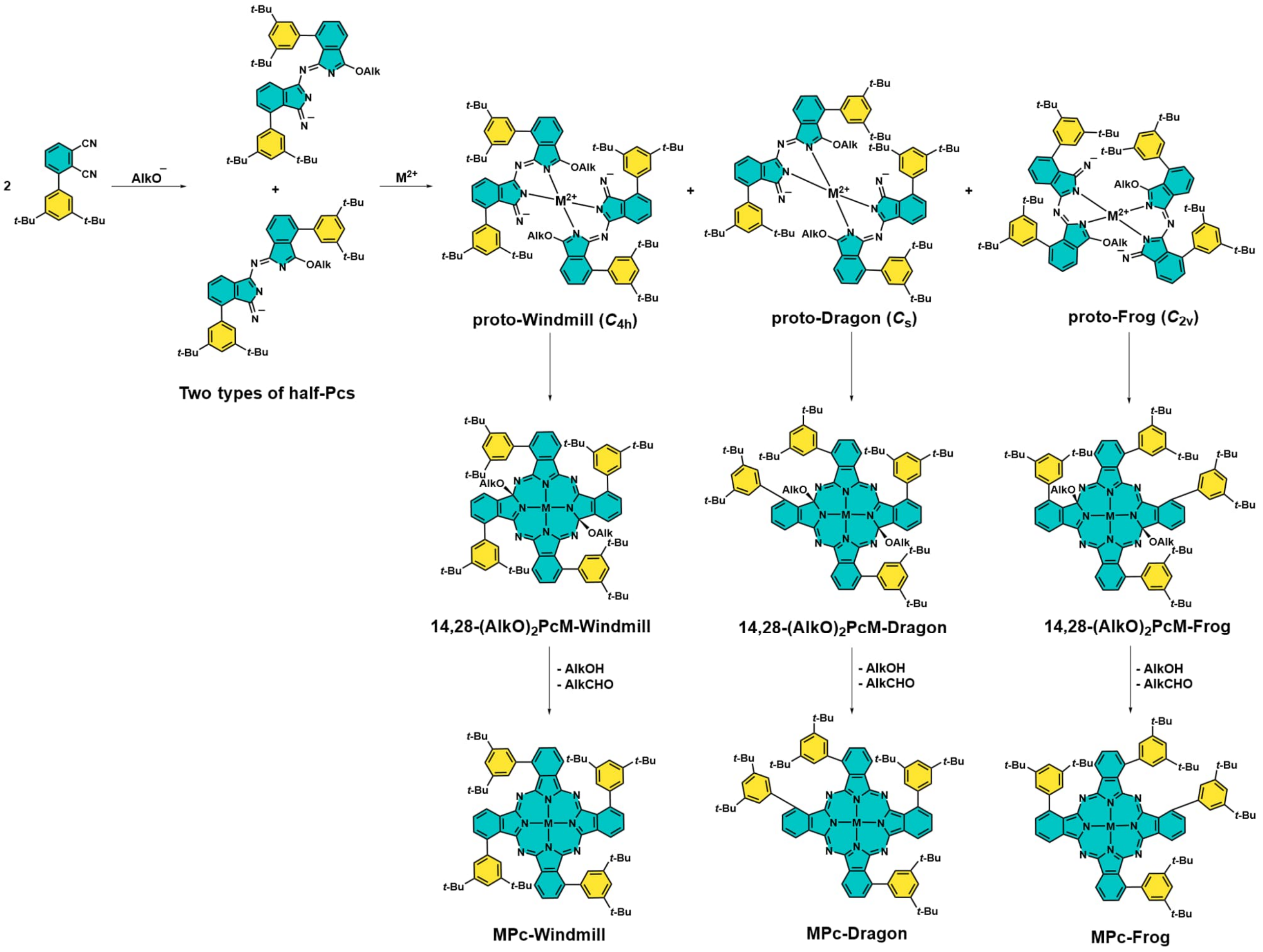

Scheme S1: Plausible mechanism for the formation of the regioisomeric Pcs.

Experimental information

HR-MS data

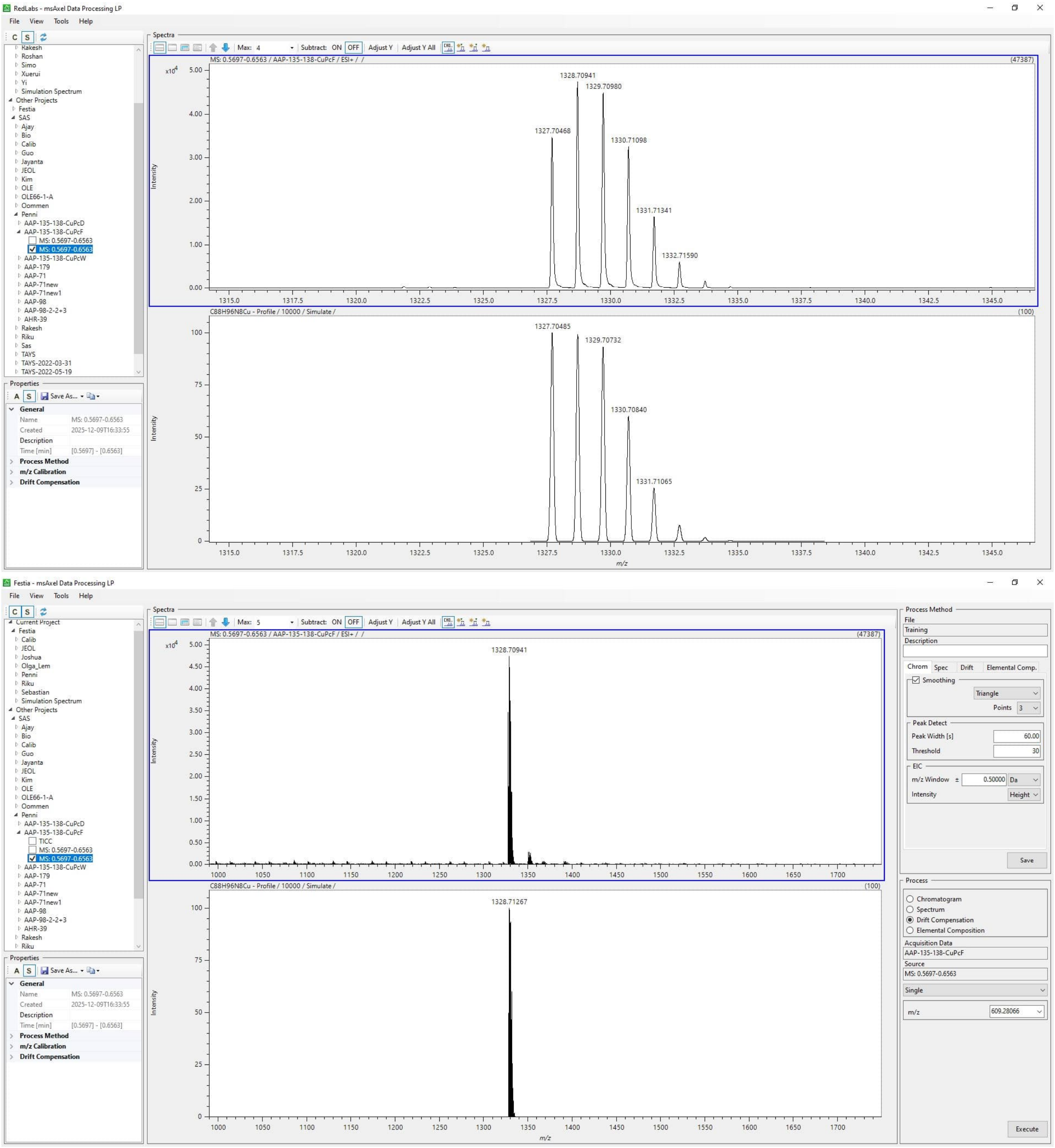

Figure S1: High-resolution ESI-TOF mass spectrum of **CuPc-Frog** vs. the $[M+H]^{+}$ ($C_{88}H_{97}N_8Cu^{+}$) isotope model.

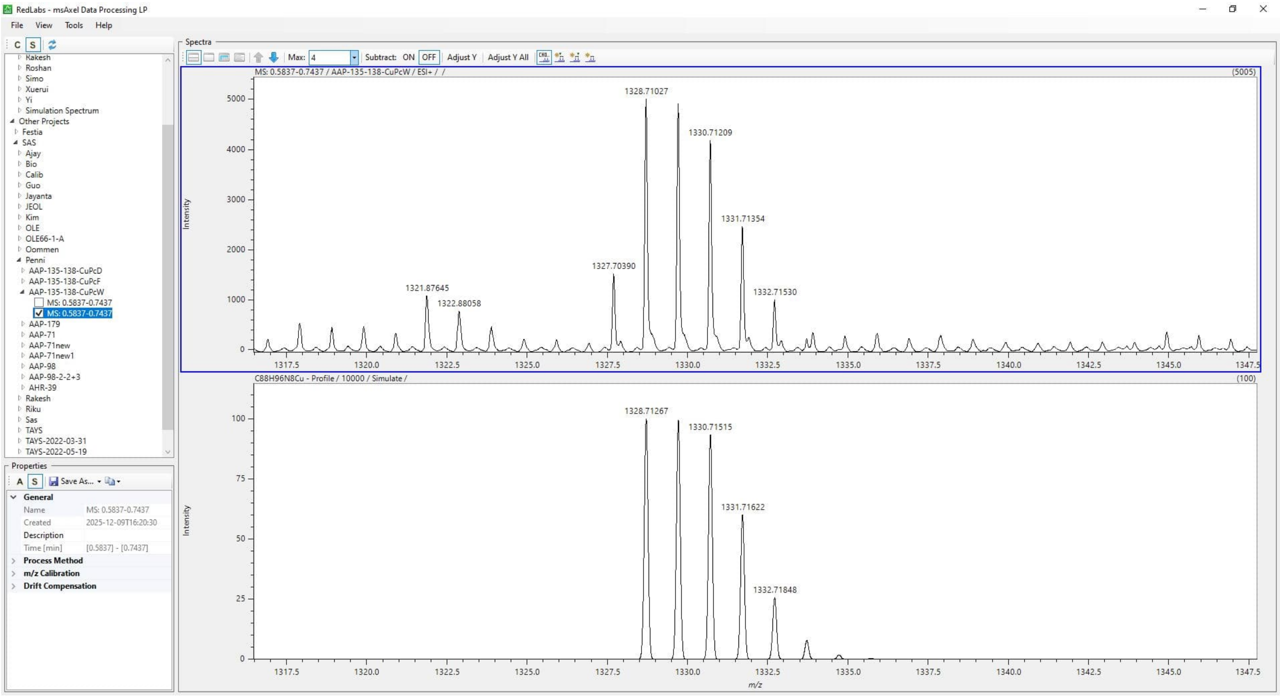

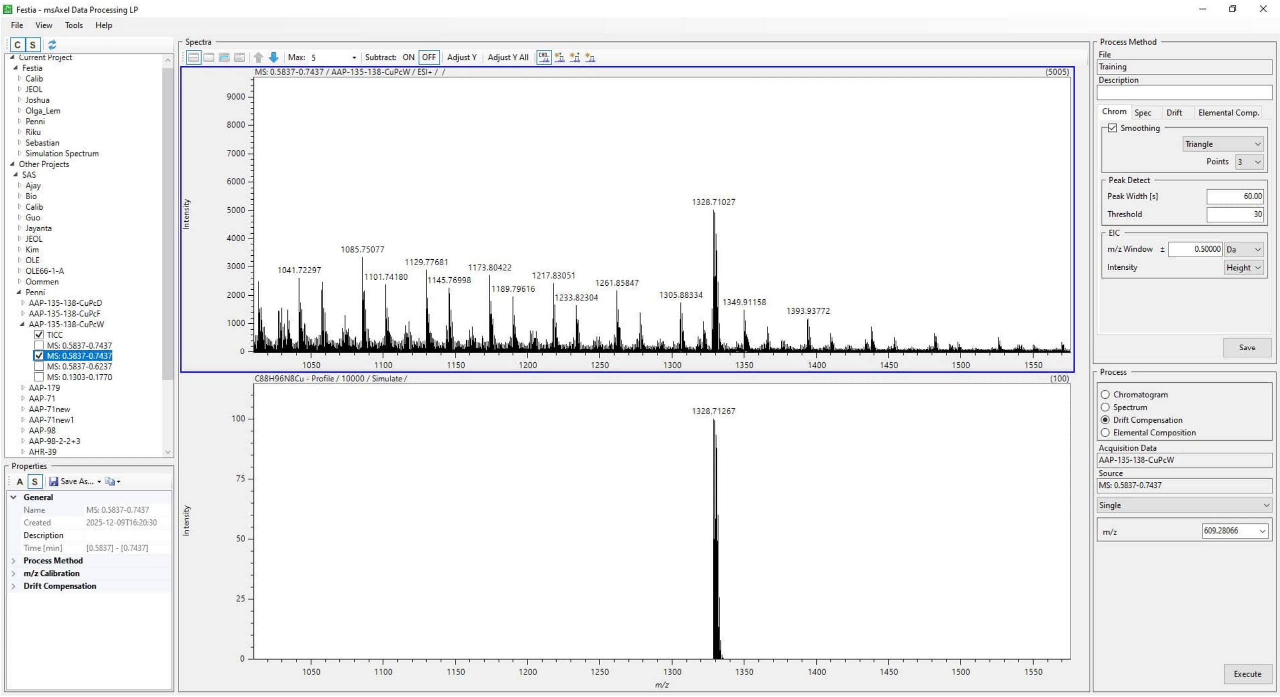

Figure S2: High-resolution ESI-TOF mass spectrum of **CuPc-Windmill** vs. the $[M+H]^+$ ($C_{88}H_{97}N_8Cu^+$) isotope model.

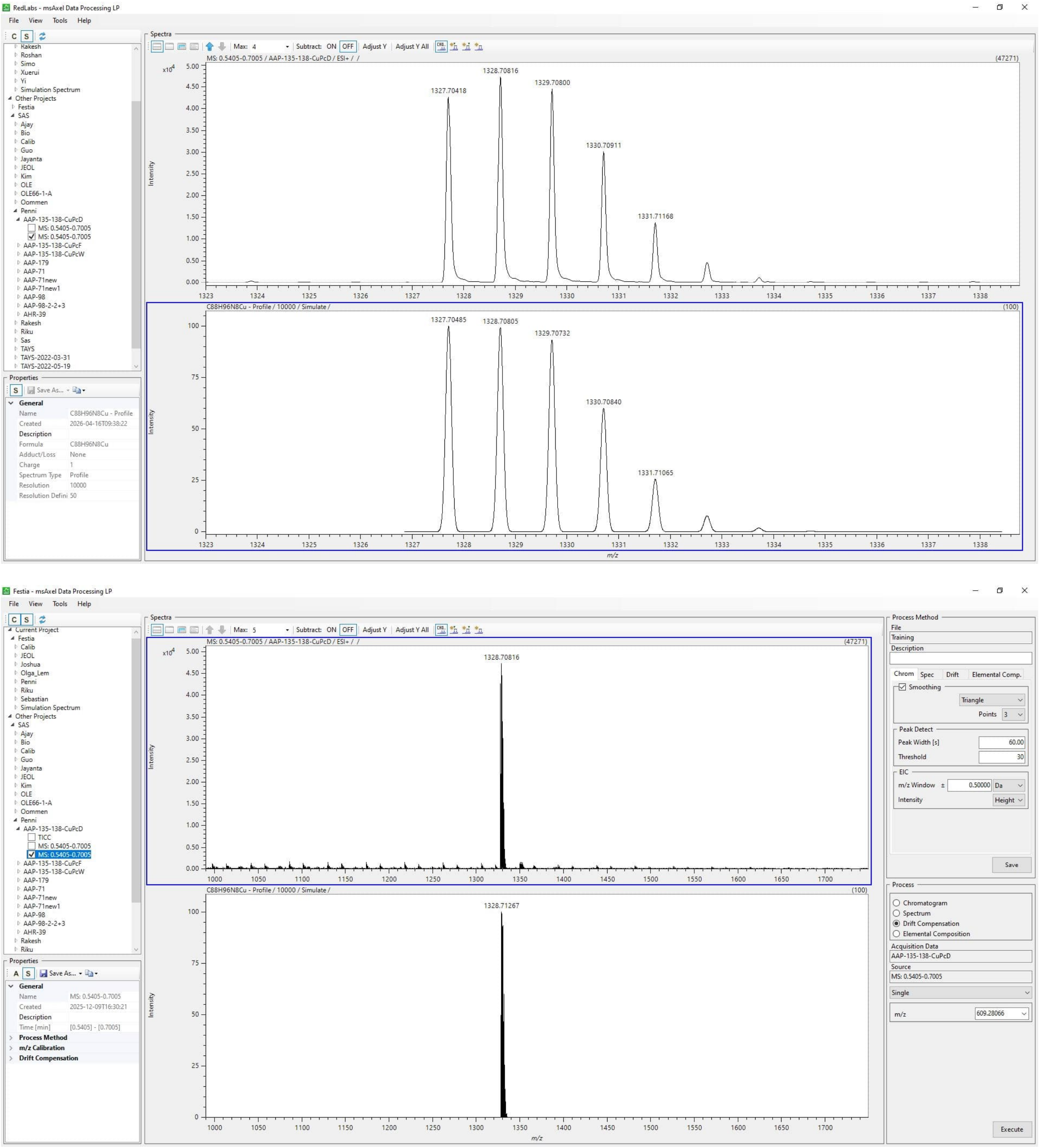

Figure S3: High-resolution ESI-TOF mass spectrum of **CuPc-Dragon** vs. the M$^+$ ($C_{88}H_{96}N_8Cu^+$) and [M+H]$^+$ ($C_{88}H_{97}N_8Cu^+$) isotope models.

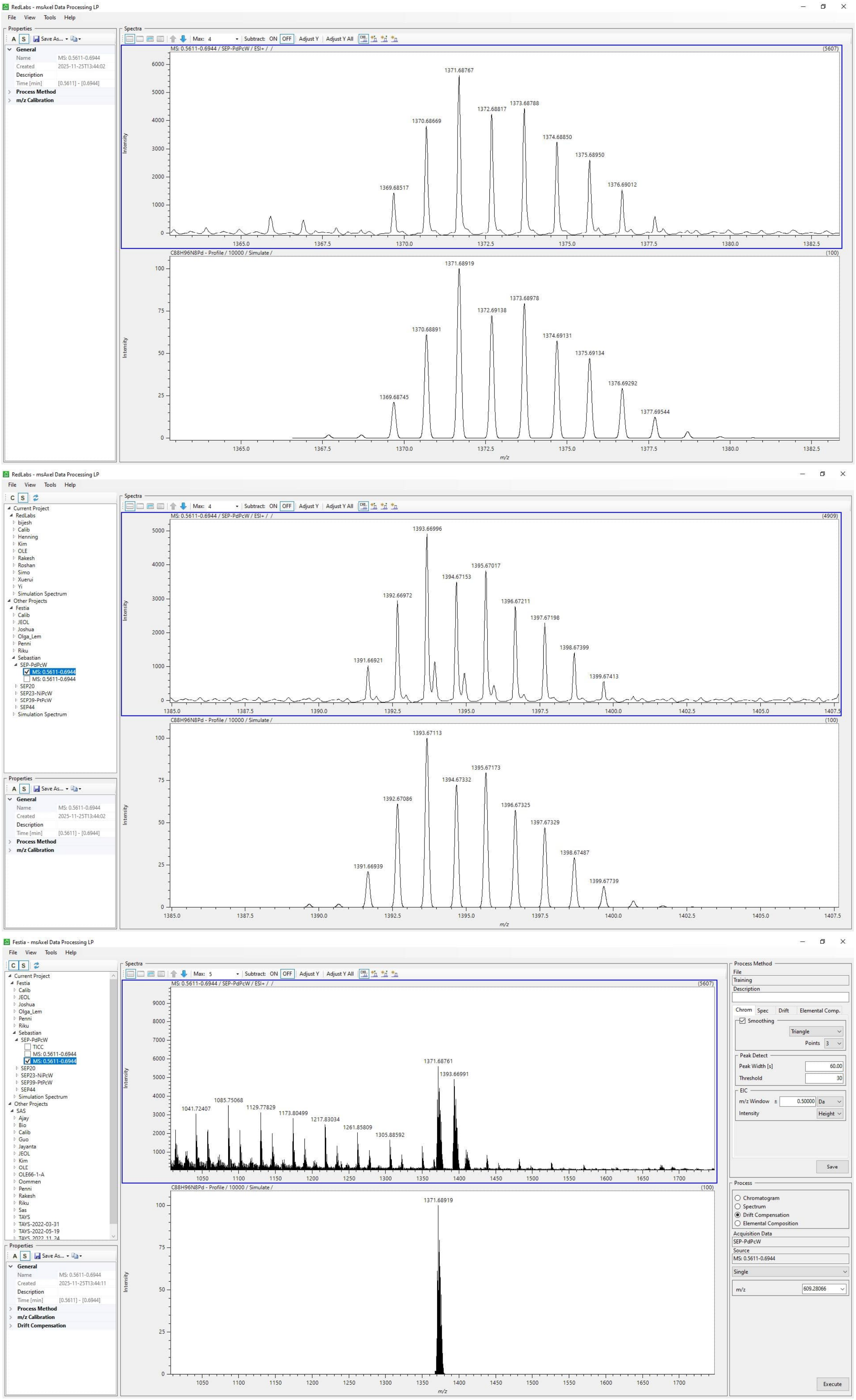

Figure S4: High-resolution ESI-TOF mass spectrum of **PdPc-Windmill** vs. the [M+H]$^+$ ($C_{88}H_{97}N_8Pd^+$) and [M+Na]$^+$ ($C_{88}H_{96}N_8PdNa^+$) isotope models.

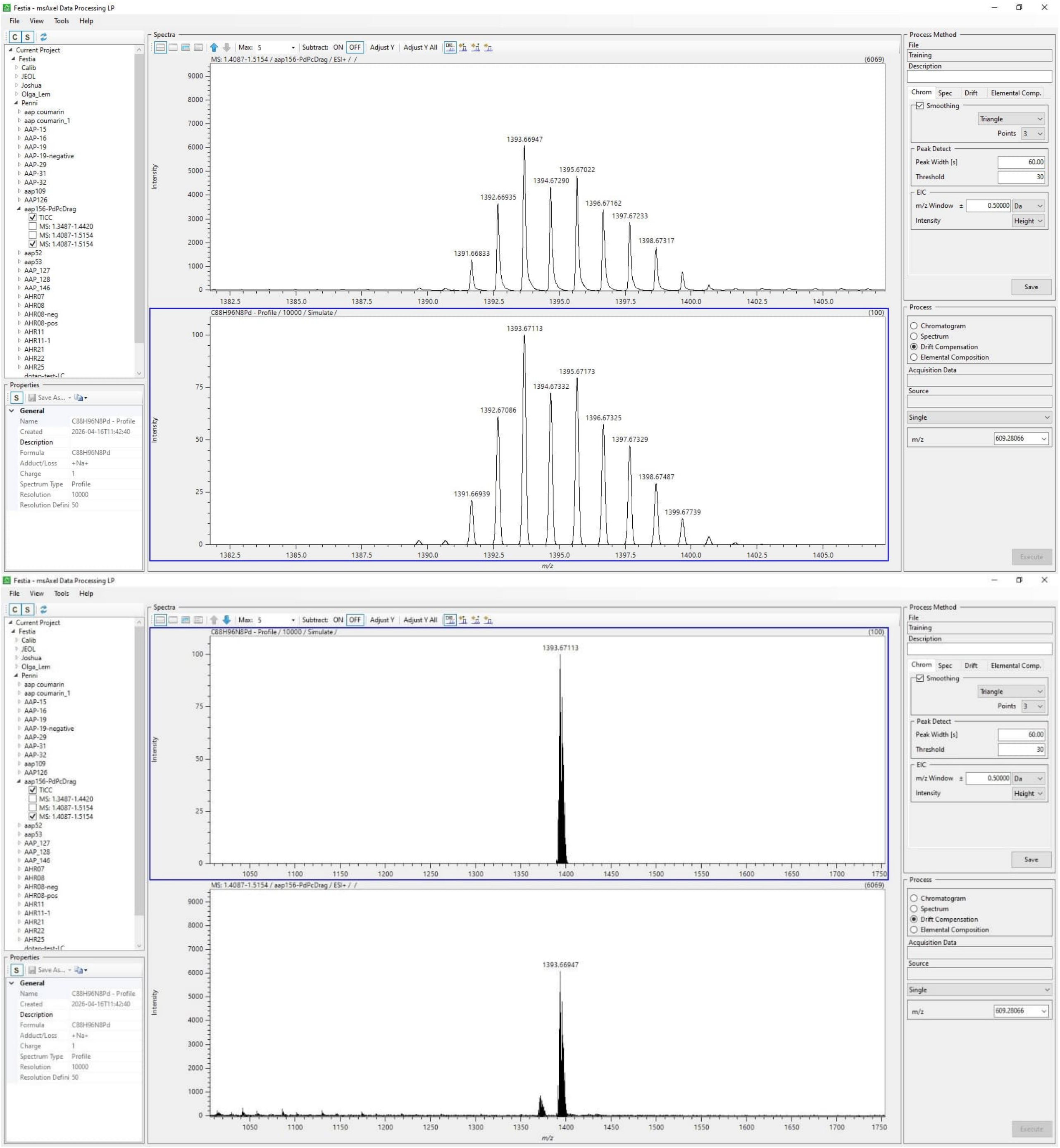

Figure S5: High-resolution ESI-TOF mass spectrum of **PdPc-Dragon** vs. the [M+Na]+ ($C_{88}H_{96}N_8PdNa^+$) isotope model.

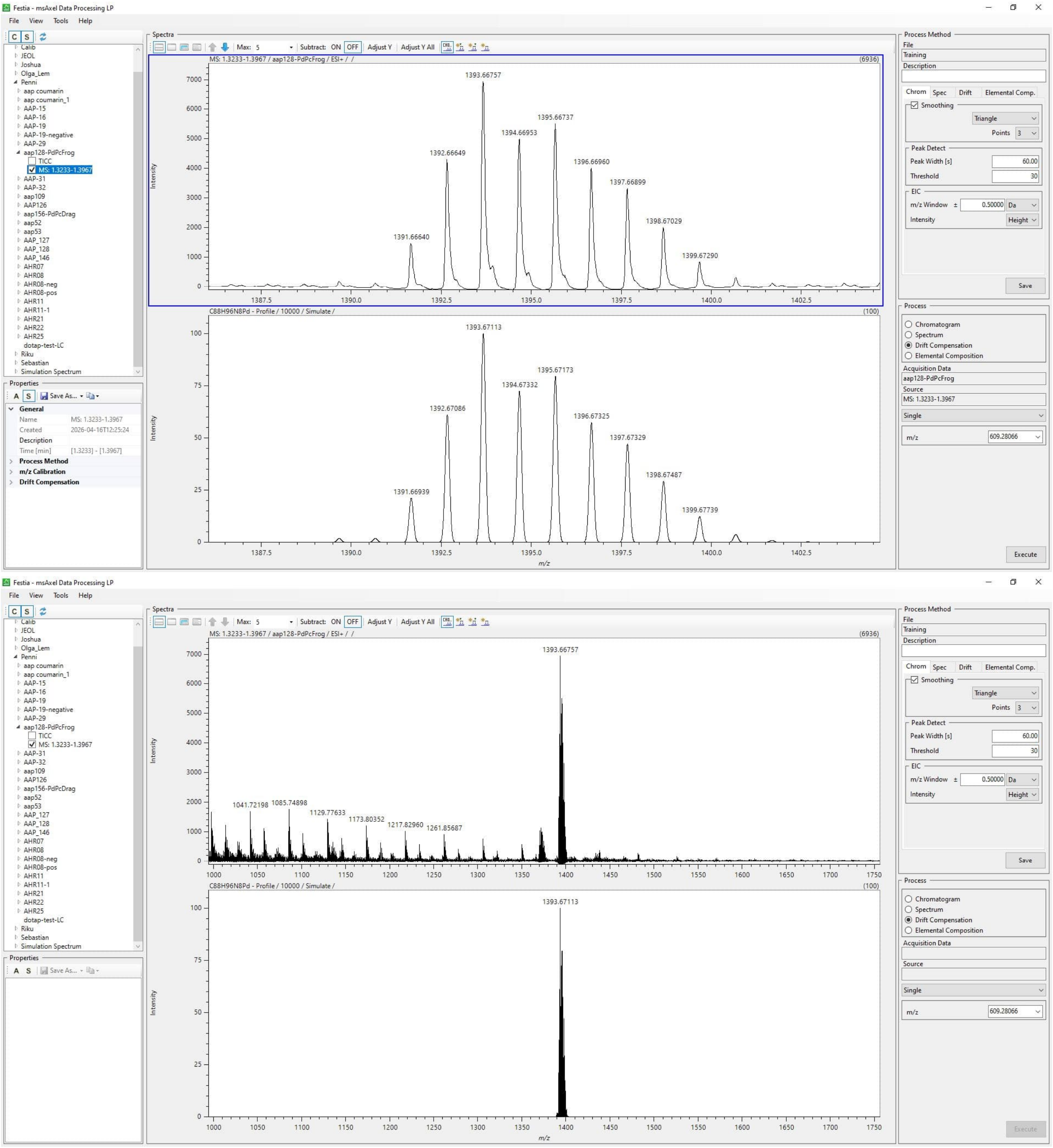

Figure S6: High-resolution ESI-TOF mass spectrum of **PdPc-Frog** vs. the $[M+Na]^+$ ($C_{88}H_{96}N_8PdNa^+$) isotope model.

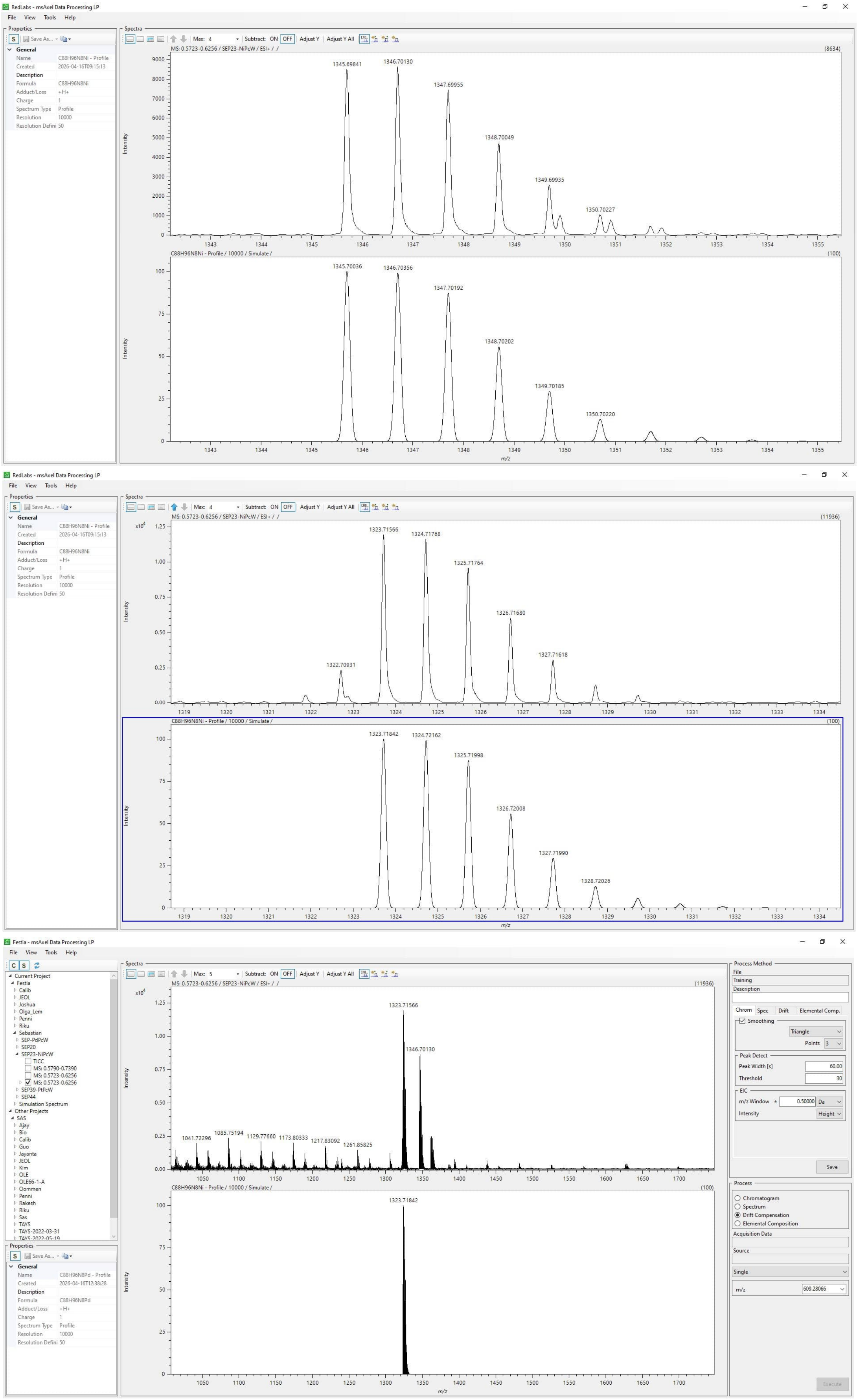

Figure S7: High-resolution ESI-TOF mass spectrum of **NiPc-Windmill** vs. the $[M+H]^+$ ($C_{88}H_{97}N_8Ni^+$) and $[M+Na]^+$ ($C_{88}H_{96}N_8NiNa^+$) isotope model.

UV–vis absorption spectra

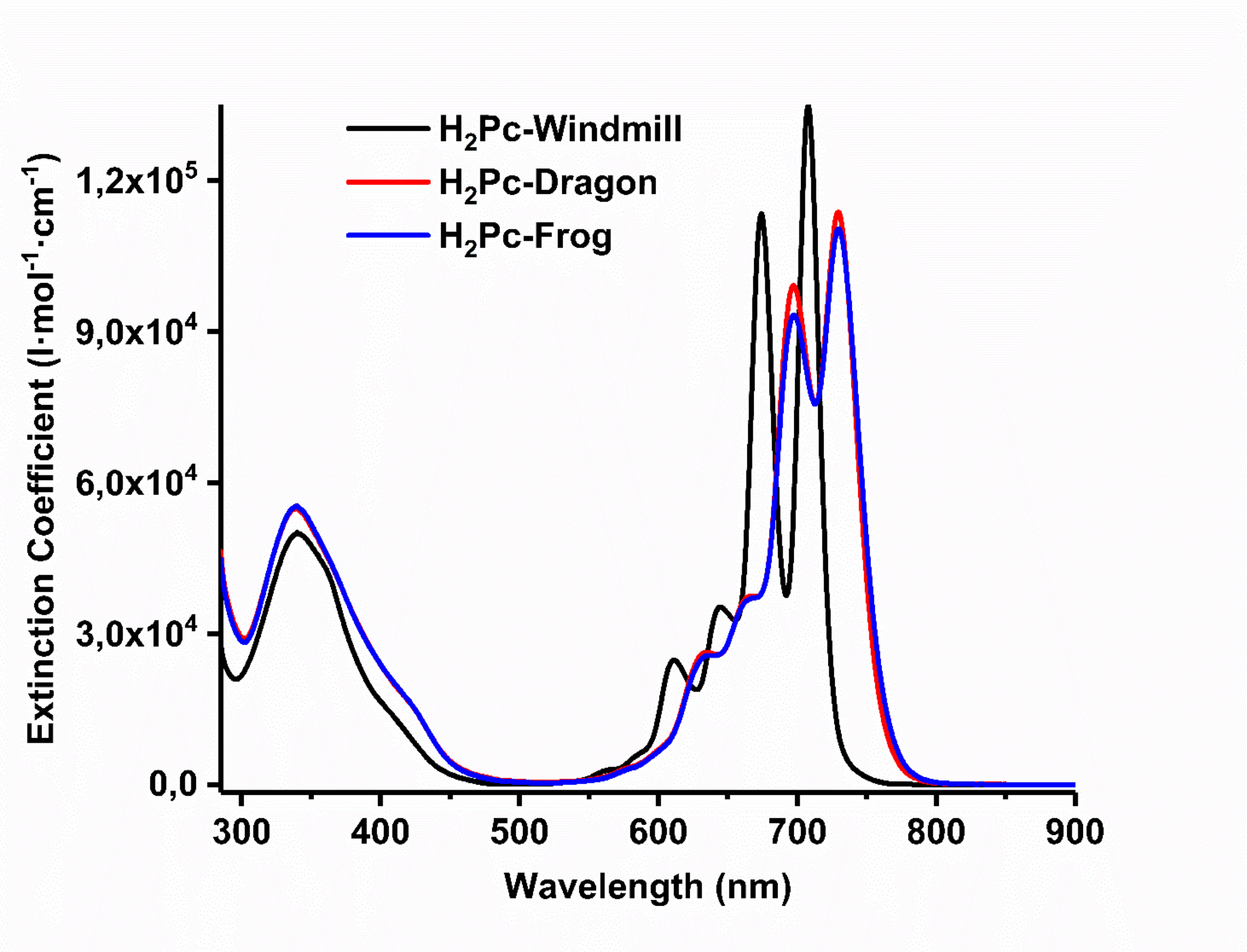

Figure S8: UV–vis absorption spectra of the individual regioisomeric **H_2Pcs** in toluene.

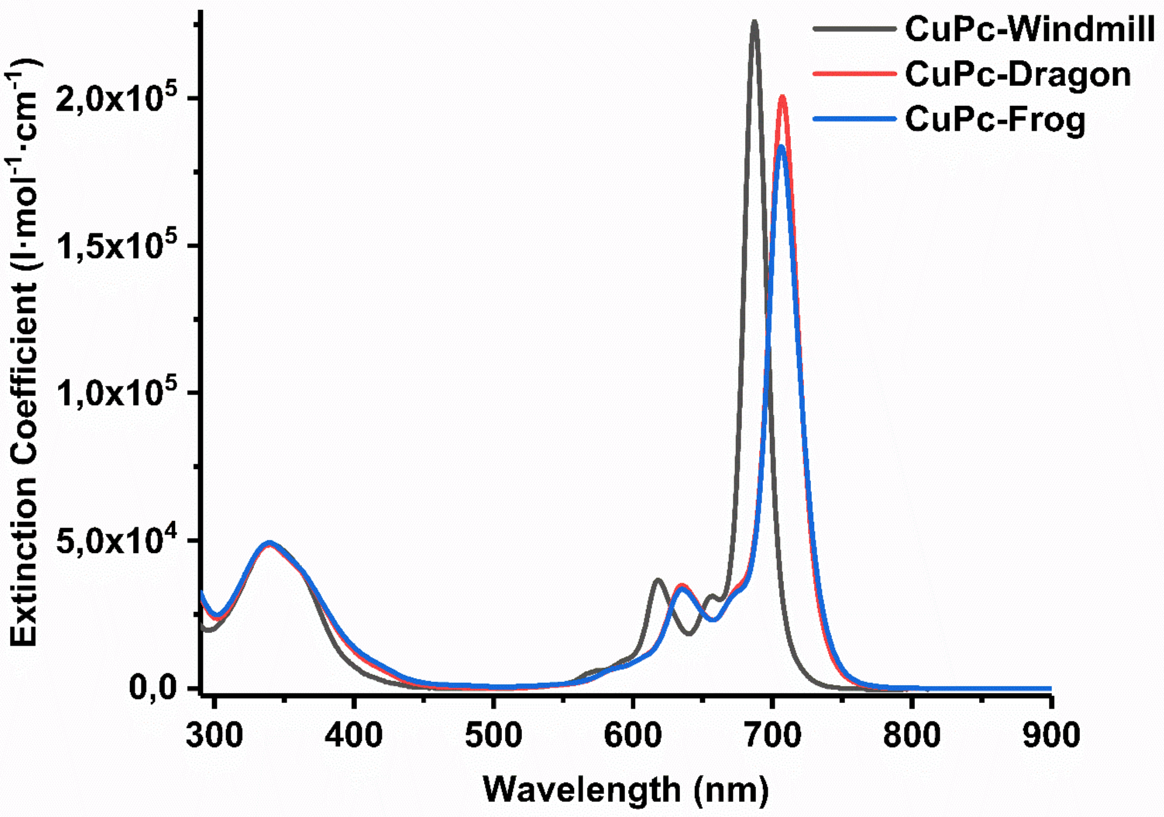

Figure S9: UV–vis absorption spectra of the individual regioisomeric **CuPcs** in toluene.

NMR spectra

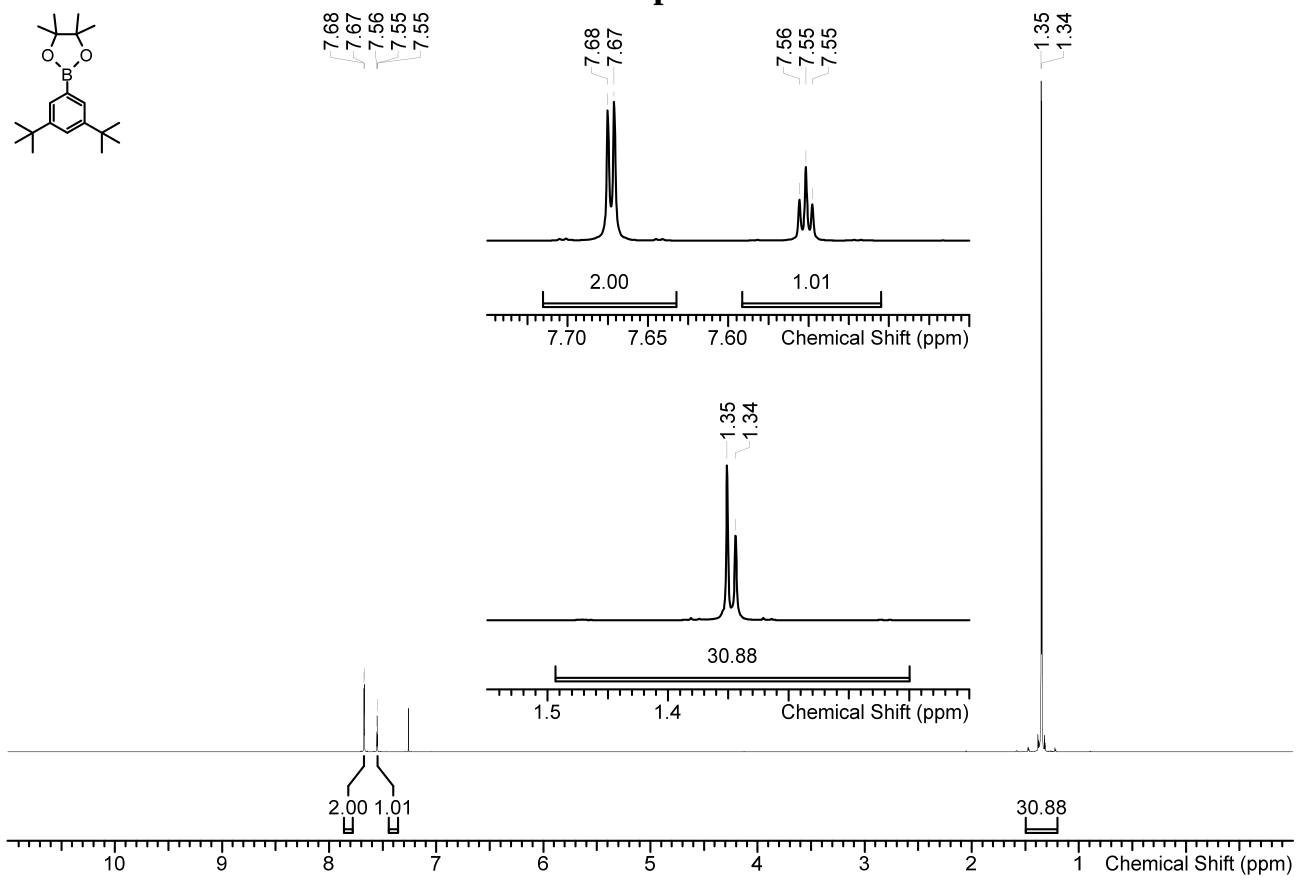

Figure S10: ^{1}H NMR spectrum (500 MHz, $CDCl_3$) of **1.**

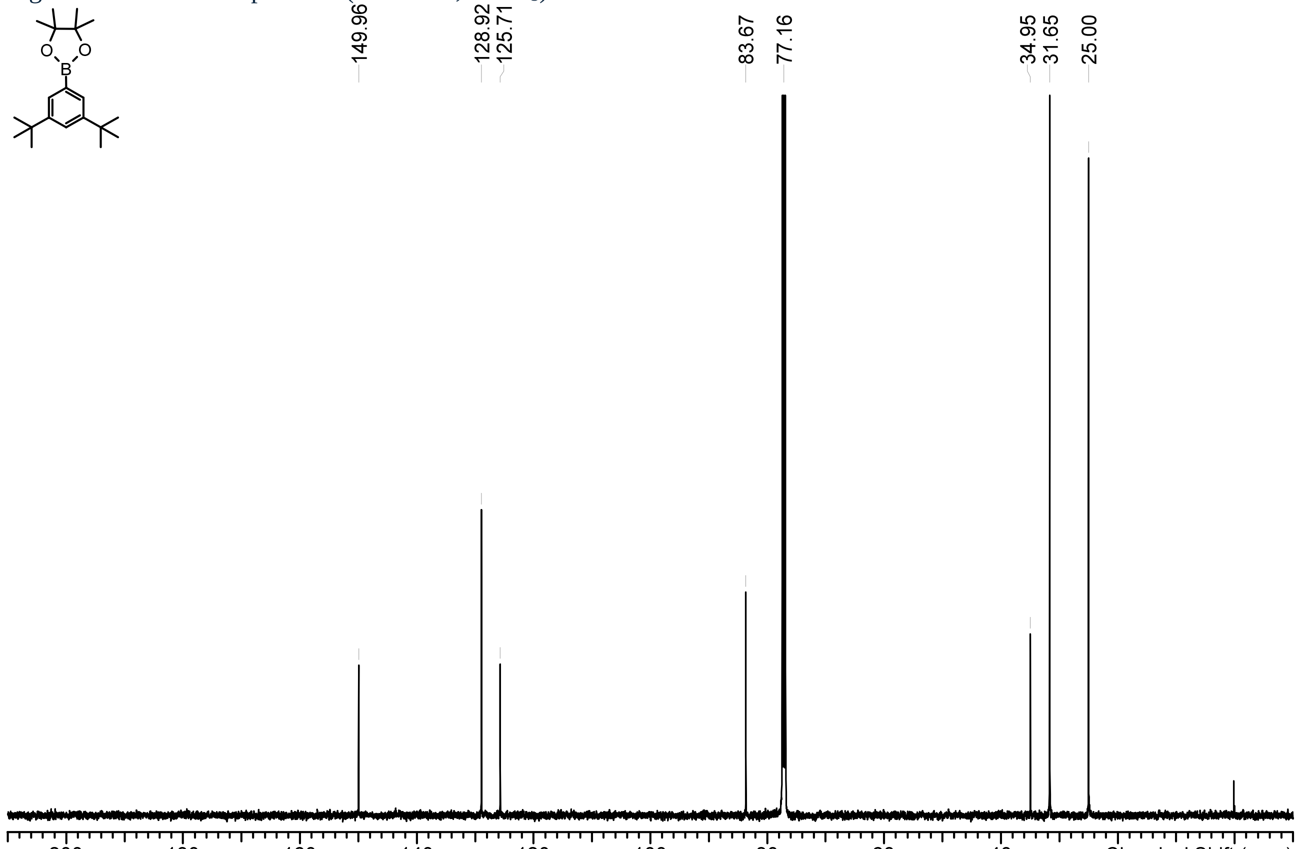

Figure S11: $^{13}C\{^{1}H\}$ NMR spectrum (126 MHz, $CDCl_3$) of **1**.

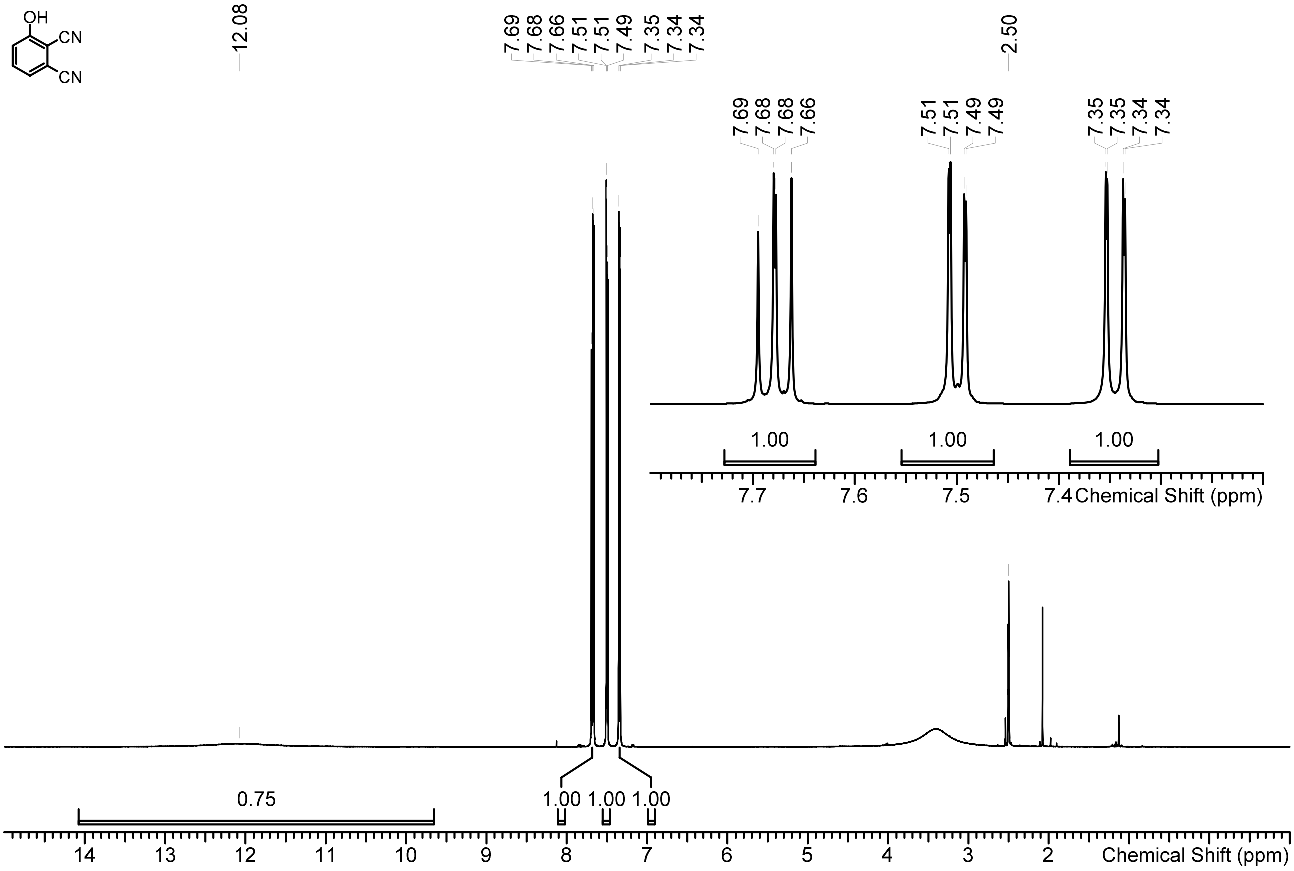

Figure S12: ^{1}H NMR spectrum (500 MHz, DMSO-d_6) of **2**.

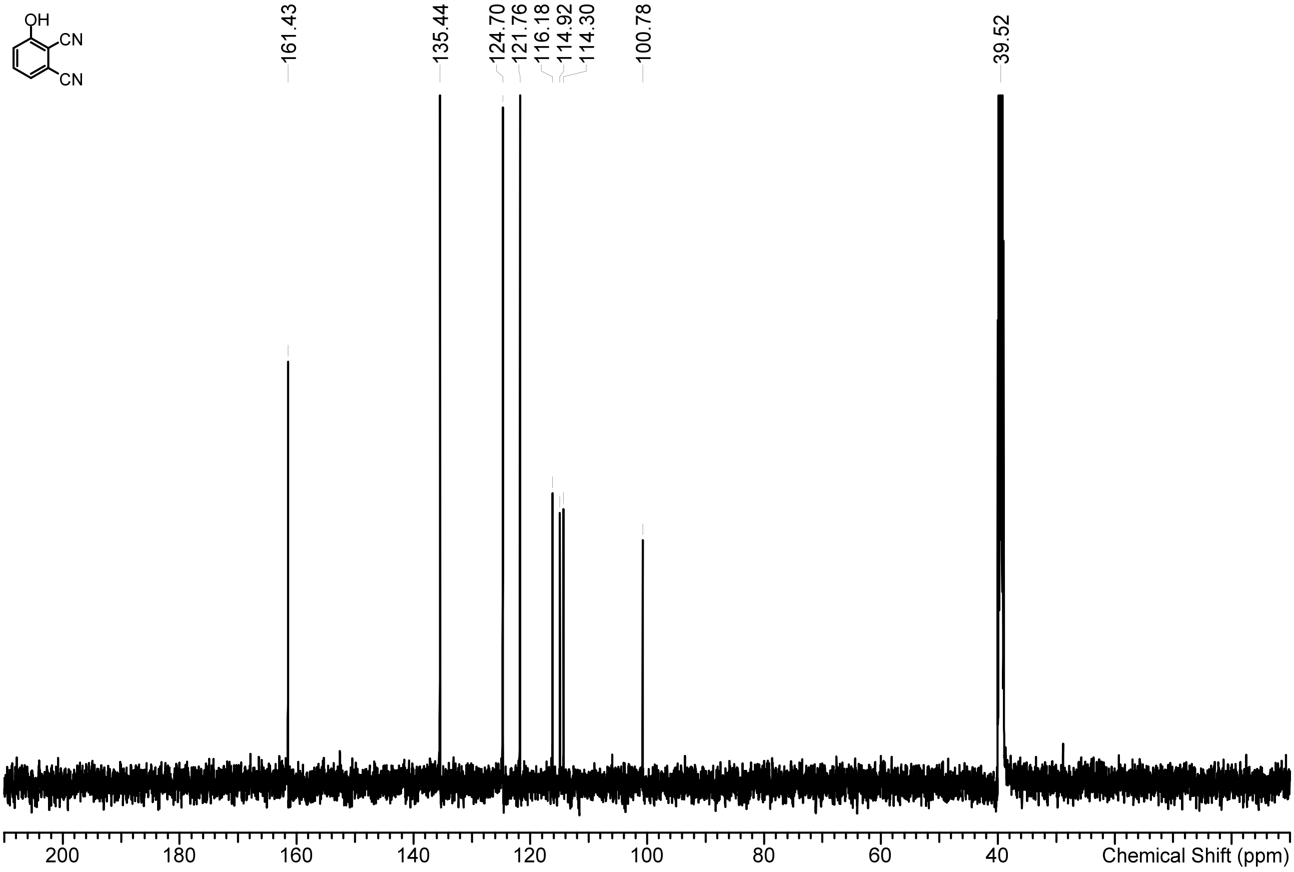

Figure S13: $^{13}C\{^{1}H\}$ NMR spectrum (126 MHz, DMSO-d_6) of **2**.

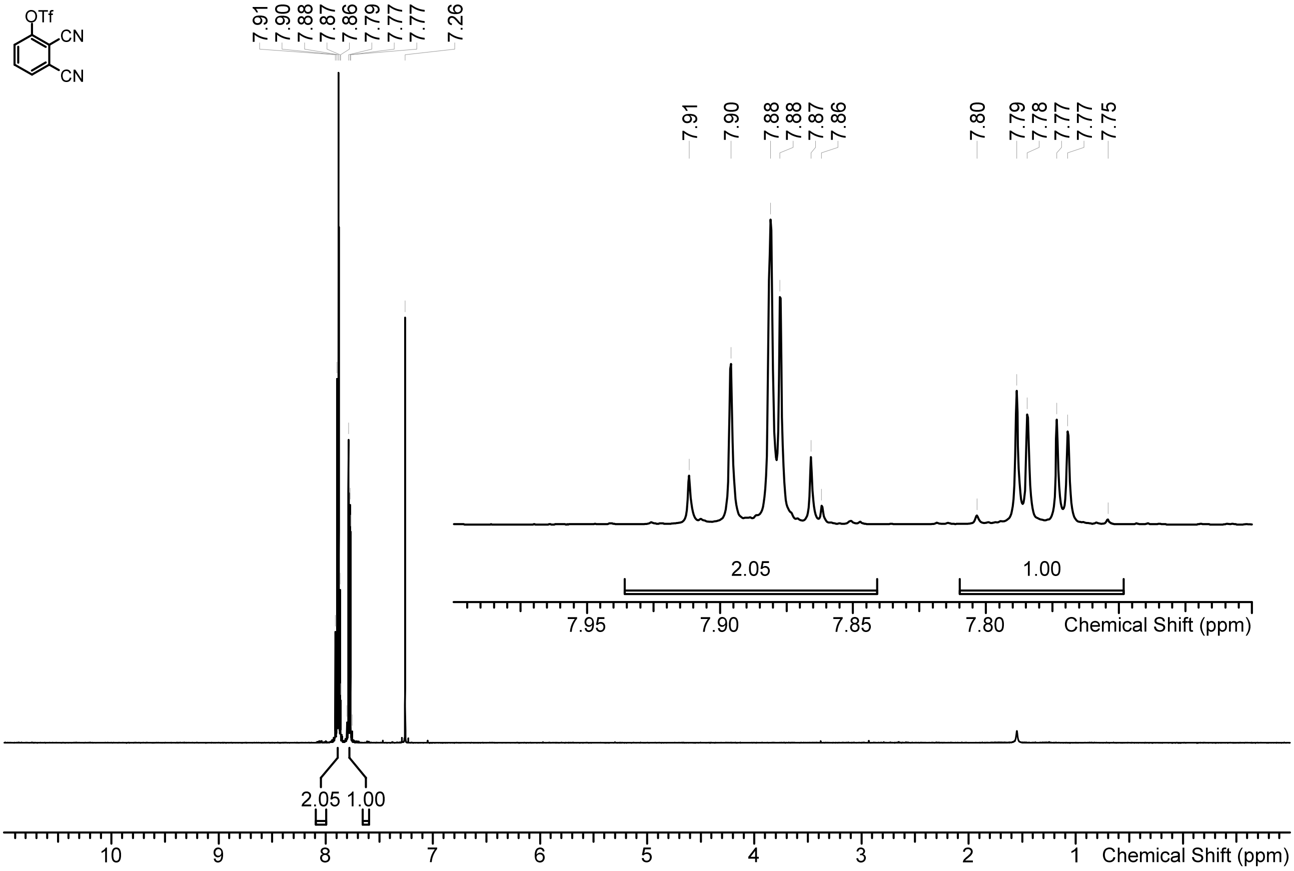

Figure S14: ^{1}H NMR spectrum (500 MHz, $CDCl_3$) of **3**.

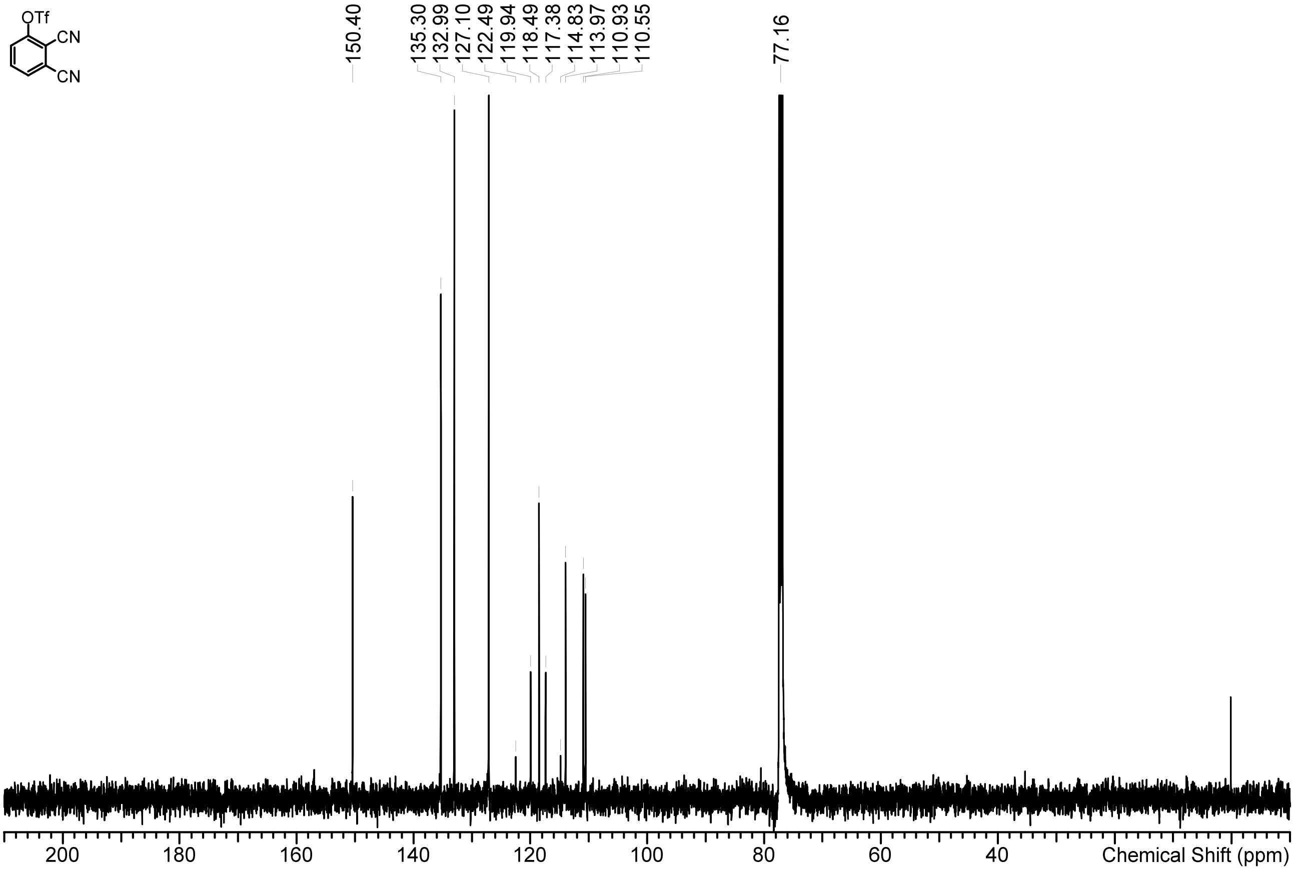

Figure S15: $^{13}C\{^{1}H\}$ NMR spectrum (126 MHz, $CDCl_3$) of **3**.

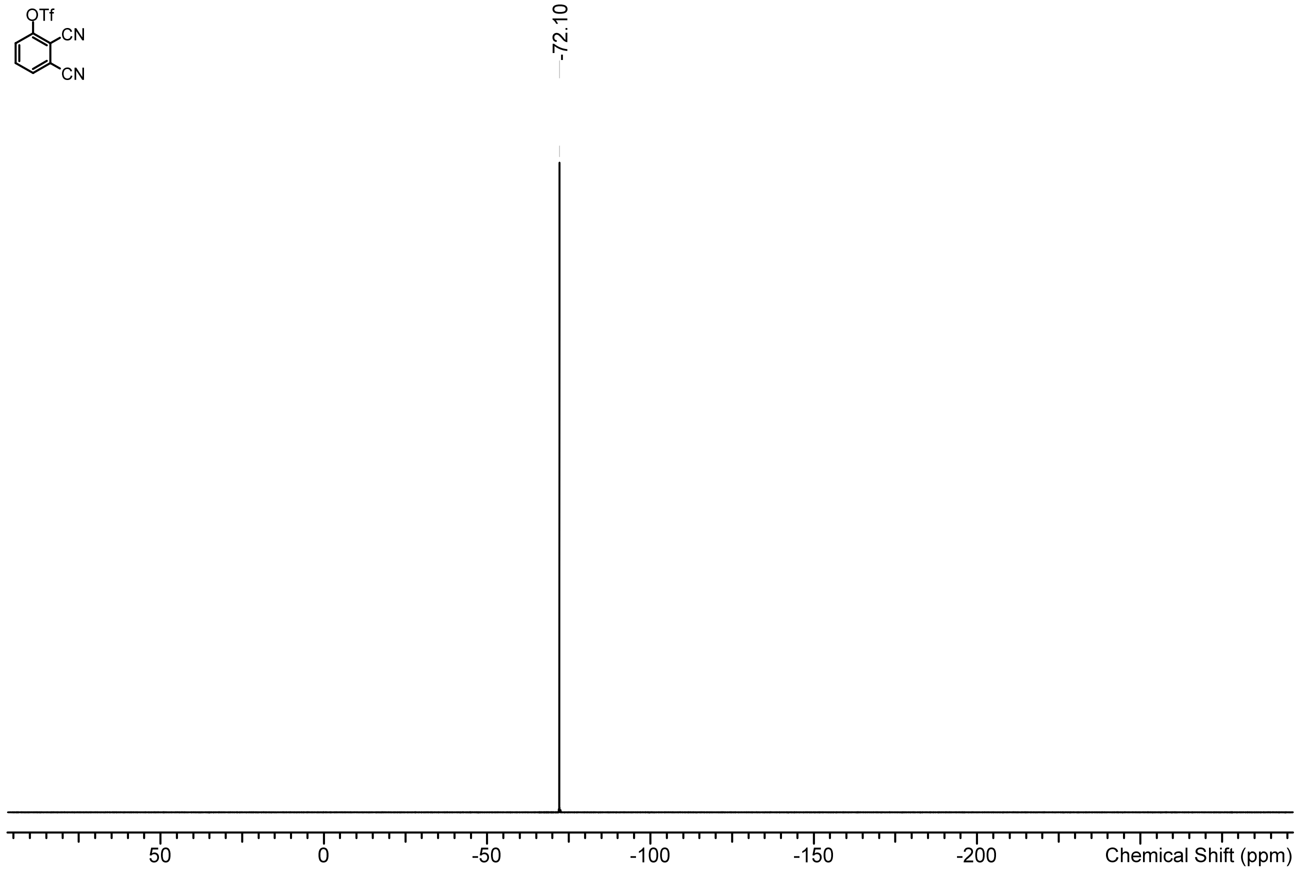

Figure S16: $^{19}F\{^{1}H\}$ NMR spectrum (471 MHz, $CDCl_3$) of **3**.

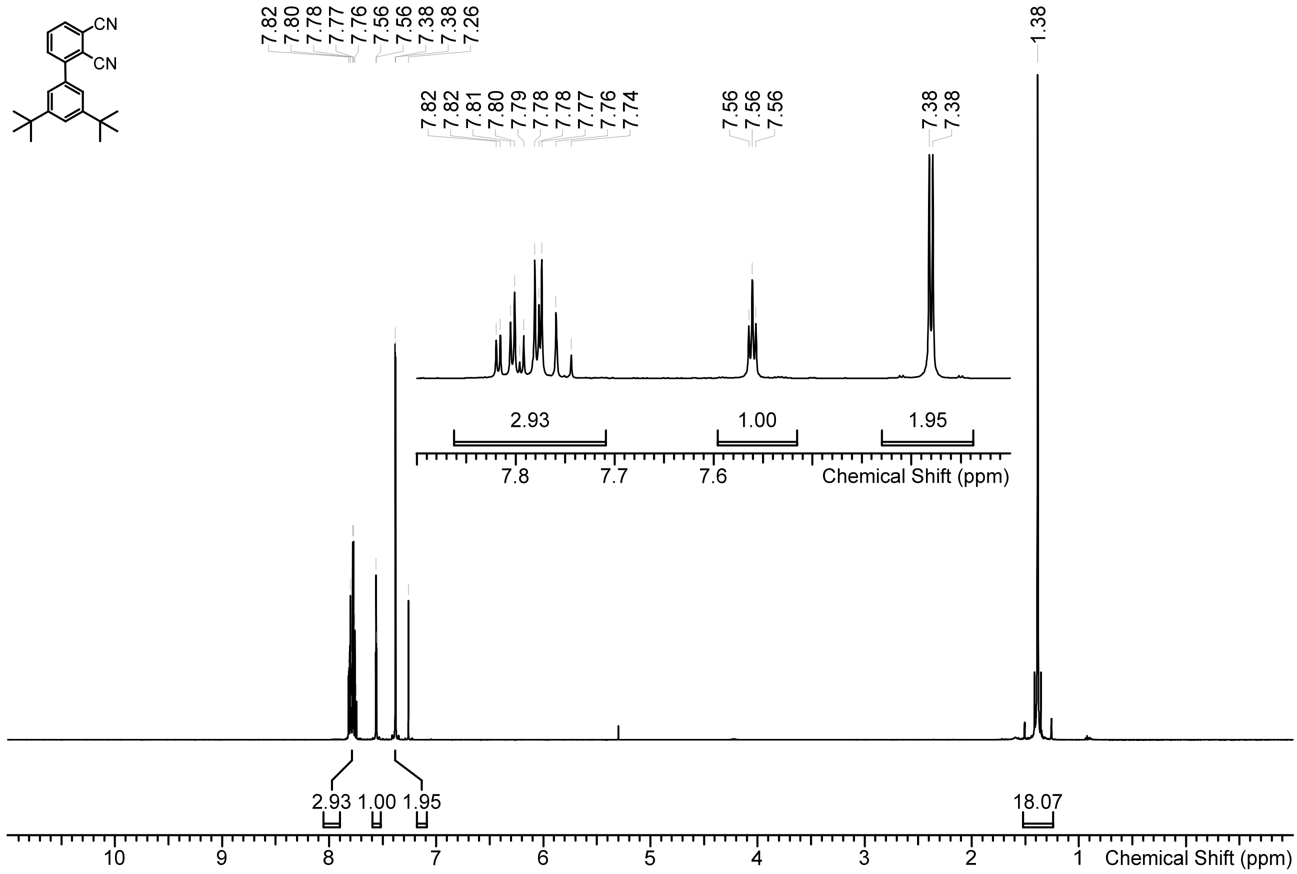

Figure S17: ^{1}H NMR spectrum (500 MHz, $CDCl_3$) of **4**.

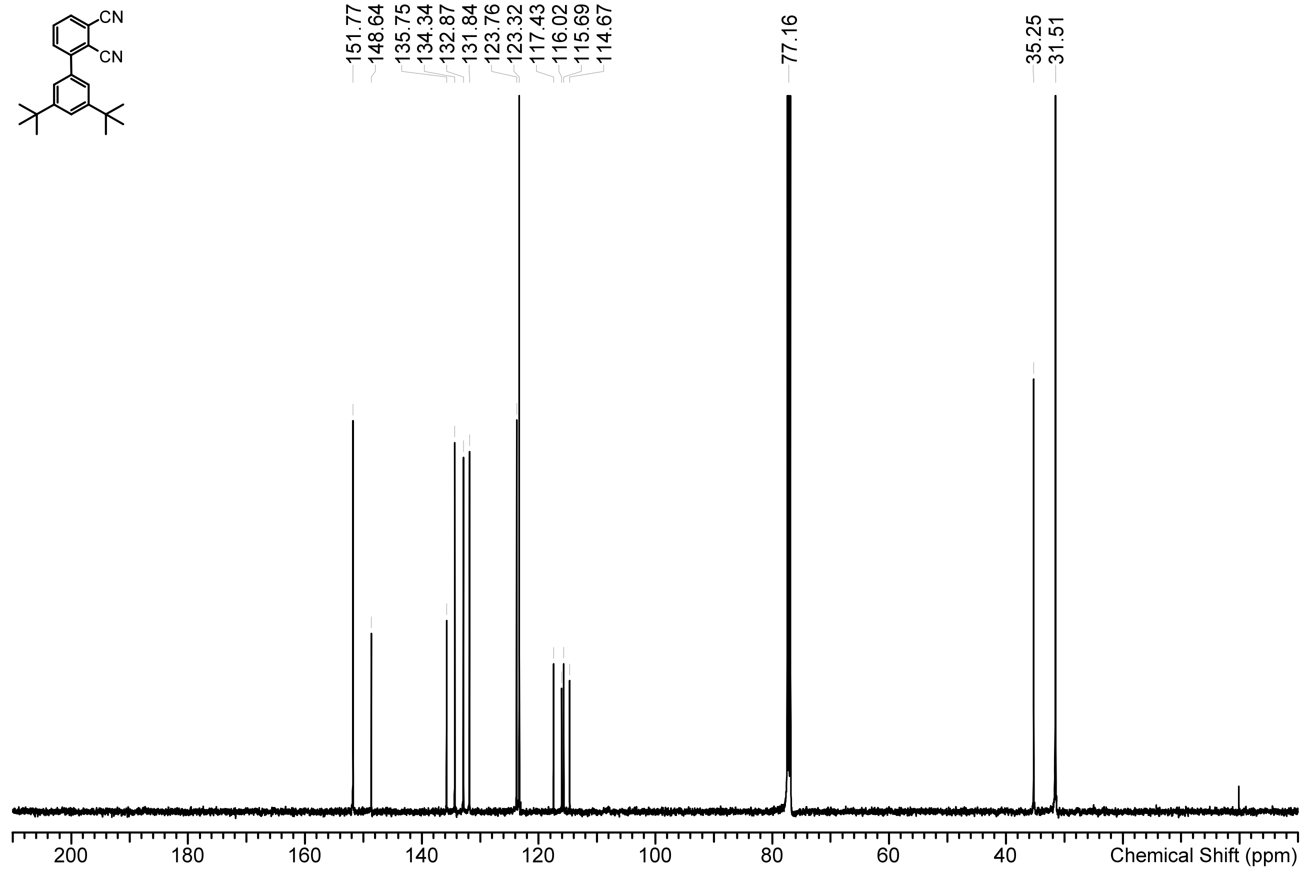

Figure S18: $^{13}C\{^{1}H\}$ NMR spectrum (126 MHz, $CDCl_3$) of **4**.

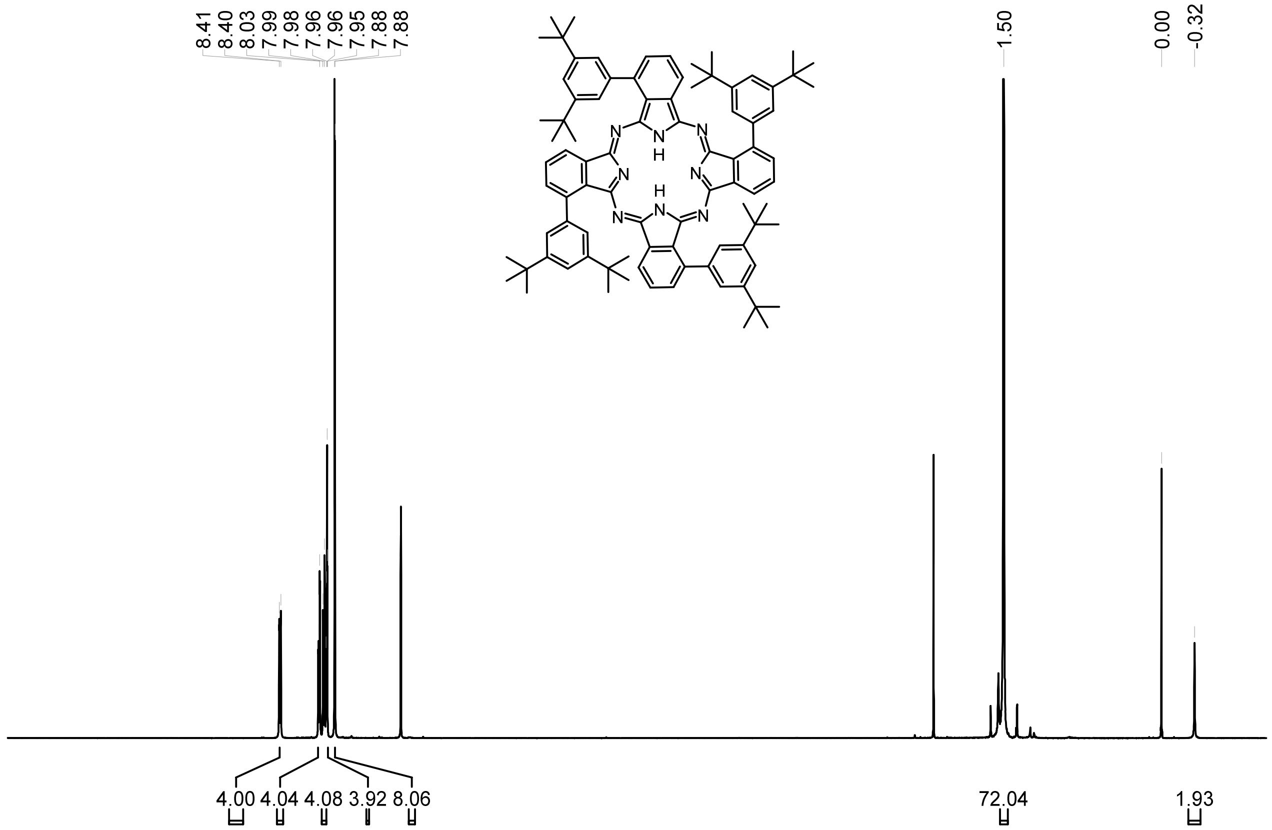

Figure S19: ^{1}H NMR spectrum (500 MHz, $CDCl_3$) of **H_2Pc-Windmill**.

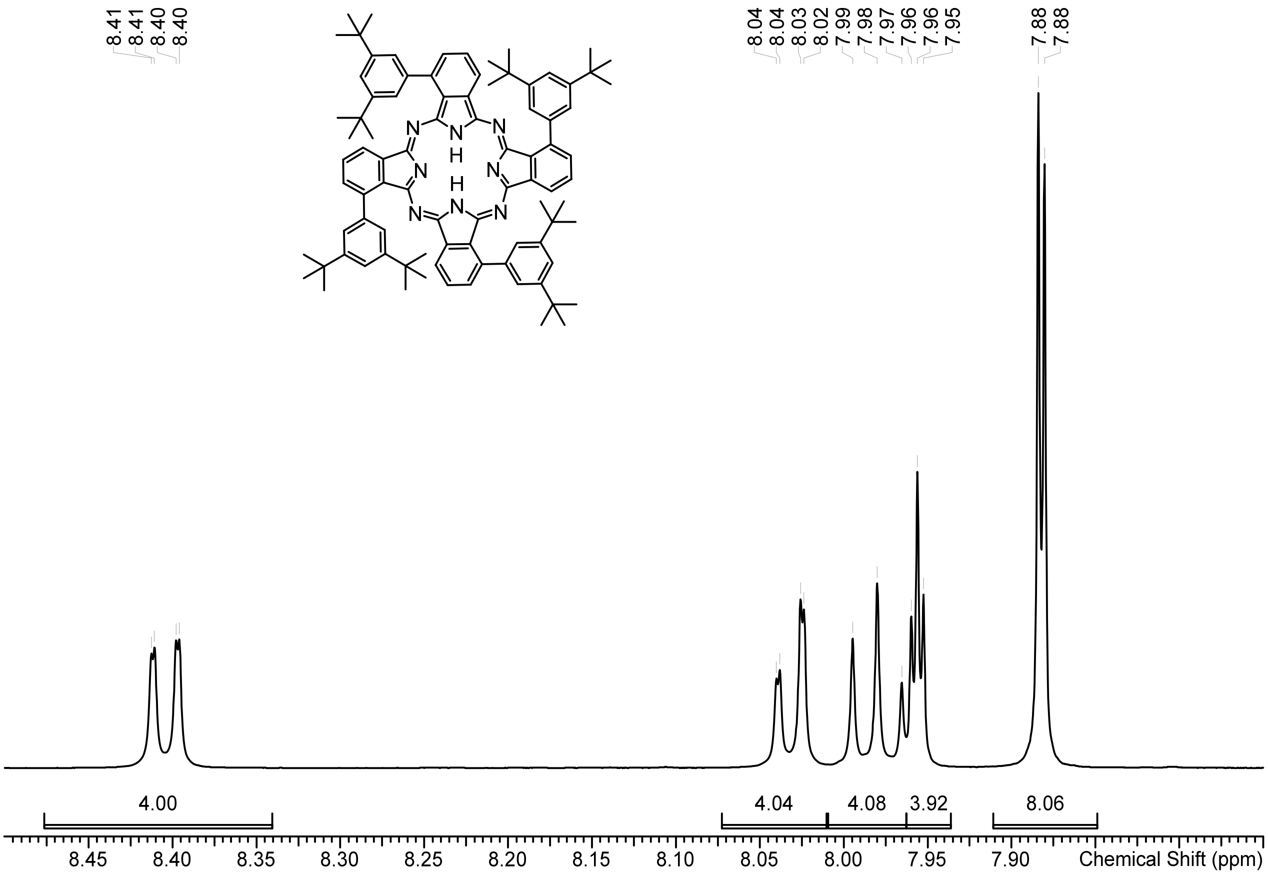

Figure S20: Aromatic region of the ^{1}H NMR spectrum (500 MHz, $CDCl_3$) of **H_2Pc-Windmill**.

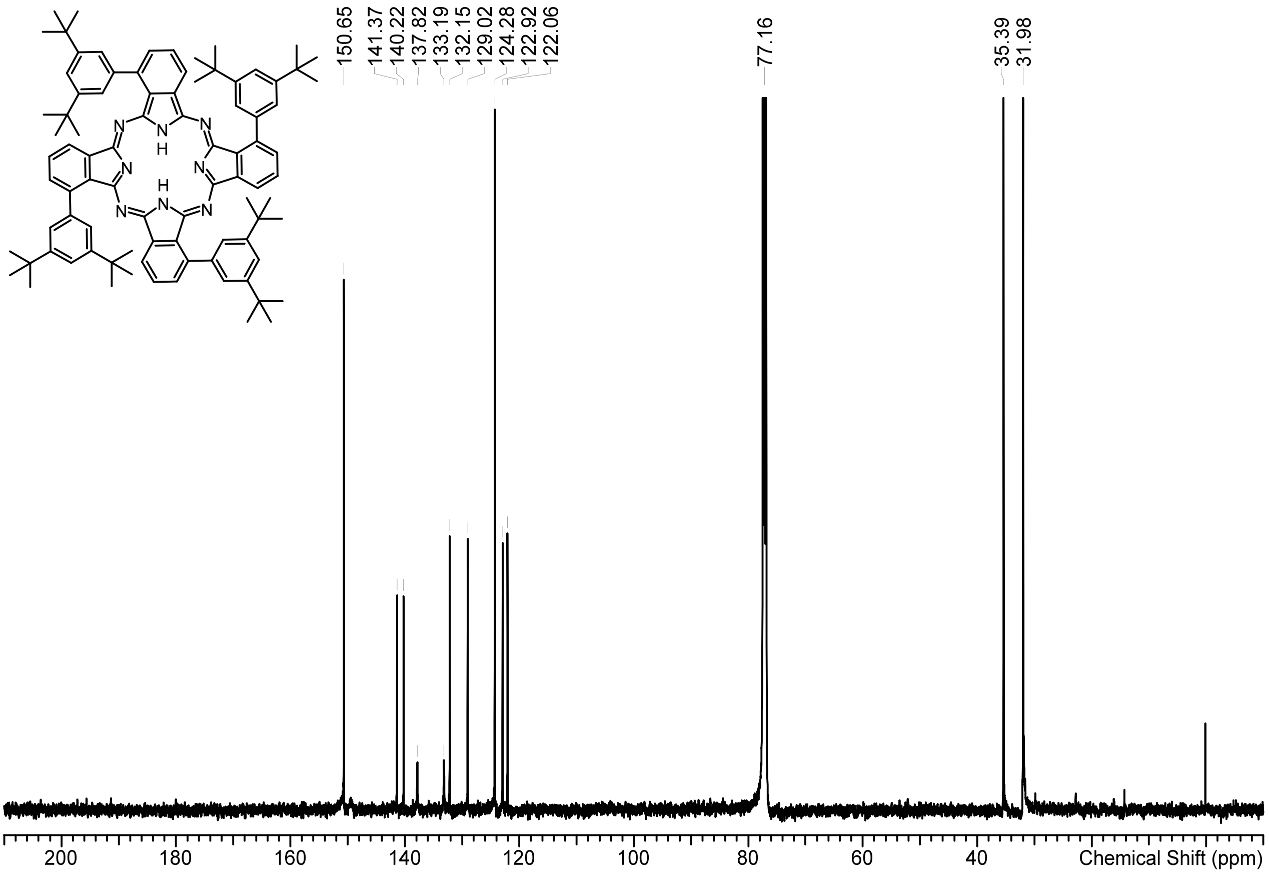

Figure S21: $^{13}C\{^{1}H\}$ NMR spectrum (126 MHz, $CDCl_3$) of **H_2Pc-Windmill**.

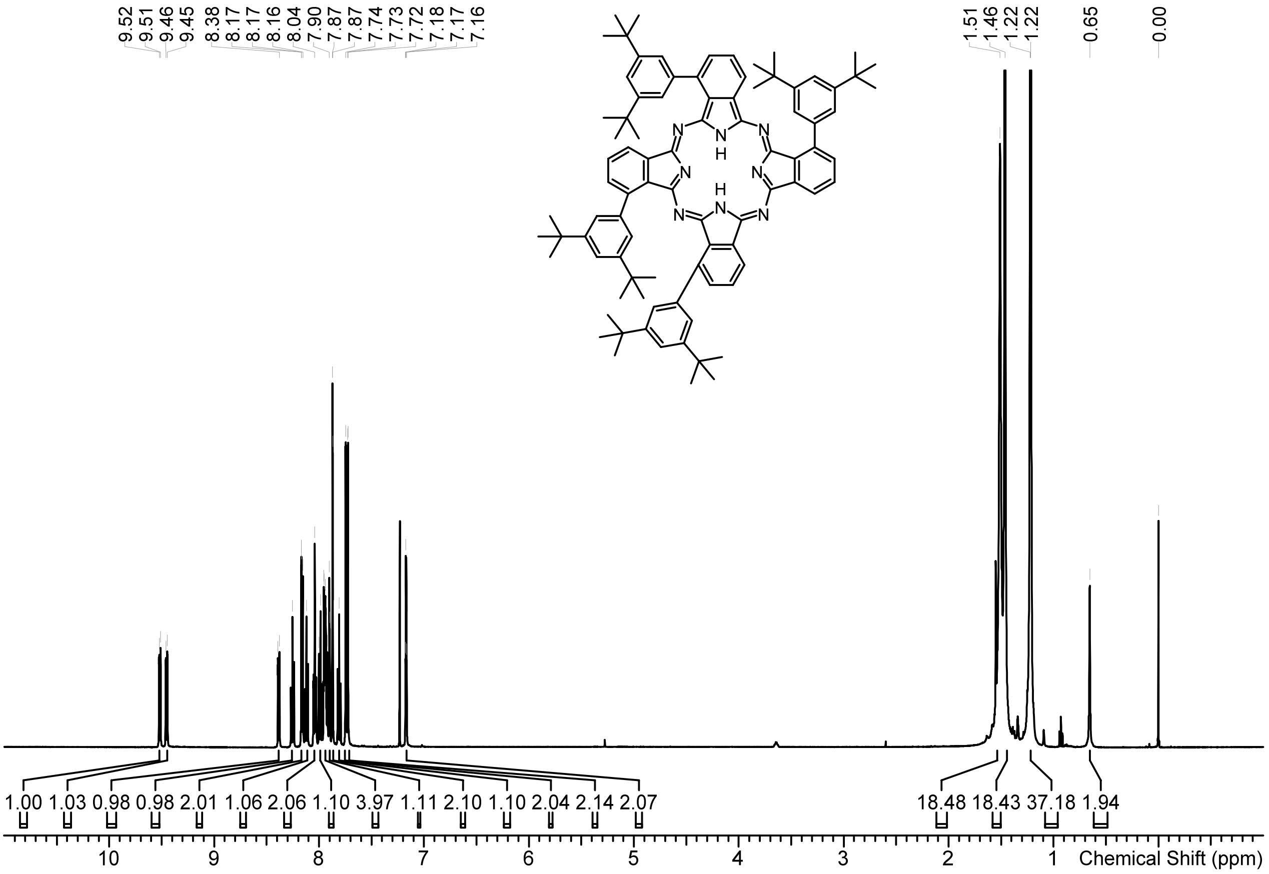

Figure S22: ^{1}H NMR spectrum (500 MHz, $CDCl_3$) of **H_2Pc-Dragon**.

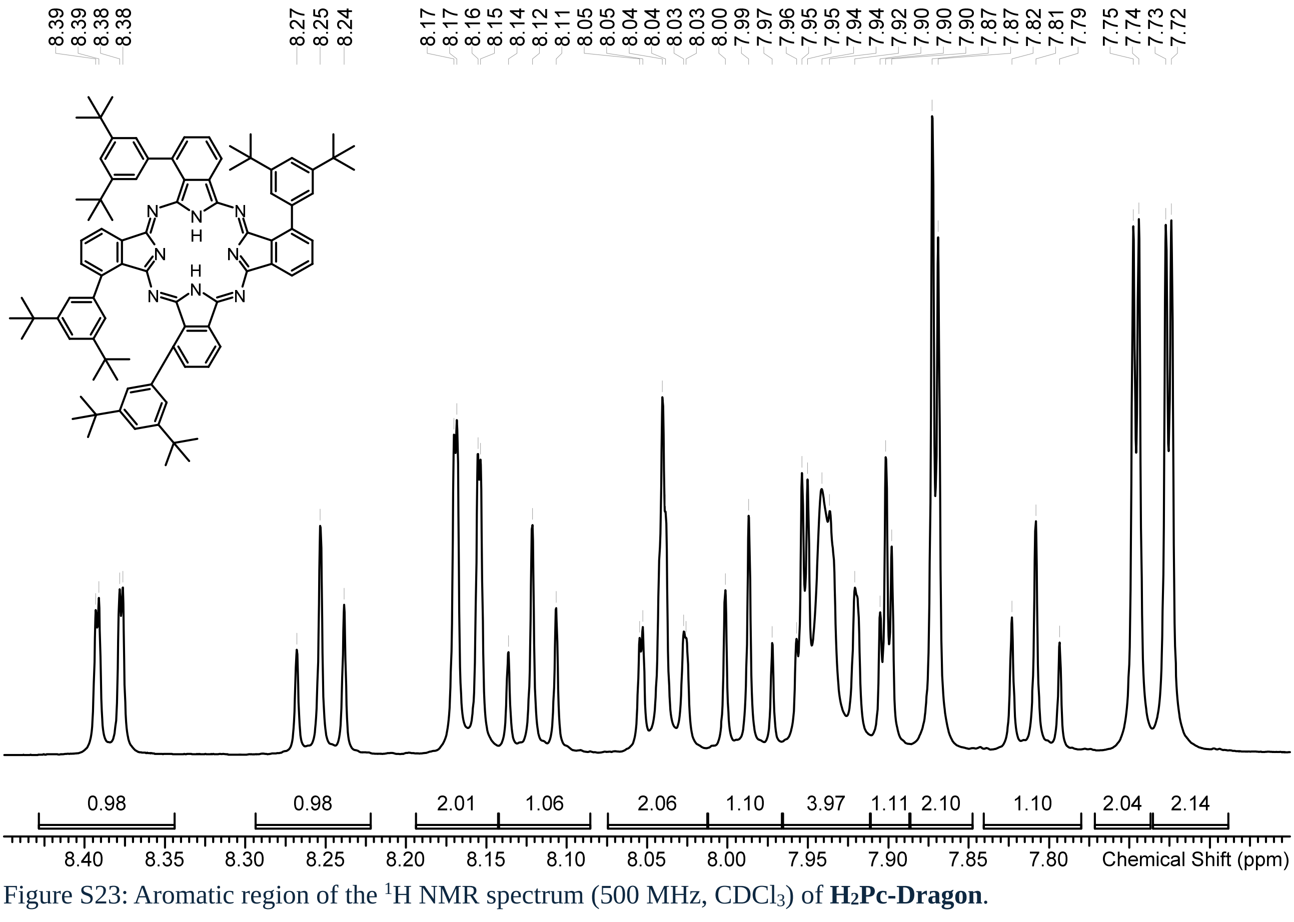

Figure S23: Aromatic region of the ^{1}H NMR spectrum (500 MHz, $CDCl_3$) of **H_2Pc-Dragon**.

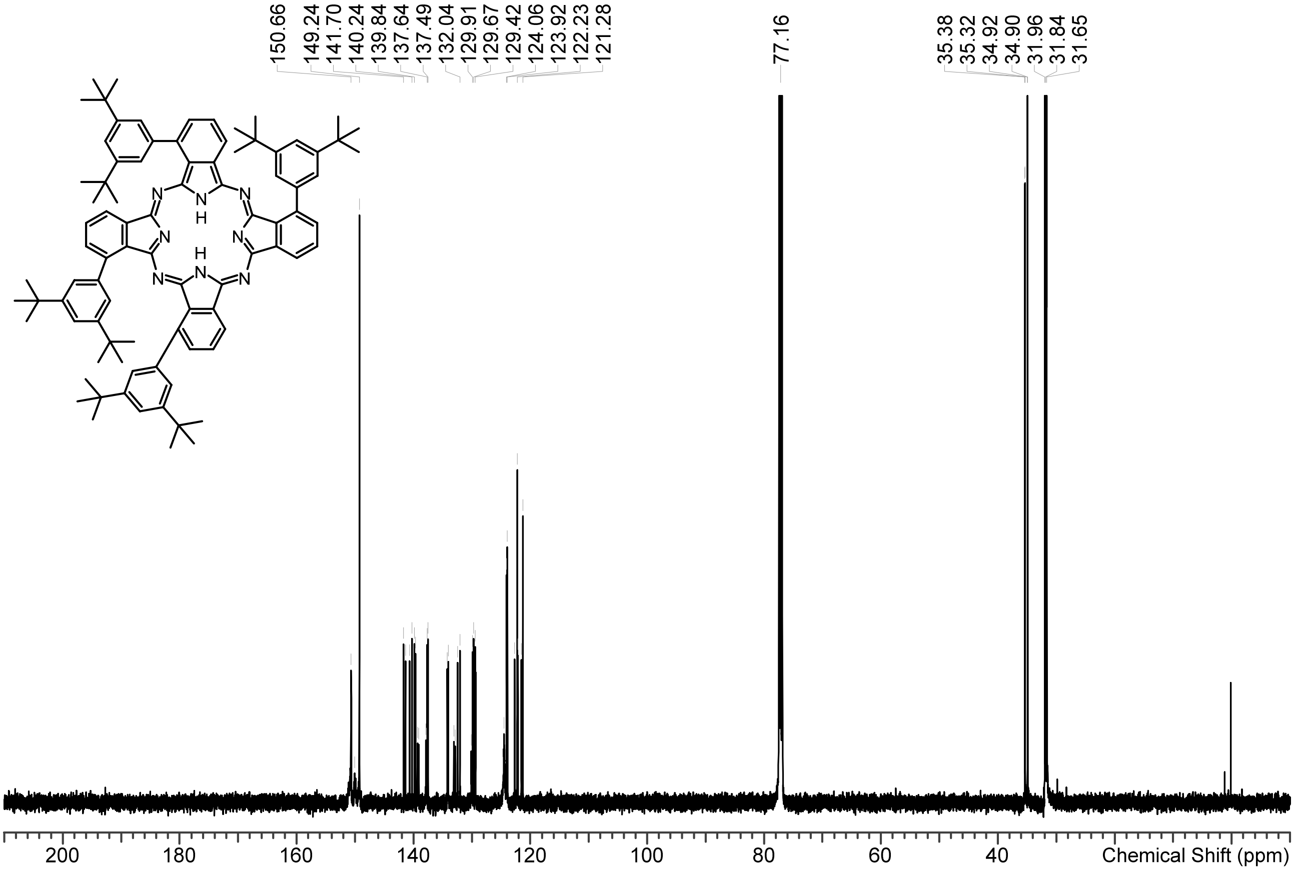

Figure S24: $^{13}C\{^{1}H\}$ NMR spectrum (126 MHz, $CDCl_3$) of **H_2Pc-Dragon**.

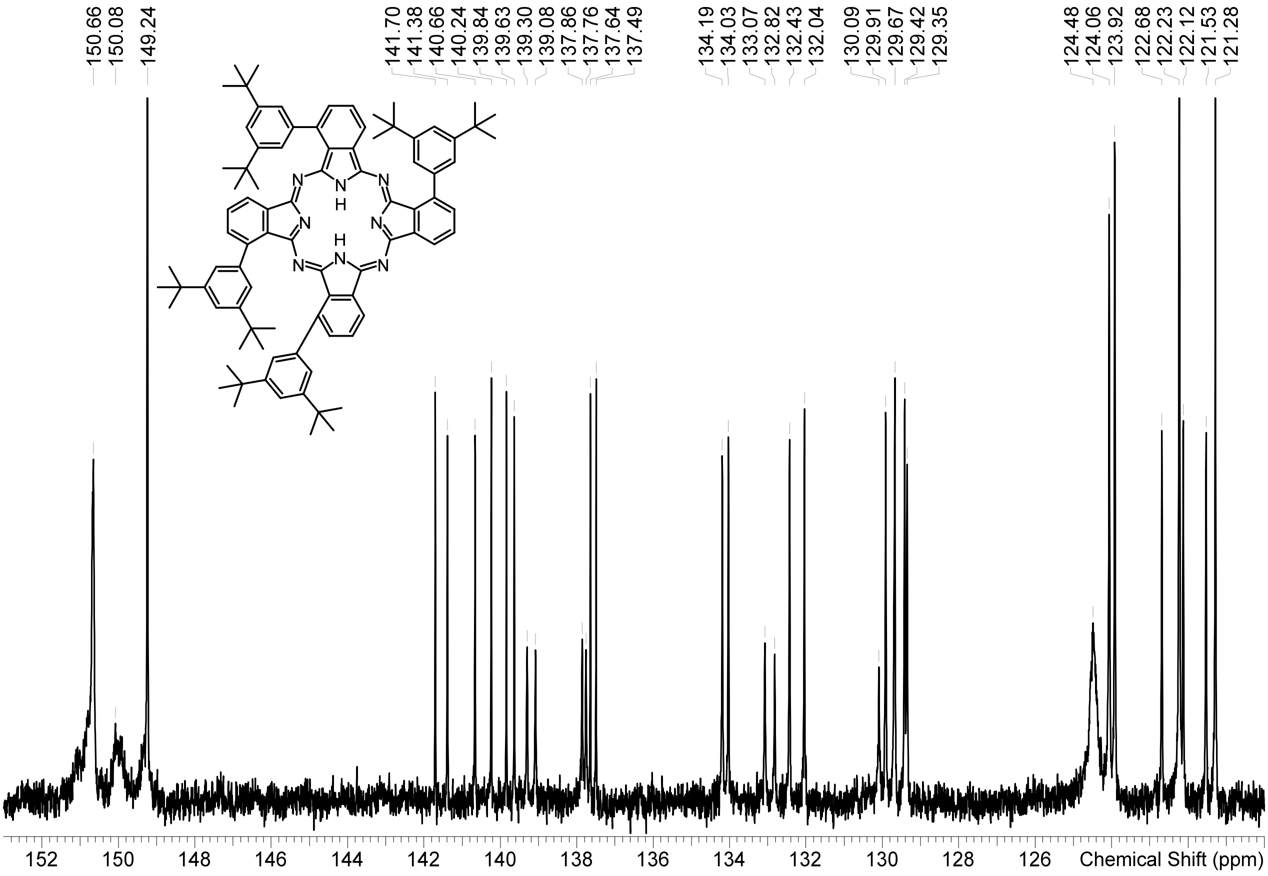

Figure S25: Aromatic region of the $^{13}C\{^{1}H\}$ NMR spectrum (126 MHz, $CDCl_3$) of **H_2Pc-Dragon**.

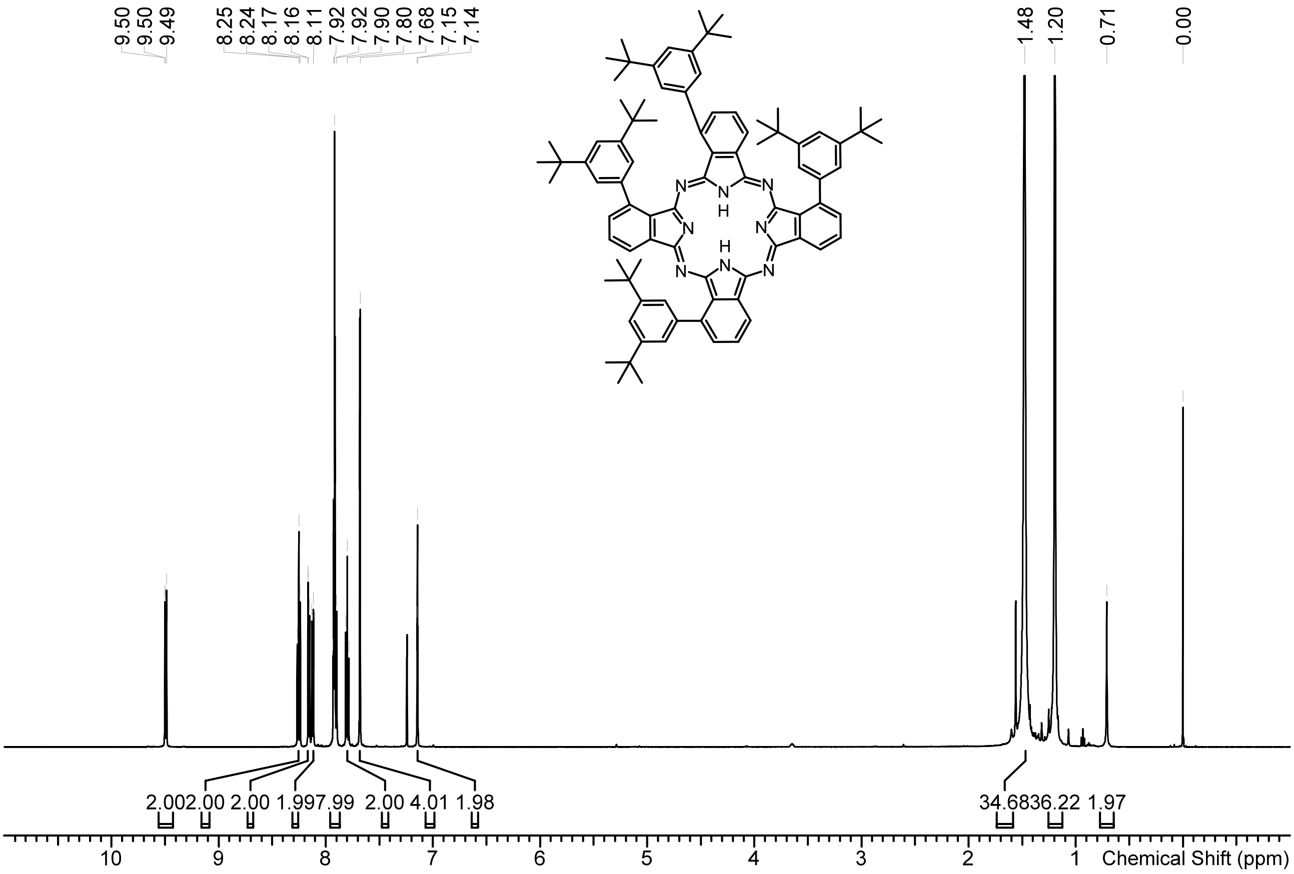

Figure S26: ^{1}H NMR spectrum (500 MHz, $CDCl_3$) of **H_2Pc-Frog**.

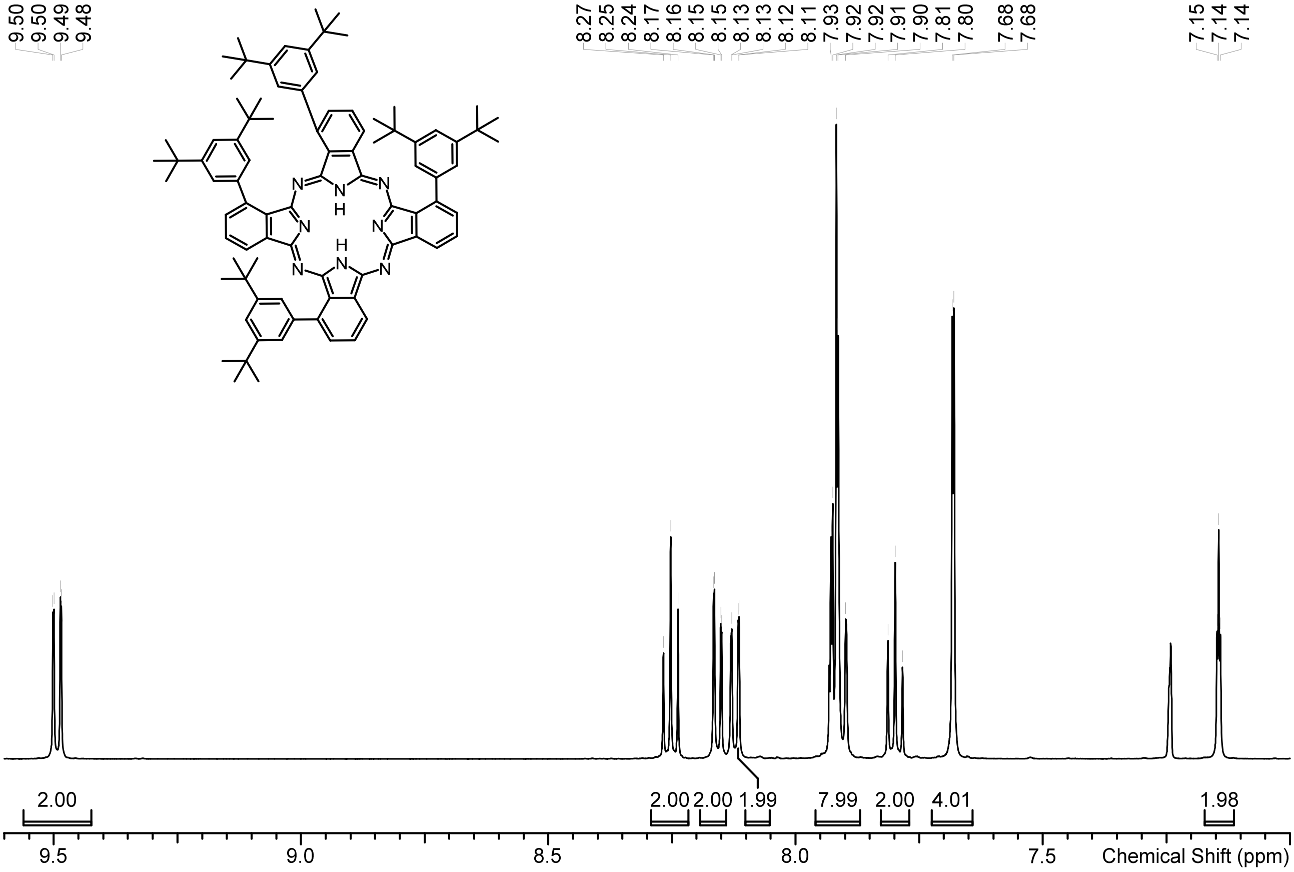

Figure S27: Aromatic region of the ^{1}H NMR spectrum (500 MHz, $CDCl_3$) of **H_2Pc-Frog**.

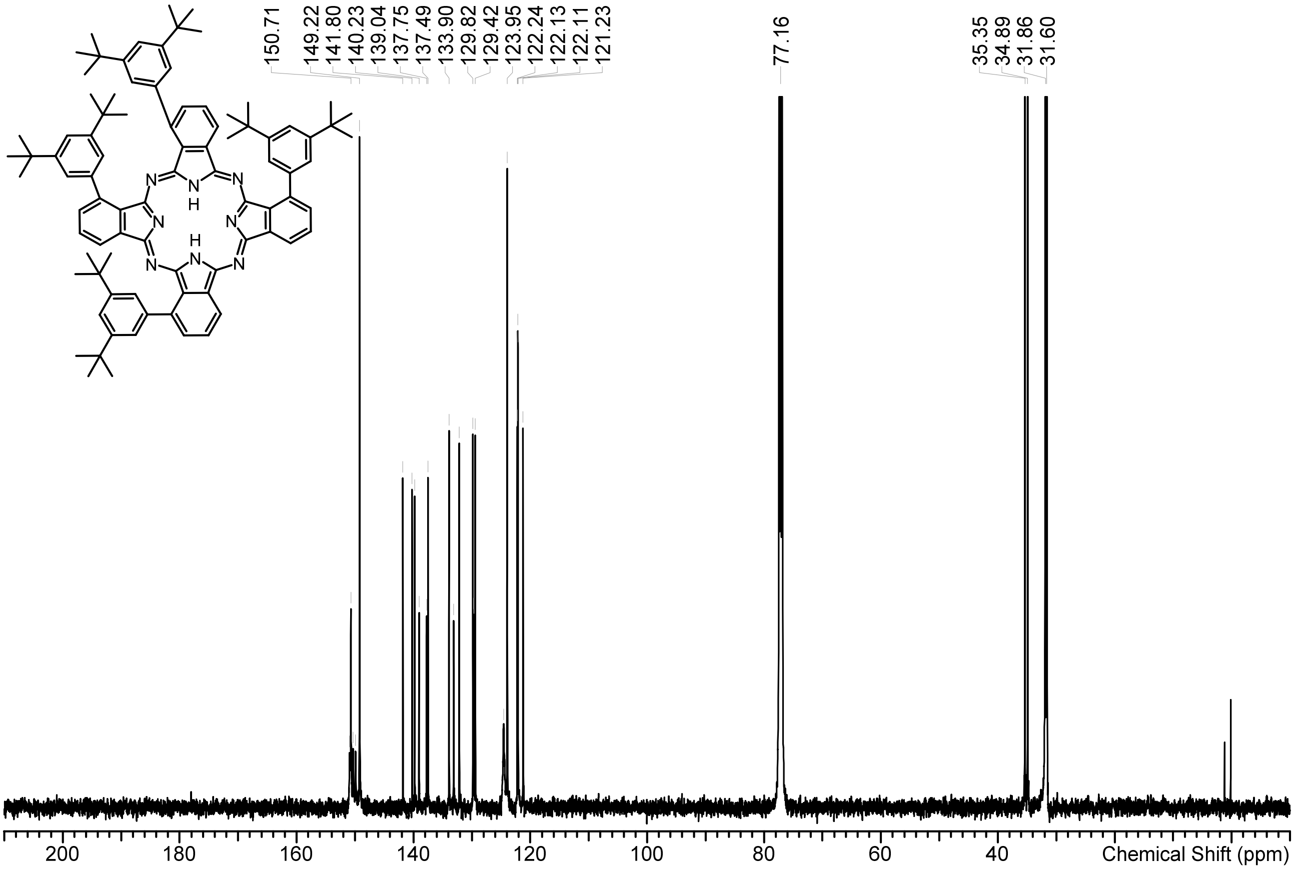

Figure S28: $^{13}C\{^{1}H\}$ NMR spectrum (126 MHz, $CDCl_3$) of **H_2Pc-Frog**.

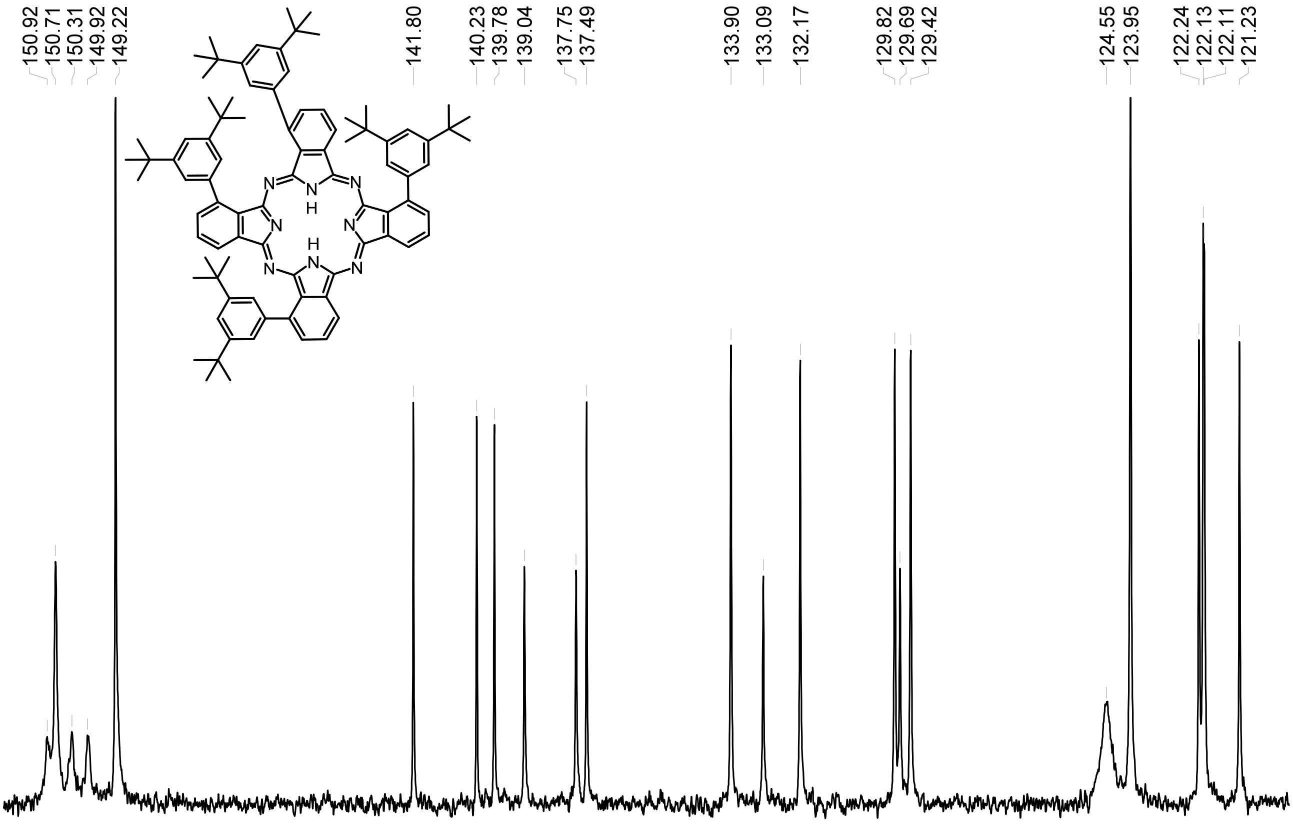

Figure S29: Aromatic region of the $^{13}C\{^{1}H\}$ NMR spectrum (126 MHz, $CDCl_3$) of **H_2Pc-Frog**.

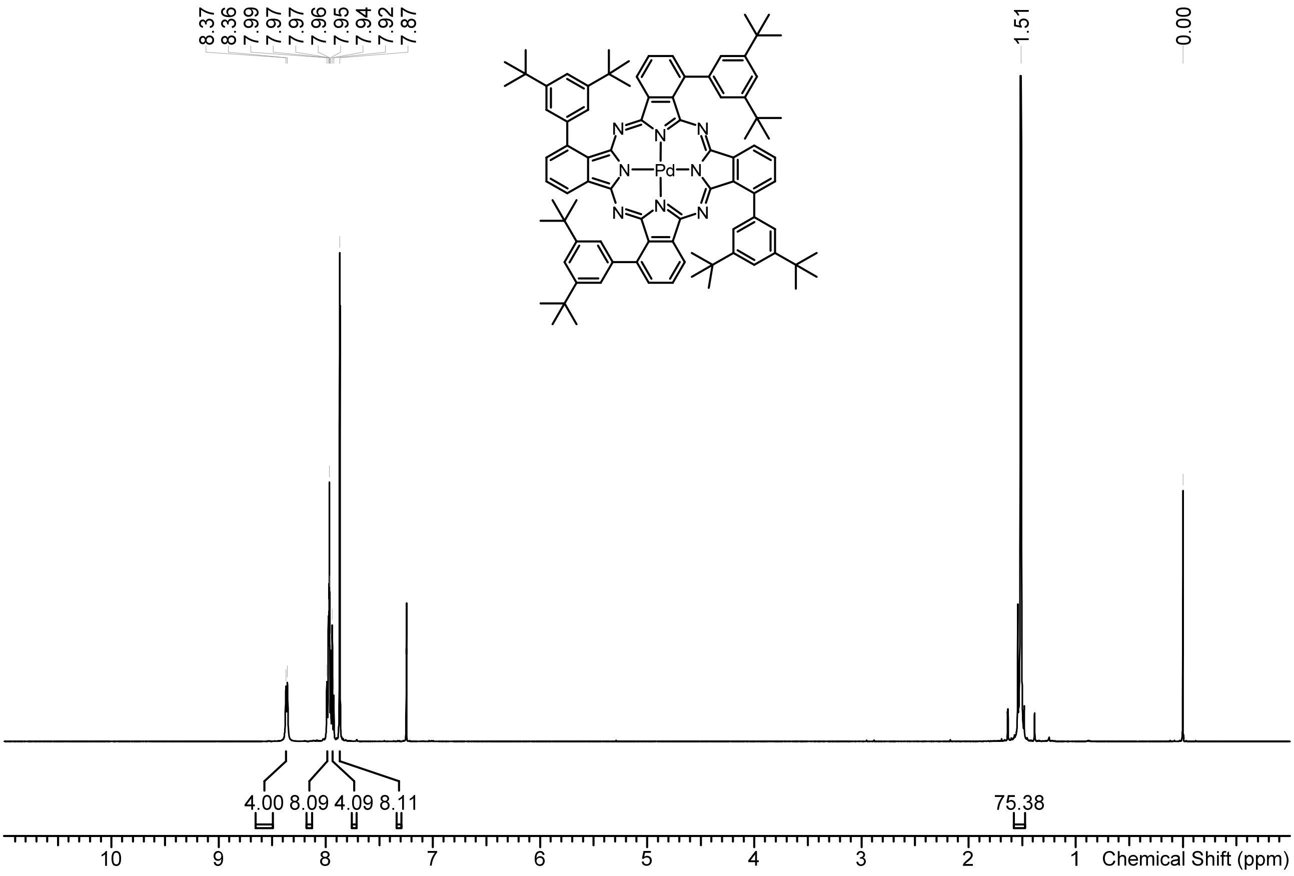

Figure S30: ^{1}H NMR spectrum (500 MHz, $CDCl_3$) of **PdPc-Windmill**.

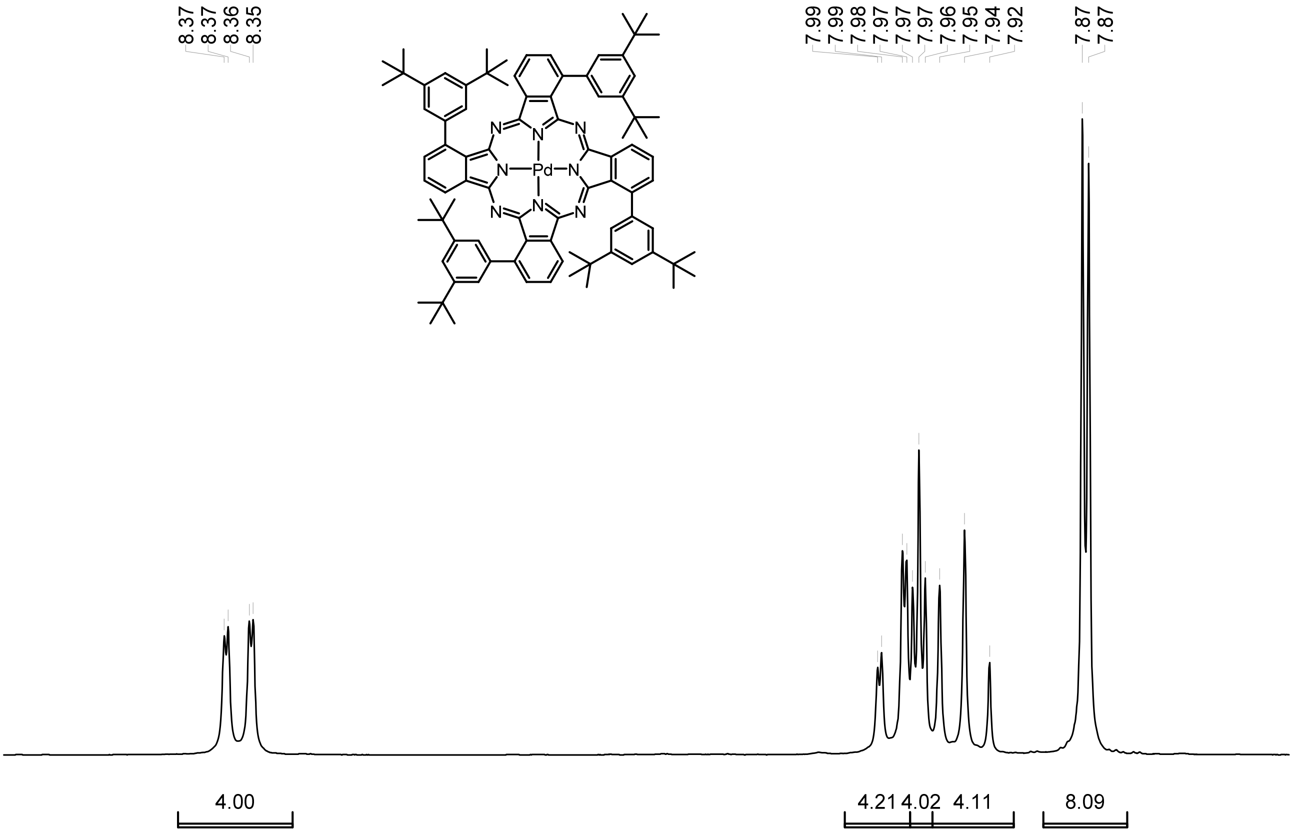

Figure S31: Aromatic region of the ^{1}H NMR spectrum (500 MHz, $CDCl_3$) of **PdPc-Windmill**.

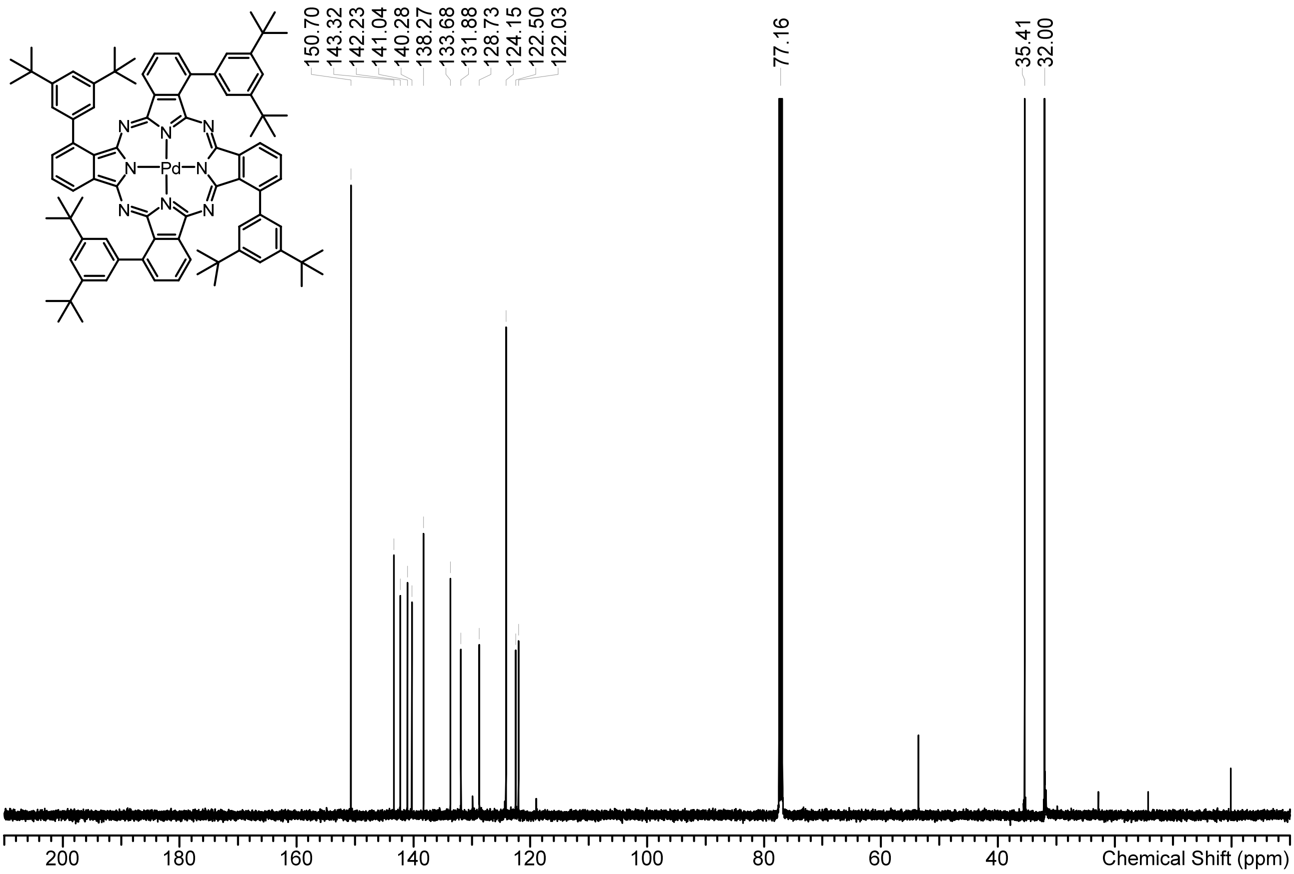

Figure S32: $^{13}C\{^1H\}$ NMR spectrum (126 MHz, $CDCl_3$) of **PdPc-Windmill**.

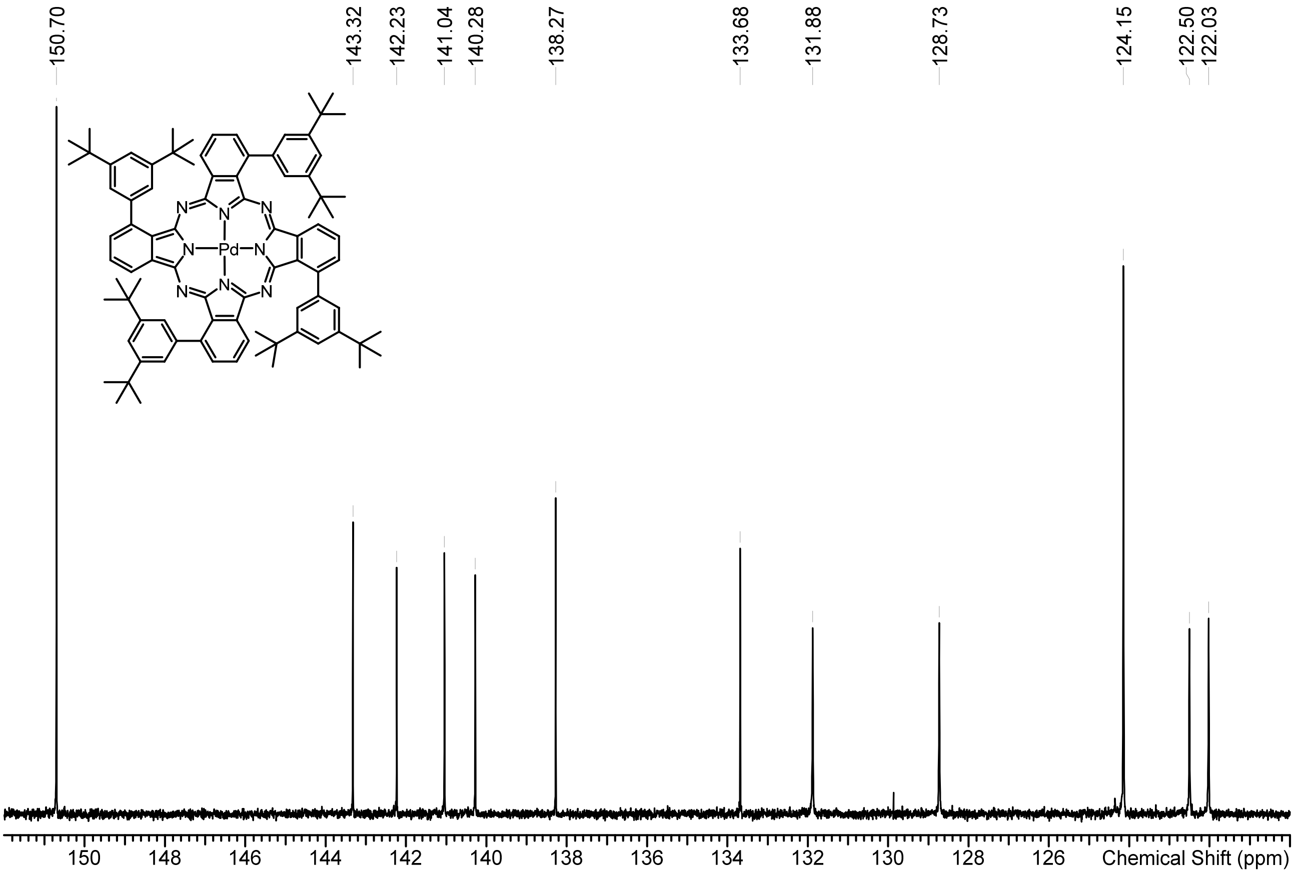

Figure S33: Aromatic region of the $^{13}C\{^1H\}$ NMR spectrum (126 MHz, $CDCl_3$) of **PdPc-Windmill**.

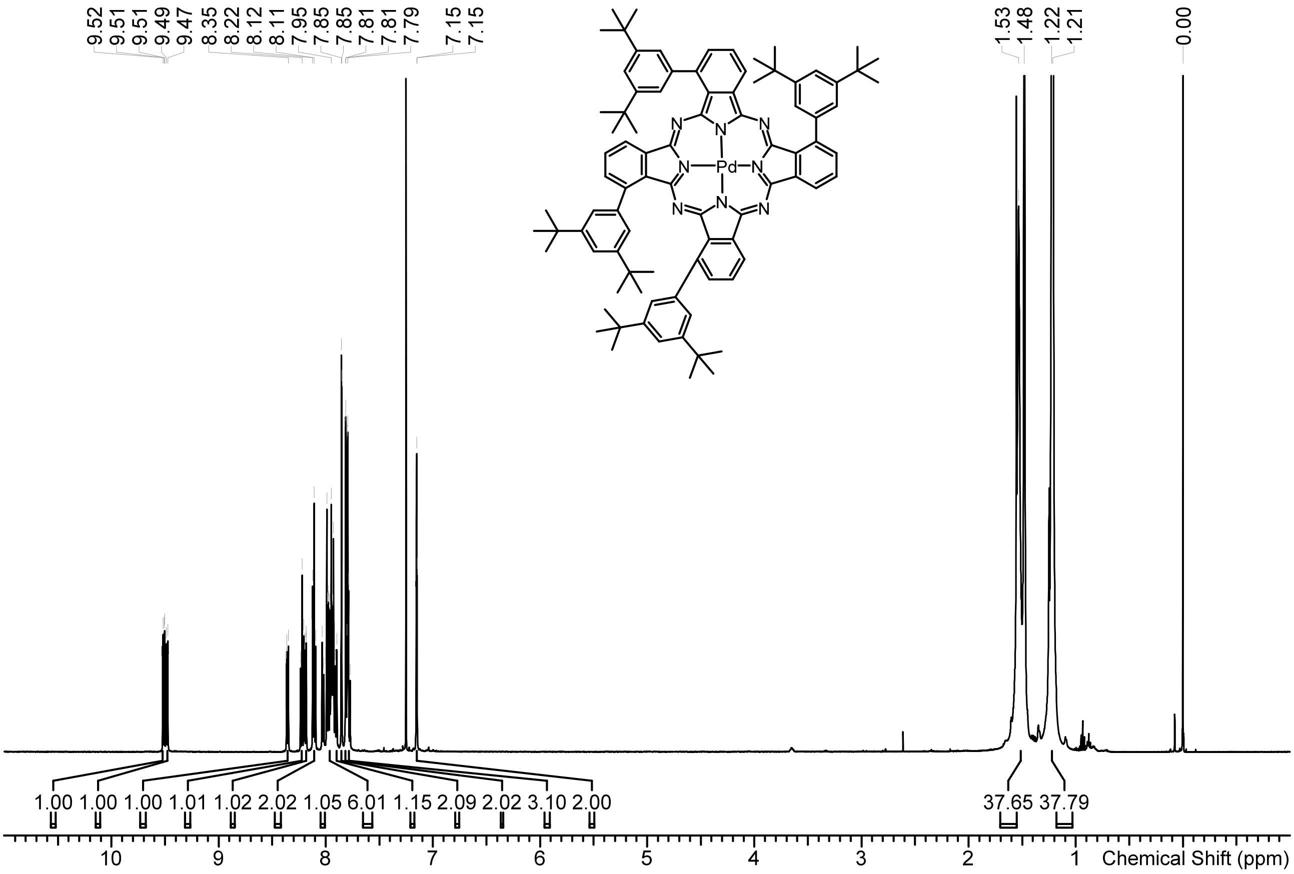

Figure S34: ^{1}H NMR spectrum (500 MHz, $CDCl_3$) of **PdPc-Dragon**.

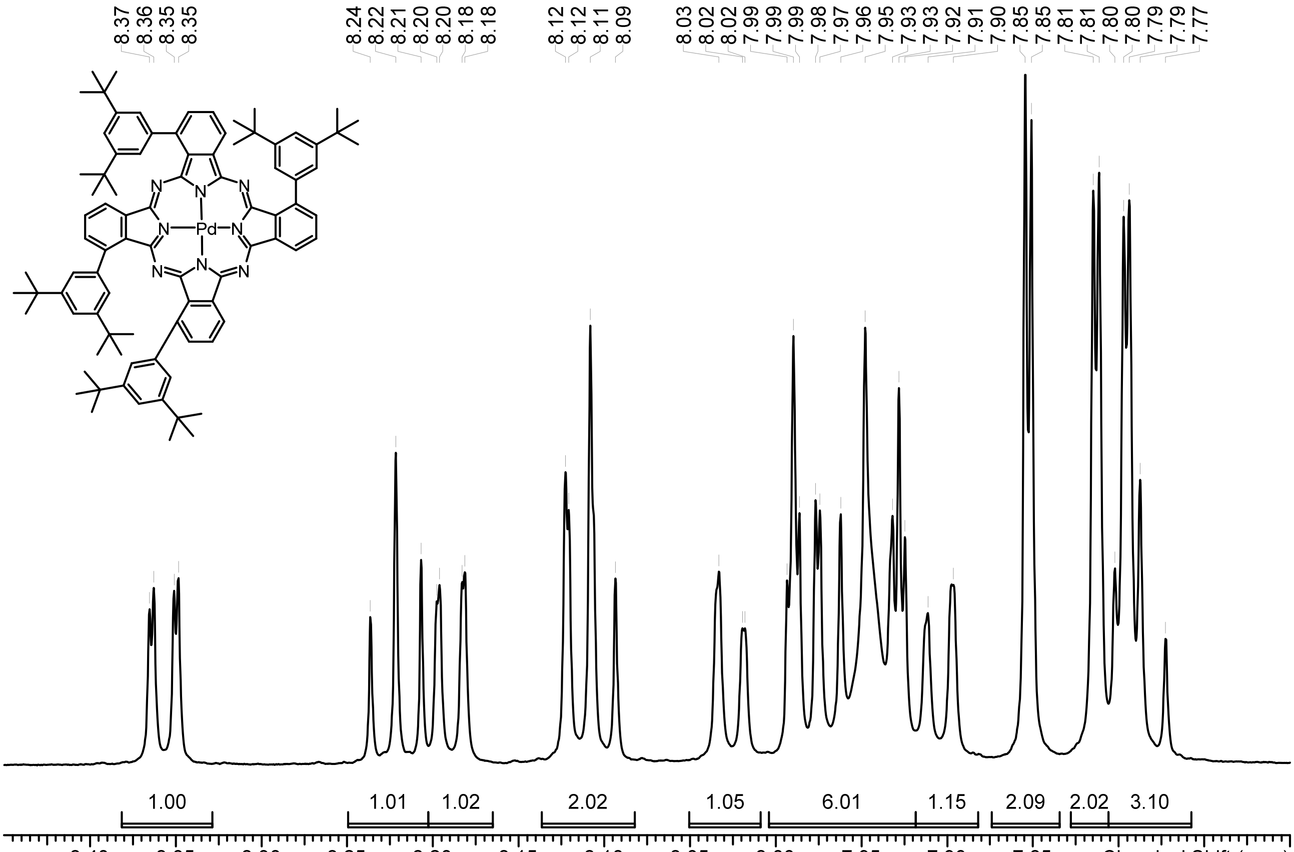

Figure S35: Aromatic region of the ^{1}H NMR spectrum (500 MHz, $CDCl_3$) of **PdPc-Dragon**.

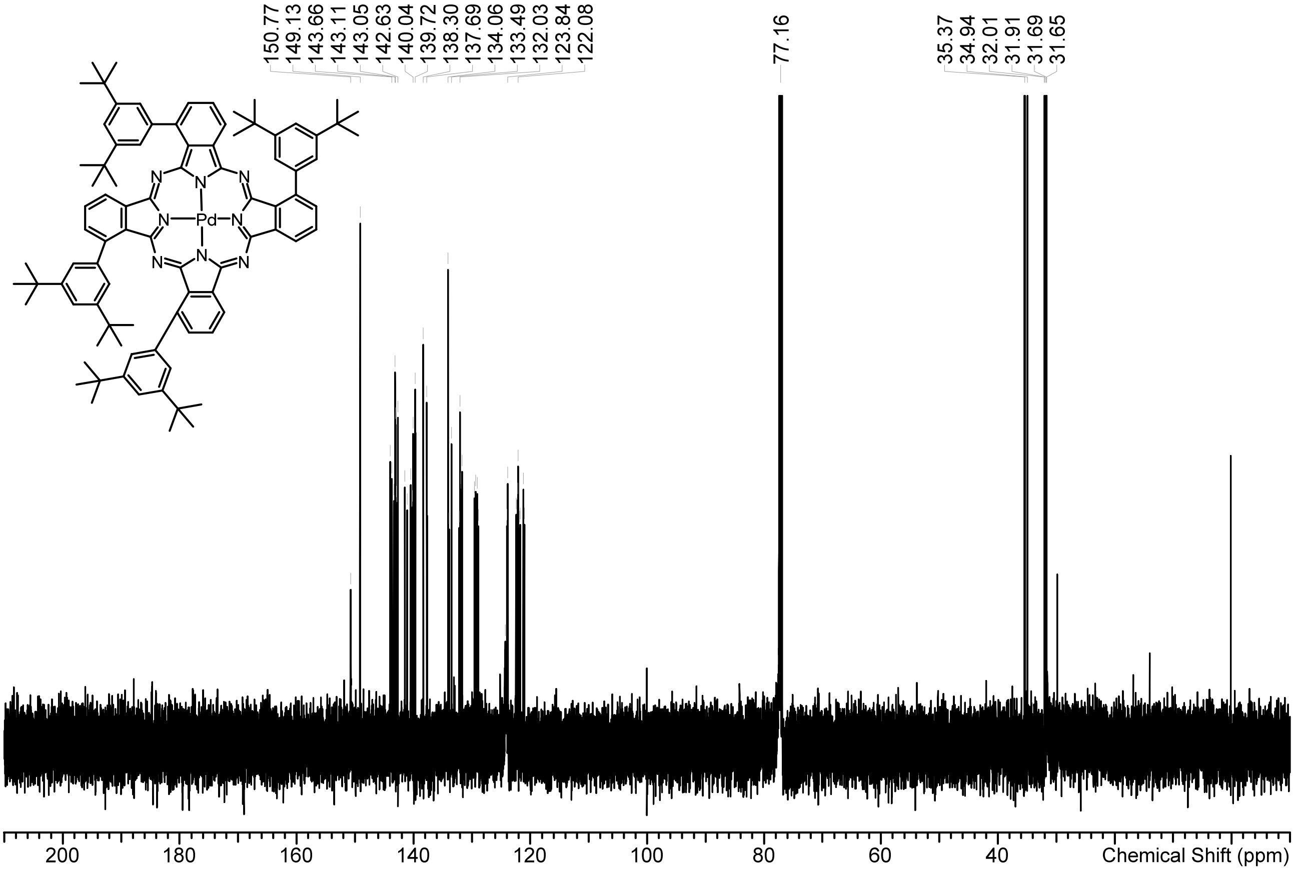

Figure S36: $^{13}C\{^{1}H\}$ NMR spectrum (126 MHz, $CDCl_3$) of **PdPc-Dragon**.

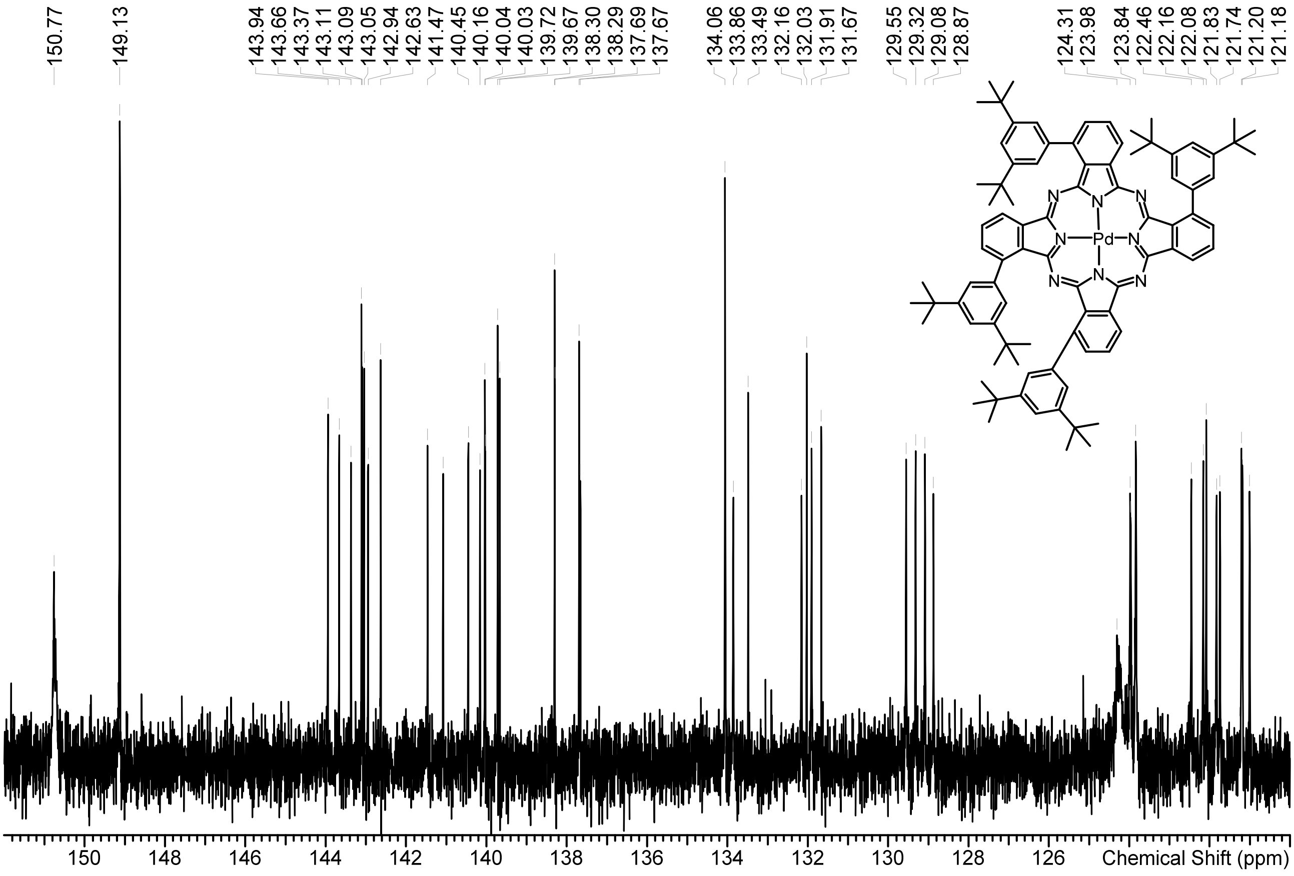

Figure S37: Aromatic region of the $^{13}C\{^{1}H\}$ NMR spectrum (126 MHz, $CDCl_3$) of **PdPc-Dragon**.

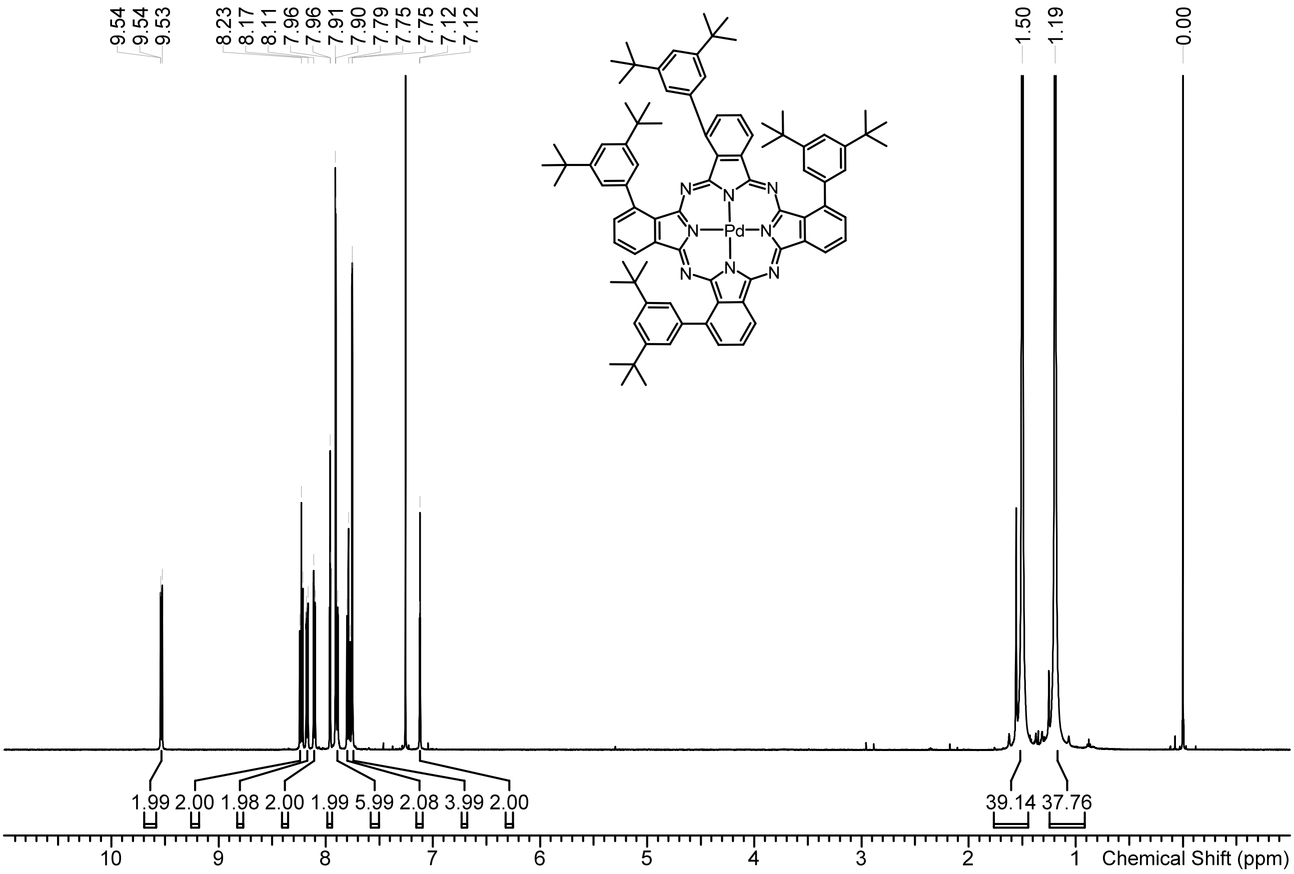

Figure S38: ^{1}H NMR spectrum (500 MHz, $CDCl_3$) of **PdPc-Frog**.

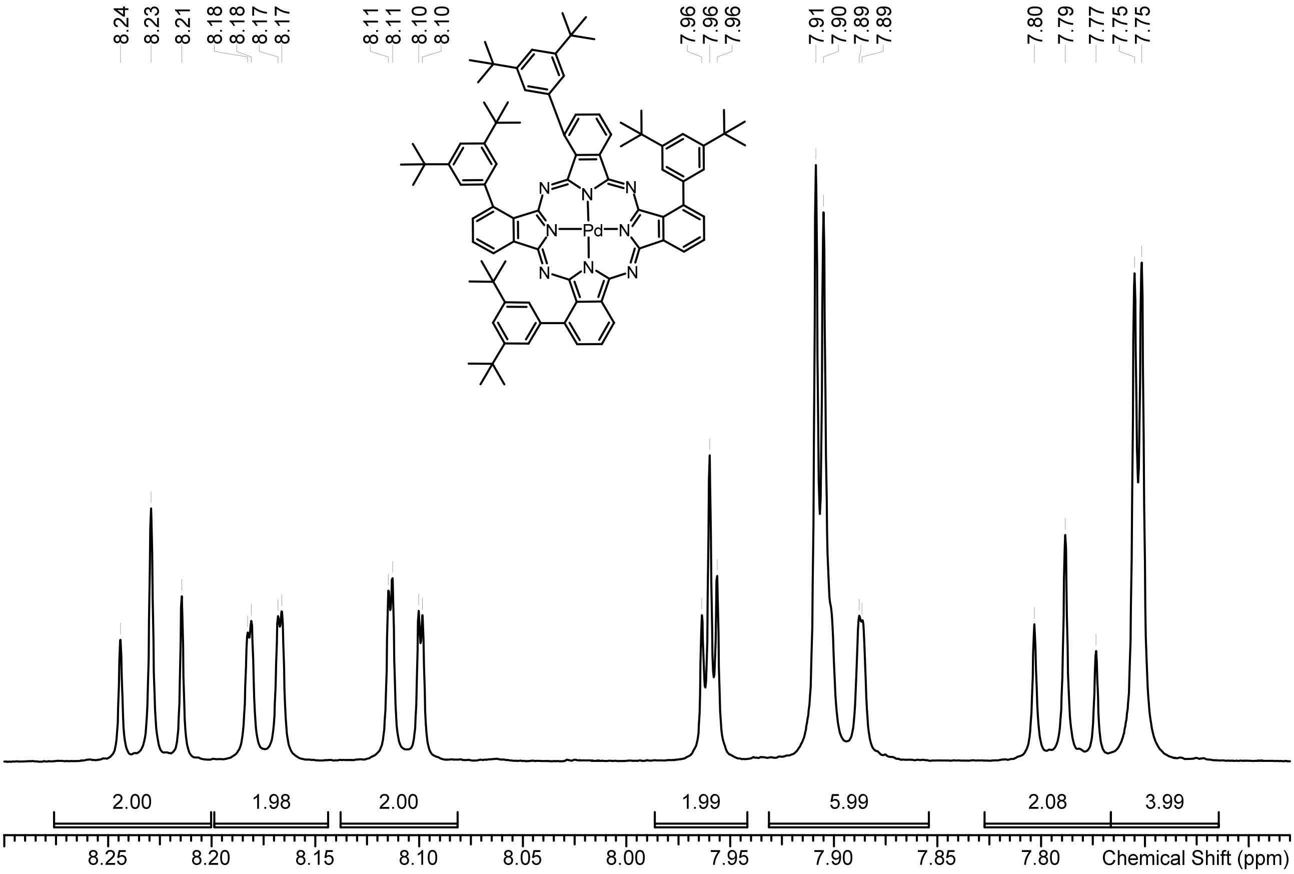

Figure S39: Aromatic region of the ^{1}H NMR spectrum (500 MHz, $CDCl_3$) of **PdPc-Frog**.

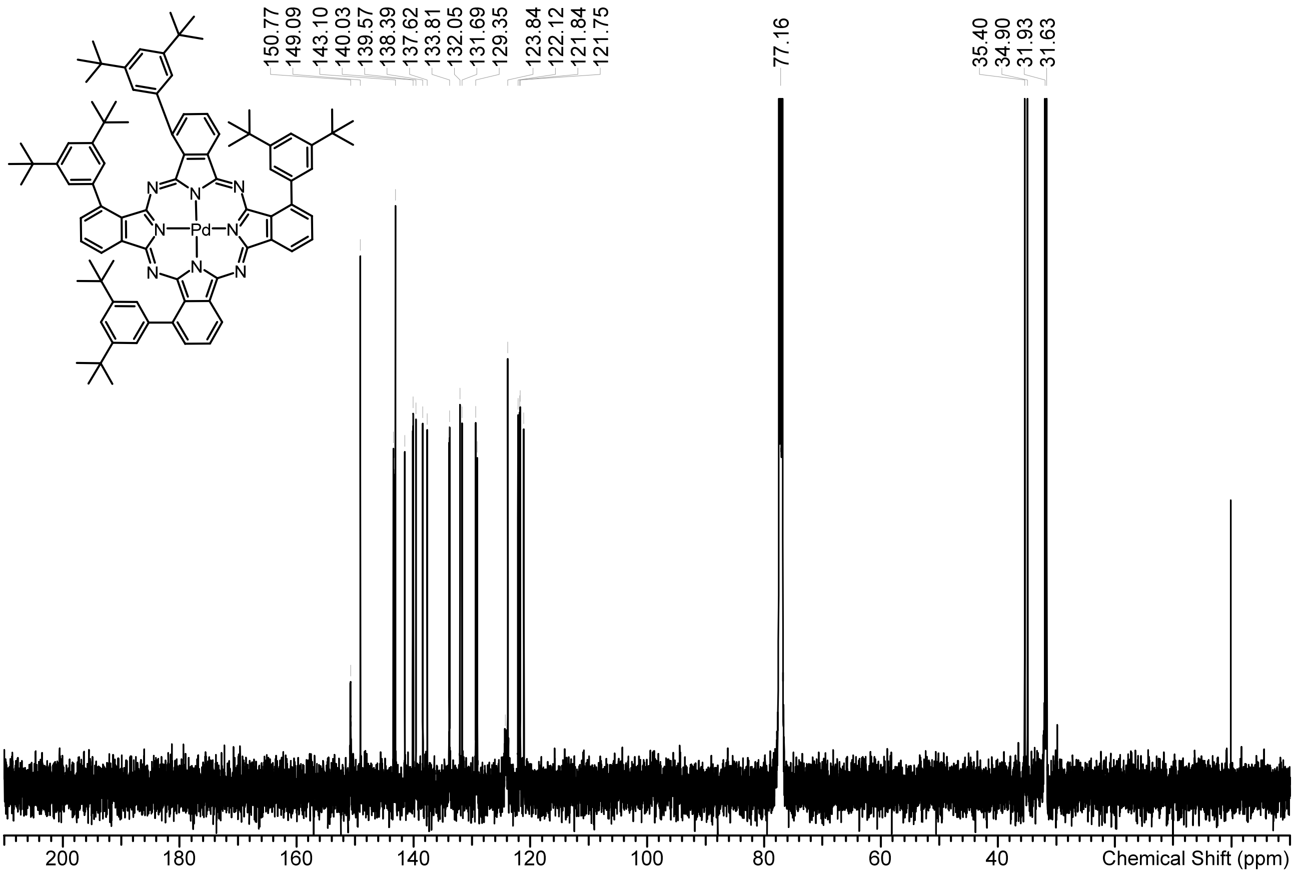

Figure S40: $^{13}C\{^1H\}$ NMR spectrum (126 MHz, $CDCl_3$) of **PdPc-Frog**.

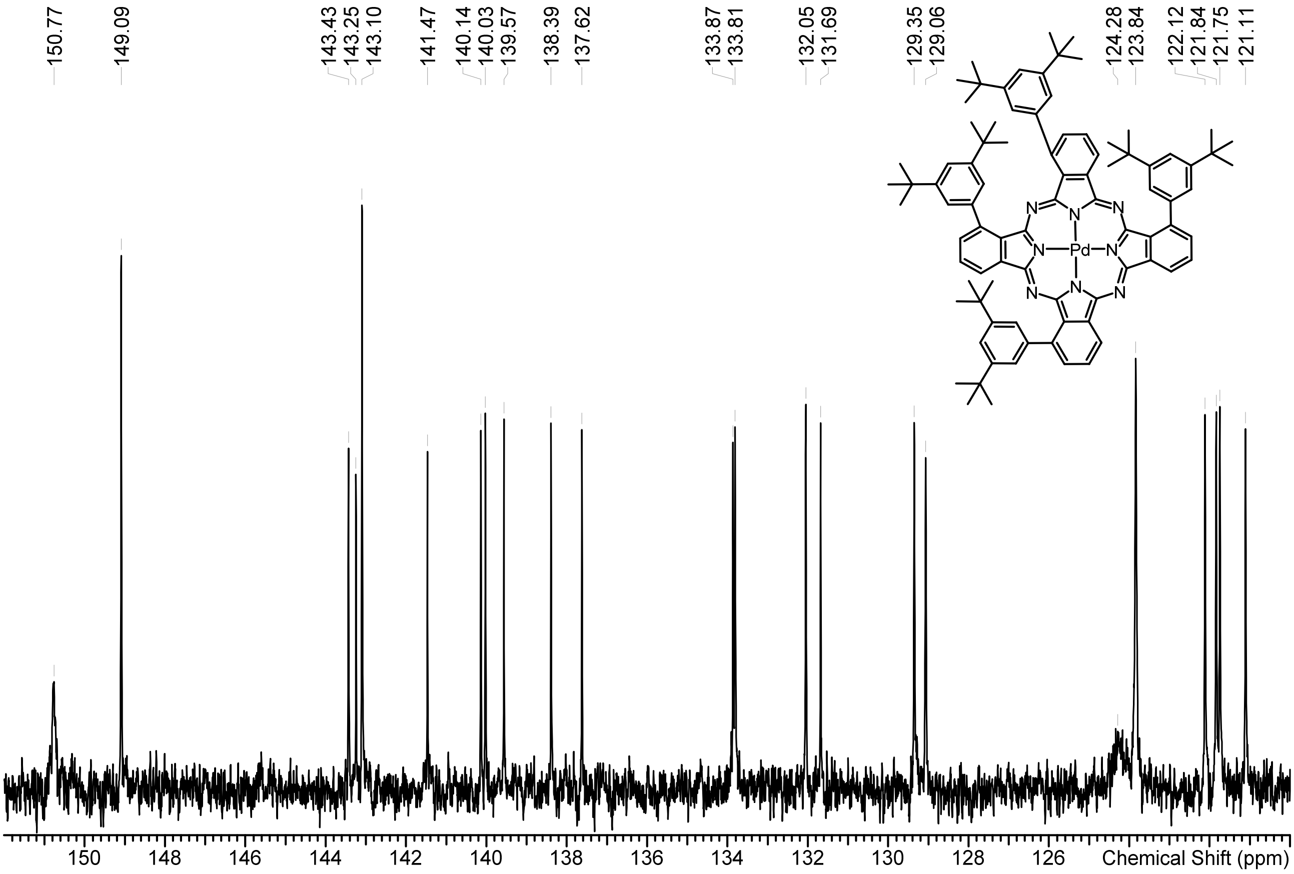

Figure S41: Aromatic region of the $^{13}C\{^1H\}$ NMR spectrum (126 MHz, $CDCl_3$) of **PdPc-Frog**.

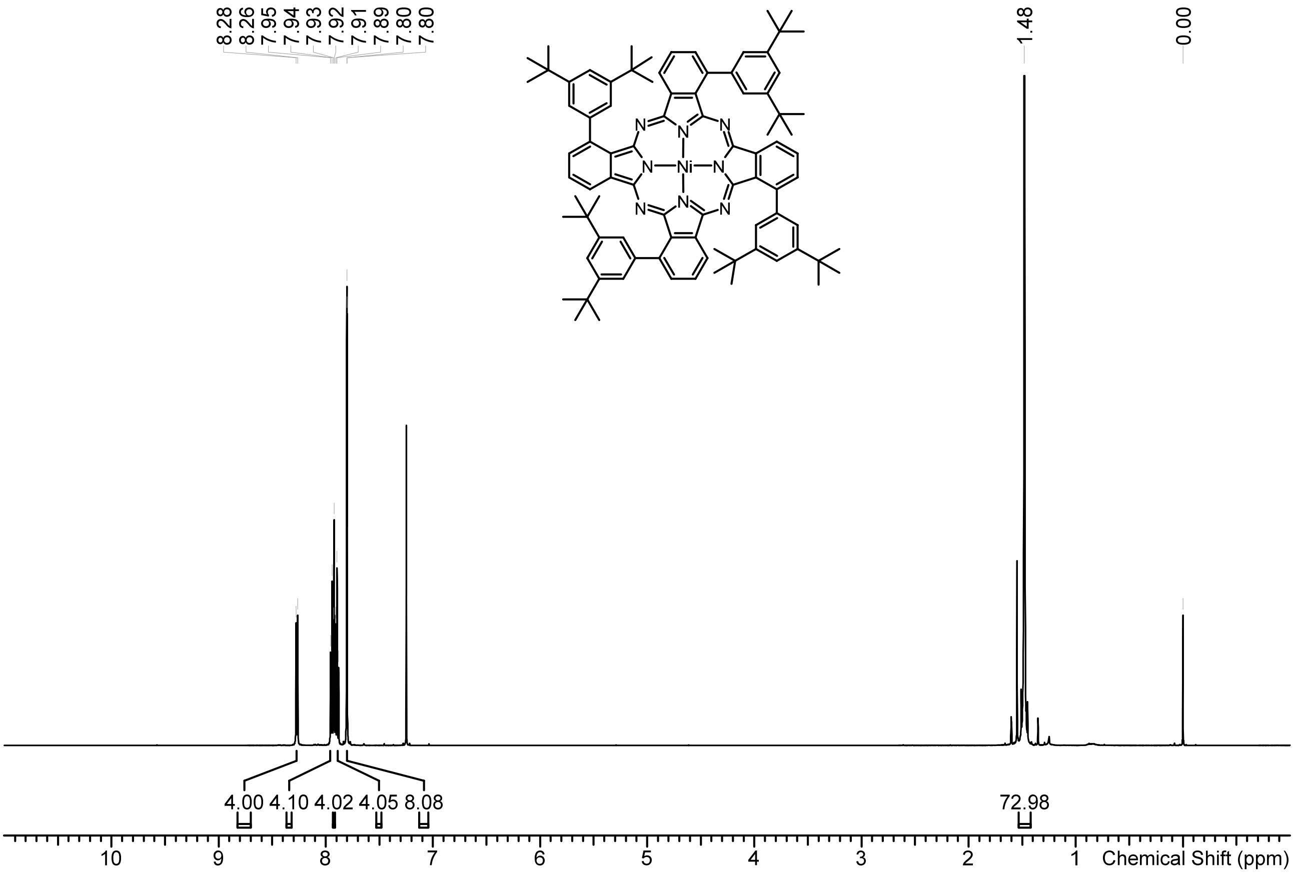

Figure S42: ^{1}H NMR spectrum (500 MHz, $CDCl_3$) of **NiPc-Windmill**.

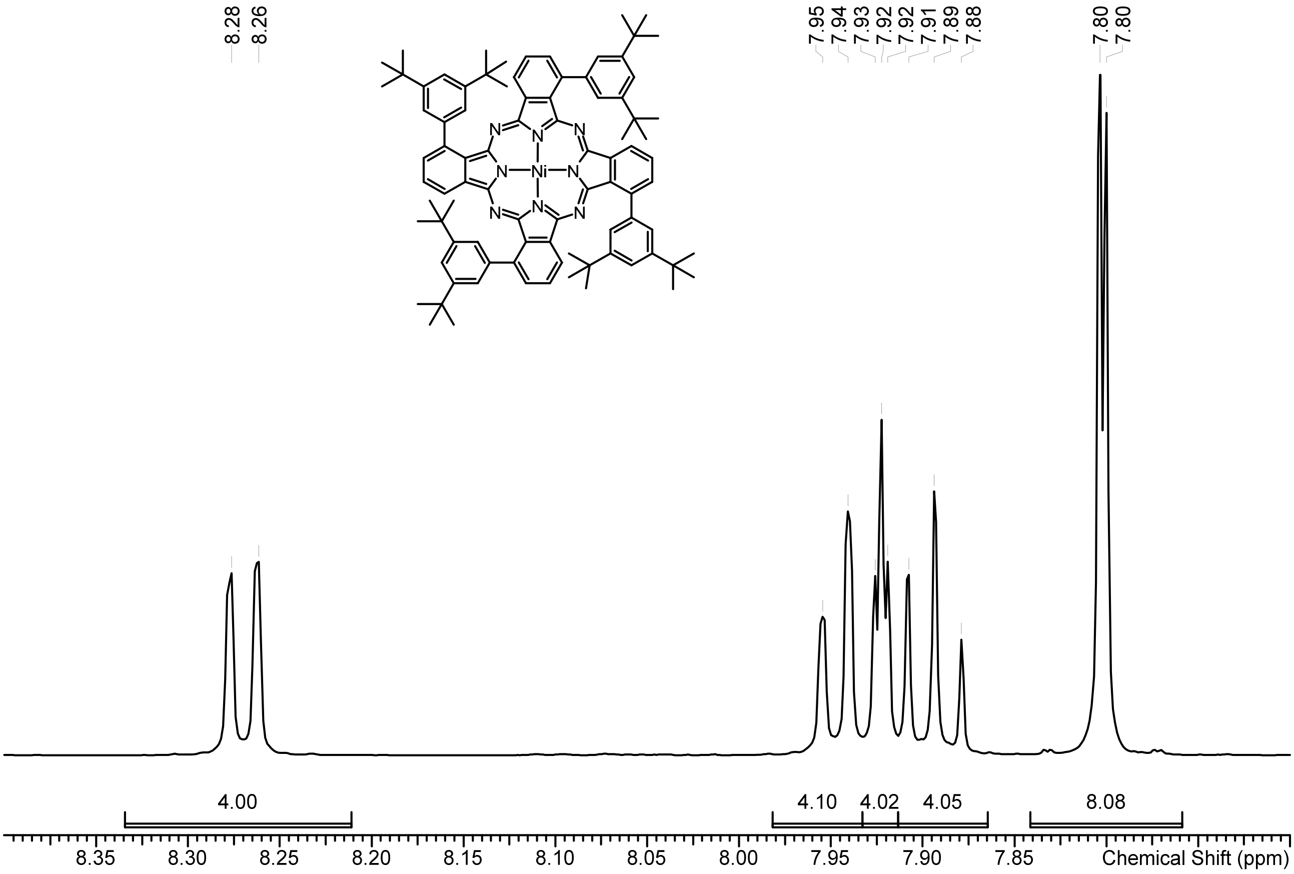

Figure S43: Aromatic region of the ^{1}H NMR spectrum (500 MHz, $CDCl_3$) of **NiPc-Windmill**.

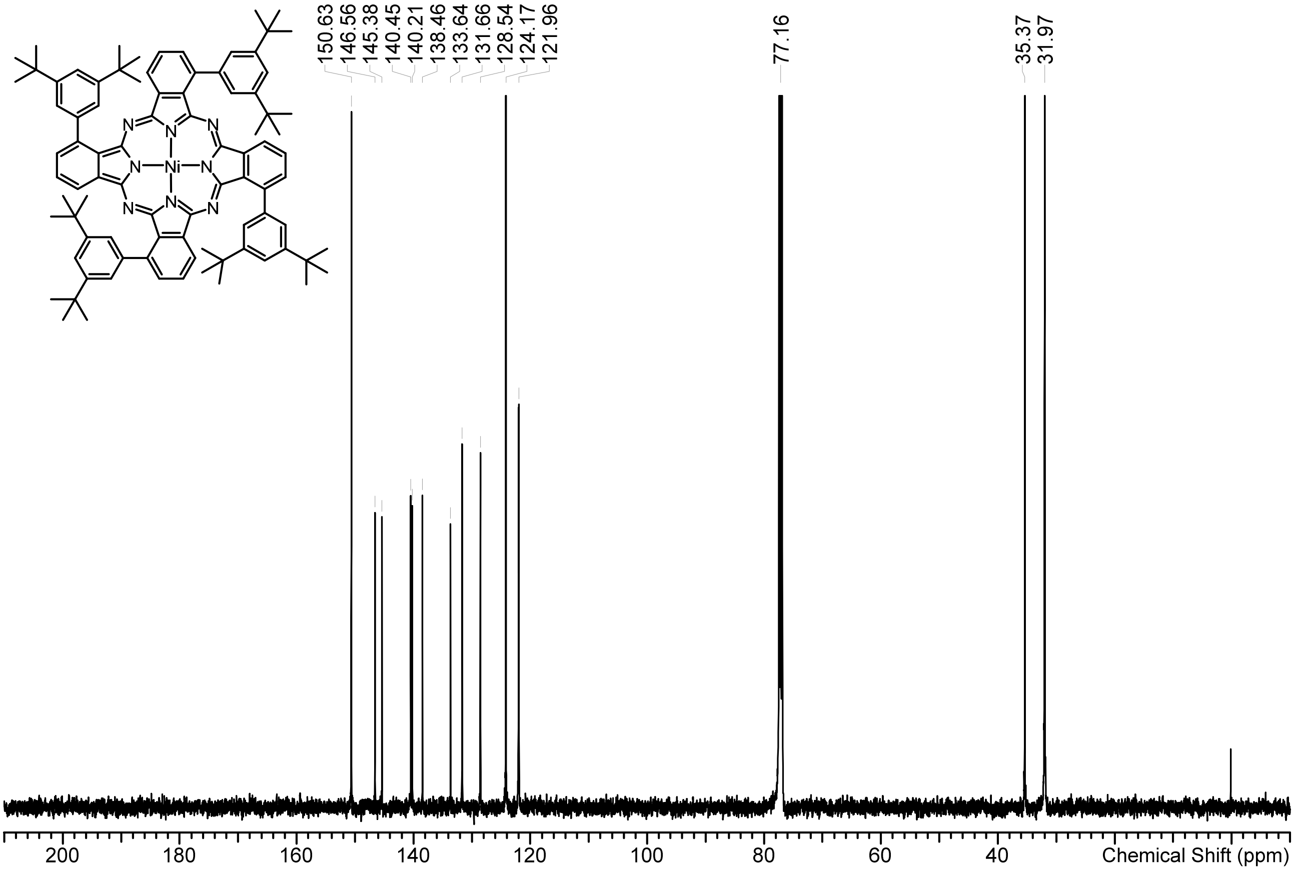

Figure S44: $^{13}C\{^{1}H\}$ NMR spectrum (126 MHz, $CDCl_3$) of **NiPc-Windmill**.

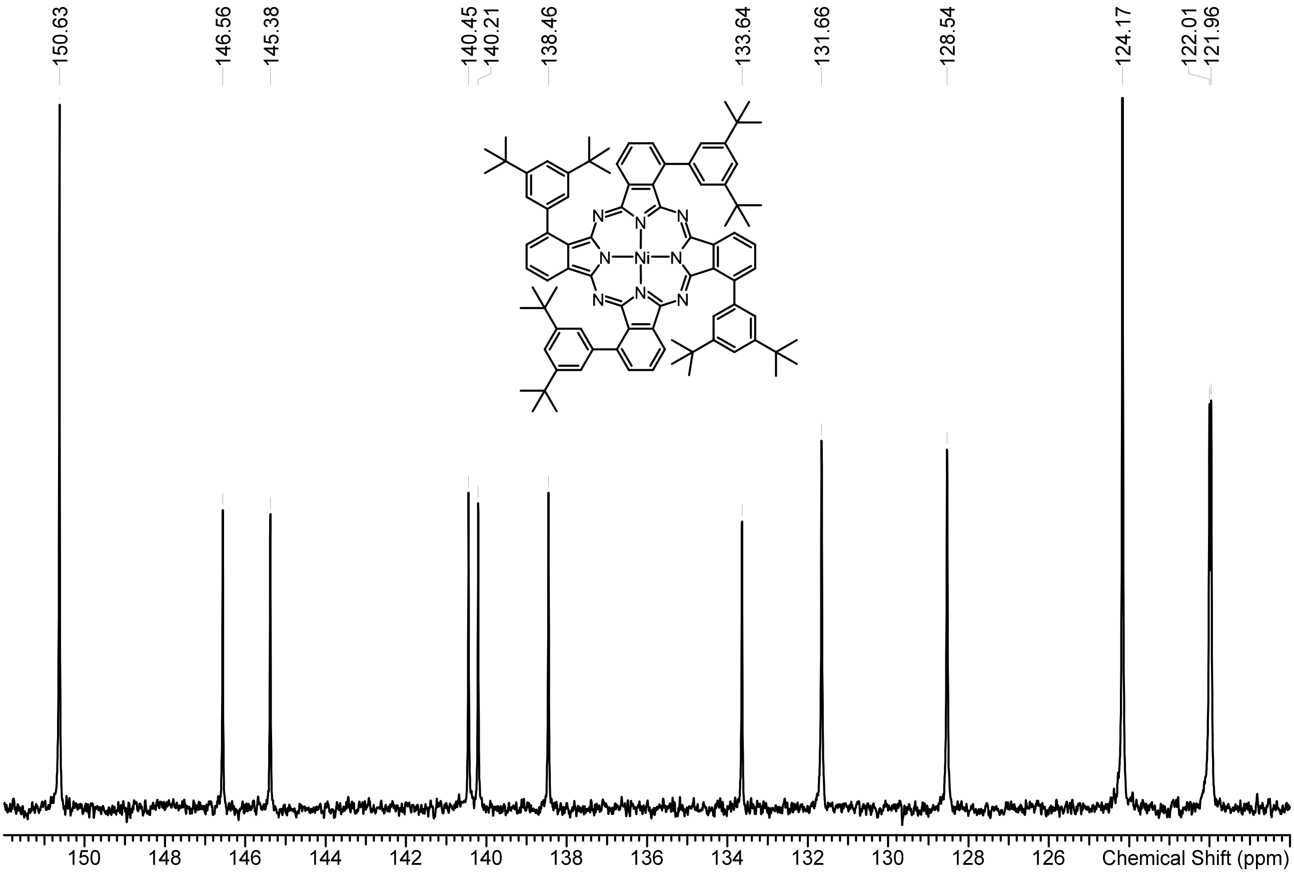

Figure S45: Aromatic region of the $^{13}C\{^{1}H\}$ NMR spectrum (126 MHz, $CDCl_3$) of **NiPc-Windmill**.

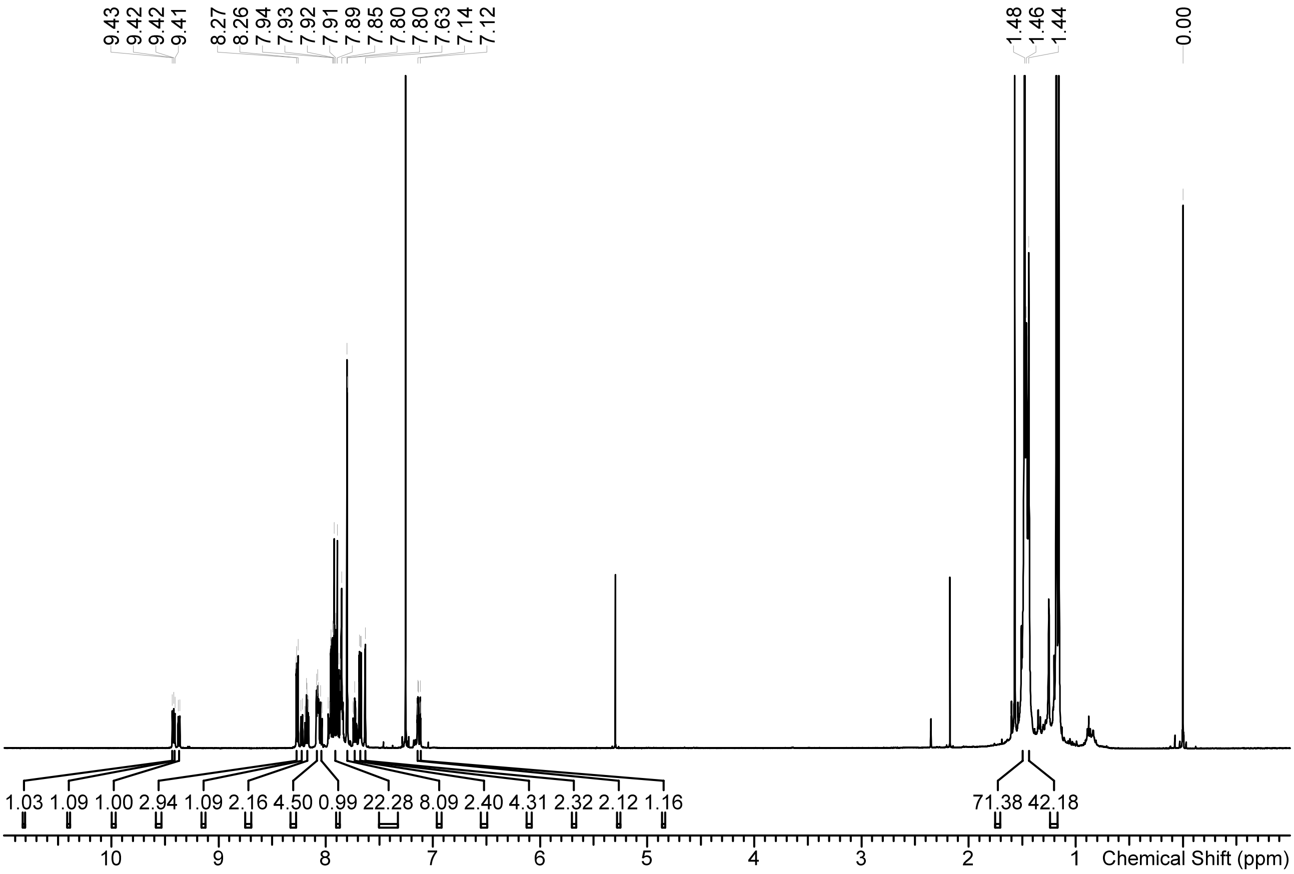

Figure S46: ^{1}H NMR spectrum (500 MHz, $CDCl_3$) of a mixture of the regioisomeric **NiPcs**.

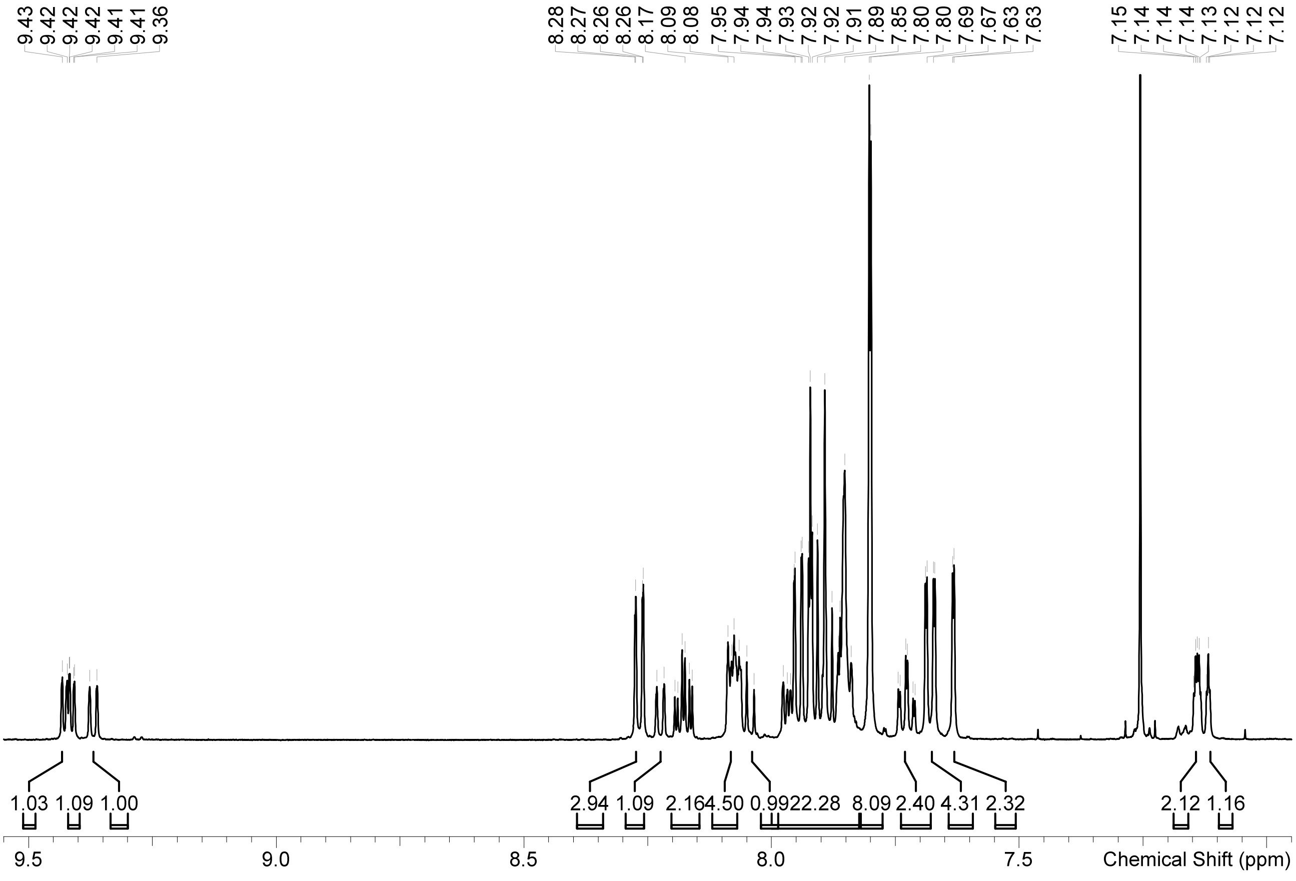

Figure S47: Aromatic region of the ^{1}H NMR spectrum (500 MHz, $CDCl_3$) of a mixture of the regioisomeric **NiPcs**.

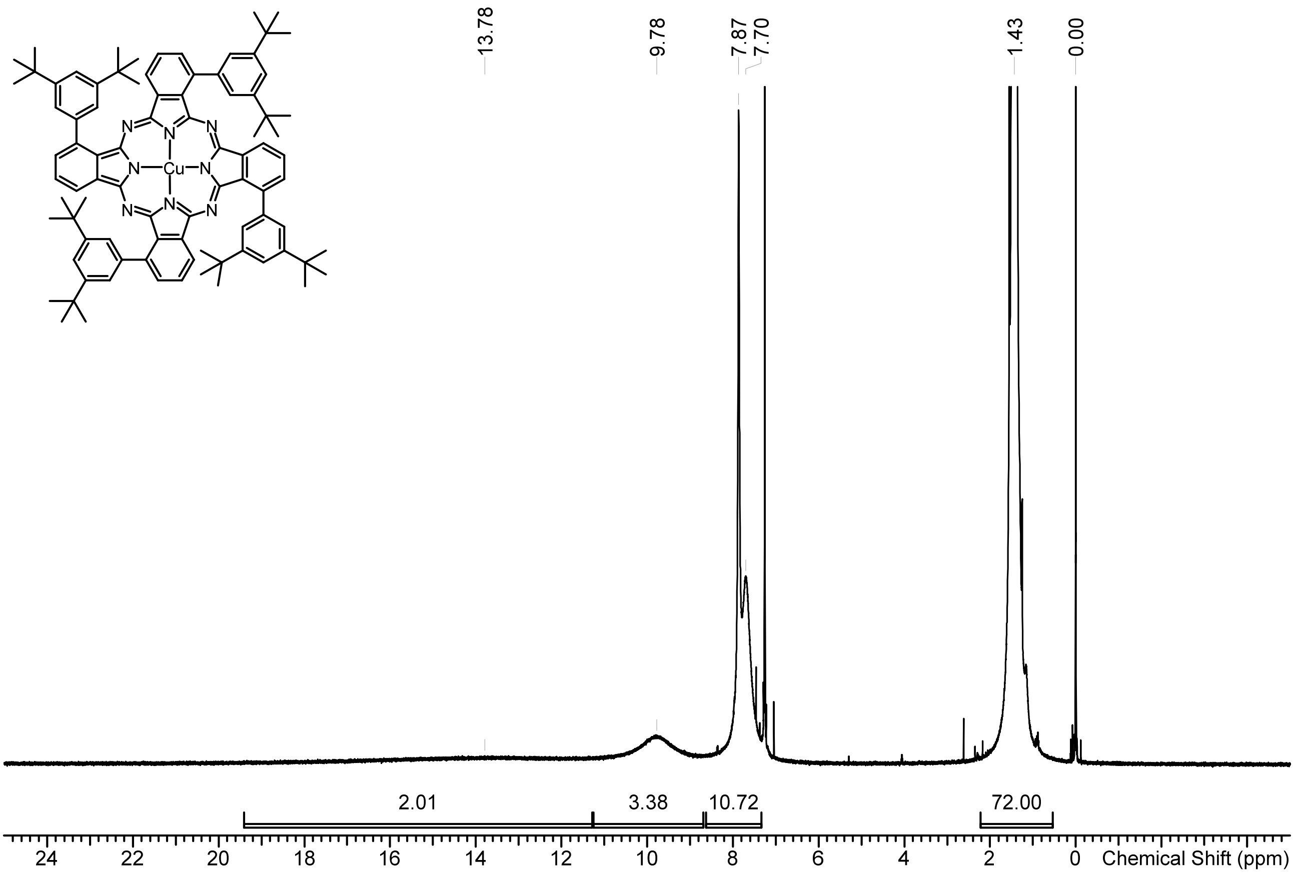

Figure S48: ^{1}H NMR spectrum (500 MHz, $CDCl_3$) of **CuPc-Windmill**.

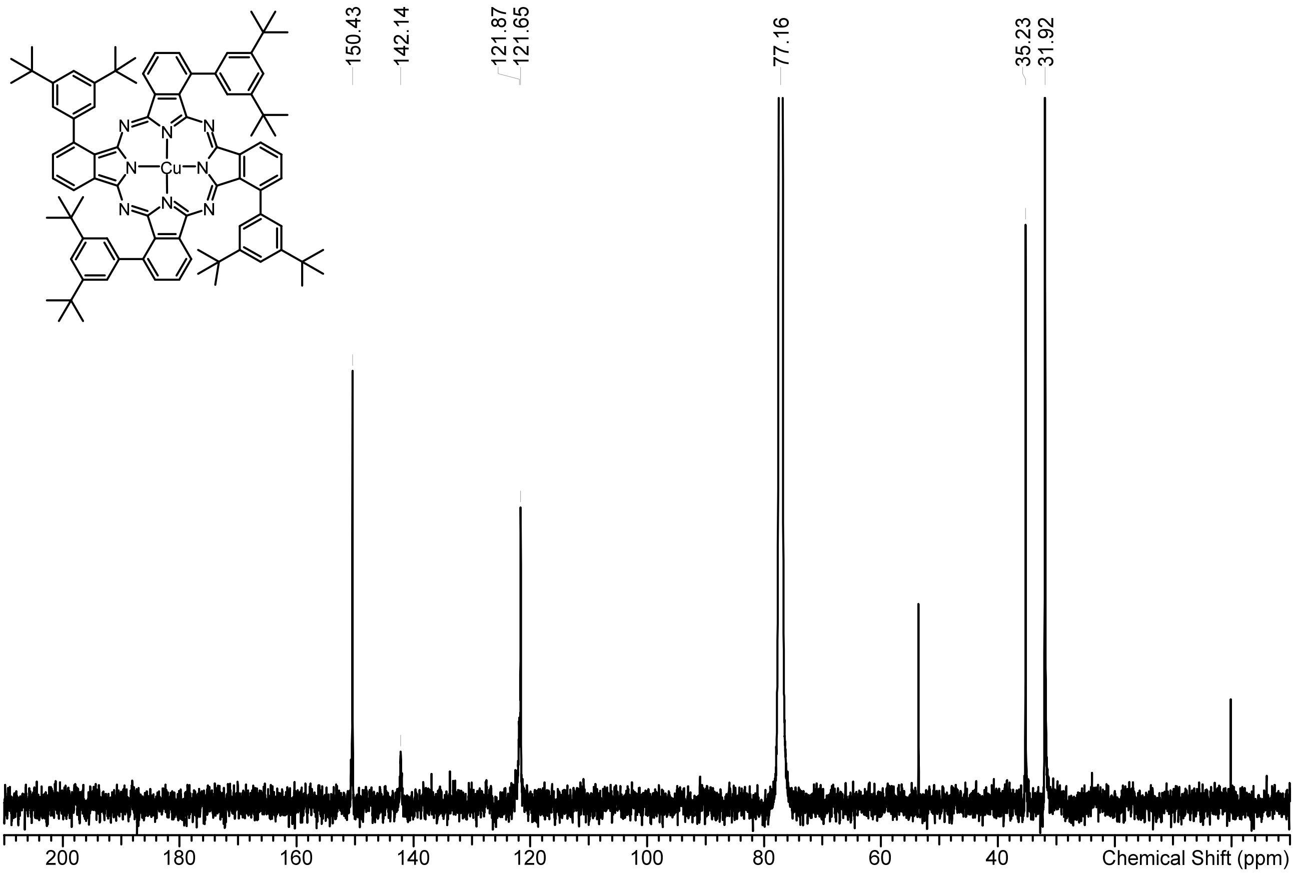

Figure S49: $^{13}C\{^{1}H\}$ NMR spectrum (126 MHz, $CDCl_3$) of **CuPc-Windmill**.

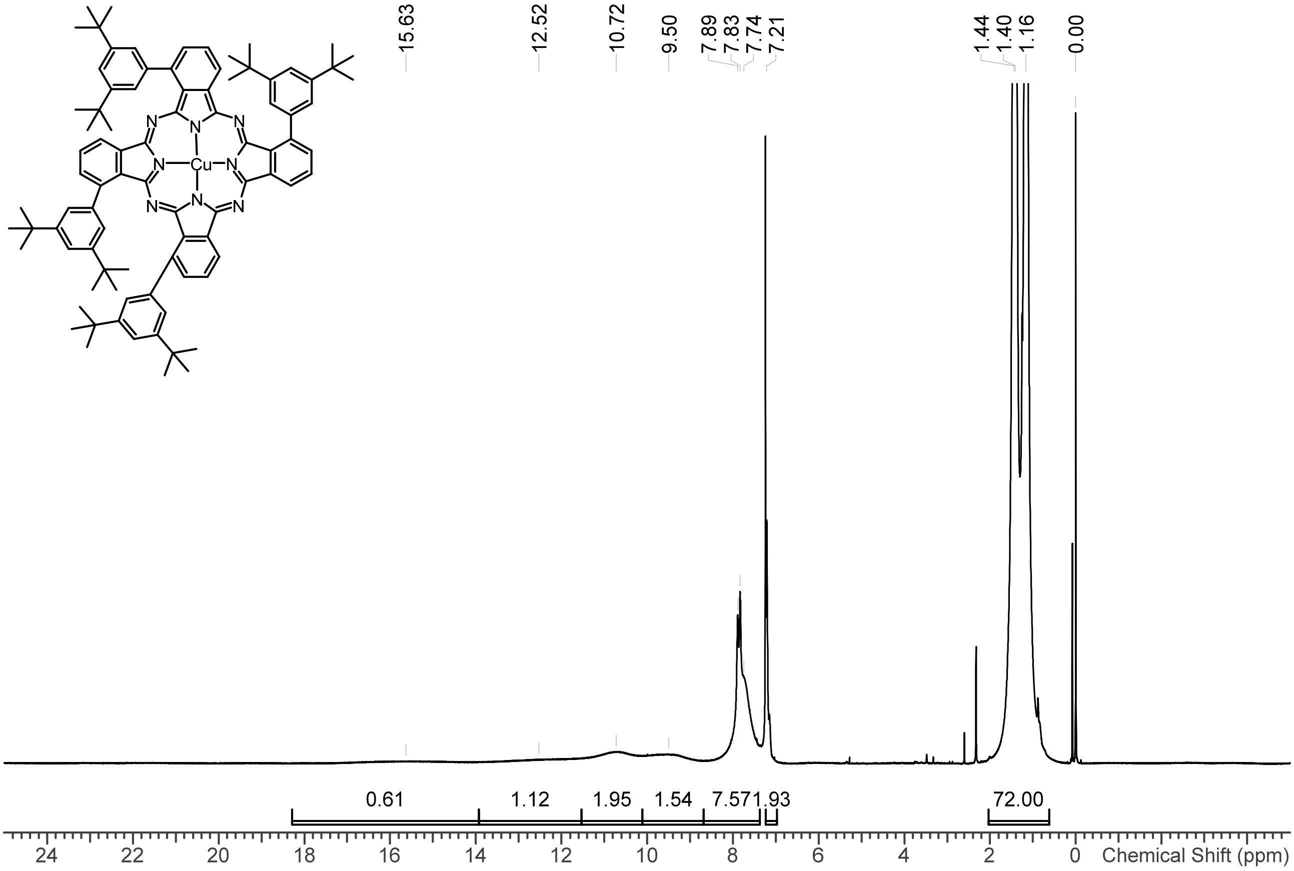

Figure S50: ^{1}H NMR spectrum (500 MHz, $CDCl_3$) of **CuPc-Dragon**.

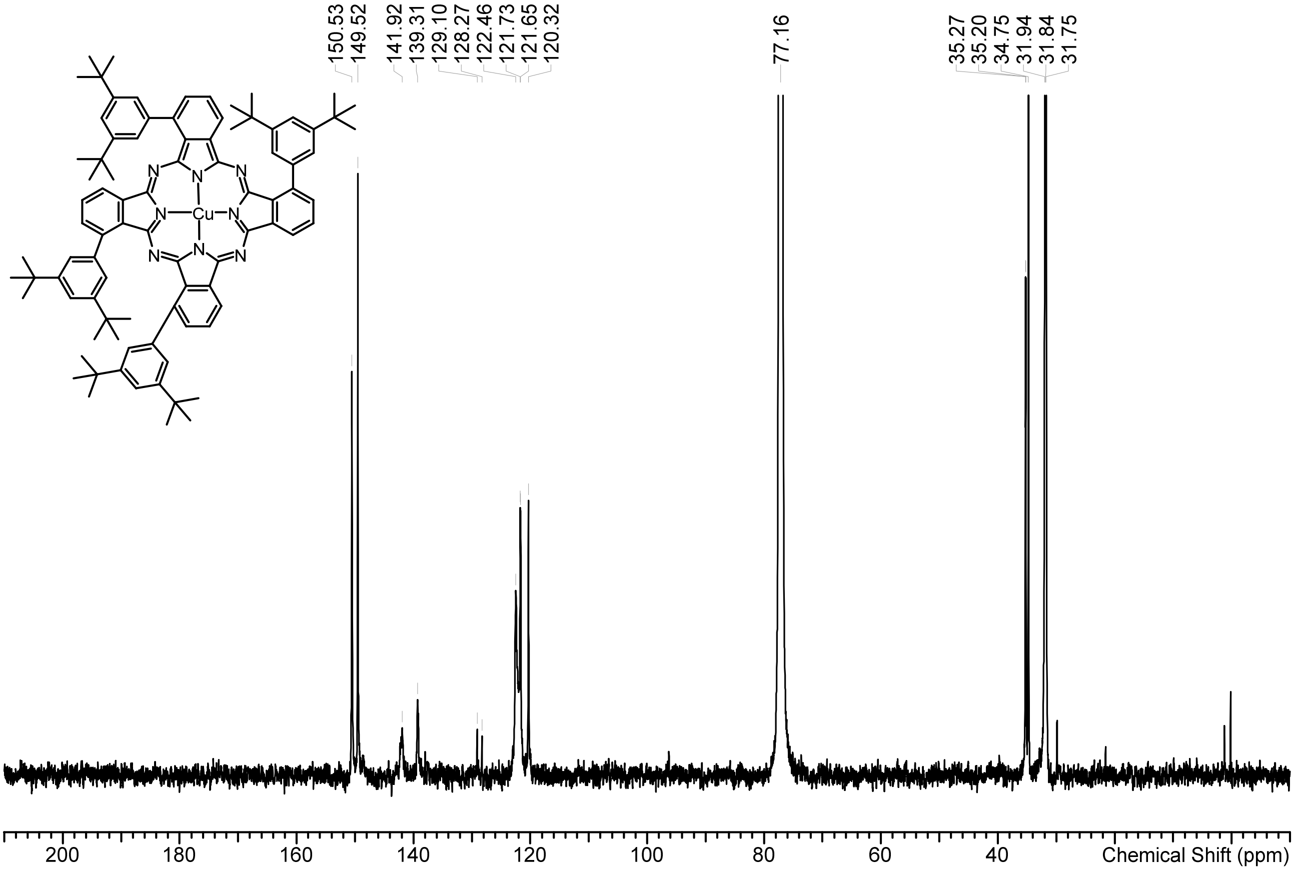

Figure S51: ^{13}C{^{1}H} NMR spectrum (126 MHz, $CDCl_3$) of **CuPc-Dragon**.

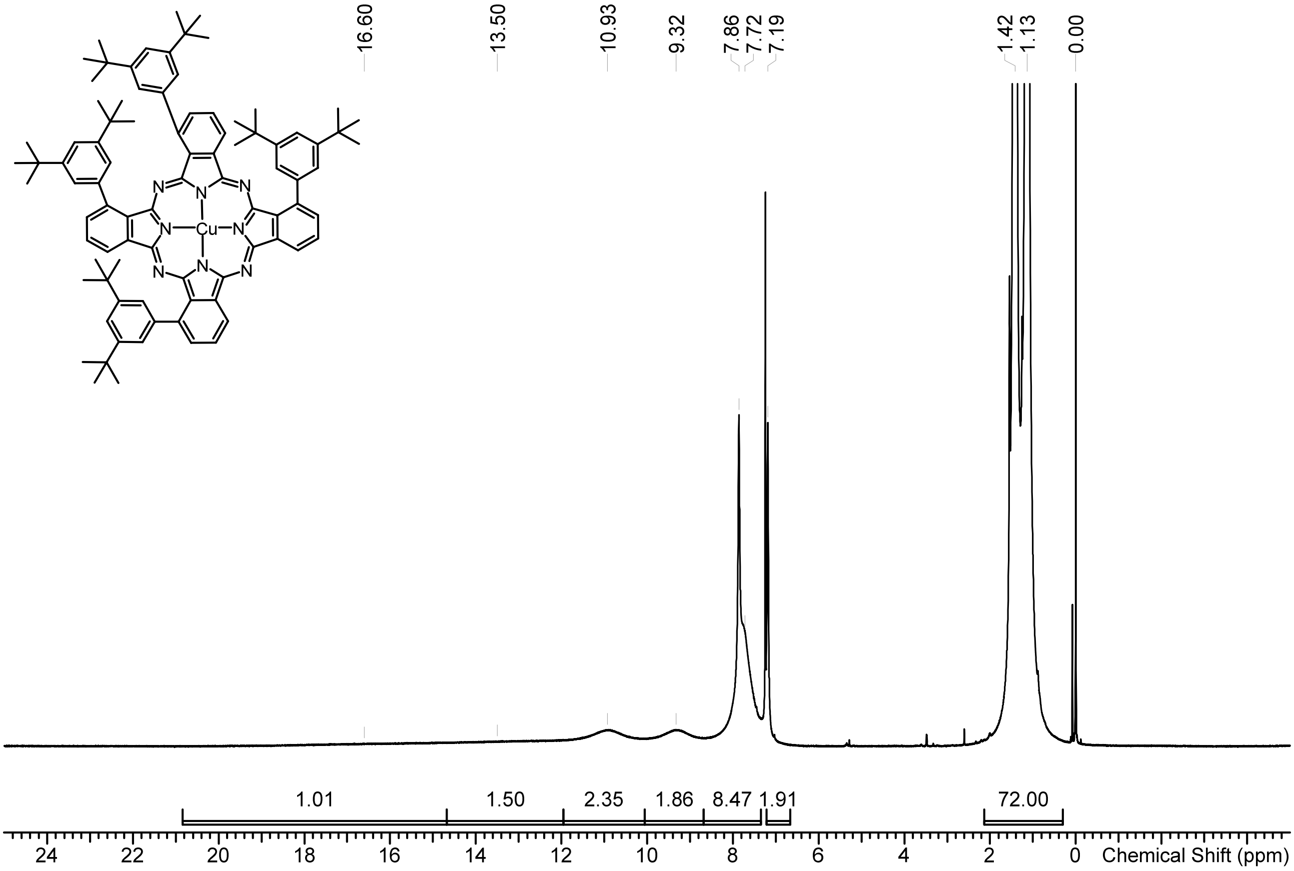

Figure S52: ^{1}H NMR spectrum (500 MHz, $CDCl_3$) of **CuPc-Frog**.

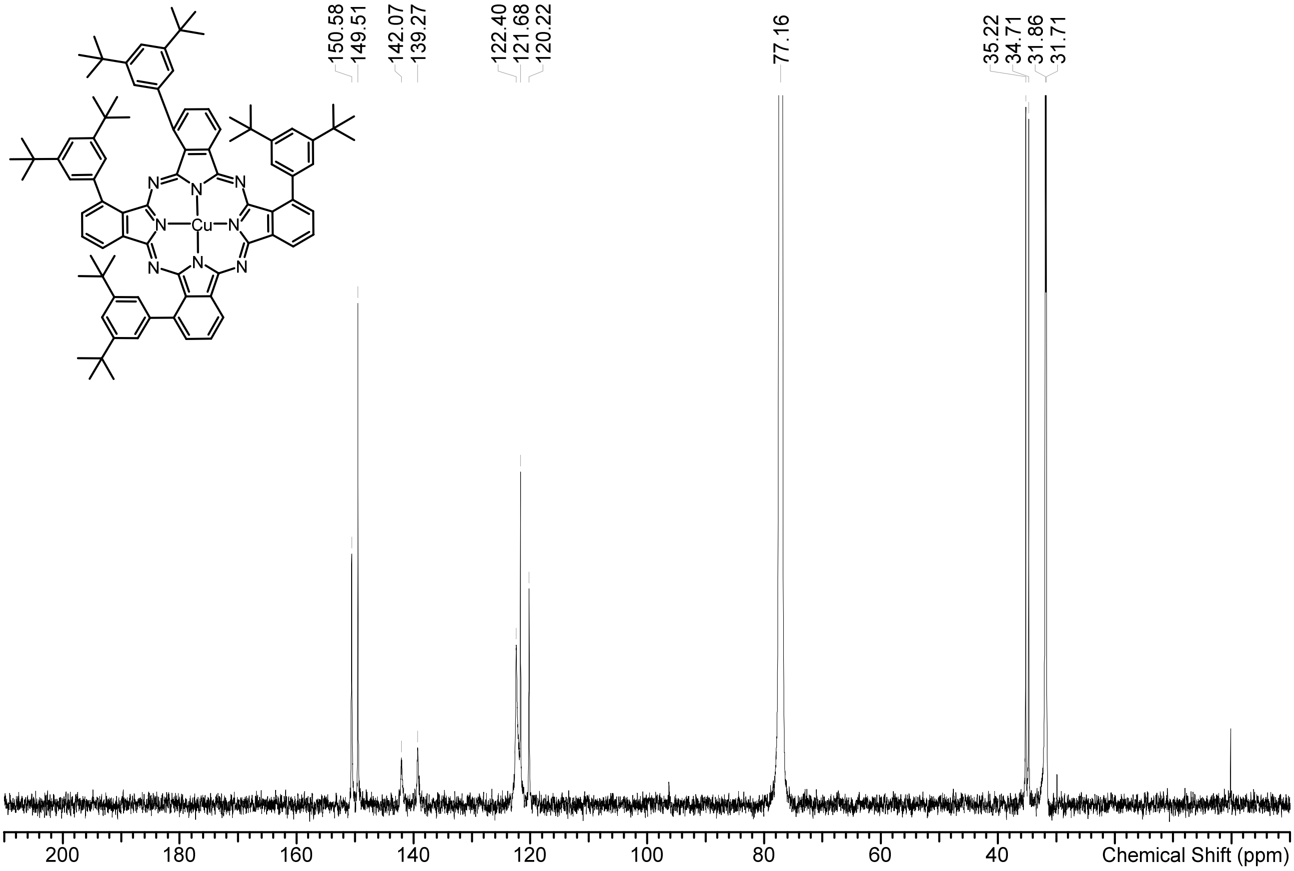

Figure S53: ^{13}C{^{1}H} NMR spectrum (126 MHz, $CDCl_3$) of **CuPc-Frog**.

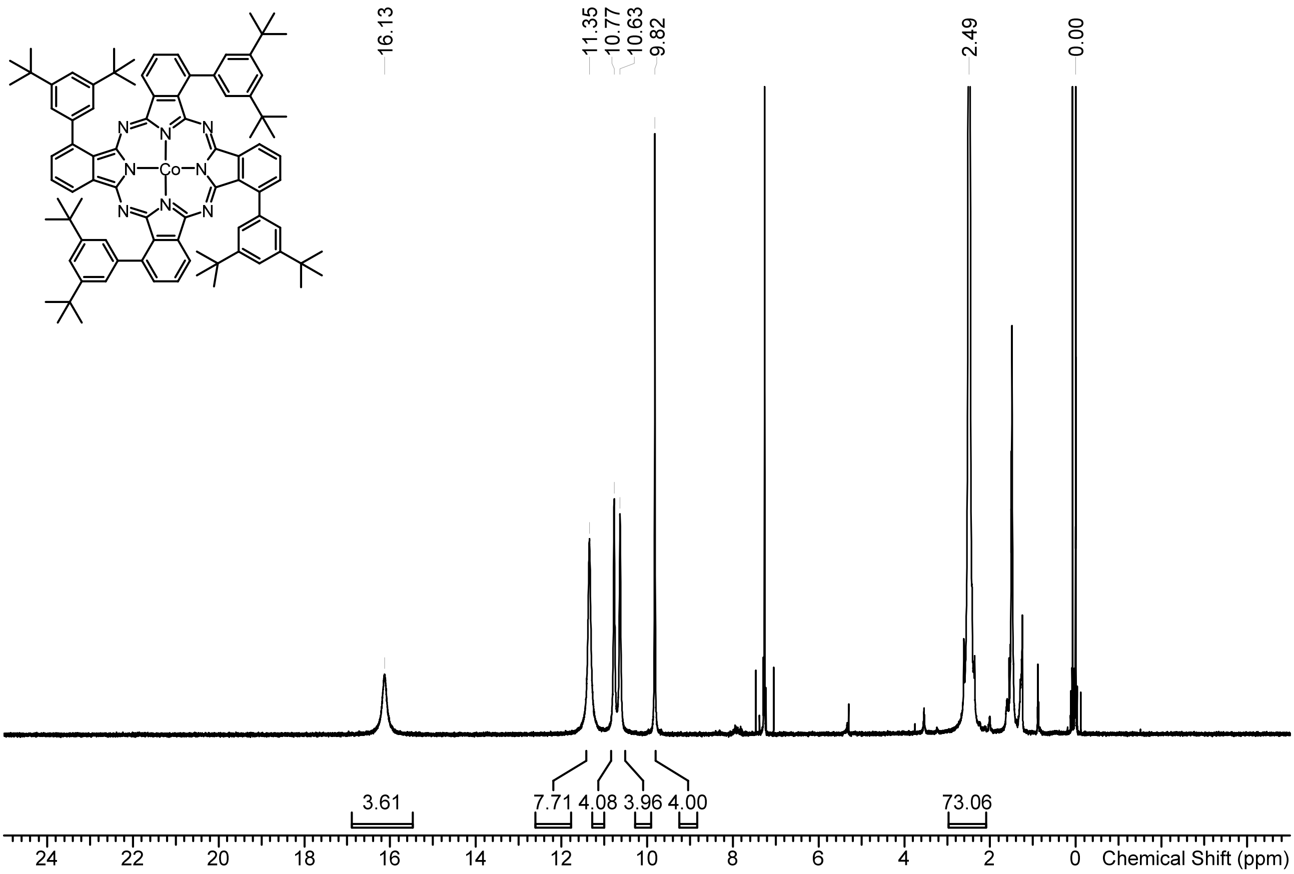

Figure S54: ^{1}H NMR spectrum (500 MHz, $CDCl_3$) of **CoPc-Windmill**.

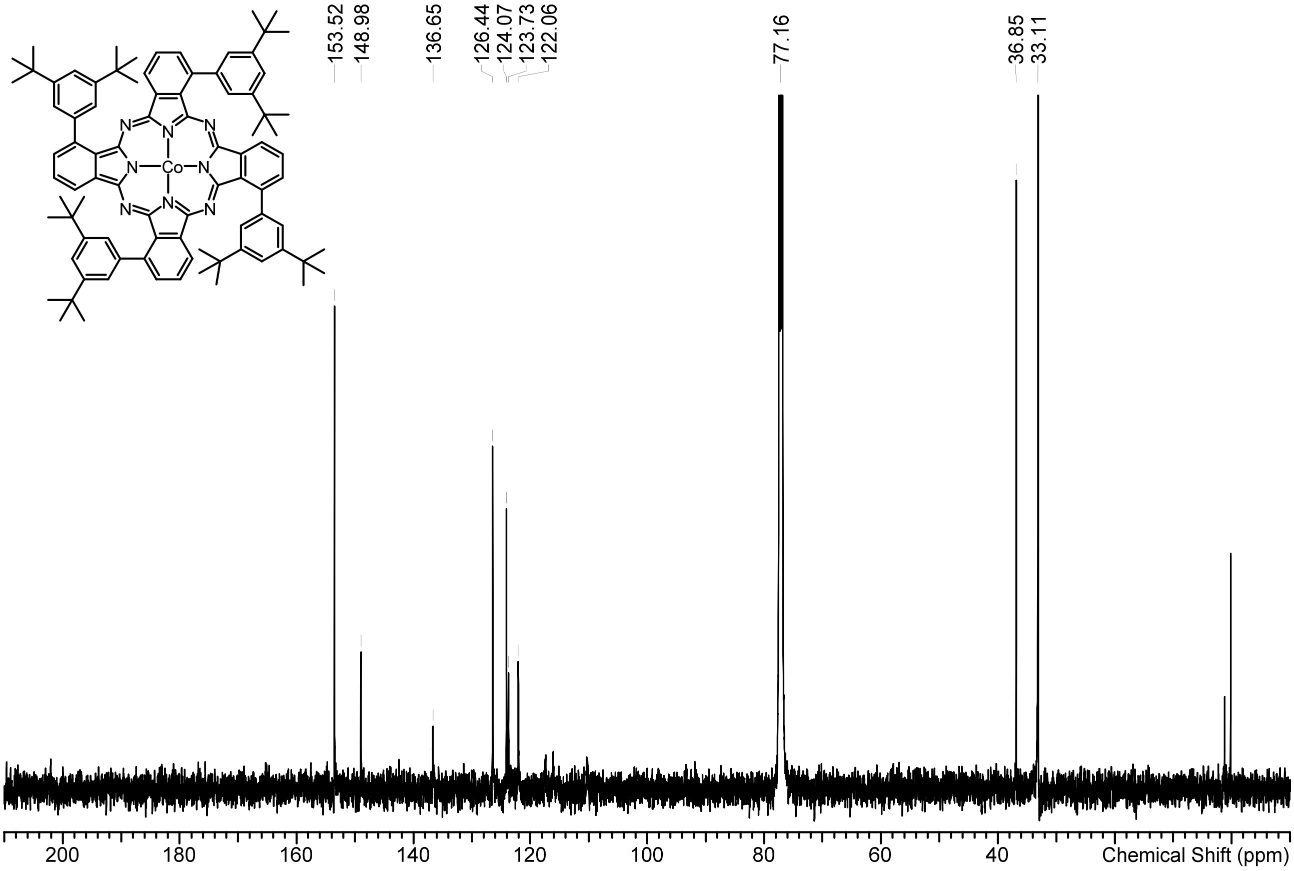

Figure S55: $^{13}C\{^{1}H\}$ NMR spectrum (126 MHz, $CDCl_3$) of **CoPc-Windmill**.

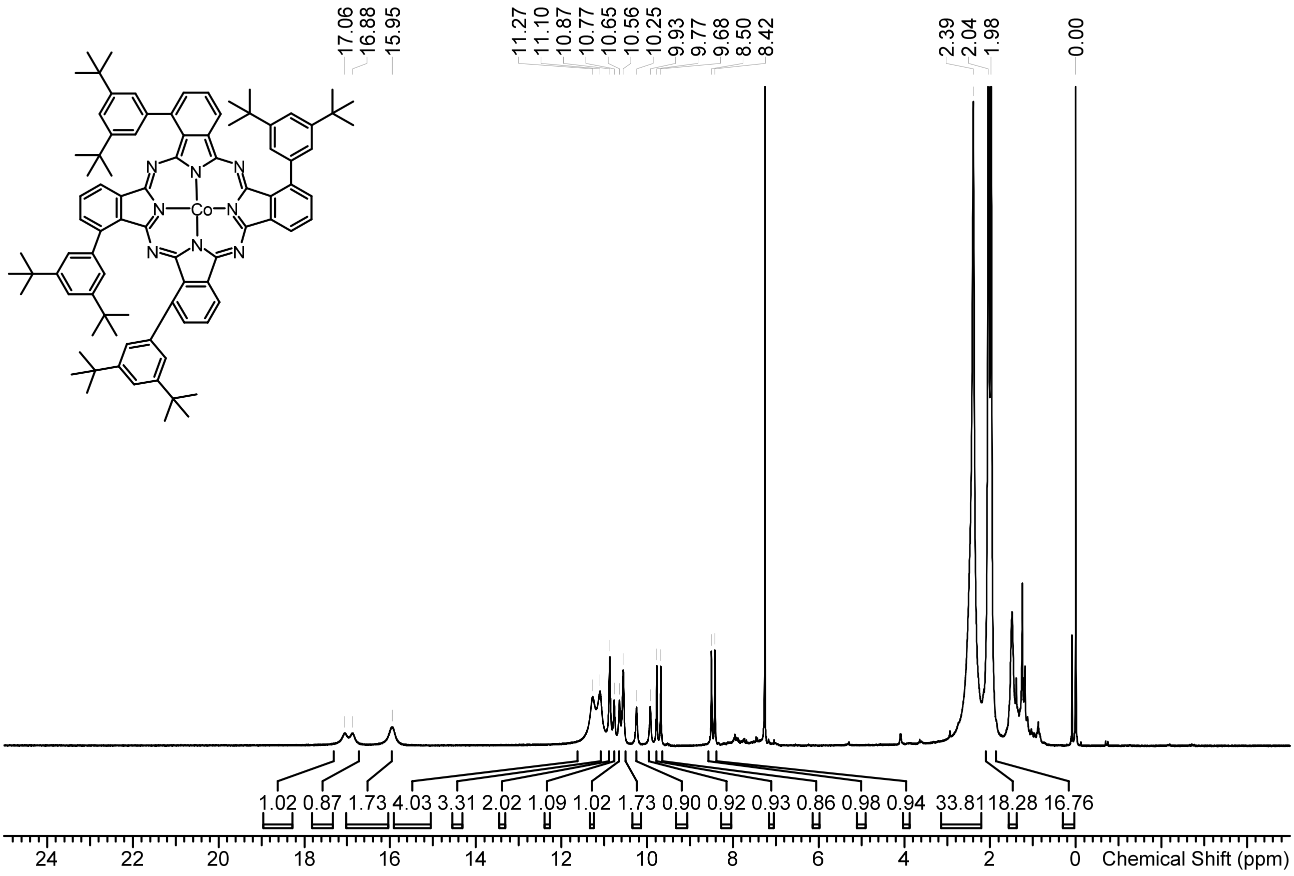

Figure S56: ^{1}H NMR spectrum (500 MHz, $CDCl_3$) of **CoPc-Dragon**.

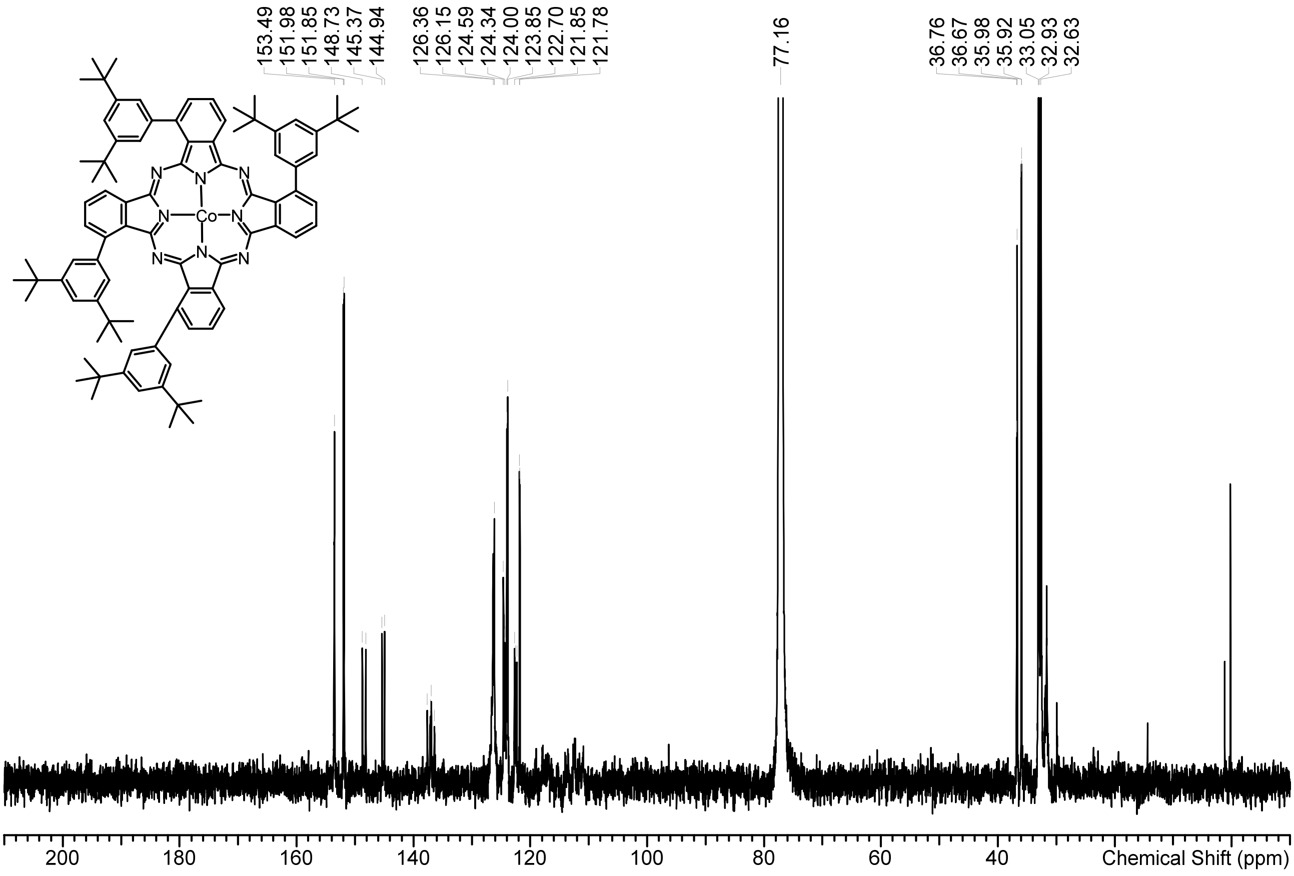

Figure S57: $^{13}C\{^{1}H\}$ NMR spectrum (126 MHz, $CDCl_3$) of **CoPc- Dragon**.

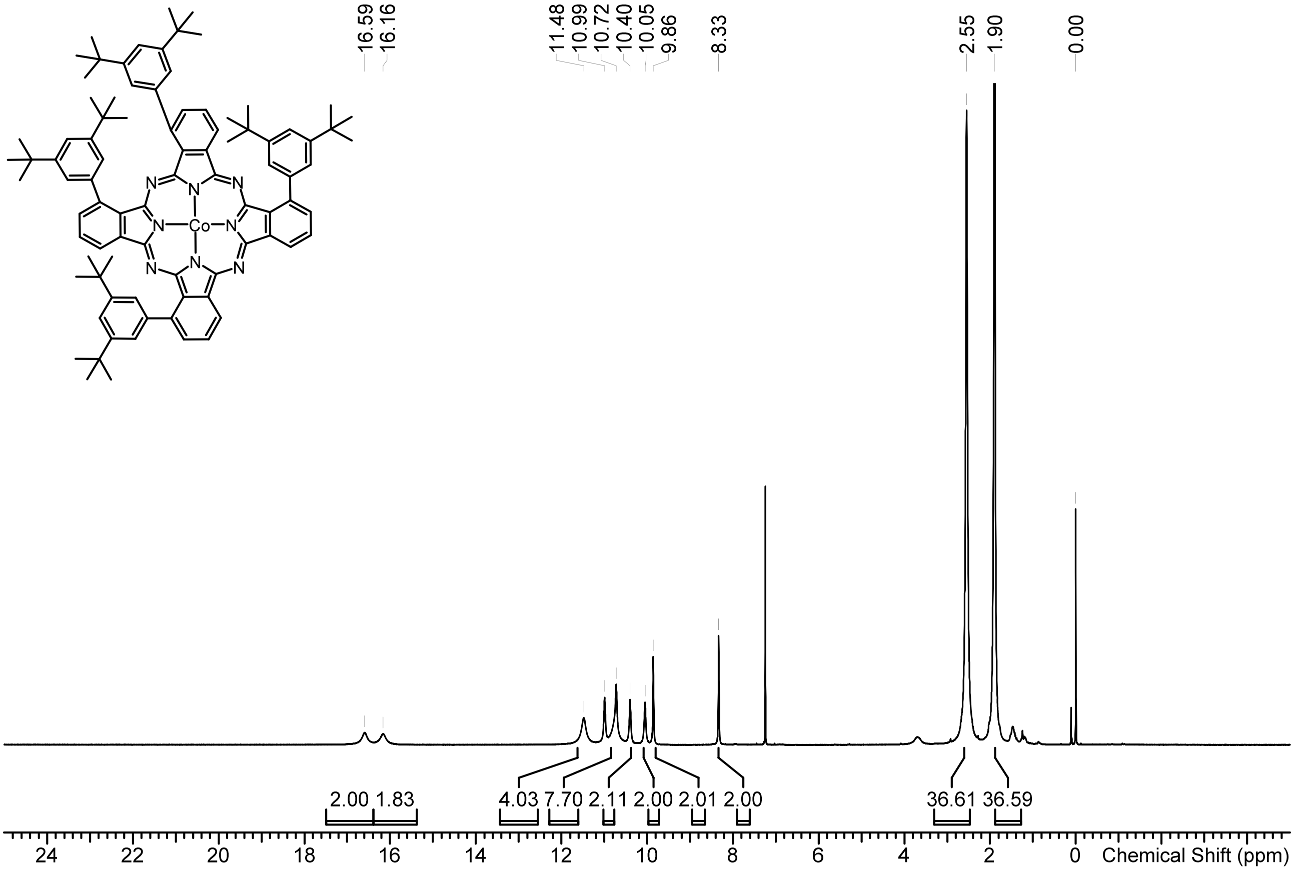

Figure S58: ^{1}H NMR spectrum (500 MHz, $CDCl_3$) of **CoPc-Frog**.

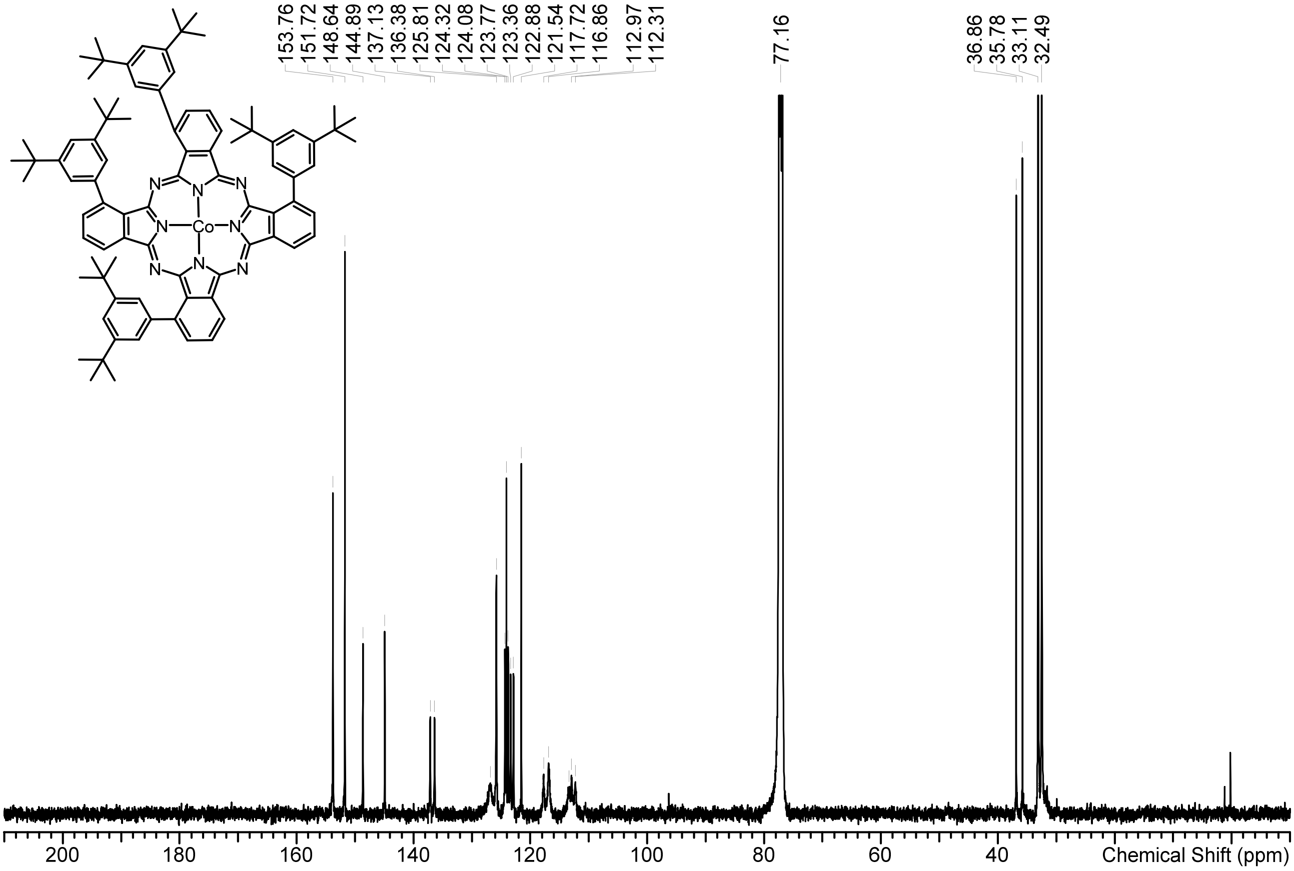

Figure S59: $^{13}C\{^1H\}$ NMR spectrum (126 MHz, $CDCl_3$) of **CoPc-Frog**.

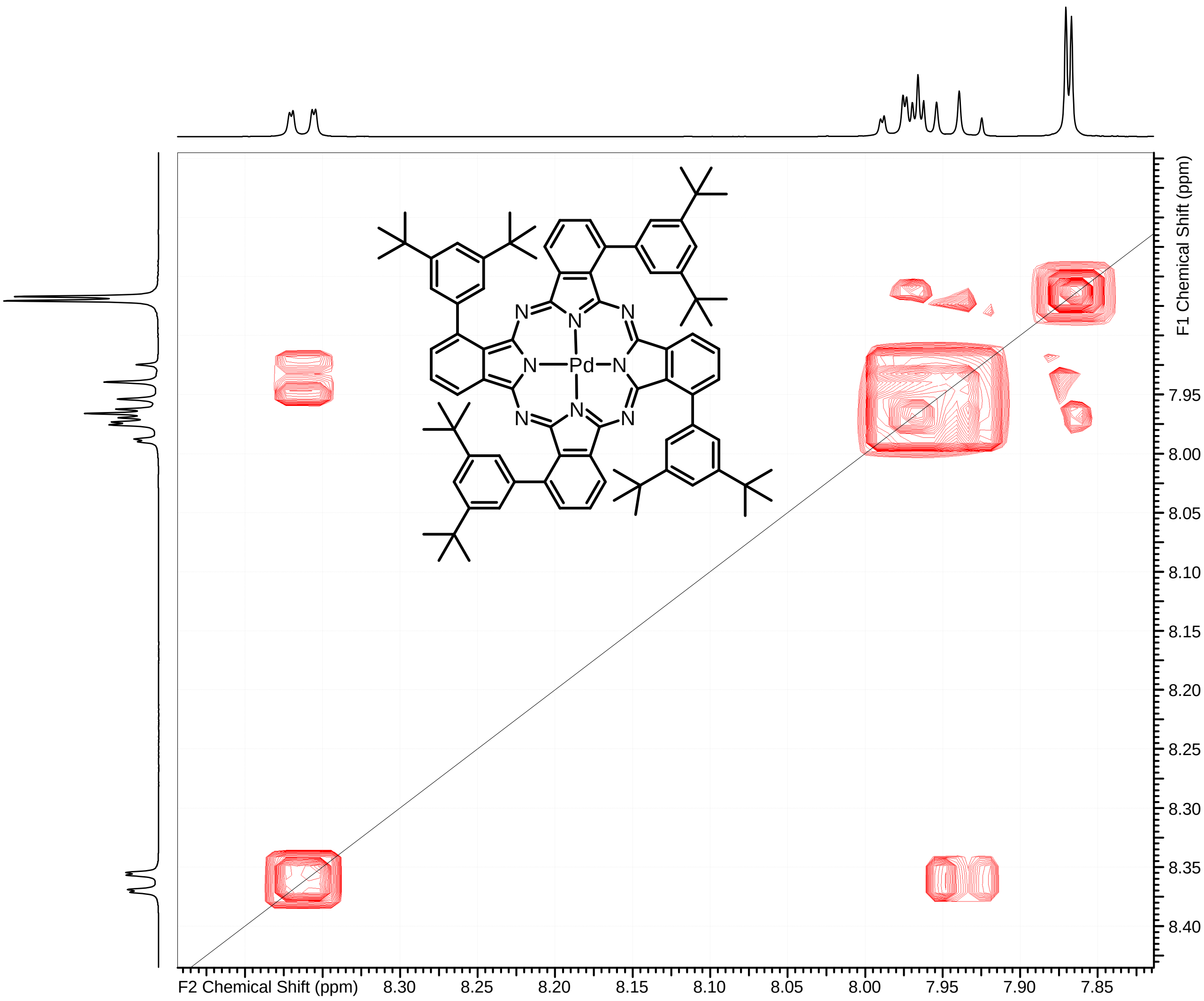

Figure S60: Aromatic region of the ^{1}H–^{1}H COSY NMR spectrum (500 MHz, $CDCl_3$) of **PdPc-Windmill**.

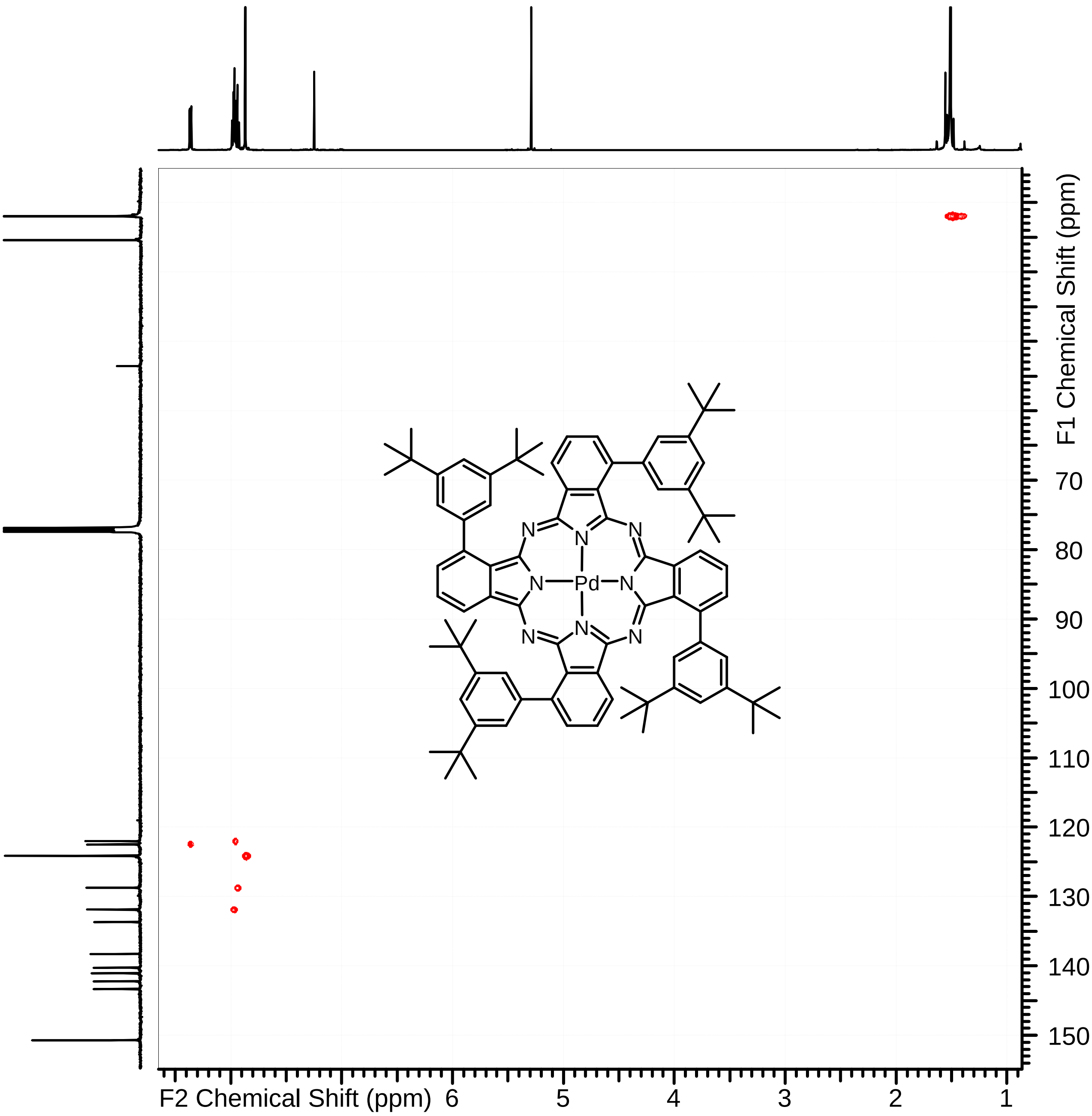

Figure S61: ^{1}H–^{13}C HSQC NMR spectrum (500 MHz, $CDCl_3$) of **PdPc-Windmill**.

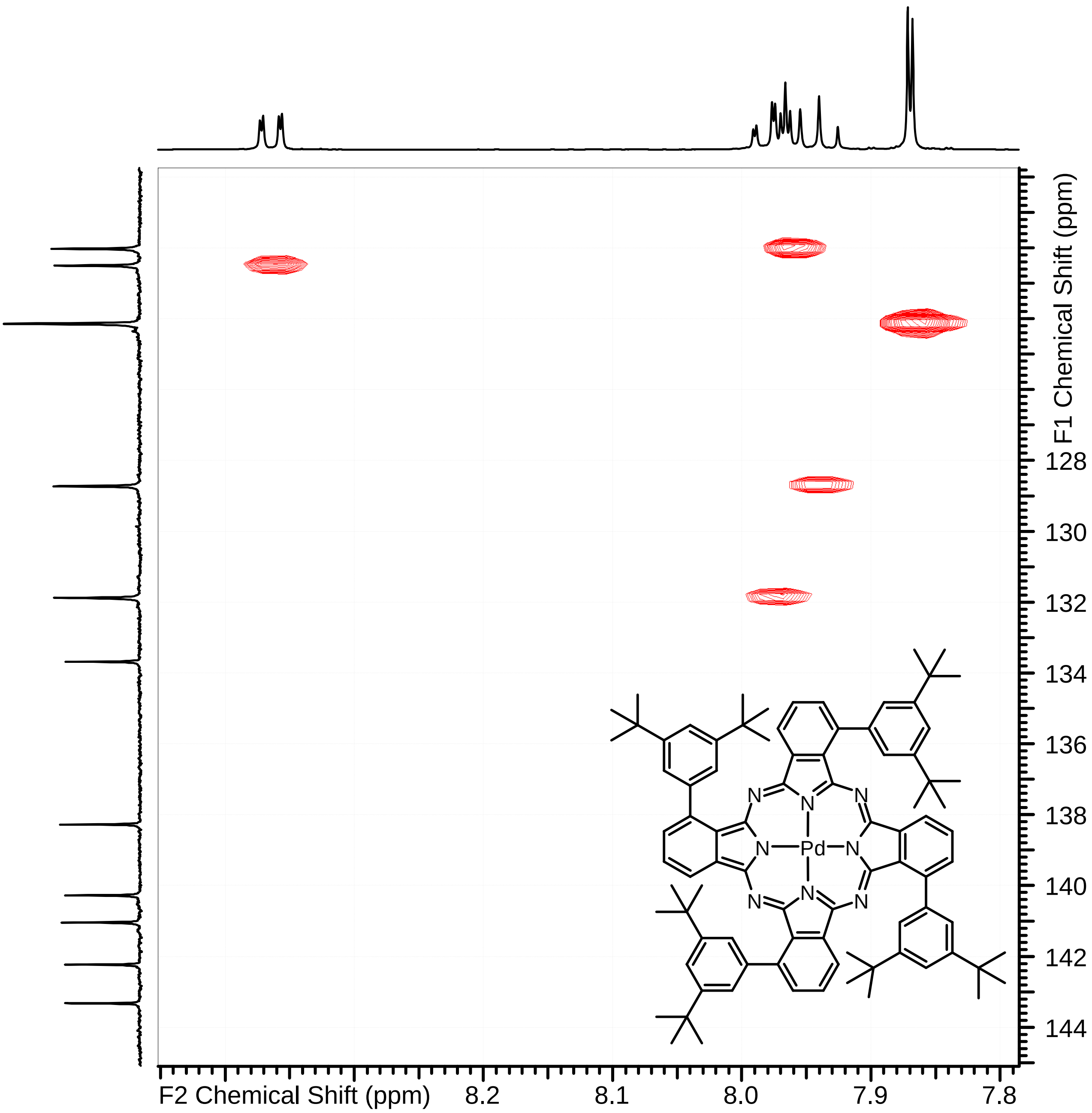

Figure S62: Aromatic region of the ^{1}H–^{13}C HSQC NMR spectrum (500 MHz, $CDCl_3$) of **PdPc-Windmill**.

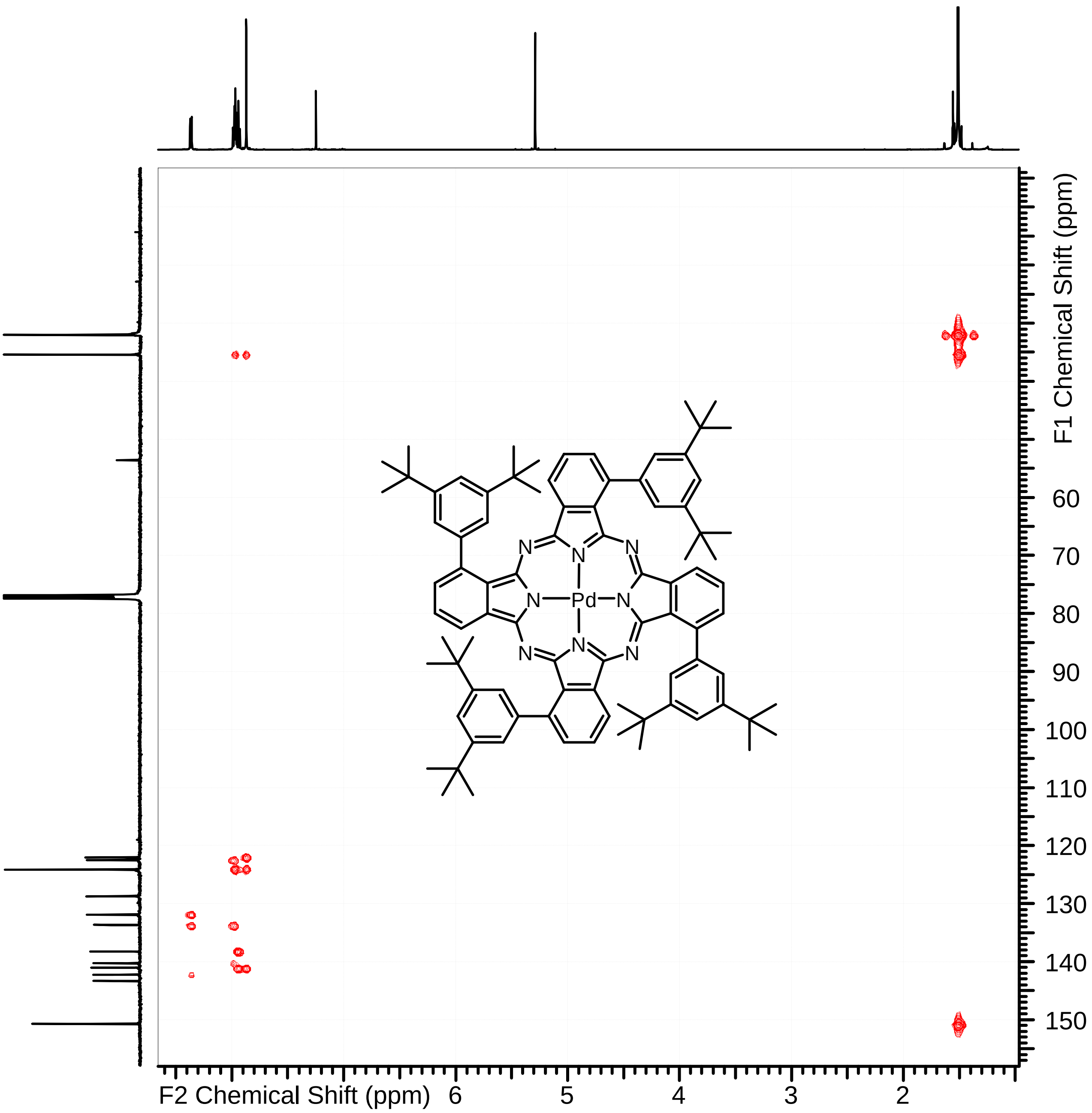

Figure S63: ^{1}H–^{13}C HMBC NMR spectrum (500 MHz, $CDCl_3$) of **PdPc-Windmill**.

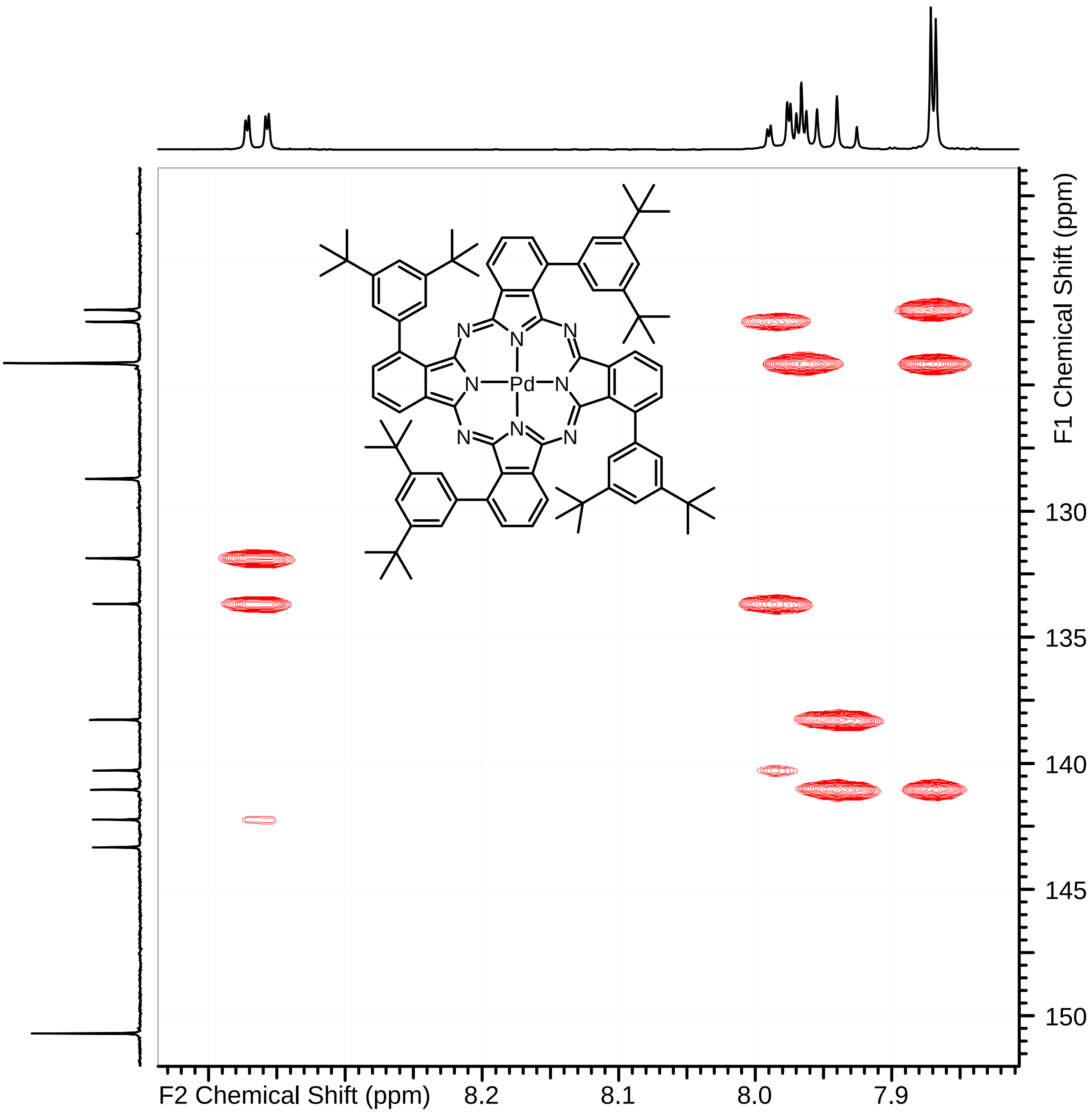

Figure S64: Aromatic region of the ^{1}H–^{13}C HMBC NMR spectrum (500 MHz, $CDCl_3$) of **PdPc-Windmill**.

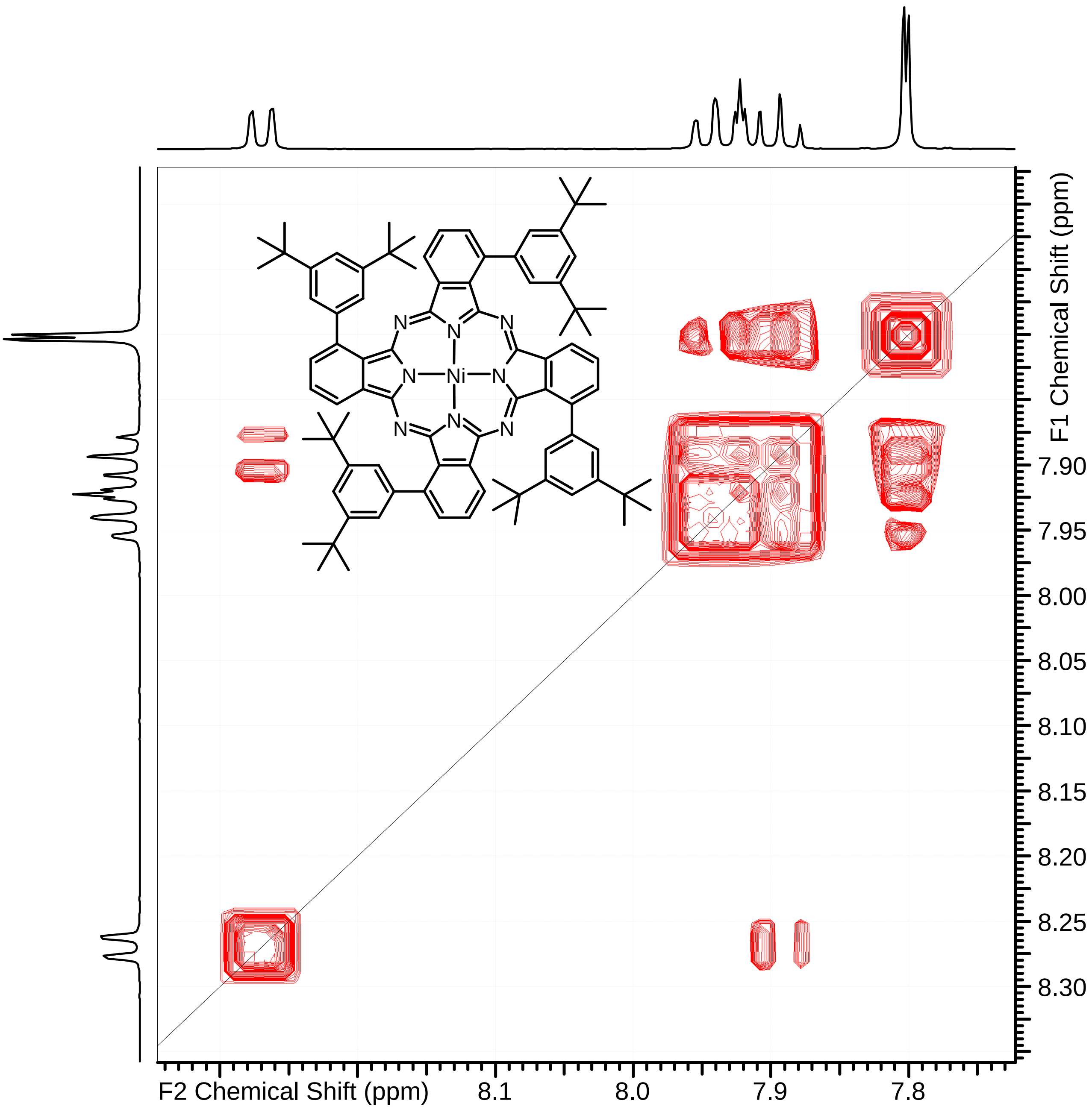

Figure S65: Aromatic region of the ^{1}H–^{1}H COSY NMR spectrum (500 MHz, $CDCl_3$) of **NiPc-Windmill**.

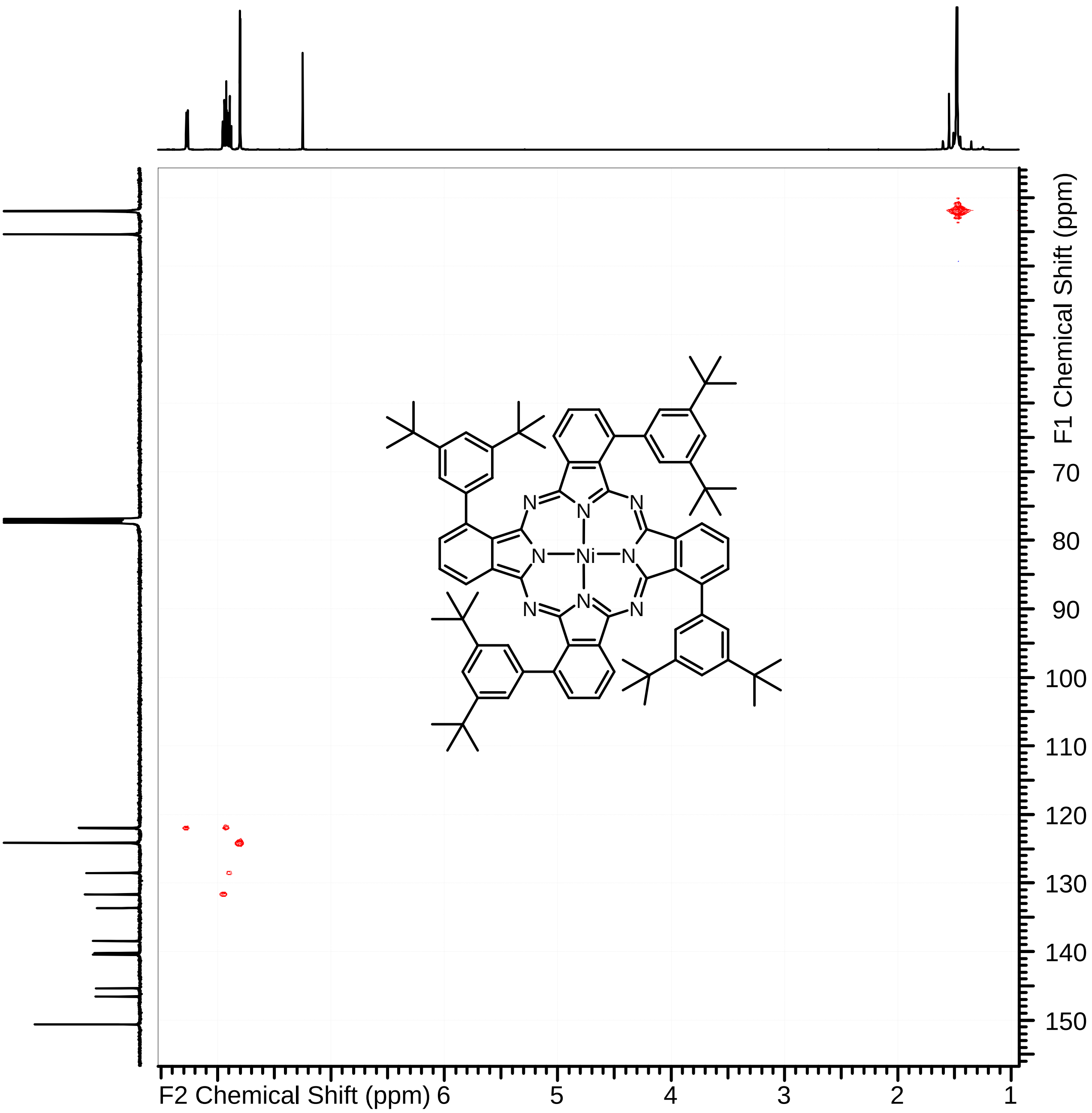

Figure S66: ^{1}H–^{13}C HSQC NMR spectrum (500 MHz, $CDCl_3$) of **NiPc-Windmill**.

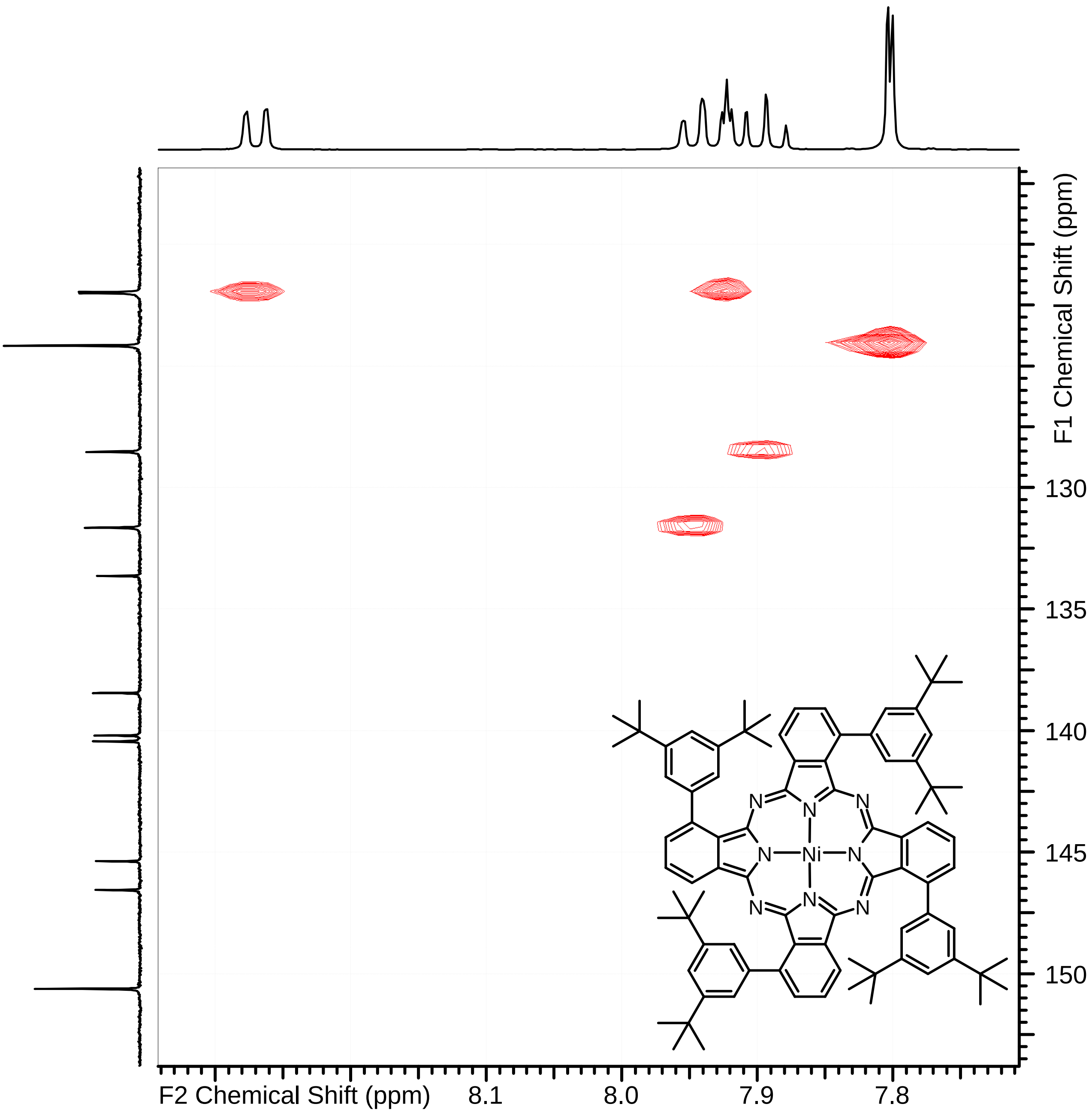

Figure S67: Aromatic region of the ^{1}H–^{13}C HSQC NMR spectrum (500 MHz, $CDCl_3$) of **NiPc-Windmill**.

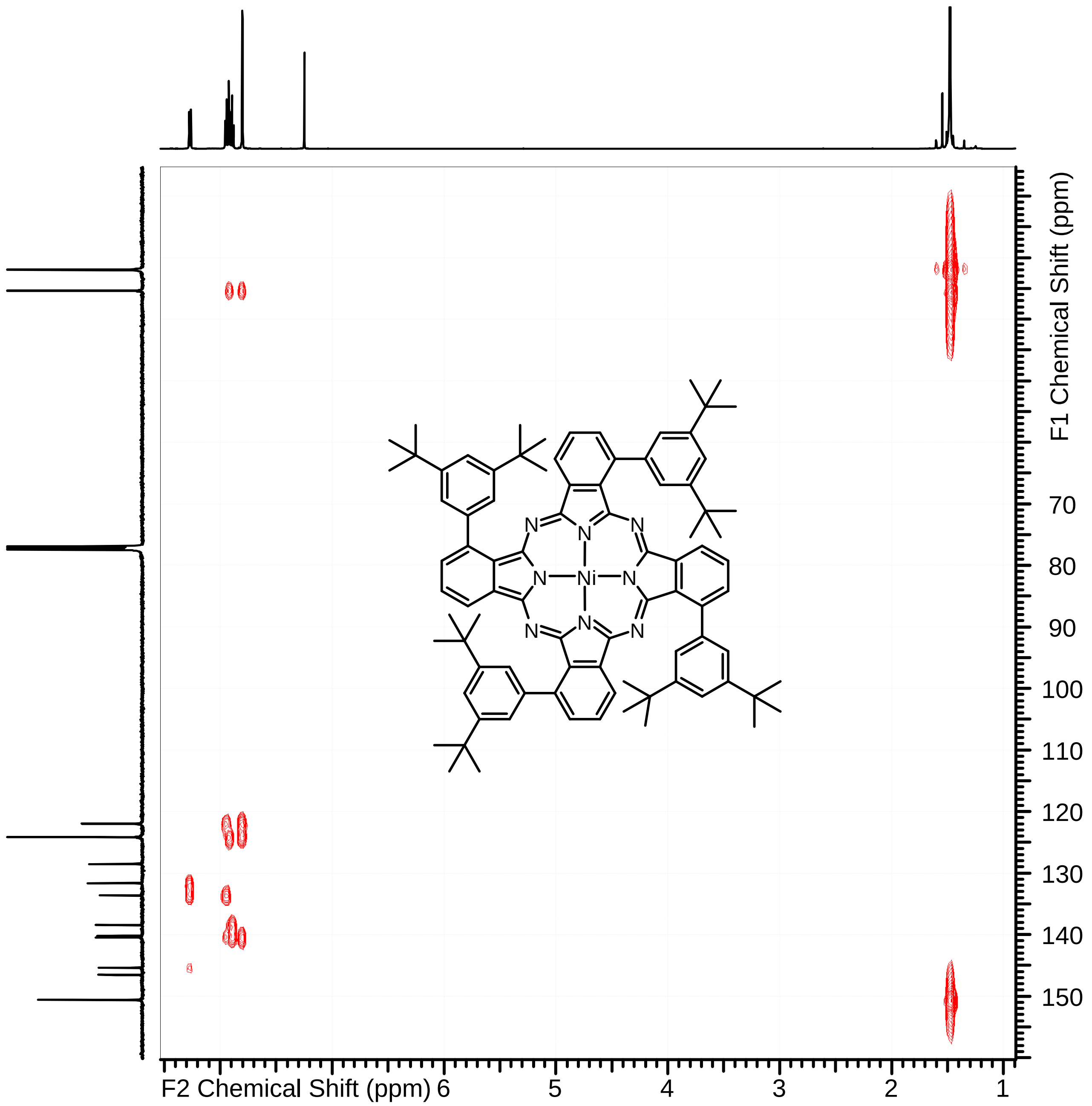

Figure S68: ^{1}H–^{13}C HMBC NMR spectrum (500 MHz, $CDCl_3$) of **NiPc-Windmill**.

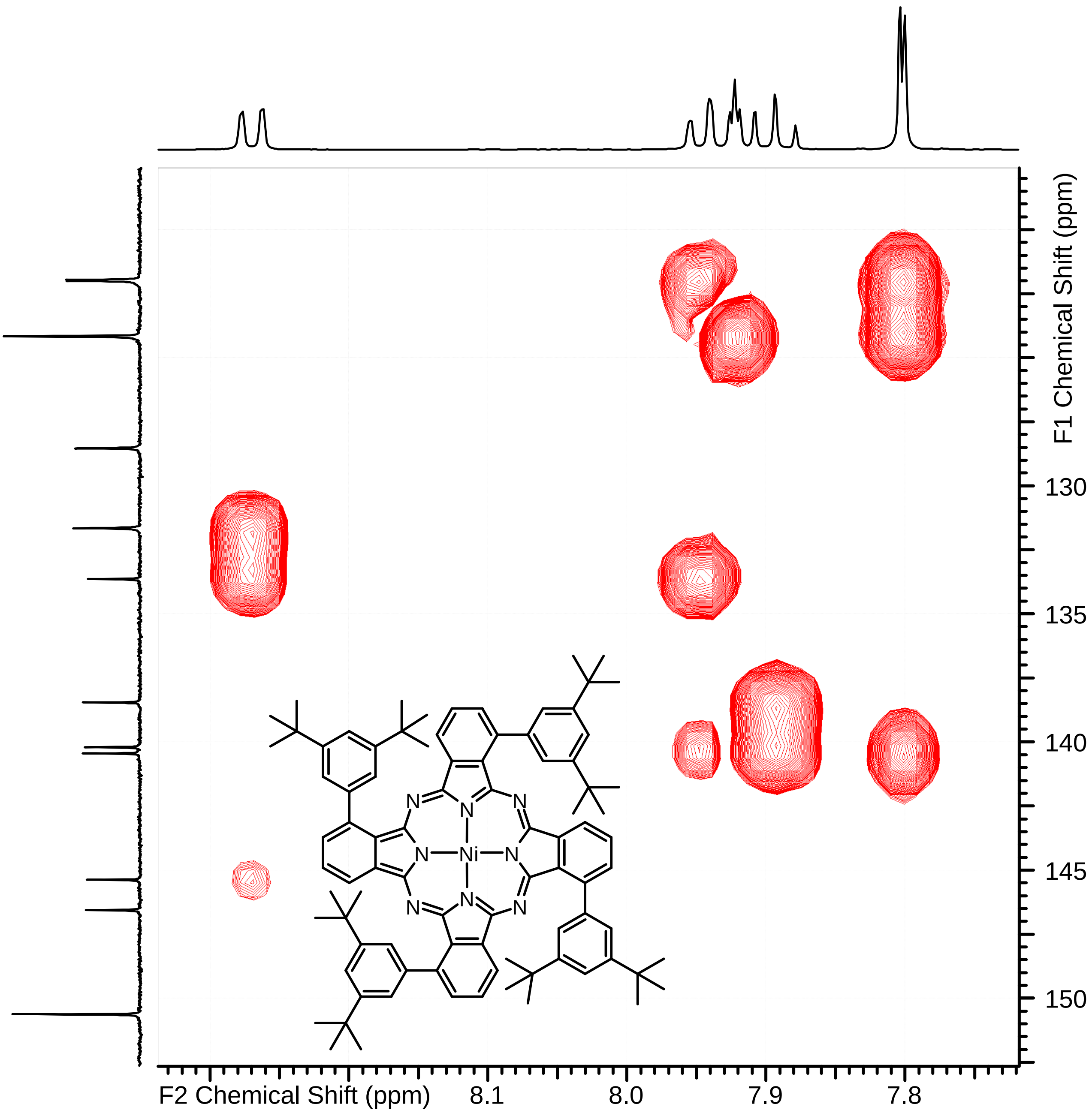

Figure S69: Aromatic region of the ^{1}H–^{13}C HMBC NMR spectrum (500 MHz, $CDCl_3$) of **NiPc-Windmill**.

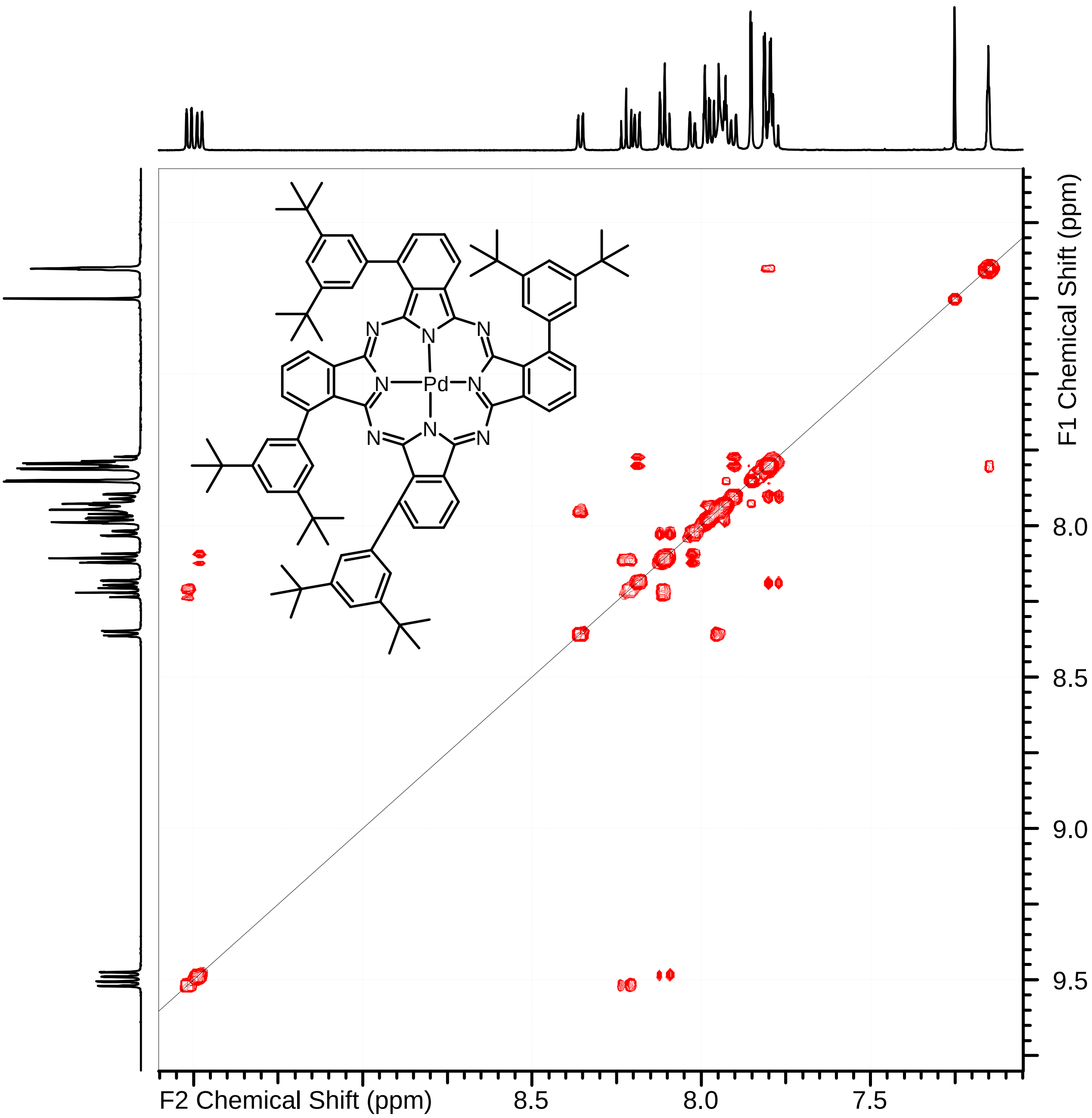

Figure S70: Aromatic region of the ^{1}H–^{1}H COSY NMR spectrum (500 MHz, $CDCl_3$) of **PdPc-Dragon**.

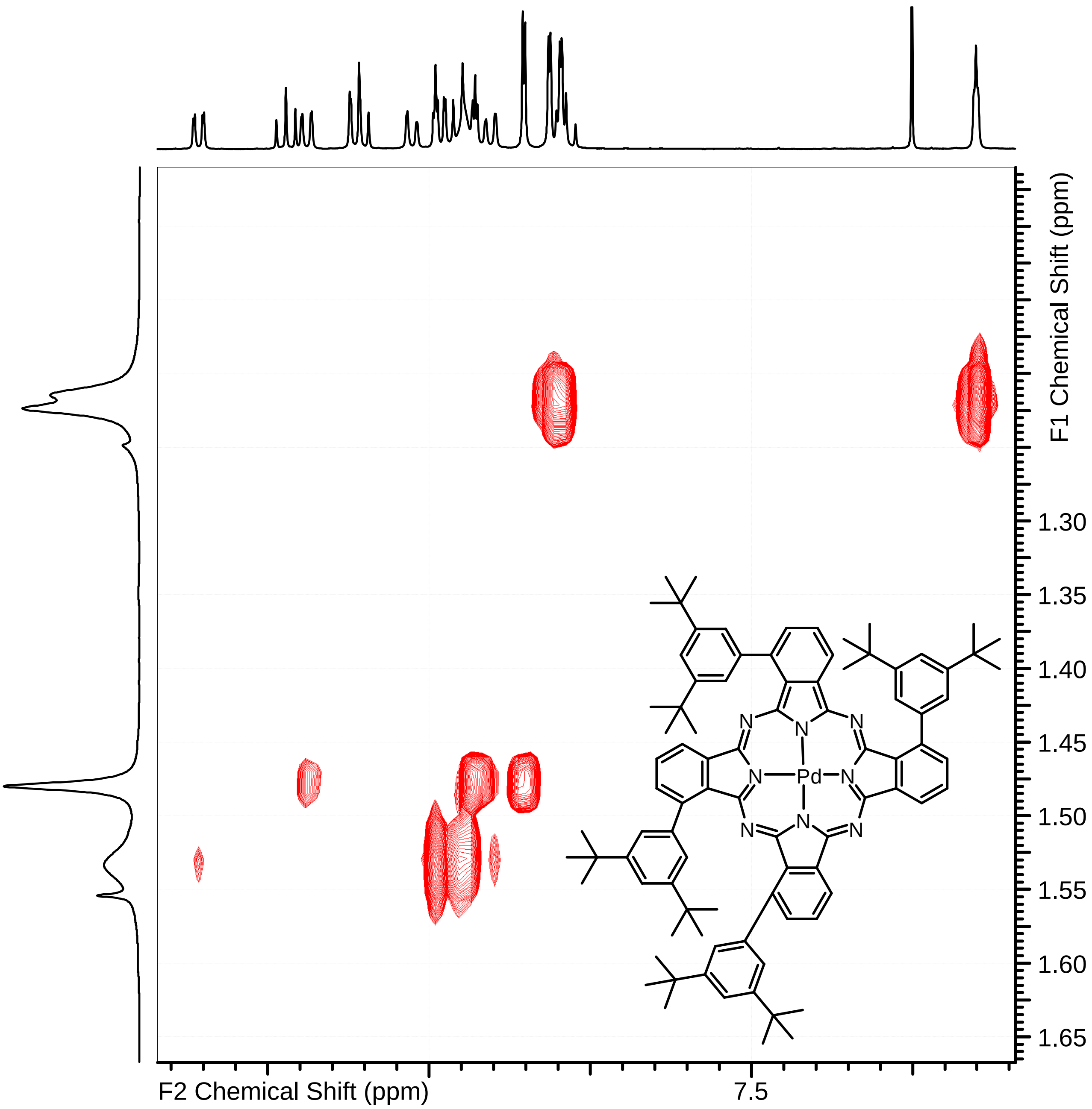

Figure S71: ^{1}H–^{1}H NOESY NMR spectrum (500 MHz, $CDCl_3$) of **PdPc-Dragon**.

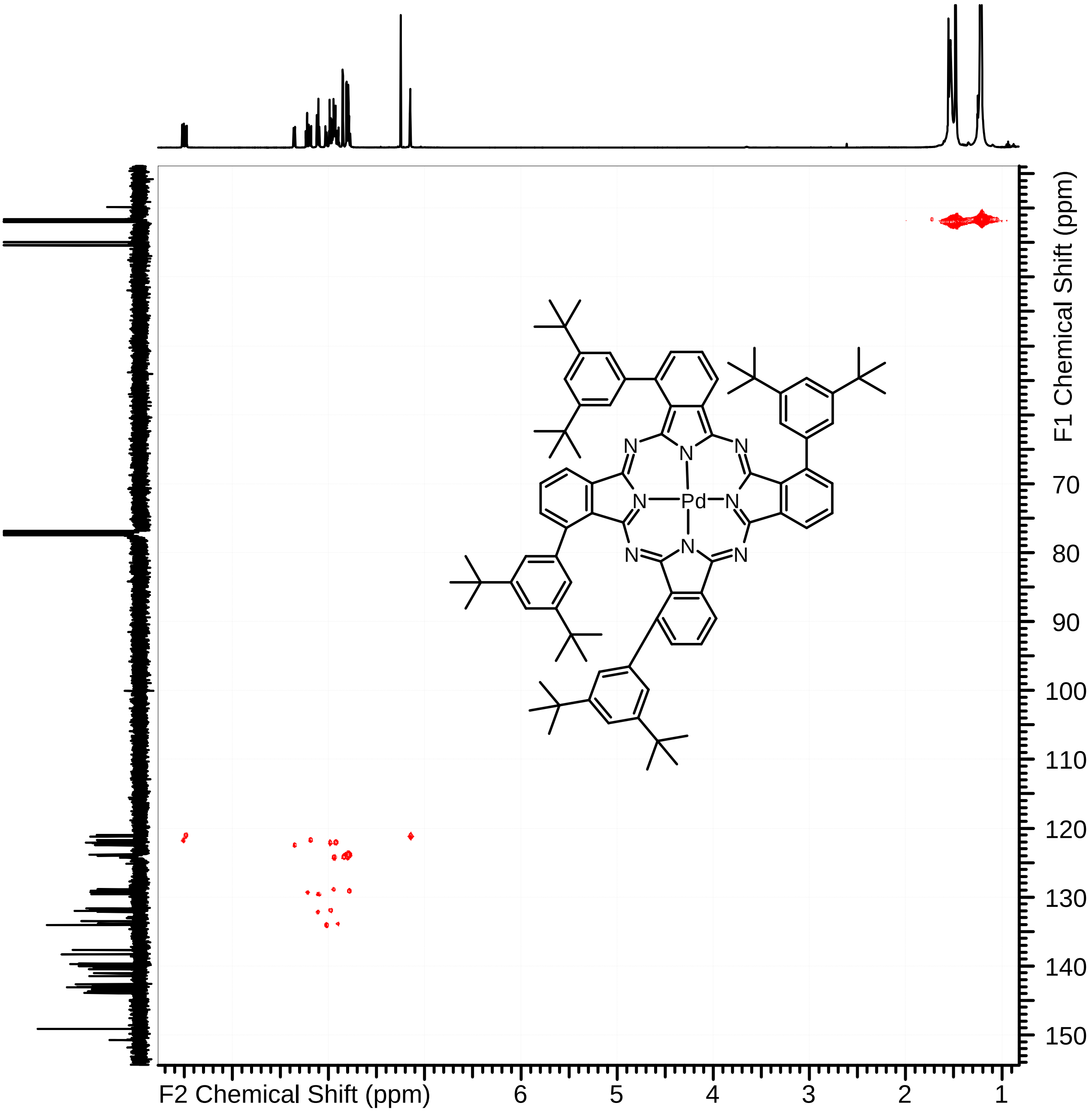

Figure S72: ^{1}H–^{13}C HSQC NMR spectrum (500 MHz, $CDCl_3$) of **PdPc-Dragon**.

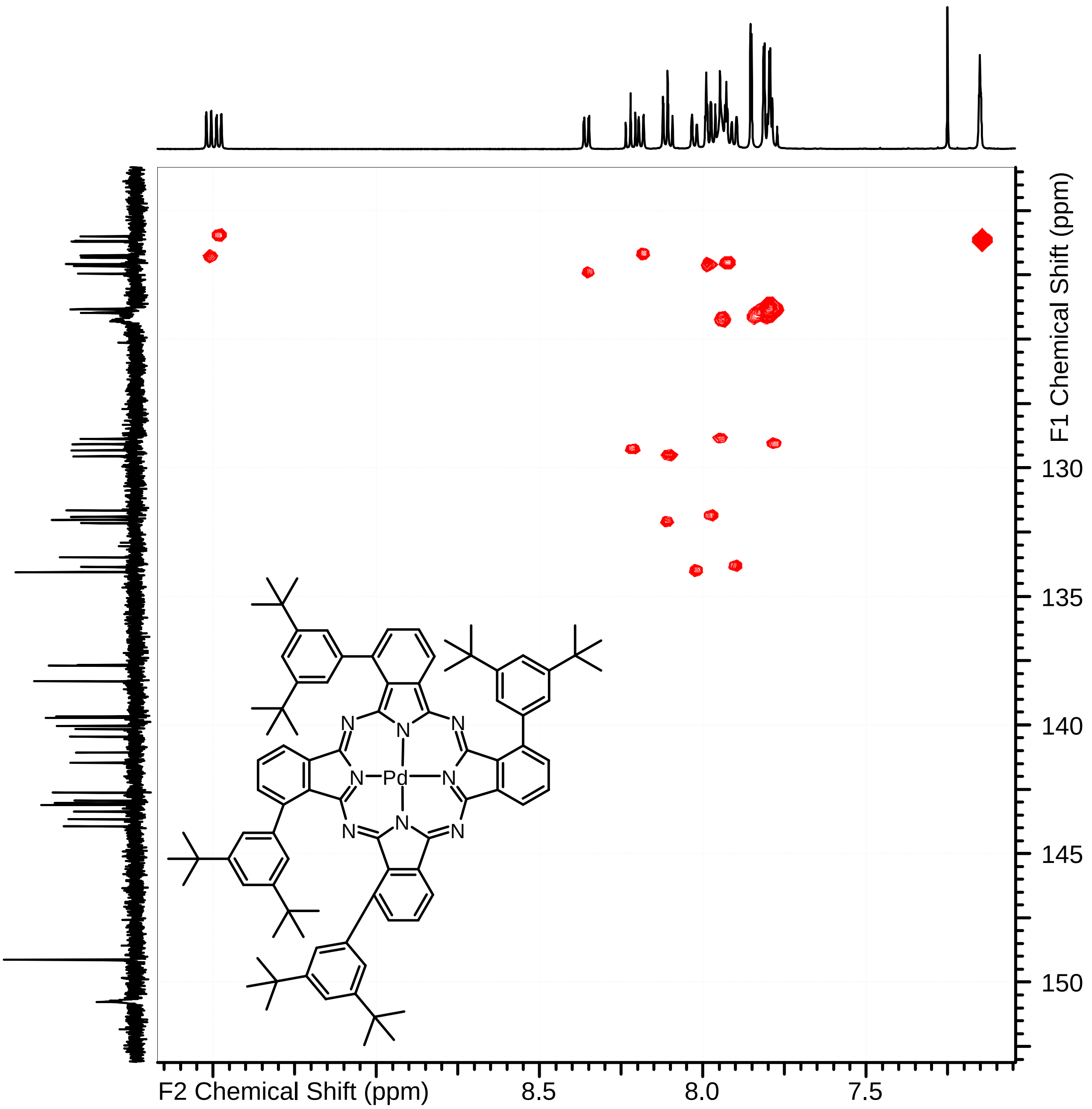

Figure S73: Aromatic region of the ^{1}H–^{13}C HSQC NMR spectrum (500 MHz, $CDCl_3$) of **PdPc-Dragon**.

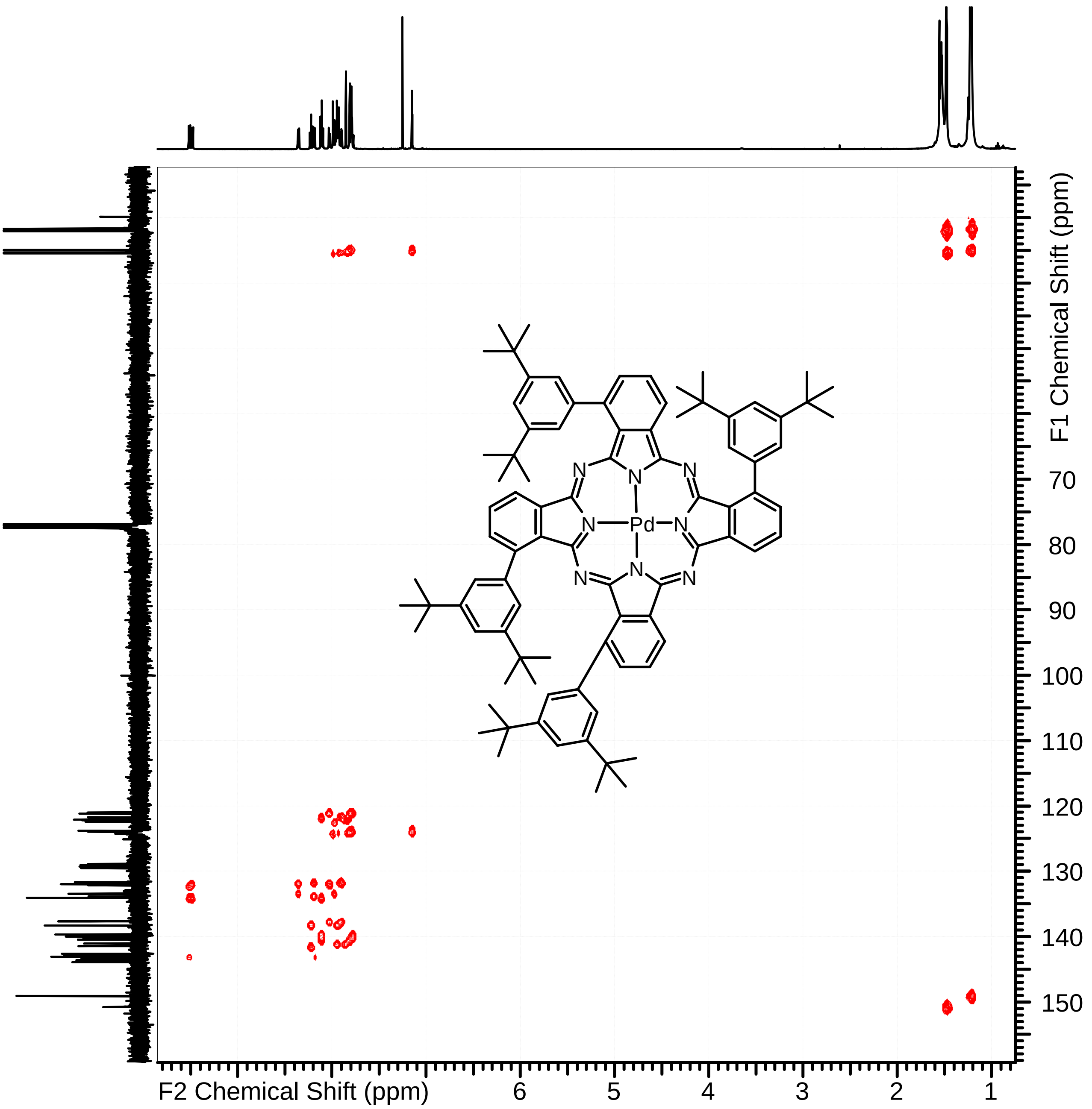

Figure S74: ^{1}H–^{13}C HMBC NMR spectrum (500 MHz, $CDCl_3$) of **PdPc-Dragon**.

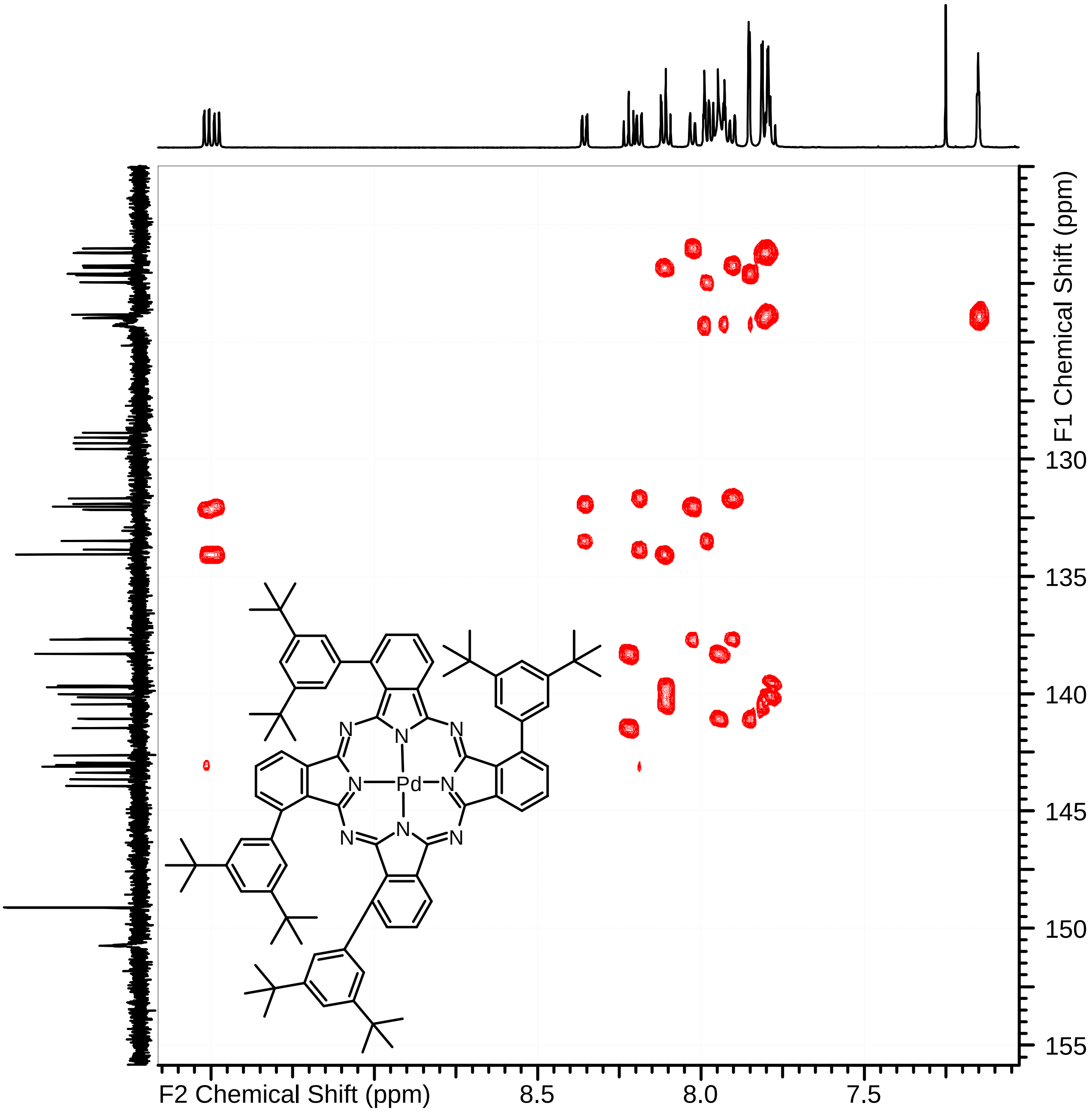

Figure S75: Aromatic region of the ^{1}H–^{13}C HMBC NMR spectrum (500 MHz, $CDCl_3$) of **PdPc-Dragon**.

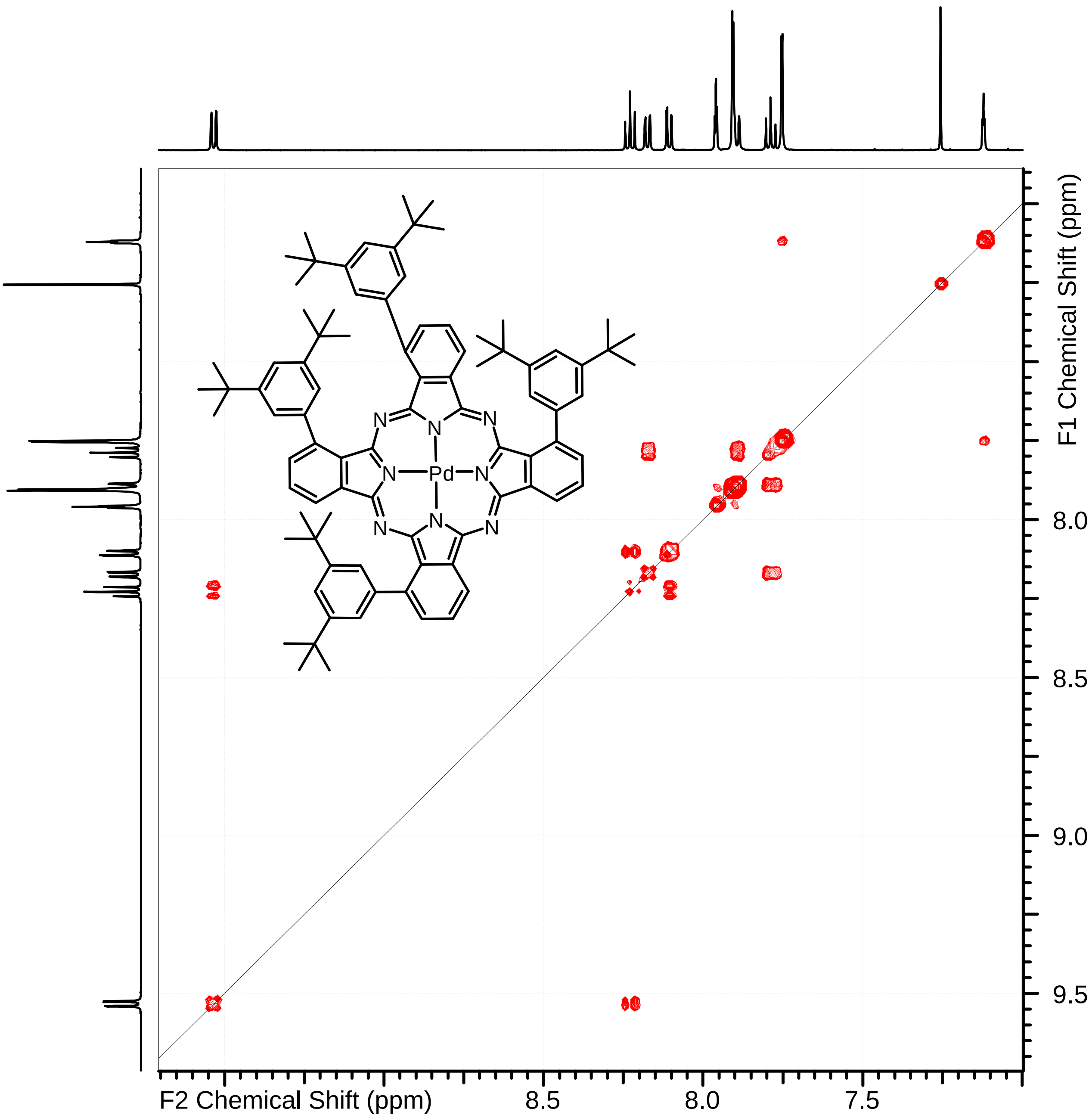

Figure S76: Aromatic region of the ^{1}H–^{1}H COSY NMR spectrum (500 MHz, $CDCl_3$) of **PdPc-Frog**.

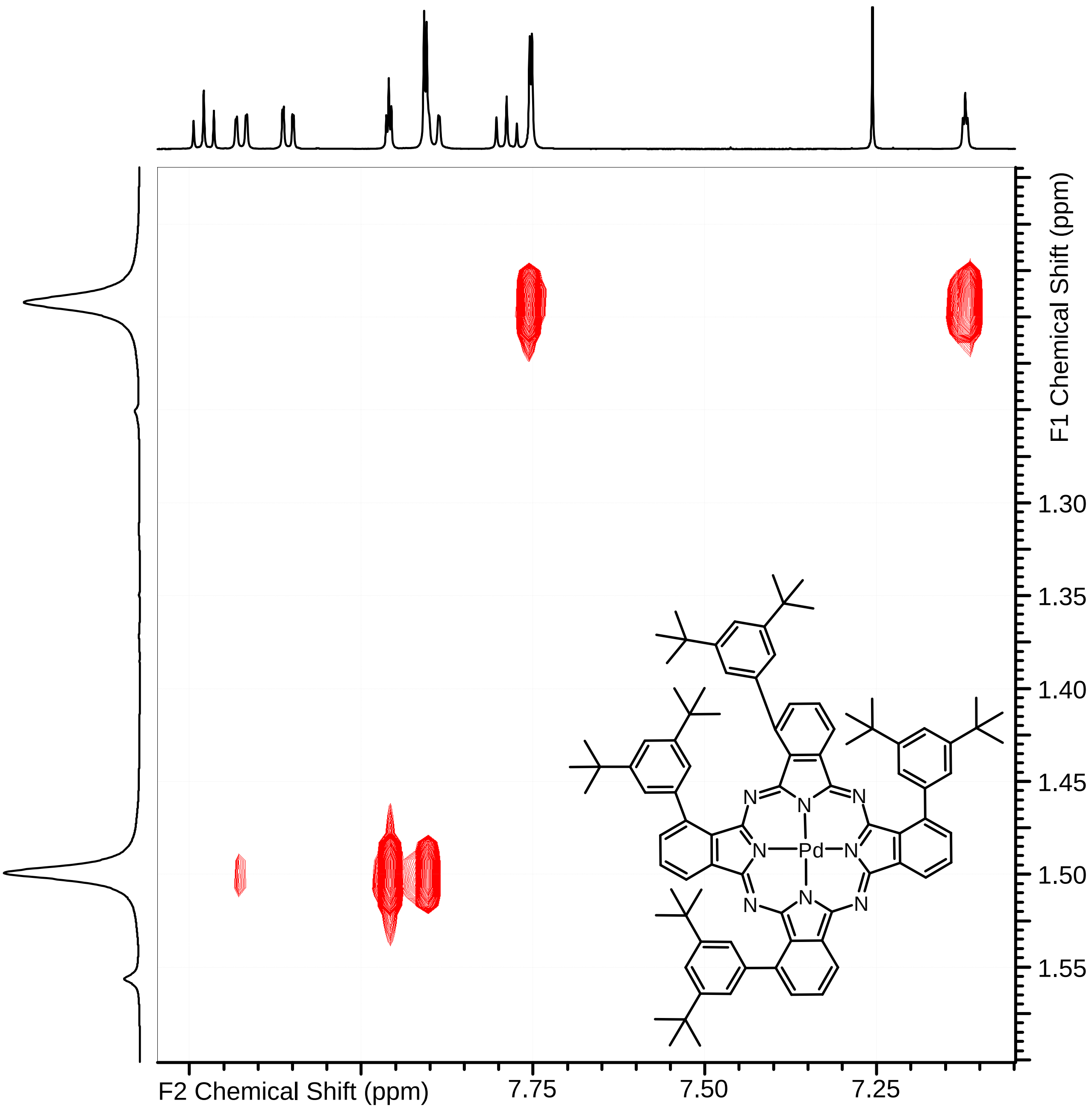

Figure S77: ^{1}H–^{1}H NOESY NMR spectrum (500 MHz, $CDCl_3$) of **PdPc-Frog**.

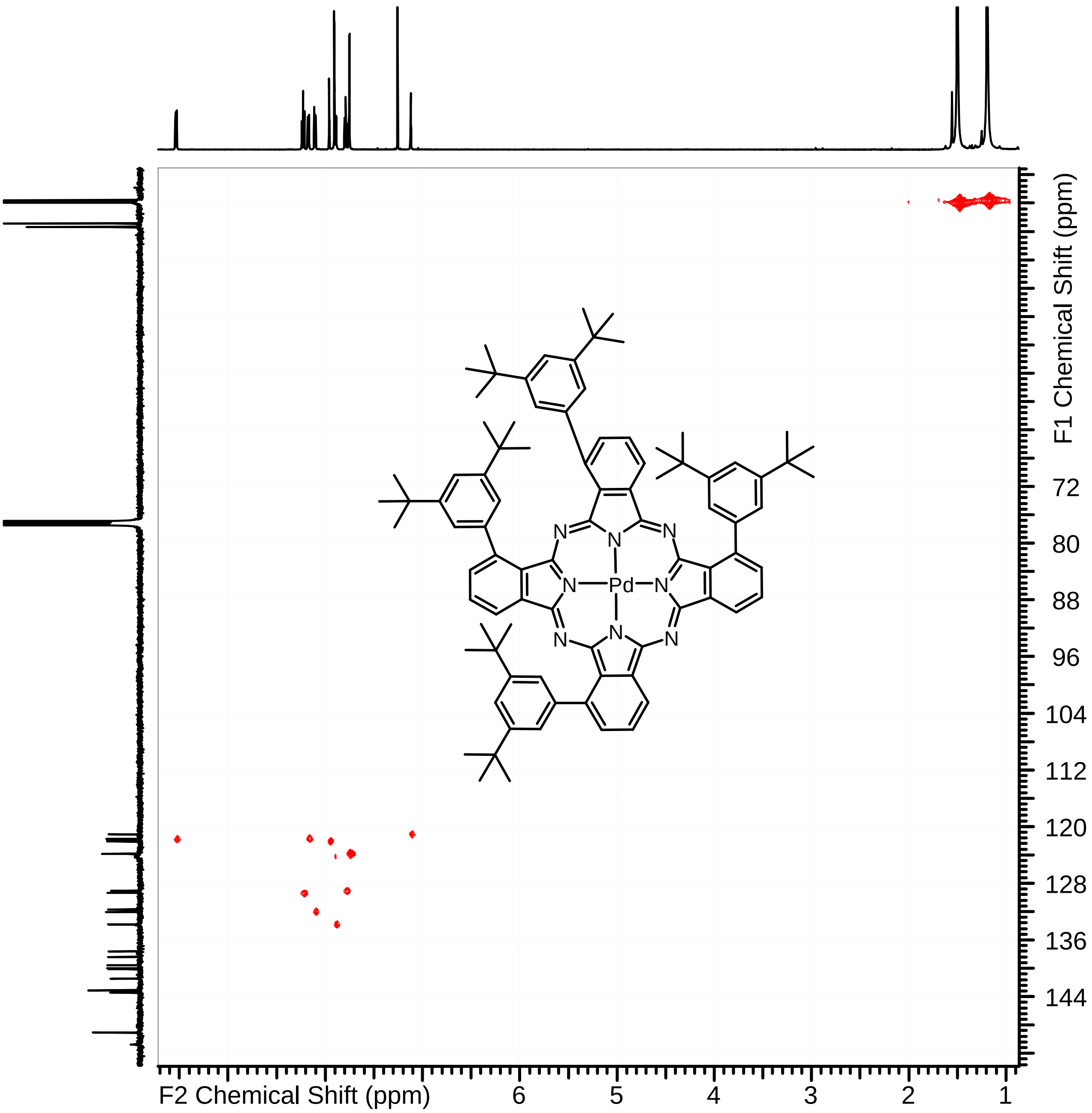

Figure S78: ^{1}H–^{13}C HSQC NMR spectrum (500 MHz, $CDCl_3$) of **PdPc-Frog**.

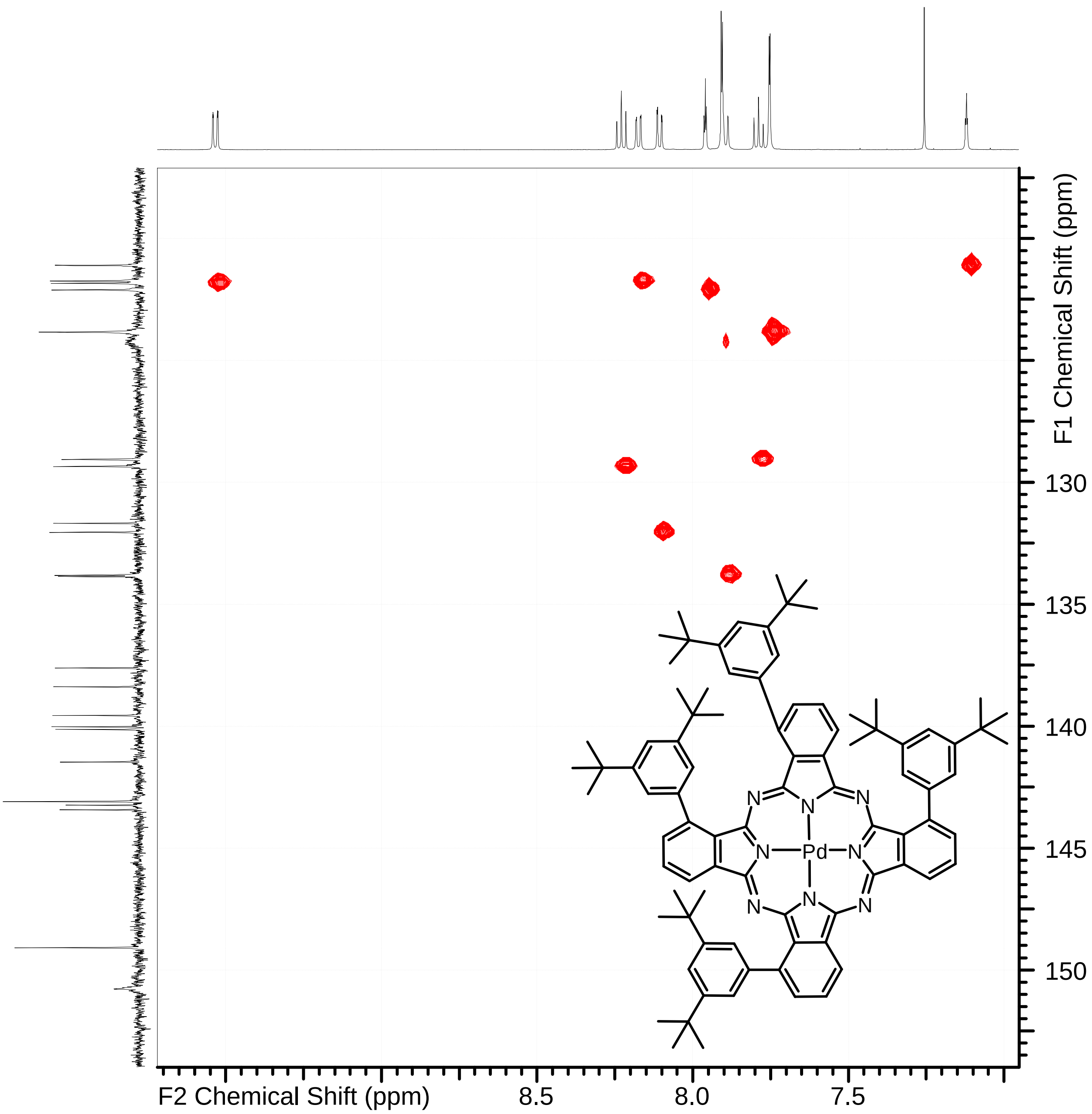

Figure S79: Aromatic region of the ^{1}H–^{13}C HSQC NMR spectrum (500 MHz, $CDCl_3$) of **PdPc-Frog**.

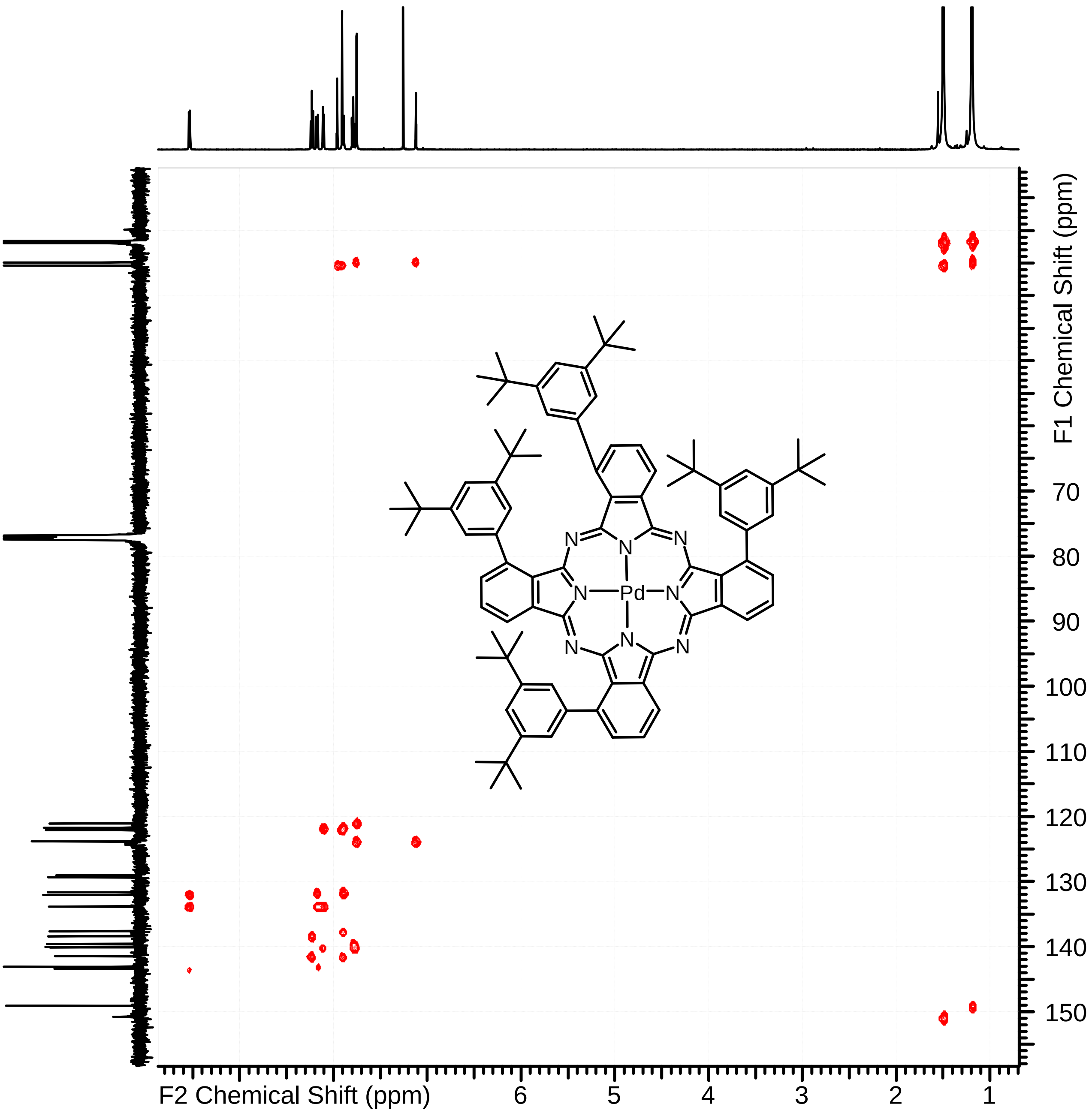

Figure S80: ^{1}H–^{13}C HMBC NMR spectrum (500 MHz, $CDCl_3$) of **PdPc-Frog**.

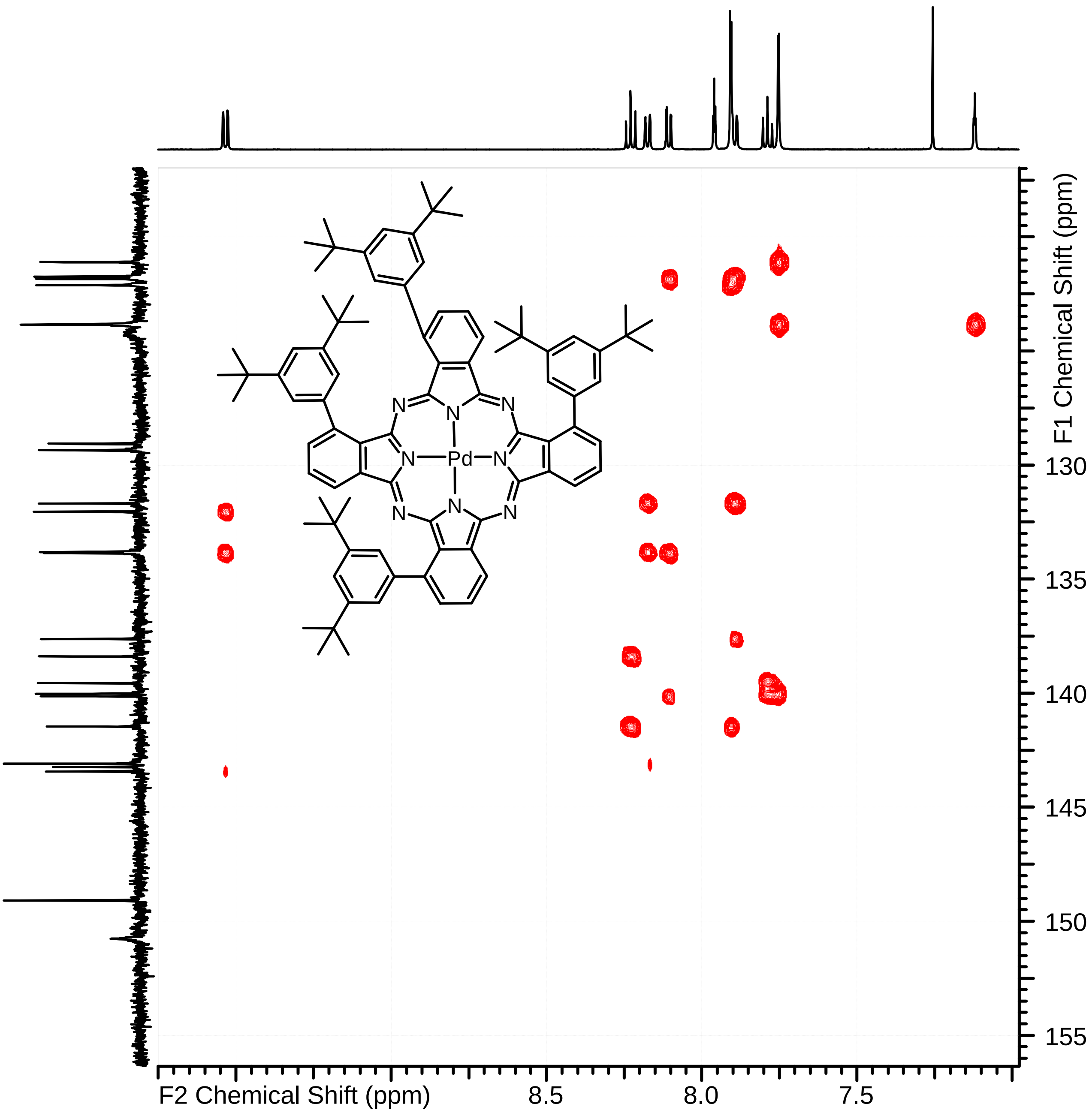

Figure S81: Aromatic region of the ^{1}H–^{13}C HMBC NMR spectrum (500 MHz, $CDCl_3$) of **PdPc-Frog**.

Crystallographic details

Non-routine refinement details

Non-hydrogen-atoms were refined with anisotropic displacement parameters. All carbon-bound hydrogen atoms were refined isotropically at calculated positions using a riding model with their U_{iso} values constrained to 1.2 times U_{eq} of their pivot atoms for aromatic or methylene hydrogen atoms and to 1.5 times U_{eq} for methyl hydrogen atoms.

The N-bound hydrogen atoms in **H_2Pc-Windmill** were refined freely with regard to location but their U_{iso} values were constrained to 1.2 times U_{eq} of their parent nitrogen atoms. The structure has three modelled/refined disorders: i) the N-bound hydrogen atoms appear on all isoindole nitrogen atoms with 33% on N1/N1A and with 67% on N3/N3A. ii) One ^{t}Bu-group (C18-C21) has a rotational disorder which was modelled with SIMU and DELU constraints at slightly harshened values. Occupancies are 55% and 45%. iii) One chloroform molecule, which could be refined, is disordered into slightly different orientations. This was modelled with SAME, SIMU and DELU, the latter two again at harshened values. Occupancies are 53% and 47%. Substantial amounts of further solvent appear as diffuse electron density smeared along channel-like voids. The respective electron density was removed with PLATON/SQUEEZE[10] resulting in 201 electrons (roughly 3.5 chloroform molecules per unit cell; Z = 1) and a total void size of 584 Å^3. The latter issue is very similar in the case of **NiPc-Windmill** with 3.6 chloroform molecules per formula, 92 electrons per unit cell and a total void size of 549 Å^3. One chloroform molecule could be refined, albeit as entirely disordered. This was modelled using DFIX commands for all C-Cl and Cl-Cl distances in addition to SIMU and DELU constraints at defaults. Occupancies are 54% and 46%. One ^{t}Bu-group (C22-C24) is also disordered which was modelled with SAME, SIMU and DELU constraints at default values. Occupancies are 55% and 45%. Two reflexes are omitted as clear outliers.

The crystal of **[CoPc-Dragon(py)$_2$]** diffracted comparably weakly. In the structure, co-crystallized solvent is only present as diffuse electron density smeared along the crystallographic channel-like voids. This electron density was removed using PLATON/SQUEEZE. The program finds 373 electrons in a total (gigantic) void size of 1276 Å^3. Z is 4 and this equals ca. 1.6 chloroform molecules per formula and 6.4 per unit cell. The complex is coordinated by pyridine at the two apical positions, of which one (N10 to C98) is disordered completely. The major component (74% occupancy) coordinates as a typical Lewis base via nitrogen. The minor component (26%) binds the cobalt atom with its π-system, i.e. both pyridine components are perpendicular to each other. The disorder was modelled with SAME, SIMU, and DELU constraints, the latter two with harshened values. In addition, the minor component was forced into a regular hexagon using AFIX. The macrocyclic ligand has several disorders, one of which is extensive. In one arm, the entire bis-^{t}Bu-C_6H_3 moiety is disordered by a slide (C31 to C44). The bond angles/geometries relative to the rest of the ligand are not all entirely chemically reasonable but further modelling did not improve the final refinement. The disorder was modelled with SADI for the bonds of C31/C31' to the anchor atom C27. Further constraints are SAME, SIMU and DELU, all at default values. Occupancies are 53% and 47%. Two other ^{t}Bu groups are disordered by a typical rotation which was modelled in both cases with SAME, SIMU and DELU at default values. Occupancies are 74% versus 26% for C16 to C18 and 76% versus 24% for C86 to C88. Experimental parameters are summarized in Table S2.

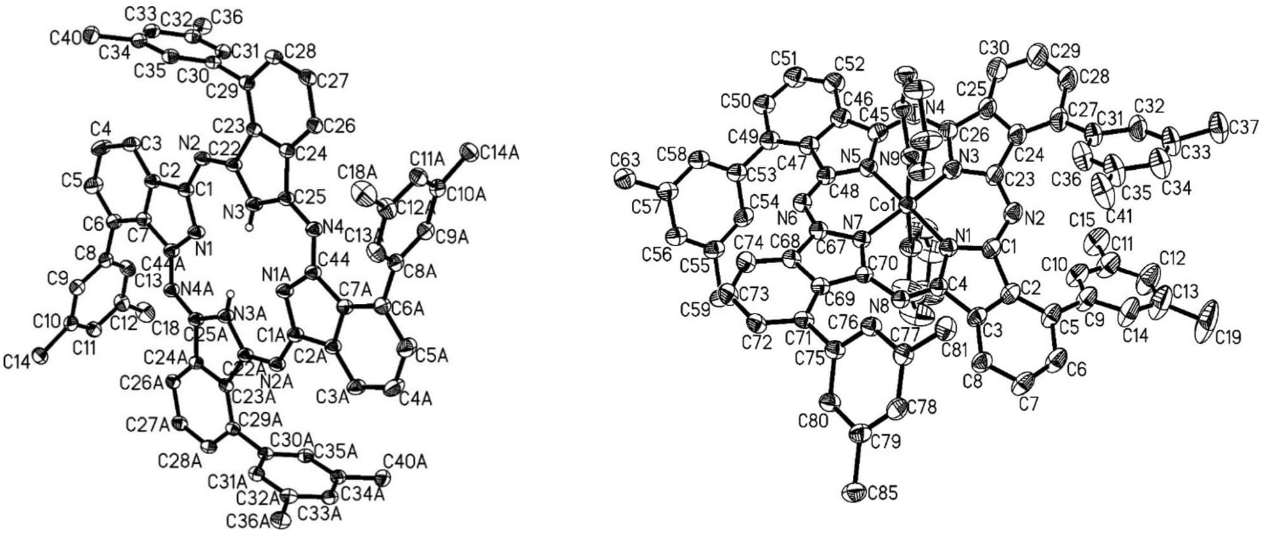

Figure S82: Molecular structures of **H_2Pc-Windmill** (**Left**; R_{int} = 4.04%) and the major component of **[CoPc-Dragon(py)$_2$]** (**Right**; R_{int} = 9.10%). Displacement ellipsoids are shown at the 50% (**Left**) and 30% (**Right**) probability levels, and hydrogen atoms, *tert*-butyl groups as well as solvent molecules are omitted for clarity. **Right:** The four isoindole rings are labelled for data evaluation: Ring **A** – N7-C67-C68-C69-C70; Ring **B** – N1-C1-C2-C3-C4; Ring **C** – N3-C23-C24-C25-C26; Ring **D** – N5-C45-C46-C47-C48.

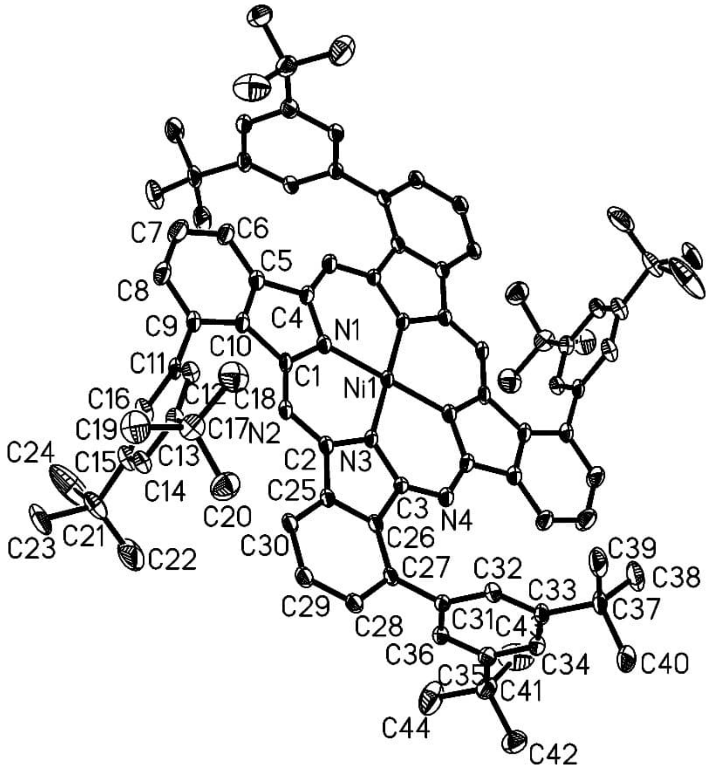

Figure S83: Molecular structure of **NiPc-Windmill** (R_{int} = 7.64%). Displacement ellipsoids are shown at the 50% probability level, and hydrogen atoms as well as solvent molecules are omitted for clarity.

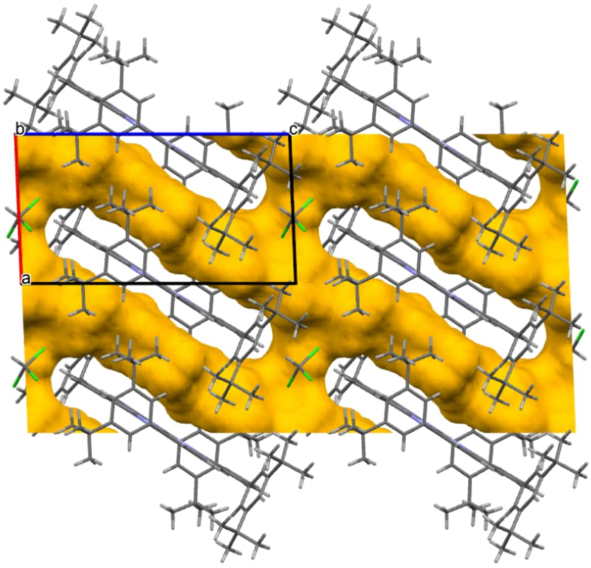

Figure S84: Visualization of the solvent-filled channels (orange) in between the stacked phthalocyanine molecules (here **H_2Pc-Windmill**) as well as the stacked columns along the crystallographic axis b. Picture created with Mercury.

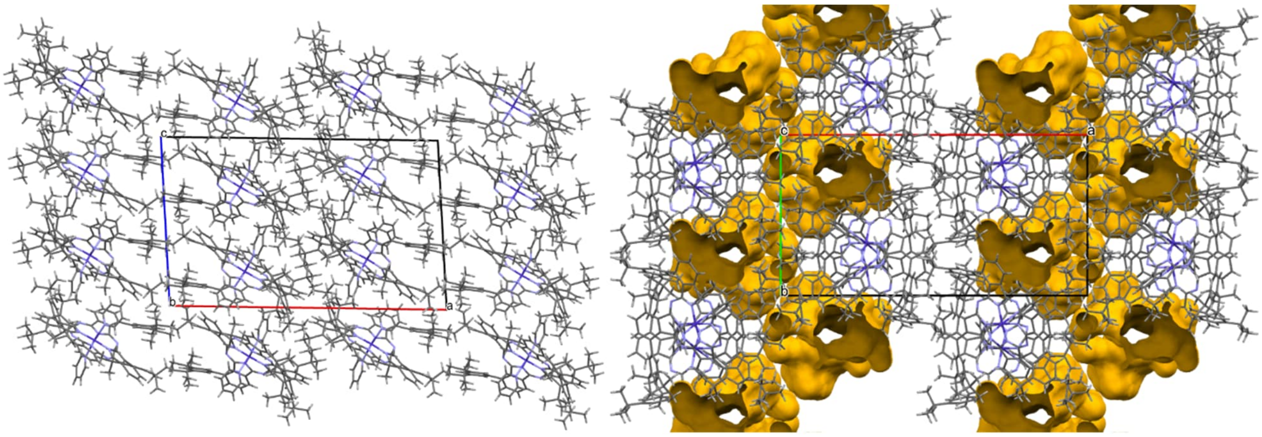

Figure S85: Visualization of the crystal lattice from **[CoPc-dragon(py)$_2$]** with columnar stacks of the molecules along crystallographic axis c (**Left**, view along axis b) and the solvent-filled interspace in between the columns (**Right**, view along axis c and solvent visualized in orange). Picture created with Mercury

Table S2: Crystallographic data.

	H_2Pc-Windmill	NiPc-Windmill	[CoPc-Dragon(py)$_2$]
CCDC number	2540123	2540124	2540125
Crystal data			
Chemical formula	$C_{88}H_{98}N_8 \cdot 2(CHCl_3)[+3.5(CHCl_3)]$	$C_{88}H_{96}N_8Ni \cdot 2(CHCl_3)[+3.6(CHCl_3)]$	$C_{98}H_{106}CoN_{10}[+1.6CHCl_3]$
M_r	1506.47	1563.17	1482.85
Crystal system, space group	Triclinic, $P\bar{1}$	Triclinic, $P\bar{1}$	Monoclinic, $P2_1/c$
Temperature (K)	121	122	120
a, *b*, *c* (Å)	9.1346 (1), 16.5089 (3), 17.1896 (3)	9.1452 (2), 16.4727 (4), 17.1941 (4)	30.8527 (10), 16.1188 (3), 18.8367 (3)
α, β, γ (°)	74.833 (2), 85.774 (1), 82.337 (1)	74.601 (2), 85.688 (2), 82.464 (2)	90, 93.802 (2), 90
V (Å^3)	2477.55 (7)	2473.46 (10)	9347.0 (4)
Z	1	1	4
Radiation type	Cu *K*a	Cu *K*a	Cu *K*a
μ (mm^{-1})	1.90	2.09	1.80
Crystal size (mm)	0.29 × 0.17 × 0.17	0.32 × 0.13 × 0.09	0.12 × 0.06 × 0.02
Data collection			
Diffractometer	XtaLAB Synergy, Single source at home/near, HyPix	XtaLAB Synergy, Single source at home/near, HyPix	XtaLAB Synergy, Single source at home/near, HyPix
Absorption correction	Gaussian/Numerical face indexed	Gaussian/Numerical face indexed	Gaussian/Numerical face indexed
T_{min}, T_{max}	0.356, 1.000	0.47, 1.19	0.656, 1.000
No. of measured, independent and observed [I > 2s(I)] reflections	49447, 10561, 9531	48170, 9982, 8979	77189, 16486, 9863 {I > 2s(I)}
R_{int}	0.040	0.076	0.091
(sin q/l)$_{max}$ (Å^{-1})	0.639	0.625	0.595
Refinement			
$R[F^2 > 2s(F^2)]$, $wR(F^2)$, S	0.057, 0.163, 1.05	0.086, 0.256, 1.06	0.069, 0.211, 1.03
No. of reflections	10561	9982	16486
No. of parameters	556	555	1244
No. of restraints	246	258	1585
H-atom treatment	H atoms treated by a mixture of independent and constrained refinement	H-atom parameters constrained	H-atom parameters constrained

$\Delta\rho_{max}$, $\Delta\rho_{min}$ (e Å^{-3})	0.40, -0.34	1.22, -1.03	0.39, -0.47

Table S3: Geometric Parameters (Å,°) for **H_2Pc-Windmill**.

**Bond lengths**			
N(1)–C(1)	1.363(2)	C(18)–C(20)	1.610(6)
N(1)–C(44)^{i}	1.373(2)	C(18)–C(21')	1.611(7)
N(2)–C(22)	1.320(2)	C(22)–C(23)	1.459(2)
N(2)–C(1)	1.334(2)	C(23)–C(29)	1.402(2)
N(3)–C(25)	1.367(2)	C(23)–C(24)	1.404(2)
N(3)–C(22)	1.374(2)	C(24)–C(26)	1.392(2)
N(4)–C(25)	1.324(2)	C(24)–C(25)	1.451(2)
N(4)–C(44)	1.331(2)	C(26)–C(27)	1.386(2)
C(1)–C(2)	1.458(2)	C(27)–C(28)	1.406(2)
C(2)–C(3)	1.389(2)	C(28)–C(29)	1.398(2)
C(2)–C(7)	1.397(2)	C(29)–C(30)	1.492(2)
C(3)–C(4)	1.390(2)	C(30)–C(35)	1.392(2)
C(4)–C(5)	1.398(3)	C(30)–C(31)	1.397(2)
C(5)–C(6)	1.396(2)	C(31)–C(32)	1.392(2)
C(6)–C(7)	1.399(2)	C(32)–C(33)	1.397(2)
C(6)–C(8)	1.490(2)	C(32)–C(36)	1.539(2)
C(7)–C(44)^{i}	1.466(2)	C(33)–C(34)	1.398(2)
C(8)–C(13)	1.391(2)	C(34)–C(35)	1.394(2)
C(8)–C(9)	1.394(2)	C(34)–C(40)	1.534(2)
C(9)–C(10)	1.393(2)	C(36)–C(39)	1.528(3)
C(10)–C(11)	1.395(2)	C(36)–C(37)	1.530(3)
C(10)–C(14)	1.536(2)	C(36)–C(38)	1.539(2)
C(11)–C(12)	1.393(2)	C(40)–C(43)	1.526(3)
C(12)–C(13)	1.394(2)	C(40)–C(42)	1.530(3)
C(12)–C(18)	1.536(2)	C(40)–C(41)	1.535(3)
C(14)–C(15)	1.527(3)	C(45)–Cl(2)	1.728(12)
C(14)–C(17)	1.533(3)	C(45)–Cl(1)	1.730(12)
C(14)–C(16)	1.536(3)	C(45)–Cl(3)	1.746(12)
C(18)–C(20')	1.404(8)	C(45')–Cl(2')	1.738(9)
C(18)–C(19)	1.437(10)	C(45')–Cl(3')	1.749(9)
C(18)–C(21)	1.519(7)	C(45')–Cl(1')	1.751(9)
C(18)–C(19')	1.575(9)		
**Bond angles**			
C(1)–N(1)–C(44)^{i}	107.84(14)	N(2)–C(22)–C(23)	124.86(14)
C(22)–N(2)–C(1)	123.56(14)	N(3)–C(22)–C(23)	107.39(13)
C(25)–N(3)–C(22	110.93(13)	C(29)–C(23)–C(24)	121.52(13)
C(25)–N(4)–C(44)	122.94(14)	C(29)–C(23)–C(22)	131.76(14)
N(2)–C(1)–N(1)	128.44(15)	C(24)–C(23)–C(22)	106.72(13)
N(2)–C(1)–C(2)	121.49(14)	C(26)–C(24)–C(23)	121.69(14)
N(1)–C(1)–C(2)	110.06(13)	C(26)–C(24)–C(25)	130.91(14)
C(3)–C(2)–C(7)	122.11(15)	C(23)–C(24)–C(25)	107.39(13)
C(3)–C(2)–C(1)	131.43(15)	N(4)–C(25)–N(3)	128.75(14)
C(7)–C(2)–C(1)	106.45(13)	N(4)–C(25)–C(24)	123.69(14)
C(2)–C(3)–C(4)	116.95(15)	N(3)–C(25)–C(24)	107.56(13)
C(3)–C(4)–C(5)	121.15(16)	C(27)–C(26)–C(24)	117.04(14)
C(6)–C(5)–C(4)	122.22(16)	C(26)–C(27)–C(28)	121.67(14)
C(5)–C(6)–C(7)	116.26(15)	C(29)–C(28)–C(27)	121.71(14)
C(5)–C(6)–C(8)	121.75(14)	C(28)–C(29)–C(23)	116.37(14)
C(7)–C(6)–C(8)	121.99(14)	C(28)–C(29)–C(30)	122.62(13)
C(2)–C(7)–C(6)	121.30(14)	C(23)–C(29)–C(30)	121.00(13)

C(2)–C(7)–C(44)^{i}	105.90(13)	C(35)–C(30)–C(31)	119.96(14)
C(6)–C(7)–C(44)^{i}	132.80(14)	C(35)–C(30)–C(29)	120.05(14)
C(13)–C(8)–C(9)	119.80(15)	C(31)–C(30)–C(29)	119.91(14)
C(13)–C(8)–C(6)	119.49(15)	C(32)–C(31)–C(30)	120.69(15)
C(9)–C(8)–C(6)	120.66(15)	C(31)–C(32)–C(33)	118.02(14)
C(10)–C(9)–C(8)	120.78(15)	C(31)–C(32)–C(36)	122.40(15)
C(9)–C(10)–C(11)	117.87(15)	C(33)–C(32)–C(36)	119.58(15)
C(9)–C(10)–C(14)	122.58(15)	C(32)–C(33)–C(34)	122.65(15)
C(11)–C(10)–C(14)	119.54(15)	C(35)–C(34)–C(33)	117.80(15)
C(12)–C(11)–C(10)	122.83(15)	C(35)–C(34)–C(40)	122.25(15)
C(11)–C(12)–C(13)	117.70(15)	C(33)–C(34)–C(40)	119.93(15)
C(11)–C(12)–C(18)	120.19(16)	C(30)–C(35)–C(34)	120.88(14)
C(13)–C(12)–C(18)	122.10(16)	C(39)–C(36)–C(37)	108.06(16)
C(8)–C(13)–C(12)	121.00(16)	C(39)–C(36)–C(32)	112.17(14)
C(15)–C(14)–C(17)	108.77(17)	C(37)–C(36)–C(32)	109.91(14)
C(15)–C(14)–C(16)	108.18(17)	C(39)–C(36)–C(38)	108.32(16)
C(17)–C(14)–C(16)	108.86(17)	C(37)–C(36)–C(38)	109.63(16)
C(15)–C(14)–C(10)	111.82(15)	C(32)–C(36)–C(38)	108.71(14)
C(17)–C(14)–C(10)	109.59(16)	C(43)–C(40)–C(42)	108.08(18)
C(16)–C(14)–C(10)	109.56(15)	C(43)–C(40)–C(34)	112.91(16)
C(19)–C(18)–C(21)	115.9(7)	C(42)–C(40)–C(34)	109.72(14)
C(20')–C(18)–C(12)	112.3(4)	C(43)–C(40)–C(41)	108.5(2)
C(19)–C(18)–C(12)	109.1(4)	C(42)–C(40)–C(41)	108.31(18)
C(21)–C(18)–C(12)	111.8(3)	C(34)–C(40)–C(41)	109.24(15)
C(20')–C(18)–C(19')	111.1(6)	N(4)–C(44)–N(1)^{i}	127.31(14)
C(12)–C(18)–C(19')	111.7(4)	N(4)–C(44)–C(7)^{i}	122.94(14)
C(19)–C(18)–C(20)	108.9(5)	N(1)^{i}–C(44)–C(7)^{i}	109.75(13)
C(21)–C(18)–C(20)	104.0(5)	Cl(2)–C(45)–Cl(1)	105.6(8)
C(12)–C(18)–C(20)	106.6(3)	Cl(2)–C(45)–Cl(3)	107.6(9)
C(20')–C(18)–C(21')	111.5(6)	Cl(1)–C(45)–Cl(3)	108.9(9)
C(12)–C(18)–C(21')	109.7(3)	Cl(2')–C(45')–Cl(3')	111.1(7)
C(19')–C(18)–C(21')	99.8(5)	Cl(2')–C(45')–Cl(1')	114.3(7)
N(2)–C(22)–N(3)	127.75(14)	Cl(3')–C(45')–Cl(1')	110.4(6)
**Torsion angles**			
C(22)–N(2)–C(1)–N(1)	–1.1(3)	C(25)–N(3)–C(22)–N(2)	–179.48(16)
C(22)–N(2)–C(1)–C(2)	178.70(15)	C(25)–N(3)–C(22)–C(23)	0.30(18)
C(44)^{i}–N(1)–C(1)–N(2)	–179.87(17)	N(2)–C(22)–C(23)–C(29)	–1.7(3)
C(44)^{i}–N(1)–C(1)–C(2)	0.34(19)	N(3)–C(22)–C(23)–C(29)	178.51(16)
N(2)–C(1)–C(2)–C(3)	–1.1(3)	N(2)–C(22)–C(23)–C(24)	179.12(15)
N(1)–C(1)–C(2)–C(3)	178.74(18)	N(3)–C(22)–C(23)–C(24)	–0.67(17)
N(2)–C(1)–C(2)–C(7)	179.93(15)	C(29)–C(23)–C(24)–C(26)	0.6(2)
N(1)–C(1)–C(2)–C(7)	–0.26(19)	C(22)–C(23)–C(24)–C(26)	179.93(14)
C(7)–C(2)–C(3)–C(4)	0.7(3)	C(29)–C(23)–C(24)–C(25)	–178.52(14)
C(1)–C(2)–C(3)–C(4)	–178.17(19)	C(22)–C(23)–C(24)–C(25)	0.77(17)
C(2)–C(3)–C(4)–C(5)	–1.0(3)	C(44)–N(4)–C(25)–N(3)	0.5(3)
C(3)–C(4)–C(5)–C(6)	0.2(3)	C(44)–N(4)–C(25)–C(24)	–179.38(15)
C(4)–C(5)–C(6)–C(7)	0.8(3)	C(22)–N(3)–C(25)–N(4)	–179.69(16)
C(4)–C(5)–C(6)–C(8)	–178.59(19)	C(22)–N(3)–C(25)–C(24)	0.18(19)
C(3)–C(2)–C(7)–C(6)	0.3(3)	C(26)–C(24)–C(25)–N(4)	0.2(3)
C(1)–C(2)–C(7)–C(6)	179.43(15)	C(23)–C(24)–C(25)–N(4)	179.27(15)
C(3)–C(2)–C(7)–C(44)^{i}	–179.03(16)	C(26)–C(24)–C(25)–N(3)	–179.66(16)
C(1)–C(2)–C(7)–C(44)^{i}	0.08(18)	C(23)–C(24)–C(25)–N(3)	–0.61(18)
C(5)–C(6)–C(7)–C(2)	–1.0(3)	C(23)–C(24)–C(26)–C(27)	–0.4(2)
C(8)–C(6)–C(7)–C(2)	178.32(15)	C(25)–C(24)–C(26)–C(27)	178.48(16)
C(5)–C(6)–C(7)–C(44)^{i}	178.13(18)	C(24)–C(26)–C(27)–C(28)	0.1(2)
C(8)–C(6)–C(7)–C(44)^{i}	–2.5(3)	C(26)–C(27)–C(28)–C(29)	0.1(3)
C(5)–C(6)–C(8)–C(13)	92.3(2)	C(27)–C(28)–C(29)–C(23)	0.0(2)

C(7)–C(6)–C(8)–C(13)	–87.0(2)	C(27)–C(28)–C(29)–C(30)	–179.80(14)
C(5)–C(6)–C(8)–C(9)	–90.2(2)	C(24)–C(23)–C(29)–C(28)	–0.4(2)
C(7)–C(6)–C(8)–C(9)	90.5(2)	C(22)–C(23)–C(29)–C(28)	–179.50(16)
C(13)–C(8)–C(9)–C(10)	0.0(2)	C(24)–C(23)–C(29)–C(30)	179.43(14)
C(6)–C(8)–C(9)–C(10)	–177.46(15)	C(22)–C(23)–C(29)–C(30)	0.3(3)
C(8)–C(9)–C(10)–C(11)	–0.5(2)	C(28)–C(29)–C(30)–C(35)	–89.12(19)
C(8)–C(9)–C(10)–C(14)	178.80(15)	C(23)–C(29)–C(30)–C(35)	91.05(18)
C(9)–C(10)–C(11)–C(12)	1.1(3)	C(28)–C(29)–C(30)–C(31)	94.36(19)
C(14)–C(10)–C(11)–C(12)	–178.19(16)	C(23)–C(29)–C(30)–C(31)	–85.47(19)
C(10)–C(11)–C(12)–C(13)	–1.2(3)	C(35)–C(30)–C(31)–C(32)	–1.1(2)
C(10)–C(11)–C(12)–C(18)	178.00(17)	C(29)–C(30)–C(31)–C(32)	175.40(14)
C(9)–C(8)–C(13)–C(12)	–0.1(3)	C(30)–C(31)–C(32)–C(33)	1.0(2)
C(6)–C(8)–C(13)–C(12)	177.36(16)	C(30)–C(31)–C(32)–C(36)	–179.06(14)
C(11)–C(12)–C(13)–C(8)	0.7(3)	C(31)–C(32)–C(33)–C(34)	–0.8(2)
C(18)–C(12)–C(13)–C(8)	–178.49(18)	C(36)–C(32)–C(33)–C(34)	179.28(15)
C(9)–C(10)–C(14)–C(15)	–4.8(2)	C(32)–C(33)–C(34)–C(35)	0.6(2)
C(11)–C(10)–C(14)–C(15)	174.40(17)	C(32)–C(33)–C(34)–C(40)	–177.77(15)
C(9)–C(10)–C(14)–C(17)	115.86(19)	C(31)–C(30)–C(35)–C(34)	1.0(2)
C(11)–C(10)–C(14)–C(17)	–64.9(2)	C(29)–C(30)–C(35)–C(34)	–175.55(14)
C(9)–C(10)–C(14)–C(16)	–124.77(18)	C(33)–C(34)–C(35)–C(30)	–0.7(2)
C(11)–C(10)–C(14)–C(16)	54.5(2)	C(40)–C(34)–C(35)–C(30)	177.66(14)
C(11)–C(12)–C(18)–C(20')	80.4(8)	C(31)–C(32)–C(36)–C(39)	3.1(2)
C(13)–C(12)–C(18)–C(20')	–100.4(8)	C(33)–C(32)–C(36)–C(39)	–176.97(16)
C(11)–C(12)–C(18)–C(19)	–66.6(6)	C(31)–C(32)–C(36)–C(37)	123.36(18)
C(13)–C(12)–C(18)–C(19)	112.6(6)	C(33)–C(32)–C(36)–C(37)	–56.7(2)
C(11)–C(12)–C(18)–C(21)	163.9(6)	C(31)–C(32)–C(36)–C(38)	–116.65(18)
C(13)–C(12)–C(18)–C(21)	–16.9(7)	C(33)–C(32)–C(36)–C(38)	63.3(2)
C(11)–C(12)–C(18)–C(19')	–45.3(5)	C(35)–C(34)–C(40)–C(43)	5.4(3)
C(13)–C(12)–C(18)–C(19')	133.9(5)	C(33)–C(34)–C(40)–C(43)	–176.24(19)
C(11)–C(12)–C(18)–C(20)	50.8(4)	C(35)–C(34)–C(40)–C(42)	126.05(18)
C(13)–C(12)–C(18)–C(20)	–130.0(3)	C(33)–C(34)–C(40)–C(42)	–55.6(2)
C(11)–C(12)–C(18)–C(21')	–155.0(4)	C(35)–C(34)–C(40)–C(41)	–115.4(2)
C(13)–C(12)–C(18)–C(21')	24.1(5)	C(33)–C(34)–C(40)–C(41)	63.0(2)
C(1)–N(2)–C(22)–N(3)	–0.6(3)	C(25)–N(4)–C(44)–N(1)^{i}	–1.3(3)
C(1)–N(2)–C(22)–C(23)	179.70(15)	C(25)–N(4)–C(44)–C(7)^{i}	178.22(15)

symmetry codes: (i) -x+2, -y+1, -z+1

Table S4: Geometric Parameters (Å,°) for **NiPc-Windmill**.

**Bond lengths**			
Ni1—N1^{i}	1.904 (2)	C21—C23'	1.436 (14)
Ni1—N1	1.904 (2)	C21—C22	1.457 (18)
Ni1—N3	1.905 (2)	C21—C24	1.516 (13)
Ni1—N3^{i}	1.905 (2)	C21—C22'	1.594 (16)
N1—C4	1.379 (4)	C21—C24'	1.604 (14)
N1—C1	1.388 (3)	C21—C23	1.607 (10)
N2—C1	1.307 (4)	C25—C30	1.395 (4)
N2—C2	1.332 (4)	C25—C26	1.399 (4)
N3—C2	1.374 (3)	C26—C27	1.398 (4)

N3—C3	1.380 (3)	C27—C28	1.402 (4)
N4—C4^{i}	1.314 (4)	C27—C31	1.494 (4)
N4—C3	1.321 (4)	C28—C29	1.406 (4)
C1—C10	1.457 (4)	C29—C30	1.386 (4)
C2—C25	1.440 (4)	C31—C36	1.387 (4)
C3—C26	1.452 (4)	C31—C32	1.394 (4)
C4—C5	1.450 (4)	C32—C33	1.402 (4)
C5—C6	1.391 (4)	C33—C34	1.392 (5)
C5—C10	1.391 (4)	C33—C37	1.540 (4)
C6—C7	1.397 (4)	C34—C35	1.404 (4)
C7—C8	1.387 (5)	C35—C36	1.396 (4)
C8—C9	1.395 (4)	C35—C41	1.530 (4)
C9—C10	1.404 (4)	C37—C39	1.528 (5)
C9—C11	1.488 (4)	C37—C38	1.532 (5)
C11—C16	1.391 (4)	C37—C40	1.537 (5)
C11—C12	1.391 (4)	C41—C44	1.528 (5)
C12—C13	1.391 (4)	C41—C42	1.531 (5)
C13—C14	1.404 (4)	C41—C43	1.551 (6)
C13—C17	1.527 (4)	Cl1—C45	1.755 (7)
C14—C15	1.386 (5)	Cl2—C45	1.748 (8)
C15—C16	1.395 (5)	Cl3—C45	1.753 (8)
C15—C21	1.526 (4)	Cl1'—C45'	1.762 (8)
C17—C18	1.528 (5)	Cl2'—C45'	1.757 (8)
C17—C19	1.534 (5)	Cl3'—C45'	1.761 (8)
C17—C20	1.536 (5)		
**Bond angles**			
N1^{i}—Ni1—N1	180.0	C19—C17—C20	109.2 (3)
N1^{i}—Ni1—N3	90.17 (10)	C22—C21—C24	118.8 (12)
N1—Ni1—N3	89.83 (10)	C23'—C21—C15	111.5 (8)
N1^{i}—Ni1—N3^{i}	89.83 (10)	C22—C21—C15	108.4 (7)
N1—Ni1—N3^{i}	90.17 (10)	C24—C21—C15	111.3 (6)
N3—Ni1—N3^{i}	180.0	C23'—C21—C22'	110.4 (11)
C4—N1—C1	106.8 (2)	C15—C21—C22'	113.0 (7)
C4—N1—Ni1	126.12 (19)	C23'—C21—C24'	109.5 (12)
C1—N1—Ni1	127.09 (19)	C15—C21—C24'	110.9 (5)
C1—N2—C2	121.2 (2)	C22'—C21—C24'	101.2 (9)
C2—N3—C3	106.3 (2)	C22—C21—C23	108.3 (8)
C2—N3—Ni1	126.86 (19)	C24—C21—C23	102.5 (8)
C3—N3—Ni1	126.80 (18)	C15—C21—C23	106.8 (5)
C4^{i}—N4—C3	120.8 (3)	C30—C25—C26	121.9 (3)
N2—C1—N1	127.3 (2)	C30—C25—C2	131.4 (3)
N2—C1—C10	122.8 (2)	C26—C25—C2	106.7 (2)
N1—C1—C10	109.8 (2)	C27—C26—C25	121.7 (2)
N2—C2—N3	127.7 (3)	C27—C26—C3	132.7 (3)
N2—C2—C25	121.6 (2)	C25—C26—C3	105.6 (2)
N3—C2—C25	110.7 (2)	C26—C27—C28	116.3 (3)
N4—C3—N3	127.5 (2)	C26—C27—C31	121.6 (2)
N4—C3—C26	121.9 (2)	C28—C27—C31	122.1 (2)
N3—C3—C26	110.6 (2)	C27—C28—C29	121.6 (3)
N4^{i}—C4—N1	128.6 (3)	C30—C29—C28	121.8 (3)

N4^{i}—C4—C5	121.2 (3)	C29—C30—C25	116.8 (3)
N1—C4—C5	110.2 (2)	C36—C31—C32	120.0 (3)
C6—C5—C10	122.5 (3)	C36—C31—C27	120.2 (2)
C6—C5—C4	130.6 (3)	C32—C31—C27	119.6 (3)
C10—C5—C4	106.8 (2)	C31—C32—C33	120.4 (3)
C5—C6—C7	116.2 (3)	C34—C33—C32	118.1 (3)
C8—C7—C6	121.4 (3)	C34—C33—C37	119.8 (3)
C7—C8—C9	122.7 (3)	C32—C33—C37	122.1 (3)
C8—C9—C10	115.7 (3)	C33—C34—C35	122.6 (3)
C8—C9—C11	121.8 (3)	C36—C35—C34	117.5 (3)
C10—C9—C11	122.5 (3)	C36—C35—C41	123.0 (3)
C5—C10—C9	121.4 (3)	C34—C35—C41	119.5 (3)
C5—C10—C1	106.4 (2)	C31—C36—C35	121.3 (3)
C9—C10—C1	132.2 (3)	C39—C37—C38	108.0 (3)
C16—C11—C12	120.2 (3)	C39—C37—C40	108.7 (3)
C16—C11—C9	119.5 (3)	C38—C37—C40	109.6 (3)
C12—C11—C9	120.3 (3)	C39—C37—C33	112.3 (3)
C13—C12—C11	120.7 (3)	C38—C37—C33	109.9 (3)
C12—C13—C14	117.6 (3)	C40—C37—C33	108.2 (3)
C12—C13—C17	122.8 (3)	C44—C41—C35	112.6 (3)
C14—C13—C17	119.6 (3)	C44—C41—C42	108.3 (3)
C15—C14—C13	123.0 (3)	C35—C41—C42	109.9 (3)
C14—C15—C16	117.7 (3)	C44—C41—C43	108.2 (4)
C14—C15—C21	120.1 (3)	C35—C41—C43	109.2 (3)
C16—C15—C21	122.2 (3)	C42—C41—C43	108.4 (3)
C11—C16—C15	120.8 (3)	Cl2—C45—Cl3	109.5 (7)
C13—C17—C18	111.8 (3)	Cl2—C45—Cl1	110.5 (7)
C13—C17—C19	109.8 (3)	Cl3—C45—Cl1	111.3 (6)
C18—C17—C19	108.2 (3)	Cl2'—C45'—Cl3'	109.5 (7)
C13—C17—C20	109.7 (3)	Cl2'—C45'—Cl1'	109.0 (8)
C18—C17—C20	108.0 (3)	Cl3'—C45'—Cl1'	108.2 (7)
**Torsion angles**			
C2—N2—C1—N1	0.7 (5)	C12—C13—C17—C20	-124.2 (3)
C2—N2—C1—C10	-179.3 (3)	C14—C13—C17—C20	55.2 (4)
C4—N1—C1—N2	179.6 (3)	C14—C15—C21—C23'	80.4 (15)
Ni1—N1—C1—N2	-1.1 (4)	C16—C15—C21—C23'	-101.2 (14)
C4—N1—C1—C10	-0.4 (3)	C14—C15—C21—C22	-64.8 (10)
Ni1—N1—C1—C10	178.87 (19)	C16—C15—C21—C22	113.6 (10)
C1—N2—C2—N3	0.7 (5)	C14—C15—C21—C24	162.7 (11)
C1—N2—C2—C25	178.9 (3)	C16—C15—C21—C24	-18.9 (11)
C3—N3—C2—N2	179.0 (3)	C14—C15—C21—C22'	-44.6 (10)
Ni1—N3—C2—N2	-1.5 (4)	C16—C15—C21—C22'	133.8 (9)
C3—N3—C2—C25	0.7 (3)	C14—C15—C21—C24'	-157.3 (8)
Ni1—N3—C2—C25	-179.86 (19)	C16—C15—C21—C24'	21.1 (9)
C4^{i}—N4—C3—N3	0.9 (5)	C14—C15—C21—C23	51.6 (6)
C4^{i}—N4—C3—C26	179.9 (3)	C16—C15—C21—C23	-130.0 (6)
C2—N3—C3—N4	178.5 (3)	N2—C2—C25—C30	1.0 (5)
Ni1—N3—C3—N4	-1.0 (4)	N3—C2—C25—C30	179.5 (3)
C2—N3—C3—C26	-0.7 (3)	N2—C2—C25—C26	-178.9 (3)
Ni1—N3—C3—C26	179.88 (19)	N3—C2—C25—C26	-0.4 (3)

C1—N1—C4—N4^{i}	-179.9 (3)	C30—C25—C26—C27	-1.5 (4)
Ni1—N1—C4—N4^{i}	0.9 (5)	C2—C25—C26—C27	178.5 (2)
C1—N1—C4—C5	0.5 (3)	C30—C25—C26—C3	-179.9 (3)
Ni1—N1—C4—C5	-178.8 (2)	C2—C25—C26—C3	0.0 (3)
N4^{i}—C4—C5—C6	-1.1 (5)	N4—C3—C26—C27	3.0 (5)
N1—C4—C5—C6	178.5 (3)	N3—C3—C26—C27	-177.8 (3)
N4^{i}—C4—C5—C10	179.9 (3)	N4—C3—C26—C25	-178.8 (3)
N1—C4—C5—C10	-0.4 (3)	N3—C3—C26—C25	0.4 (3)
C10—C5—C6—C7	0.5 (5)	C25—C26—C27—C28	1.0 (4)
C4—C5—C6—C7	-178.3 (3)	C3—C26—C27—C28	178.9 (3)
C5—C6—C7—C8	-0.6 (6)	C25—C26—C27—C31	-179.1 (2)
C6—C7—C8—C9	-0.5 (6)	C3—C26—C27—C31	-1.1 (5)
C7—C8—C9—C10	1.6 (5)	C26—C27—C28—C29	-0.1 (4)
C7—C8—C9—C11	-178.2 (3)	C31—C27—C28—C29	180.0 (3)
C6—C5—C10—C9	0.7 (5)	C27—C28—C29—C30	-0.3 (4)
C4—C5—C10—C9	179.8 (3)	C28—C29—C30—C25	-0.1 (4)
C6—C5—C10—C1	-178.9 (3)	C26—C25—C30—C29	1.0 (4)
C4—C5—C10—C1	0.2 (3)	C2—C25—C30—C29	-178.9 (3)
C8—C9—C10—C5	-1.7 (5)	C26—C27—C31—C36	-91.2 (3)
C11—C9—C10—C5	178.1 (3)	C28—C27—C31—C36	88.7 (3)
C8—C9—C10—C1	177.7 (3)	C26—C27—C31—C32	85.0 (3)
C11—C9—C10—C1	-2.5 (5)	C28—C27—C31—C32	-95.0 (3)
N2—C1—C10—C5	-179.9 (3)	C36—C31—C32—C33	0.6 (4)
N1—C1—C10—C5	0.1 (3)	C27—C31—C32—C33	-175.6 (3)
N2—C1—C10—C9	0.6 (5)	C31—C32—C33—C34	-0.4 (4)
N1—C1—C10—C9	-179.4 (3)	C31—C32—C33—C37	179.0 (3)
C8—C9—C11—C16	92.2 (4)	C32—C33—C34—C35	0.1 (4)
C10—C9—C11—C16	-87.6 (4)	C37—C33—C34—C35	-179.3 (3)
C8—C9—C11—C12	-90.3 (4)	C33—C34—C35—C36	-0.1 (4)
C10—C9—C11—C12	89.9 (4)	C33—C34—C35—C41	177.7 (3)
C16—C11—C12—C13	-0.2 (4)	C32—C31—C36—C35	-0.7 (4)
C9—C11—C12—C13	-177.6 (3)	C27—C31—C36—C35	175.6 (3)
C11—C12—C13—C14	-0.4 (4)	C34—C35—C36—C31	0.4 (4)
C11—C12—C13—C17	179.1 (3)	C41—C35—C36—C31	-177.3 (3)
C12—C13—C14—C15	1.0 (5)	C34—C33—C37—C39	176.6 (3)
C17—C13—C14—C15	-178.5 (3)	C32—C33—C37—C39	-2.7 (4)
C13—C14—C15—C16	-0.9 (5)	C34—C33—C37—C38	56.3 (4)
C13—C14—C15—C21	177.5 (3)	C32—C33—C37—C38	-123.1 (3)
C12—C11—C16—C15	0.2 (5)	C34—C33—C37—C40	-63.4 (4)
C9—C11—C16—C15	177.7 (3)	C32—C33—C37—C40	117.3 (3)
C14—C15—C16—C11	0.4 (5)	C36—C35—C41—C44	-5.5 (5)
C21—C15—C16—C11	-178.1 (3)	C34—C35—C41—C44	176.8 (4)
C12—C13—C17—C18	-4.3 (4)	C36—C35—C41—C42	-126.4 (3)
C14—C13—C17—C18	175.1 (3)	C34—C35—C41—C42	55.9 (4)
C12—C13—C17—C19	115.8 (3)	C36—C35—C41—C43	114.8 (4)
C14—C13—C17—C19	-64.8 (4)	C34—C35—C41—C43	-62.9 (4)

symmetry codes: (i) -x+1, -y+1, -z+1

Table S5: Geometric Parameters (Å,°) for **[CoPc-Dragon(py)$_2$]**.

**Bond lengths**			
Co(1)–N(3)	1.903(3)	C(33')–C(34')	1.374(16)
Co(1)–N(7)	1.909(3)	C(33')–C(37')	1.535(14)
Co(1)–N(1)	1.916(3)	C(34')–C(35')	1.404(14)
Co(1)–N(5)	1.916(3)	C(35')–C(36')	1.378(13)
Co(1)–N(10)	2.236(5)	C(35')–C(41')	1.514(15)
Co(1)–N(9)	2.272(3)	C(37')–C(39')	1.49(2)
N(1)–C(4)	1.366(4)	C(37')–C(38')	1.55(2)
N(1)–C(1)	1.378(5)	C(37')–C(40')	1.574(18)
N(2)–C(1)	1.320(5)	C(41')–C(43')	1.509(16)
N(2)–C(23)	1.322(5)	C(41')–C(42')	1.516(15)
N(3)–C(26)	1.375(5)	C(41')–C(44')	1.550(16)
N(3)–C(23)	1.382(5)	C(45)–C(46)	1.465(5)
N(4)–C(45)	1.320(5)	C(46)–C(47)	1.399(6)
N(4)–C(26)	1.326(5)	C(46)–C(52)	1.400(5)
N(5)–C(48)	1.377(5)	C(47)–C(49)	1.398(5)
N(5)–C(45)	1.378(5)	C(47)–C(48)	1.458(5)
N(6)–C(48)	1.323(5)	C(49)–C(50)	1.393(5)
N(6)–C(67)	1.325(4)	C(49)–C(53)	1.481(5)
N(7)–C(67)	1.371(4)	C(50)–C(51)	1.403(7)
N(7)–C(70)	1.374(4)	C(51)–C(52)	1.377(7)
N(8)–C(4)	1.317(5)	C(53)–C(58)	1.391(6)
N(8)–C(70)	1.328(4)	C(53)–C(54)	1.402(5)
N(9)–C(89)	1.321(5)	C(54)–C(55)	1.395(6)
N(9)–C(93)	1.334(4)	C(55)–C(56)	1.405(6)
C(1)–C(2)	1.483(5)	C(55)–C(59)	1.538(5)
C(2)–C(3)	1.403(5)	C(56)–C(57)	1.402(5)
C(2)–C(5)	1.415(6)	C(57)–C(58)	1.402(6)
C(3)–C(8)	1.388(5)	C(57)–C(63)	1.535(6)
C(3)–C(4)	1.443(5)	C(59)–C(62)	1.512(7)
C(5)–C(6)	1.399(6)	C(59)–C(61)	1.536(6)
C(5)–C(9)	1.482(6)	C(59)–C(60)	1.542(6)
C(6)–C(7)	1.386(6)	C(63)–C(66)	1.516(7)
C(7)–C(8)	1.375(6)	C(63)–C(65)	1.517(6)
C(9)–C(14)	1.387(7)	C(63)–C(64)	1.525(6)
C(9)–C(10)	1.387(6)	C(67)–C(68)	1.451(5)
C(10)–C(11)	1.392(7)	C(68)–C(74)	1.385(5)
C(11)–C(12)	1.393(8)	C(68)–C(69)	1.397(4)
C(11)–C(15)	1.535(8)	C(69)–C(71)	1.405(5)
C(12)–C(13)	1.386(8)	C(69)–C(70)	1.449(5)
C(13)–C(14)	1.385(8)	C(71)–C(72)	1.393(5)
C(13)–C(19)	1.553(9)	C(71)–C(75)	1.479(4)
C(15)–C(16')	1.45(3)	C(72)–C(73)	1.394(5)
C(15)–C(17')	1.52(2)	C(73)–C(74)	1.385(5)
C(15)–C(16)	1.527(11)	C(75)–C(80)	1.393(4)
C(15)–C(18)	1.547(11)	C(75)–C(76)	1.398(5)
C(15)–C(17)	1.576(10)	C(76)–C(77)	1.386(5)
C(15)–C(18')	1.58(2)	C(77)–C(78)	1.398(5)
C(19)–C(20)	1.524(17)	C(77)–C(81)	1.529(5)
C(19)–C(21)	1.549(10)	C(78)–C(79)	1.391(5)
C(19)–C(22)	1.554(16)	C(79)–C(80)	1.398(5)
C(23)–C(24)	1.462(5)	C(79)–C(85)	1.528(5)
C(24)–C(25)	1.403(6)	C(81)–C(83)	1.524(6)
C(24)–C(27)	1.405(7)	C(81)–C(82)	1.530(6)

C(25)–C(30)	1.374(6)	C(81)–C(84)	1.538(6)
C(25)–C(26)	1.441(6)	C(85)–C(86')	1.439(17)
C(27)–C(28)	1.412(7)	C(85)–C(87)	1.477(7)
C(27)–C(31')	1.474(12)	C(85)–C(88)	1.521(7)
C(27)–C(31)	1.541(11)	C(85)–C(87')	1.529(19)
C(28)–C(29)	1.388(8)	C(85)–C(86)	1.561(7)
C(29)–C(30)	1.381(8)	C(85)–C(88')	1.652(18)
C(31)–C(36)	1.396(12)	C(89)–C(90)	1.369(6)
C(31)–C(32)	1.415(11)	C(90)–C(91)	1.377(5)
C(32)–C(33)	1.384(13)	C(91)–C(92)	1.359(6)
C(33)–C(34)	1.370(14)	C(92)–C(93)	1.373(5)
C(33)–C(37)	1.534(12)	N(10)–C(94)	1.315(7)
C(34)–C(35)	1.410(13)	N(10)–C(98)	1.374(8)
C(35)–C(36)	1.374(12)	C(94)–C(95)	1.382(10)
C(35)–C(41)	1.516(14)	C(95)–C(96)	1.388(10)
C(37)–C(39)	1.51(2)	C(96)–C(97)	1.332(9)
C(37)–C(38)	1.537(17)	C(97)–C(98)	1.377(9)
C(37)–C(40)	1.570(15)	N(10')–C(94')	1.3900
C(41)–C(43)	1.491(16)	N(10')–C(98')	1.3900
C(41)–C(42)	1.498(14)	C(94')–C(95')	1.3900
C(41)–C(44)	1.561(15)	C(95')–C(96')	1.3900
C(31')–C(36')	1.370(14)	C(96')–C(97')	1.3900
C(31')–C(32')	1.401(13)	C(97')–C(98')	1.3900
C(32')–C(33')	1.380(14)		
**Bond angles**			
N(3)–Co(1)–N(7)	176.53(13)	C(34')–C(33')–C(32')	118.5(10)
N(3)–Co(1)–N(1)	89.83(13)	C(34')–C(33')–C(37')	122.7(12)
N(7)–Co(1)–N(1)	90.09(12)	C(32')–C(33')–C(37')	118.9(13)
N(3)–Co(1)–N(5)	90.11(13)	C(33')–C(34')–C(35')	123.7(11)
N(7)–Co(1)–N(5)	90.34(12)	C(36')–C(35')–C(34')	116.3(12)
N(1)–Co(1)–N(5)	174.00(12)	C(36')–C(35')–C(41')	122.0(12)
N(3)–Co(1)–N(10)	88.28(16)	C(34')–C(35')–C(41')	121.3(11)
N(7)–Co(1)–N(10)	95.18(14)	C(31')–C(36')–C(35')	121.1(12)
N(1)–Co(1)–N(10)	86.68(14)	C(39')–C(37')–C(33')	115.6(13)
N(5)–Co(1)–N(10)	87.32(15)	C(39')–C(37')–C(38')	110.7(15)
N(3)–Co(1)–N(9)	87.06(12)	C(33')–C(37')–C(38')	107.5(15)
N(7)–Co(1)–N(9)	89.48(11)	C(39')–C(37')–C(40')	109.2(14)
N(1)–Co(1)–N(9)	92.10(11)	C(33')–C(37')–C(40')	106.7(11)
N(5)–Co(1)–N(9)	93.89(11)	C(38')–C(37')–C(40')	106.6(14)
N(10)–Co(1)–N(9)	175.19(15)	C(43')–C(41')–C(35')	112.4(13)
C(4)–N(1)–C(1)	107.7(3)	C(43')–C(41')–C(42')	104.9(14)
C(4)–N(1)–Co(1)	125.2(2)	C(35')–C(41')–C(42')	112.6(13)
C(1)–N(1)–Co(1)	126.5(2)	C(43')–C(41')–C(44')	105.9(13)
C(1)–N(2)–C(23)	122.7(3)	C(35')–C(41')–C(44')	112.9(11)
C(26)–N(3)–C(23)	107.6(3)	C(42')–C(41')–C(44')	107.5(15)
C(26)–N(3)–Co(1)	125.9(3)	N(4)–C(45)–N(5)	127.0(4)
C(23)–N(3)–Co(1)	125.4(2)	N(4)–C(45)–C(46)	123.7(3)
C(45)–N(4)–C(26)	121.2(3)	N(5)–C(45)–C(46)	109.2(3)
C(48)–N(5)–C(45)	107.7(3)	C(47)–C(46)–C(52)	121.5(4)
C(48)–N(5)–Co(1)	125.4(2)	C(47)–C(46)–C(45)	106.8(3)
C(45)–N(5)–Co(1)	126.6(3)	C(52)–C(46)–C(45)	131.6(4)
C(48)–N(6)–C(67)	121.3(3)	C(49)–C(47)–C(46)	122.0(3)
C(67)–N(7)–C(70)	106.6(3)	C(49)–C(47)–C(48)	131.9(4)
C(67)–N(7)–Co(1)	126.2(2)	C(46)–C(47)–C(48)	106.0(3)
C(70)–N(7)–Co(1)	126.7(2)	N(6)–C(48)–N(5)	127.4(3)
C(4)–N(8)–C(70)	122.0(3)	N(6)–C(48)–C(47)	122.1(3)
C(89)–N(9)–C(93)	116.4(3)	N(5)–C(48)–C(47)	110.1(3)

C(89)–N(9)–Co(1)	121.8(2)	C(50)–C(49)–C(47)	116.2(4)
C(93)–N(9)–Co(1)	121.7(3)	C(50)–C(49)–C(53)	122.3(4)
N(2)–C(1)–N(1)	124.6(3)	C(47)–C(49)–C(53)	121.5(3)
N(2)–C(1)–C(2)	125.3(4)	C(49)–C(50)–C(51)	121.3(4)
N(1)–C(1)–C(2)	109.7(3)	C(52)–C(51)–C(50)	122.7(4)
C(3)–C(2)–C(5)	120.2(3)	C(51)–C(52)–C(46)	116.2(4)
C(3)–C(2)–C(1)	104.3(3)	C(58)–C(53)–C(54)	118.7(4)
C(5)–C(2)–C(1)	135.4(4)	C(58)–C(53)–C(49)	122.1(3)
C(8)–C(3)–C(2)	123.2(4)	C(54)–C(53)–C(49)	119.2(4)
C(8)–C(3)–C(4)	129.0(4)	C(55)–C(54)–C(53)	121.4(4)
C(2)–C(3)–C(4)	107.8(3)	C(54)–C(55)–C(56)	118.1(3)
N(8)–C(4)–N(1)	128.0(3)	C(54)–C(55)–C(59)	122.7(4)
N(8)–C(4)–C(3)	122.0(3)	C(56)–C(55)–C(59)	119.3(4)
N(1)–C(4)–C(3)	110.0(3)	C(57)–C(56)–C(55)	122.3(4)
C(6)–C(5)–C(2)	115.1(4)	C(56)–C(57)–C(58)	117.4(4)
C(6)–C(5)–C(9)	117.7(4)	C(56)–C(57)–C(63)	119.5(4)
C(2)–C(5)–C(9)	127.0(4)	C(58)–C(57)–C(63)	122.9(3)
C(7)–C(6)–C(5)	123.7(4)	C(53)–C(58)–C(57)	122.1(3)
C(8)–C(7)–C(6)	121.2(4)	C(62)–C(59)–C(61)	110.1(4)
C(7)–C(8)–C(3)	116.6(4)	C(62)–C(59)–C(55)	112.3(4)
C(14)–C(9)–C(10)	118.6(5)	C(61)–C(59)–C(55)	109.1(3)
C(14)–C(9)–C(5)	119.8(4)	C(62)–C(59)–C(60)	108.0(4)
C(10)–C(9)–C(5)	121.3(4)	C(61)–C(59)–C(60)	107.9(4)
C(9)–C(10)–C(11)	121.7(5)	C(55)–C(59)–C(60)	109.4(4)
C(10)–C(11)–C(12)	117.1(5)	C(66)–C(63)–C(65)	108.2(4)
C(10)–C(11)–C(15)	119.9(5)	C(66)–C(63)–C(64)	109.9(5)
C(12)–C(11)–C(15)	122.9(5)	C(65)–C(63)–C(64)	107.0(4)
C(13)–C(12)–C(11)	123.2(6)	C(66)–C(63)–C(57)	108.6(4)
C(14)–C(13)–C(12)	117.3(6)	C(65)–C(63)–C(57)	111.7(4)
C(14)–C(13)–C(19)	119.0(6)	C(64)–C(63)–C(57)	111.4(3)
C(12)–C(13)–C(19)	123.7(6)	N(6)–C(67)–N(7)	127.2(3)
C(13)–C(14)–C(9)	122.1(5)	N(6)–C(67)–C(68)	122.3(3)
C(16')–C(15)–C(17')	115.8(18)	N(7)–C(67)–C(68)	110.5(3)
C(16')–C(15)–C(11)	104.3(13)	C(74)–C(68)–C(69)	122.4(3)
C(17')–C(15)–C(11)	104.4(12)	C(74)–C(68)–C(67)	131.4(3)
C(16)–C(15)–C(11)	110.4(6)	C(69)–C(68)–C(67)	106.2(3)
C(16)–C(15)–C(18)	111.1(8)	C(68)–C(69)–C(71)	120.5(3)
C(11)–C(15)–C(18)	109.7(6)	C(68)–C(69)–C(70)	106.0(3)
C(16)–C(15)–C(17)	107.4(7)	C(71)–C(69)–C(70)	133.4(3)
C(11)–C(15)–C(17)	112.7(6)	N(8)–C(70)–N(7)	125.1(3)
C(18)–C(15)–C(17)	105.5(7)	N(8)–C(70)–C(69)	124.0(3)
C(16')–C(15)–C(18')	114.4(16)	N(7)–C(70)–C(69)	110.5(3)
C(17')–C(15)–C(18')	108.1(17)	C(72)–C(71)–C(69)	116.5(3)
C(11)–C(15)–C(18')	109.1(9)	C(72)–C(71)–C(75)	121.4(3)
C(20)–C(19)–C(21)	110.7(11)	C(69)–C(71)–C(75)	122.1(3)
C(20)–C(19)–C(13)	109.3(9)	C(71)–C(72)–C(73)	122.2(3)
C(21)–C(19)–C(13)	111.4(7)	C(74)–C(73)–C(72)	121.2(4)
C(20)–C(19)–C(22)	109.6(9)	C(73)–C(74)–C(68)	117.1(3)
C(21)–C(19)–C(22)	108.9(10)	C(80)–C(75)–C(76)	118.7(3)
C(13)–C(19)–C(22)	106.9(9)	C(80)–C(75)–C(71)	120.6(3)
N(2)–C(23)–N(3)	125.8(3)	C(76)–C(75)–C(71)	120.7(3)
N(2)–C(23)–C(24)	124.5(4)	C(77)–C(76)–C(75)	122.0(3)
N(3)–C(23)–C(24)	109.3(3)	C(76)–C(77)–C(78)	117.4(3)
C(25)–C(24)–C(27)	120.5(4)	C(76)–C(77)–C(81)	122.4(3)
C(25)–C(24)–C(23)	105.8(4)	C(78)–C(77)–C(81)	120.2(3)
C(27)–C(24)–C(23)	133.7(4)	C(79)–C(78)–C(77)	122.8(3)
C(30)–C(25)–C(24)	122.5(5)	C(78)–C(79)–C(80)	117.8(3)
C(30)–C(25)–C(26)	130.5(5)	C(78)–C(79)–C(85)	122.0(3)

C(24)–C(25)–C(26)	107.0(4)	C(80)–C(79)–C(85)	120.1(3)
N(4)–C(26)–N(3)	127.8(4)	C(75)–C(80)–C(79)	121.3(3)
N(4)–C(26)–C(25)	122.3(4)	C(83)–C(81)–C(77)	111.1(3)
N(3)–C(26)–C(25)	109.9(4)	C(83)–C(81)–C(82)	109.3(3)
C(24)–C(27)–C(28)	115.5(5)	C(77)–C(81)–C(82)	108.4(4)
C(24)–C(27)–C(31')	123.3(7)	C(83)–C(81)–C(84)	107.4(4)
C(28)–C(27)–C(31')	120.9(7)	C(77)–C(81)–C(84)	111.9(3)
C(24)–C(27)–C(31)	126.4(6)	C(82)–C(81)–C(84)	108.6(4)
C(28)–C(27)–C(31)	116.0(6)	C(87)–C(85)–C(88)	110.9(5)
C(29)–C(28)–C(27)	122.8(5)	C(86')–C(85)–C(79)	113.0(7)
C(30)–C(29)–C(28)	120.6(5)	C(87)–C(85)–C(79)	109.8(4)
C(25)–C(30)–C(29)	117.7(5)	C(88)–C(85)–C(79)	109.0(4)
C(36)–C(31)–C(32)	116.0(10)	C(86')–C(85)–C(87')	115.5(12)
C(36)–C(31)–C(27)	121.4(9)	C(79)–C(85)–C(87')	106.7(8)
C(32)–C(31)–C(27)	122.5(9)	C(87)–C(85)–C(86)	111.2(5)
C(33)–C(32)–C(31)	122.9(10)	C(88)–C(85)–C(86)	105.8(5)
C(34)–C(33)–C(32)	118.2(9)	C(79)–C(85)–C(86)	110.0(4)
C(34)–C(33)–C(37)	123.4(10)	C(86')–C(85)–C(88')	105.4(12)
C(32)–C(33)–C(37)	118.2(10)	C(79)–C(85)–C(88')	112.7(7)
C(33)–C(34)–C(35)	121.6(10)	C(87')–C(85)–C(88')	103.2(11)
C(36)–C(35)–C(34)	118.3(11)	N(9)–C(89)–C(90)	124.0(3)
C(36)–C(35)–C(41)	123.1(10)	C(89)–C(90)–C(91)	118.8(4)
C(34)–C(35)–C(41)	118.4(10)	C(92)–C(91)–C(90)	118.2(4)
C(35)–C(36)–C(31)	122.8(11)	C(91)–C(92)–C(93)	119.2(3)
C(39)–C(37)–C(33)	110.8(11)	N(9)–C(93)–C(92)	123.5(4)
C(39)–C(37)–C(38)	114.4(12)	C(94)–N(10)–C(98)	116.5(6)
C(33)–C(37)–C(38)	106.6(10)	C(94)–N(10)–Co(1)	122.7(4)
C(39)–C(37)–C(40)	109.3(10)	C(98)–N(10)–Co(1)	120.2(4)
C(33)–C(37)–C(40)	110.8(9)	N(10)–C(94)–C(95)	122.0(7)
C(38)–C(37)–C(40)	104.8(11)	C(94)–C(95)–C(96)	120.4(7)
C(43)–C(41)–C(42)	111.2(12)	C(97)–C(96)–C(95)	118.3(7)
C(43)–C(41)–C(35)	110.0(13)	C(96)–C(97)–C(98)	119.0(8)
C(42)–C(41)–C(35)	111.3(10)	N(10)–C(98)–C(97)	123.6(6)
C(43)–C(41)–C(44)	106.0(12)	C(94')–N(10')–C(98')	120.0
C(42)–C(41)–C(44)	106.3(13)	N(10')–C(94')–C(95')	120.0
C(35)–C(41)–C(44)	111.9(10)	C(96')–C(95')–C(94')	120.0
C(36')–C(31')–C(32')	121.3(11)	C(97')–C(96')–C(95')	120.0
C(36')–C(31')–C(27)	119.0(11)	C(96')–C(97')–C(98')	120.0
C(32')–C(31')–C(27)	116.9(11)	C(97')–C(98')–N(10')	120.0
C(33')–C(32')–C(31')	118.8(12)		
**Torsion angles**			
C(23)–N(2)–C(1)–N(1)	–9.5(6)	C(36')–C(35')–C(41')–C(42')	–109.9(18)
C(23)–N(2)–C(1)–C(2)	178.1(4)	C(34')–C(35')–C(41')–C(42')	62.6(18)
C(4)–N(1)–C(1)–N(2)	–166.9(3)	C(36')–C(35')–C(41')–C(44')	12(2)
Co(1)–N(1)–C(1)–N(2)	21.9(5)	C(34')–C(35')–C(41')–C(44')	–175.3(14)
C(4)–N(1)–C(1)–C(2)	6.5(4)	C(26)–N(4)–C(45)–N(5)	5.3(7)
Co(1)–N(1)–C(1)–C(2)	–164.7(2)	C(26)–N(4)–C(45)–C(46)	–176.7(4)
N(2)–C(1)–C(2)–C(3)	166.7(4)	C(48)–N(5)–C(45)–N(4)	177.7(4)
N(1)–C(1)–C(2)–C(3)	–6.6(4)	Co(1)–N(5)–C(45)–N(4)	–7.7(6)
N(2)–C(1)–C(2)–C(5)	–11.4(7)	C(48)–N(5)–C(45)–C(46)	–0.5(4)
N(1)–C(1)–C(2)–C(5)	175.3(4)	Co(1)–N(5)–C(45)–C(46)	174.0(2)
C(5)–C(2)–C(3)–C(8)	2.2(6)	N(4)–C(45)–C(46)–C(47)	179.5(4)
C(1)–C(2)–C(3)–C(8)	–176.2(3)	N(5)–C(45)–C(46)–C(47)	–2.2(4)
C(5)–C(2)–C(3)–C(4)	–177.4(3)	N(4)–C(45)–C(46)–C(52)	–4.5(7)

C(1)–C(2)–C(3)–C(4)	4.1(4)	N(5)–C(45)–C(46)–C(52)	173.9(4)
C(70)–N(8)–C(4)–N(1)	9.7(5)	C(52)–C(46)–C(47)–C(49)	3.2(6)
C(70)–N(8)–C(4)–C(3)	–172.2(3)	C(45)–C(46)–C(47)–C(49)	179.8(3)
C(1)–N(1)–C(4)–N(8)	174.5(3)	C(52)–C(46)–C(47)–C(48)	–172.7(4)
Co(1)–N(1)–C(4)–N(8)	–14.2(5)	C(45)–C(46)–C(47)–C(48)	3.9(4)
C(1)–N(1)–C(4)–C(3)	–3.8(4)	C(67)–N(6)–C(48)–N(5)	–5.5(5)
Co(1)–N(1)–C(4)–C(3)	167.5(2)	C(67)–N(6)–C(48)–C(47)	–177.6(3)
C(8)–C(3)–C(4)–N(8)	1.5(6)	C(45)–N(5)–C(48)–N(6)	–169.9(3)
C(2)–C(3)–C(4)–N(8)	–178.9(3)	Co(1)–N(5)–C(48)–N(6)	15.5(5)
C(8)–C(3)–C(4)–N(1)	180.0(3)	C(45)–N(5)–C(48)–C(47)	3.0(4)
C(2)–C(3)–C(4)–N(1)	–0.4(4)	Co(1)–N(5)–C(48)–C(47)	–171.6(2)
C(3)–C(2)–C(5)–C(6)	–2.0(6)	C(49)–C(47)–C(48)–N(6)	–6.4(6)
C(1)–C(2)–C(5)–C(6)	175.9(4)	C(46)–C(47)–C(48)–N(6)	169.0(3)
C(3)–C(2)–C(5)–C(9)	172.0(4)	C(49)–C(47)–C(48)–N(5)	–179.8(4)
C(1)–C(2)–C(5)–C(9)	–10.1(8)	C(46)–C(47)–C(48)–N(5)	–4.4(4)
C(2)–C(5)–C(6)–C(7)	0.8(7)	C(46)–C(47)–C(49)–C(50)	–5.3(6)
C(9)–C(5)–C(6)–C(7)	–173.8(5)	C(48)–C(47)–C(49)–C(50)	169.5(4)
C(5)–C(6)–C(7)–C(8)	0.4(8)	C(46)–C(47)–C(49)–C(53)	173.9(4)
C(6)–C(7)–C(8)–C(3)	–0.3(6)	C(48)–C(47)–C(49)–C(53)	–11.3(6)
C(2)–C(3)–C(8)–C(7)	–1.0(6)	C(47)–C(49)–C(50)–C(51)	3.7(6)
C(4)–C(3)–C(8)–C(7)	178.6(4)	C(53)–C(49)–C(50)–C(51)	–175.5(4)
C(6)–C(5)–C(9)–C(14)	–31.1(7)	C(49)–C(50)–C(51)–C(52)	0.0(8)
C(2)–C(5)–C(9)–C(14)	155.0(5)	C(50)–C(51)–C(52)–C(46)	–2.2(7)
C(6)–C(5)–C(9)–C(10)	142.5(5)	C(47)–C(46)–C(52)–C(51)	0.6(7)
C(2)–C(5)–C(9)–C(10)	–31.4(8)	C(45)–C(46)–C(52)–C(51)	–175.0(4)
C(14)–C(9)–C(10)–C(11)	0.9(8)	C(50)–C(49)–C(53)–C(58)	–58.2(5)
C(5)–C(9)–C(10)–C(11)	–172.8(5)	C(47)–C(49)–C(53)–C(58)	122.6(4)
C(9)–C(10)–C(11)–C(12)	0.7(9)	C(50)–C(49)–C(53)–C(54)	124.1(4)
C(9)–C(10)–C(11)–C(15)	178.7(6)	C(47)–C(49)–C(53)–C(54)	–55.0(5)
C(10)–C(11)–C(12)–C(13)	–0.9(12)	C(58)–C(53)–C(54)–C(55)	–0.6(5)
C(15)–C(11)–C(12)–C(13)	–178.9(8)	C(49)–C(53)–C(54)–C(55)	177.1(3)
C(11)–C(12)–C(13)–C(14)	–0.4(13)	C(53)–C(54)–C(55)–C(56)	–0.8(5)
C(11)–C(12)–C(13)–C(19)	177.0(9)	C(53)–C(54)–C(55)–C(59)	–179.6(3)
C(12)–C(13)–C(14)–C(9)	2.1(12)	C(54)–C(55)–C(56)–C(57)	1.1(6)
C(19)–C(13)–C(14)–C(9)	–175.4(8)	C(59)–C(55)–C(56)–C(57)	179.9(3)
C(10)–C(9)–C(14)–C(13)	–2.3(9)	C(55)–C(56)–C(57)–C(58)	0.1(5)
C(5)–C(9)–C(14)–C(13)	171.4(6)	C(55)–C(56)–C(57)–C(63)	–175.2(3)
C(10)–C(11)–C(15)–C(16')	120.3(18)	C(54)–C(53)–C(58)–C(57)	1.9(5)
C(12)–C(11)–C(15)–C(16')	–61.8(19)	C(49)–C(53)–C(58)–C(57)	–175.8(3)
C(10)–C(11)–C(15)–C(17')	–117.7(17)	C(56)–C(57)–C(58)–C(53)	–1.7(5)
C(12)–C(11)–C(15)–C(17')	60.1(18)	C(63)–C(57)–C(58)–C(53)	173.5(3)
C(10)–C(11)–C(15)–C(16)	53.3(11)	C(54)–C(55)–C(59)–C(62)	1.8(6)
C(12)–C(11)–C(15)–C(16)	–128.8(9)	C(56)–C(55)–C(59)–C(62)	–176.9(4)
C(10)–C(11)–C(15)–C(18)	–69.4(8)	C(54)–C(55)–C(59)–C(61)	–120.5(5)
C(12)–C(11)–C(15)–C(18)	108.5(9)	C(56)–C(55)–C(59)–C(61)	60.7(5)
C(10)–C(11)–C(15)–C(17)	173.4(7)	C(54)–C(55)–C(59)–C(60)	121.7(4)
C(12)–C(11)–C(15)–C(17)	–8.7(11)	C(56)–C(55)–C(59)–C(60)	–57.1(5)
C(10)–C(11)–C(15)–C(18')	–2.3(15)	C(56)–C(57)–C(63)–C(66)	76.8(5)
C(12)–C(11)–C(15)–C(18')	175.5(14)	C(58)–C(57)–C(63)–C(66)	–98.2(5)
C(14)–C(13)–C(19)–C(20)	–57.5(13)	C(56)–C(57)–C(63)–C(65)	–163.9(4)
C(12)–C(13)–C(19)–C(20)	125.2(10)	C(58)–C(57)–C(63)–C(65)	21.0(5)
C(14)–C(13)–C(19)–C(21)	179.8(10)	C(56)–C(57)–C(63)–C(64)	–44.3(5)

C(12)–C(13)–C(19)–C(21)	2.5(16)	C(58)–C(57)–C(63)–C(64)	140.6(4)
C(14)–C(13)–C(19)–C(22)	61.0(13)	C(48)–N(6)–C(67)–N(7)	–9.6(5)
C(12)–C(13)–C(19)–C(22)	–116.3(10)	C(48)–N(6)–C(67)–C(68)	173.4(3)
C(1)–N(2)–C(23)–N(3)	–14.0(6)	C(70)–N(7)–C(67)–N(6)	–174.4(3)
C(1)–N(2)–C(23)–C(24)	174.2(4)	Co(1)–N(7)–C(67)–N(6)	13.1(5)
C(26)–N(3)–C(23)–N(2)	–167.6(4)	C(70)–N(7)–C(67)–C(68)	2.9(3)
Co(1)–N(3)–C(23)–N(2)	23.5(6)	Co(1)–N(7)–C(67)–C(68)	–169.6(2)
C(26)–N(3)–C(23)–C(24)	5.3(5)	N(6)–C(67)–C(68)–C(74)	–2.7(5)
Co(1)–N(3)–C(23)–C(24)	–163.6(3)	N(7)–C(67)–C(68)–C(74)	179.9(3)
N(2)–C(23)–C(24)–C(25)	167.5(4)	N(6)–C(67)–C(68)–C(69)	177.6(3)
N(3)–C(23)–C(24)–C(25)	–5.5(5)	N(7)–C(67)–C(68)–C(69)	0.2(3)
N(2)–C(23)–C(24)–C(27)	–15.3(9)	C(74)–C(68)–C(69)–C(71)	–1.1(5)
N(3)–C(23)–C(24)–C(27)	171.7(6)	C(67)–C(68)–C(69)–C(71)	178.7(3)
C(27)–C(24)–C(25)–C(30)	5.4(9)	C(74)–C(68)–C(69)–C(70)	177.3(3)
C(23)–C(24)–C(25)–C(30)	–177.0(5)	C(67)–C(68)–C(69)–C(70)	–3.0(3)
C(27)–C(24)–C(25)–C(26)	–174.2(5)	C(4)–N(8)–C(70)–N(7)	7.9(4)
C(23)–C(24)–C(25)–C(26)	3.4(5)	C(4)–N(8)–C(70)–C(69)	–180.0(3)
C(45)–N(4)–C(26)–N(3)	6.4(7)	C(67)–N(7)–C(70)–N(8)	168.3(3)
C(45)–N(4)–C(26)–C(25)	–174.0(4)	Co(1)–N(7)–C(70)–N(8)	–19.3(4)
C(23)–N(3)–C(26)–N(4)	176.5(4)	C(67)–N(7)–C(70)–C(69)	–4.8(3)
Co(1)–N(3)–C(26)–N(4)	–14.7(7)	Co(1)–N(7)–C(70)–C(69)	167.62(19)
C(23)–N(3)–C(26)–C(25)	–3.1(5)	C(68)–C(69)–C(70)–N(8)	–168.2(3)
Co(1)–N(3)–C(26)–C(25)	165.7(3)	C(71)–C(69)–C(70)–N(8)	9.8(5)
C(30)–C(25)–C(26)–N(4)	0.5(9)	C(68)–C(69)–C(70)–N(7)	4.9(3)
C(24)–C(25)–C(26)–N(4)	–180.0(4)	C(71)–C(69)–C(70)–N(7)	–177.0(3)
C(30)–C(25)–C(26)–N(3)	–179.9(6)	C(68)–C(69)–C(71)–C(72)	1.5(4)
C(24)–C(25)–C(26)–N(3)	–0.4(6)	C(70)–C(69)–C(71)–C(72)	–176.3(3)
C(25)–C(24)–C(27)–C(28)	–5.8(8)	C(68)–C(69)–C(71)–C(75)	–176.1(3)
C(23)–C(24)–C(27)–C(28)	177.3(6)	C(70)–C(69)–C(71)–C(75)	6.1(5)
C(25)–C(24)–C(27)–C(31')	–179.6(9)	C(69)–C(71)–C(72)–C(73)	–0.9(5)
C(23)–C(24)–C(27)–C(31')	3.5(12)	C(75)–C(71)–C(72)–C(73)	176.8(3)
C(25)–C(24)–C(27)–C(31)	156.9(8)	C(71)–C(72)–C(73)–C(74)	–0.4(5)
C(23)–C(24)–C(27)–C(31)	–20.0(11)	C(72)–C(73)–C(74)–C(68)	0.9(5)
C(24)–C(27)–C(28)–C(29)	2.1(10)	C(69)–C(68)–C(74)–C(73)	–0.2(5)
C(31')–C(27)–C(28)–C(29)	176.0(9)	C(67)–C(68)–C(74)–C(73)	–179.8(3)
C(31)–C(27)–C(28)–C(29)	–162.5(8)	C(72)–C(71)–C(75)–C(80)	52.8(5)
C(27)–C(28)–C(29)–C(30)	2.4(12)	C(69)–C(71)–C(75)–C(80)	–129.7(4)
C(24)–C(25)–C(30)–C(29)	–0.7(10)	C(72)–C(71)–C(75)–C(76)	–127.5(4)
C(26)–C(25)–C(30)–C(29)	178.8(6)	C(69)–C(71)–C(75)–C(76)	50.0(5)
C(28)–C(29)–C(30)–C(25)	–3.1(11)	C(80)–C(75)–C(76)–C(77)	0.6(6)
C(24)–C(27)–C(31)–C(36)	–18.9(17)	C(71)–C(75)–C(76)–C(77)	–179.1(3)
C(28)–C(27)–C(31)–C(36)	143.7(12)	C(75)–C(76)–C(77)–C(78)	–0.3(6)
C(24)–C(27)–C(31)–C(32)	161.1(9)	C(75)–C(76)–C(77)–C(81)	179.9(4)
C(28)–C(27)–C(31)–C(32)	–36.3(14)	C(76)–C(77)–C(78)–C(79)	–0.3(6)
C(36)–C(31)–C(32)–C(33)	–1(2)	C(81)–C(77)–C(78)–C(79)	179.4(4)
C(27)–C(31)–C(32)–C(33)	178.6(11)	C(77)–C(78)–C(79)–C(80)	0.7(6)
C(31)–C(32)–C(33)–C(34)	2.3(19)	C(77)–C(78)–C(79)–C(85)	179.5(4)
C(31)–C(32)–C(33)–C(37)	–173.1(12)	C(76)–C(75)–C(80)–C(79)	–0.2(6)
C(32)–C(33)–C(34)–C(35)	–3.1(19)	C(71)–C(75)–C(80)–C(79)	179.5(3)
C(37)–C(33)–C(34)–C(35)	172.0(12)	C(78)–C(79)–C(80)–C(75)	–0.4(6)
C(33)–C(34)–C(35)–C(36)	3(2)	C(85)–C(79)–C(80)–C(75)	–179.2(4)
C(33)–C(34)–C(35)–C(41)	–172.6(12)	C(76)–C(77)–C(81)–C(83)	–141.6(4)
C(34)–C(35)–C(36)–C(31)	–2(2)	C(78)–C(77)–C(81)–C(83)	38.6(5)
C(41)–C(35)–C(36)–C(31)	173.3(14)	C(76)–C(77)–C(81)–C(82)	98.2(4)
C(32)–C(31)–C(36)–C(35)	1(2)	C(78)–C(77)–C(81)–C(82)	–81.6(5)

C(27)–C(31)–C(36)–C(35)	–178.6(14)	C(76)–C(77)–C(81)–C(84)	–21.6(6)
C(34)–C(33)–C(37)–C(39)	119.7(15)	C(78)–C(77)–C(81)–C(84)	158.6(4)
C(32)–C(33)–C(37)–C(39)	–65.2(16)	C(78)–C(79)–C(85)–C(86')	62.1(11)
C(34)–C(33)–C(37)–C(38)	–115.3(14)	C(80)–C(79)–C(85)–C(86')	–119.1(11)
C(32)–C(33)–C(37)–C(38)	59.8(15)	C(78)–C(79)–C(85)–C(87)	–118.0(6)
C(34)–C(33)–C(37)–C(40)	–1.8(18)	C(80)–C(79)–C(85)–C(87)	60.8(6)
C(32)–C(33)–C(37)–C(40)	173.3(11)	C(78)–C(79)–C(85)–C(88)	120.4(5)
C(36)–C(35)–C(41)–C(43)	120.8(18)	C(80)–C(79)–C(85)–C(88)	–60.8(5)
C(34)–C(35)–C(41)–C(43)	–63.7(16)	C(78)–C(79)–C(85)–C(87')	–65.9(11)
C(36)–C(35)–C(41)–C(42)	–115.4(17)	C(80)–C(79)–C(85)–C(87')	112.8(11)
C(34)–C(35)–C(41)–C(42)	60.1(18)	C(78)–C(79)–C(85)–C(86)	4.8(7)
C(36)–C(35)–C(41)–C(44)	3(2)	C(80)–C(79)–C(85)–C(86)	–176.5(5)
C(34)–C(35)–C(41)–C(44)	178.8(13)	C(78)–C(79)–C(85)–C(88')	–178.5(10)
C(24)–C(27)–C(31')–C(36')	–50.3(18)	C(80)–C(79)–C(85)–C(88')	0.3(11)
C(28)–C(27)–C(31')–C(36')	136.2(14)	C(93)–N(9)–C(89)–C(90)	0.2(7)
C(24)–C(27)–C(31')–C(32')	148.3(10)	Co(1)–N(9)–C(89)–C(90)	176.1(4)
C(28)–C(27)–C(31')–C(32')	–25.2(17)	N(9)–C(89)–C(90)–C(91)	–1.6(9)
C(36')–C(31')–C(32')–C(33')	0(2)	C(89)–C(90)–C(91)–C(92)	2.0(9)
C(27)–C(31')–C(32')–C(33')	161.0(12)	C(90)–C(91)–C(92)–C(93)	–1.1(8)
C(31')–C(32')–C(33')–C(34')	3(2)	C(89)–N(9)–C(93)–C(92)	0.8(6)
C(31')–C(32')–C(33')–C(37')	–177.0(13)	Co(1)–N(9)–C(93)–C(92)	–175.1(3)
C(32')–C(33')–C(34')–C(35')	–3(2)	C(91)–C(92)–C(93)–N(9)	–0.4(7)
C(37')–C(33')–C(34')–C(35')	177.6(13)	C(98)–N(10)–C(94)–C(95)	2.5(14)
C(33')–C(34')–C(35')–C(36')	–1(2)	Co(1)–N(10)–C(94)–C(95)	–168.0(8)
C(33')–C(34')–C(35')–C(41')	–174.4(13)	N(10)–C(94)–C(95)–C(96)	–4.5(18)
C(32')–C(31')–C(36')–C(35')	–4(3)	C(94)–C(95)–C(96)–C(97)	6.0(19)
C(27)–C(31')–C(36')–C(35')	–164.9(15)	C(95)–C(96)–C(97)–C(98)	–5.8(18)
C(34')–C(35')–C(36')–C(31')	5(2)	C(94)–N(10)–C(98)–C(97)	–2.4(14)
C(41')–C(35')–C(36')–C(31')	177.7(15)	Co(1)–N(10)–C(98)–C(97)	168.5(8)
C(34')–C(33')–C(37')–C(39')	178.0(15)	C(96)–C(97)–C(98)–N(10)	4.1(17)
C(32')–C(33')–C(37')–C(39')	–2(2)	C(98')–N(10')–C(94')–C(95')	0.0
C(34')–C(33')–C(37')–C(38')	–57.8(19)	N(10')–C(94')–C(95')–C(96')	0.0
C(32')–C(33')–C(37')–C(38')	122.5(16)	C(94')–C(95')–C(96')–C(97')	0.0

C(34')–C(33')–C(37')–C(40')	56(2)	C(95')–C(96')–C(97')–C(98')	0.0
C(32')–C(33')–C(37')–C(40')	–123.4(15)	C(96')–C(97')–C(98')–N(10')	0.0
C(36')–C(35')–C(41')–C(43')	131.9(18)	C(94')–N(10')–C(98')–C(97')	0.0
C(34')–C(35')–C(41')–C(43')	–55.6(18)		

Liposomal stability

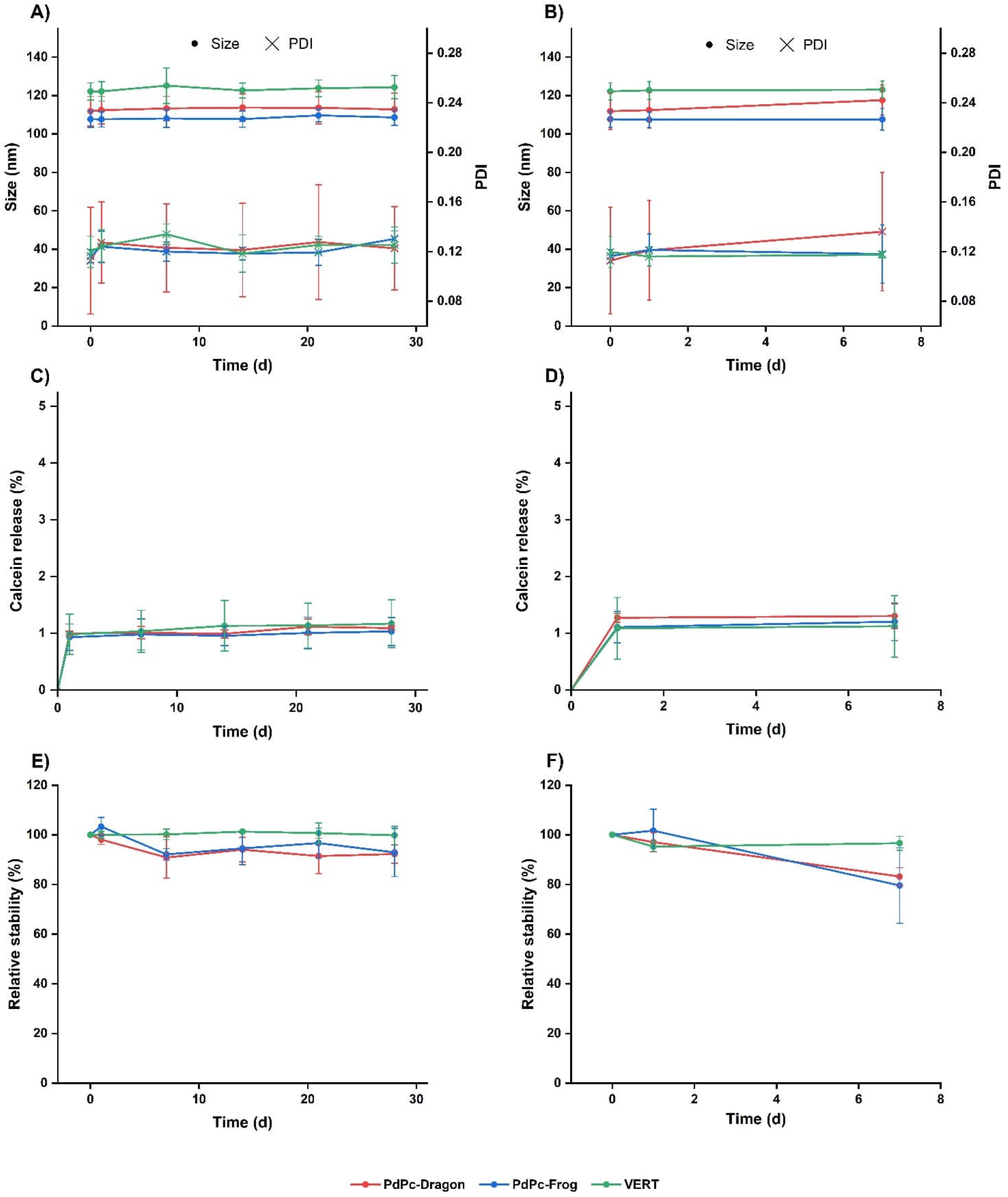

Figure S86: Stability of liposomes during a 30-day storage period at 4 °C (left), and a 7-day storage period at 37 °C (right). The stability assessment was based on the particle size and PDI of liposomes (A, B), and on the passive leakage of calcein from liposomes, indicating the integrity of the liposomal lipid bilayer (C, D). Also, the stability of the photosensitizers in liposomes was determined from their absorbance at the wavelength of maximum absorption, for which pyridine was added into the liposome solution at a 3:1 (V/V) ratio (E, F).

PdPc stability in serum

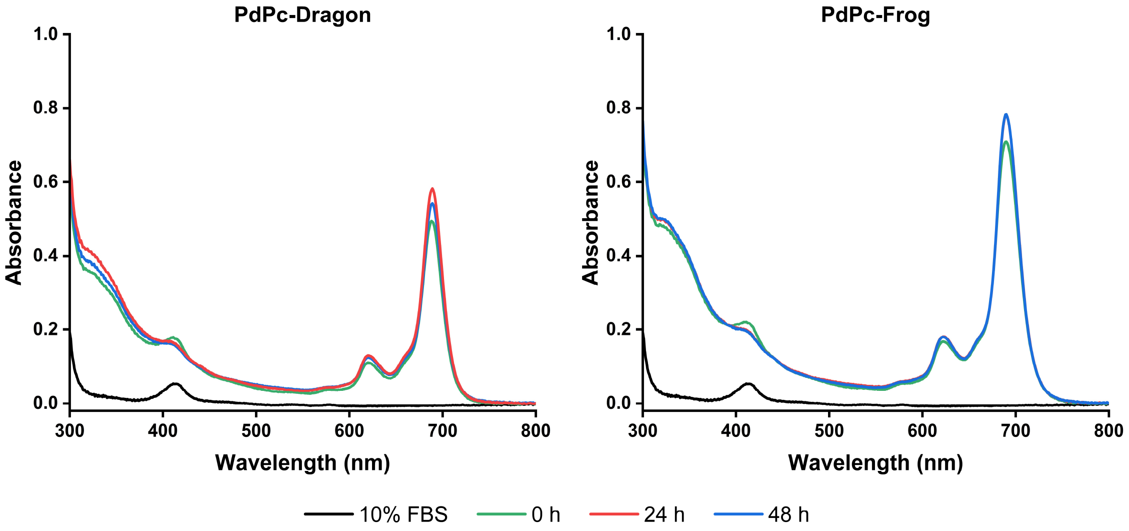

Figure S87: Stability of PdPc-Dragon and PdPc-Frog in liposomes in 10% fetal bovine serum (FBS) during 48-hour incubation at 37 ^{o}C under continuous shaking (250 rpm). The absorbance spectra of the samples were measured at several time points. The absorption spectra of 100% FBS were also recorded.

Calcein release in the presence of an oxygen scavenger

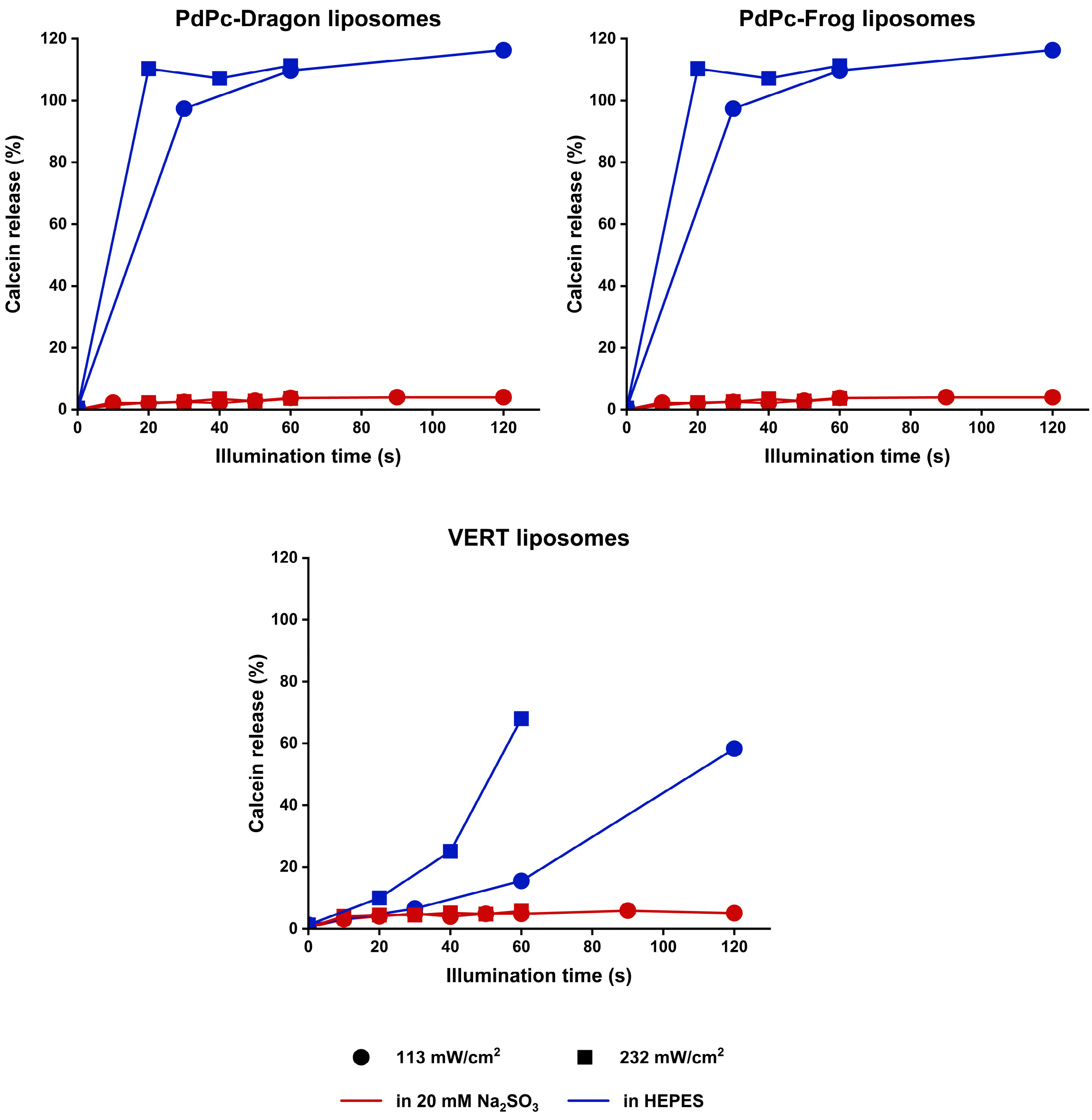

Figure S88. The relative calcein release from PdPc and VERT liposomes at power densities of 113 mW/cm^2 (circles) and 232 mW/cm^2 (squares) with a wavelength of 690 nm to demonstrate ROS-dependent cargo release. The illumination was performed in the presence of 20 mM Na_2SO_3 to create oxygen-depleted conditions (red lines). Controls were illuminated in HEPES buffer (blue lines). Na_2SO_3 had no effect on liposome particle size, solution pH, or photosensitizer absorbance (data not shown).

References

1 R. N. Da Silva, Â. Cunha and A. C. Tomé, *Eur. J. Med. Chem.*, 2018, **154**, 60–67.
2 S. Vagin and M. Hanack, *Eur. J. Org. Chem.*, 2004, **2004**, 600–606.
3 T. Kimura, C. Kudo and S. Nakajo, *Eur. J. Inorg. Chem.*, 2019, **2019**, 4006–4013.
4 P. Zimcik, A. Malkova, L. Hruba, M. Miletin and V. Novakova, *Dyes Pigments*, 2017, **136**, 715–723.
5 E. F. A. Carvalho, M. J. F. Calvete, A. C. Tomé and J. A. S. Cavaleiro, *Tetrahedron Lett.*, 2009, **50**, 6882–6885.
6 A. Tillo, M. Stolarska, M. Kryjewski, L. Popenda, L. Sobotta, S. Jurga, J. Mielcarek and T. Goslinski, *Dyes Pigments*, 2016, **127**, 110–115.
7 J. Szymczak, T. Rebis, M. Kotkowiak, B. Wicher, L. Sobotta, E. Tykarska, J. Mielcarek and M. Kryjewski, *Dyes Pigments*, 2021, **191**, 109357.
8 D. F. O'Shea, M. A. Miller, H. Matsueda and J. S. Lindsey, *Inorg. Chem.*, 1996, **35**, 7325–7338.
9 K. D. Mulholland, S. Yoon, C. C. Rennie, E. K. Sitch, A. I. McKay, K. Edkins and R. M. Edkins, *Chem. Commun.*, 2020, **56**, 8452–8455.
10 A. L. Spek, *Acta Crystallogr. Sect. C Struct. Chem.*, 2015, **71**, 9–18.
